# National trends in total cholesterol obscure heterogeneous changes in HDL and non-HDL cholesterol and total-to-HDL cholesterol ratio: a pooled analysis of 458 population-based studies in Asian and Western countries

**DOI:** 10.1093/ije/dyz099

**Published:** 2019-07-18

**Authors:** NCD Risk Factor Collaboration (NCD-RisC), Cristina Taddei, Rod Jackson, Bin Zhou, Honor Bixby, Goodarz Danaei, Mariachiara Di Cesare, Kari Kuulasmaa, Kaveh Hajifathalian, James Bentham, James E Bennett, Wichai Aekplakorn, Renata Cifkova, Jean Dallongeville, Dirk DeBacquer, Simona Giampaoli, Vilmundur Gudnason, Young-Ho Khang, Tiina Laatikainen, JimI Mann, Pedro Marques-Vidal, George A Mensah, Martina Müller-Nurasyid, Toshiharu Ninomiya, Janina Petkeviciene, Fernando Rodríguez-Artalejo, Jennifer Servais, Stefan Söderberg, Bill Stavreski, Tom Wilsgaard, Tomasz Zdrojewski, Dong Zhao, Gretchen A Stevens, Stefan Savin, Melanie J Cowan, Leanne M Riley, Majid Ezzati, Robert J Adams, Wichai Aekplakorn, Wolfgang Ahrens, Philippe Amouyel, Antoinette Amuzu, Sigmund A Anderssen, Inger Ariansen, Dominique Arveiler, Thor Aspelund, Juha Auvinen, Mária Avdicová, Maciej Banach, Piotr Bandosz, José R Banegas, Carlo M Barbagallo, Iqbal Bata, Louise A Baur, Robert Beaglehole, James E Bennett, Gailute Bernotiene, Yufang Bi, Asako Bienek, Cecilia Björkelund, Simona Bo, Bernhard O Boehm, Marialaura Bonaccio, Vanina Bongard, Rossana Borchini, Herman Borghs, Juergen Breckenkamp, Hermann Brenner, Graziella Bruno, Markus A Busch, Antonio Cabrera de León, Vincenzo Capuano, Felipe F Casanueva, Juan-Pablo Casas, Carmelo A Caserta, Laura Censi, Fangfang Chen, Shuohua Chen, María-Dolores Chirlaque, Belong Cho, Yumi Cho, Jerzy Chudek, Renata Cifkova, Frank Claessens, Janine Clarke, Els Clays, Cyrus Cooper, Simona Costanzo, Dominique Cottel, Chris Cowell, Ana B Crujeiras, Liufu Cui, Graziella D'Arrigo, Jean Dallongeville, Luc Dauchet, Guy De Backer, Dirk De Bacquer, Giovanni de Gaetano, Stefaan De Henauw, Delphine De Smedt, Elaine Dennison, Valérie Deschamps, Augusto DiCastelnuovo, Annette J Dobson, Chiara Donfrancesco, Angela Döring, Wojciech Drygas, Yong Du, Elzbieta Dziankowska-Zaborszczyk, Robert Eggertsen, Ulf Ekelund, Roberto Elosua, Johan G Eriksson, Alun Evans, David Faeh, Francisco J Felix-Redondo, Daniel Fernández-Bergés, Marika Ferrari, Jean Ferrieres, Joseph D Finn, Ann-Sofie Forslund, Maria Forsner, Guillermo Frontera, Yuki Fujita, Zbigniew Gaciong, Fabio Galvano, Jingli Gao, Manoli Garcia-de-la-Hera, Sarah P Garnett, Jean-Michel Gaspoz, Magda Gasull, Louise Gates, Simona Giampaoli, Francesco Gianfagna, Tiffany K Gill, Jonathan Giovannelli, David Goltzman, Marcela GonzalezGross, Frederic Gottrand, Sidsel Graff-Iversen, Dušan Grafnetter, Ronald D Gregor, Tomasz Grodzicki, Giuseppe Grosso, Grabriella Gruden, Dongfeng Gu, Pilar Guallar-Castillón, Elias F Gudmundsson, Vilmundur Gudnason, Idris Guessous, Johanna Gunnlaugsdottir, Felix Gutzwiller, Rebecca Hardy, Jun Hata, Teresa Haugsgjerd, Alison J Hayes, Jiang He, Yuna He, Sauli Herrala, Ilpo TapaniHihtaniemi, Michael Hobbs, Wilma M Hopman, José MaríaHuerta, Inge Huybrechts, Licia Iacoviello, Anna G Iannone, Nayu Ikeda, Masanori Iwasaki, Rod Jackson, Konrad Jamrozik, Imre Janszky, Marjo-Riitta Jarvelin, Grazyna Jasienska, Garry Jennings, Seung-lyeal Jeong, Chao QiangJiang, Michel Joffres, Jari J Jokelainen, Jost B Jonas, Jacek Jóźwiak, Eero O Kajantie, Jussi Kauhanen, Ulrich Keil, Sirkka Keinänen-Kiukaanniemi, Mathilde Kersting, Young-Ho Khang, Ursula Kiechl-Kohlendorfer, Stefan Kiechl, Jeongseon Kim, Yeon-Yong Kim, Jurate Klumbiene, Michael Knoflach, Stephanie Ko, Elin Kolle, Raija Korpelainen, Seppo Koskinen, Katsuyasu Kouda, Wolfgang Kratzer, Susi Kriemler, Steinar Krokstad, Urho M Kujala, Pawel Kurjata, Kari Kuulasmaa, Tiina Laatikainen, Tai HingLam, Vera Lanska, Georg Lappas, Lars E Laugsand, Jeonghee Lee, Terho Lehtimäki, Yanping Li, Christa L Lilly, Xu Lin, Lars Lind, Lauren Lissner, Jing Liu, Esther Lopez-Garcia, Roberto Lorbeer, José EugenioLozano, Dalia Luksiene, Annamari Lundqvist, Robert Lundqvist, Per Lytsy, Guansheng Ma, Suka Machi, Stefania Maggi, Dianna J Magliano, JimI Mann, Enzo Manzato, Pedro Marques-Vidal, Ellisiv B Mathiesen, Stela McLachlan, Rachael M McLean, Scott B McLean, Aline Meirhaeghe, Christa Meisinger, Patricia Metcalf, Jie Mi, Jody C Miller, Luis A Moreno, Suzanne Morin, Malgorzata Mossakowska, Maria L Muiesan, Martina Müller-Nurasyid, Jaakko Mursu, Harunobu Nakamura, Jana Námešná, Matthias Nauck, Eva MariaNavarrete-Muñoz, William A Neal, Ilona Nenko, Teemu J Niiranen, Guang Ning, Toshiharu Ninomiya, Marianna Noale, Sawada Norie, Davide Noto, Terence O'Neill, Dermot O'Reilly, Kyungwon Oh, Örn Olafsson, Fred MichelPaccaud, Andrzej Pajak, Luigi Palmieri, Francesco Panza, Winsome R Parnell, Markku Peltonen, Annette Peters, Astrid Petersmann, Janina Petkeviciene, Iris Pigeot, Lorenza Pilotto, Aleksandra Piwonska, Pedro Plans-Rubió, Miquel Porta, Jacqueline F Price, Jardena J Puder, Soile E Puhakka, Ricardas Radisauskas, Olli Raitakari, Rafel Ramos, Josep Redon, Fernando Rigo, Fernando Rodríguez-Artalejo, MaríadelCristo Rodriguez-Perez, Dora Romaguera, Kimmo Ronkainen, Annika Rosengren, Joel G R Roy, Jean-Bernard Ruidavets, Marcin Rutkowski, Benoit Salanave, Diego Salmerón, Veikko Salomaa, Jukka T Salonen, Massimo Salvetti, Susana Sans, Jouko L Saramies, Kai-Uwe Saum, Christa Scheidt-Nave, Anja Schienkiewitz, Sabine Schipf, Carsten O Schmidt, Ben Schöttker, Sylvain Sebert, Abhijit Sen, Jennifer Servais, Jonathan E Shaw, Kenji Shibuya, Dong WookShin, Rahman Shiri, Judith Simons, Leon A Simons, Michael Sjöström, Jolanta Slowikowska-Hilczer, Przemyslaw Slusarczyk, Stefan Söderberg, Vincenzo Solfrizzi, Emily Sonestedt, Aicha Soumare, Jan A Staessen, Maria G Stathopoulou, Bill Stavreski, Jostein Steene-Johannessen, Peter Stehle, Jutta Stieber, Doris Stöckl, Jakub Stokwiszewski, Johan Sundström, Paibul Suriyawongpaisal, Abdonas Tamosiunas, Eng JooTan, Anne Taylor, Grethe Tell, Lutgarde Thijs, HannaK Tolonen, Roman Topór-Madry, María JoséTormo, Maties Torrent, Shoichiro Tsugane, Tomi-Pekka Tuomainen, Jaakko Tuomilehto, Christophe Tzourio, Hannu M T Uusitalo, Koen Van Herck, Dirk Vanderschueren, Diego Vanuzzo, Lars Vatten, Tomas Vega, Giovanni Veronesi, Jesus Vioque, JyrkiK Virtanen, Sophie Visvikis-Siest, Peter Vollenweider, Sari Voutilainen, Martine Vrijheid, Aline Wagner, Anne Wagner, Ming-Dong Wang, Qian Wang, Ya XingWang, S Goya Wannamethee, Wenbin Wei, Peter H Whincup, Andrzej Wiecek, Johann Willeit, Peter Willeit, Tom Wilsgaard, Bogdan Wojtyniak, Andrew Wong, Mark Woodward, Aleksander GiwercmanWu, Frederick C Wu, Shouling Wu, Haiquan Xu, Liang Xu, Weili Yan, Xiaoguang Yang, Xingwang Ye, Akihiro Yoshihara, Sabina Zambon, Tomasz Zdrojewski, Dong Zhao, Wenhua Zhao

**Keywords:** Total cholesterol, LDL cholesterol, HDL cholesterol, blood lipids, multi-country study

## Abstract

**Background:**

Although high-density lipoprotein (HDL) and non-HDL cholesterol have opposite associations with coronary heart disease, multi-country reports of lipid trends only use total cholesterol (TC). Our aim was to compare trends in total, HDL and non-HDL cholesterol and the total-to-HDL cholesterol ratio in Asian and Western countries.

**Methods:**

We pooled 458 population-based studies with 82.1 million participants in 23 Asian and Western countries. We estimated changes in mean total, HDL and non-HDL cholesterol and mean total-to-HDL cholesterol ratio by country, sex and age group.

**Results:**

Since ∼1980, mean TC increased in Asian countries. In Japan and South Korea, the TC rise was due to rising HDL cholesterol, which increased by up to 0.17 mmol/L per decade in Japanese women; in China, it was due to rising non-HDL cholesterol. TC declined in Western countries, except in Polish men. The decline was largest in Finland and Norway, at ∼0.4 mmol/L per decade. The decline in TC in most Western countries was the net effect of an increase in HDL cholesterol and a decline in non-HDL cholesterol, with the HDL cholesterol increase largest in New Zealand and Switzerland. Mean total-to-HDL cholesterol ratio declined in Japan, South Korea and most Western countries, by as much as ∼0.7 per decade in Swiss men (equivalent to ∼26% decline in coronary heart disease risk per decade). The ratio increased in China.

**Conclusions:**

HDL cholesterol has risen and the total-to-HDL cholesterol ratio has declined in many Western countries, Japan and South Korea, with only a weak correlation with changes in TC or non-HDL cholesterol.

**Key Messages**

- Total cholesterol (TC) has increased in Asian countries. In Japan and South Korea, the TC rise was largely due to an increase in HDL cholesterol; in China, it was due to a rise in non-HDL cholesterol.
- The observed decline in TC in most Western countries was the net effect of an increase in HDL cholesterol and a decline in non-HDL cholesterol.
- The total-to-HDL cholesterol ratio has declined in many Western countries, Japan and South Korea, with only a weak correlation with changes in TC or non-HDL cholesterol.
- Countries’ comparative performance in reducing the risks associated with blood lipids is only partially captured by trends in TC.

## Introduction

Blood cholesterol is one of the most important risk factors for coronary heart disease (CHD).^1–4^ Population-level data on blood cholesterol are an important input for planning and evaluating the impacts of public health interventions and treatment programmes on entire countries and communities. Comparable data in different countries can help to benchmark success in lowering cholesterol across countries and to understand the reasons behind different trends, both those that were a result of active interventions and unplanned secular changes in nutrition and health behaviours.

Multi-country reporting of lipid trends has so far been based on total cholesterol (TC).^5^^,^^6^ However, high-density lipoprotein (HDL) and non-HDL or low-density lipoprotein (LDL) cholesterol have opposite associations with CHD^1^^,^^2^ and can respond differently to changes in diet and treatment. Currently, there are no comparable cross-country data on lipid fractions, including LDL and HDL cholesterol, and the total-to-HDL cholesterol ratio; only studies in individual countries have reported such trends.^7–27^ To fill this important gap, we used population-based data to analyse and compare long-term changes in TC, HDL and non-HDL cholesterol, and the total-to-HDL cholesterol ratio in Western and Asian countries over a period of more than 30 years.

## Methods

### Primary outcomes

For this analysis, we used mean total, HDL and non-HDL cholesterol and mean total-to-HDL cholesterol ratio as primary outcomes. The hazardous effects of blood cholesterol on CHD were first established in the Framingham Study, focusing on TC.^28^ However, physiological studies^29^ and subsequent analyses of the Framingham Study^30^ found that the fractions of blood cholesterol carried by different lipoproteins and lipid ratios affect CHD risk differentially, and at times in opposite directions. Pooled analyses of observational epidemiological studies have established that CHD risk is associated directly with LDL and non-HDL cholesterol and inversely with HDL cholesterol.^1^^,^^2^ As a result, lipid ratios such as the total-to-HDL cholesterol ratio, which incorporates information on lipid fractions with opposite associations, have emerged as a particularly good predictor of CHD risk in clinical and epidemiological applications.^1^^,^^2^ Randomized clinical trials have also shown that lowering LDL and non-HDL cholesterol lowers CHD risk.^31–34^ In contrast, the results of observational studies on HDL cholesterol have not been replicated in randomized trials or in Mendelian randomization studies.^35–38^

We used non-HDL cholesterol rather than LDL cholesterol because most studies in our analysis had measured TC and HDL cholesterol, from which non-HDL cholesterol can be calculated by subtraction. In contrast, LDL cholesterol was directly measured in only 13% of studies. When LDL cholesterol is not directly measured, its estimation requires data on triglycerides, which were available in only 61% of studies. Further, the most commonly used estimation method, i.e. the Friedewald equation, can be inaccurate.^39^ We found that non-HDL and LDL cholesterol were correlated in studies with data on both variables (*r* = 0.93) (Supplementary Figure 1, available as Supplementary data at *IJE* online). Non-HDL cholesterol predicts CHD risk at least as well as LDL cholesterol^40^^,^^41^ because it includes cholesterol in LDL, lipoprotein(a), intermediate-density lipoprotein, very-low-density lipoprotein and lipoprotein remnants, and is thus a simple measure of cholesterol content within all atherogenic lipoproteins.

### Countries analysed

Our analyses included Asian and Western countries that had at least five population-based studies (or at least three if the studies were nationally representative) in the Non-Communicable Disease Risk Factor Collaboration (NCD-RisC) database, as described below, with measurement of total and HDL cholesterol over a period of at least 15 years from 1970 onwards, with at least one data source after 2005. Twenty-one countries, listed below, met these criteria:

- Nordic countries: Finland, Iceland and Norway.
- Eastern central Europe: Czech Republic, Lithuania, Poland and Slovakia.
- Western central Europe: Belgium, Germany and Switzerland.
- Southern Europe: France, Italy and Spain.
- High-income English-speaking countries: Australia, Canada, New Zealand, UK and USA.
- East and southeast Asia: China, Japan and South Korea.

Two additional countries, Sweden and Thailand, had sufficient data on TC but not on HDL cholesterol and were included in TC analysis only.

### Data sources

We used studies that had measured cholesterol in representative samples of the national population or of one or more subnational regions and communities. We used a database on cardiometabolic risk factors collated by NCD-RisC. NCD-RisC is a worldwide network of health researchers and practitioners whose aim is to document systematically worldwide trends and variations in NCD risk factors.^42–45^ The database was collated through multiple routes for identifying and accessing data. We accessed publicly available population-based measurement surveys [e.g. Demographic and Health Surveys (DHS), Global School-based Student Health Surveys (GSHS), the European Health Interview and Health Examination Surveys (EHIS and EHES) and those available via the Inter-university Consortium for Political and Social Research (ICPSR)]. We requested, via the World Health Organization (WHO) and its regional and country offices, help with identification and access to population-based surveys from ministries of health and other national health and statistical agencies. Requests were also sent via the World Heart Federation to its national partners. We made similar requests to the co-authors of an earlier pooled analysis of cardiometabolic risk factors^5^^,^^46–48^ and invited them to reanalyse data from their studies and join NCD-RisC. Finally, to identify major sources not accessed through the above routes, we searched and reviewed published studies as detailed previously^42–44^ and invited all eligible studies to join NCD-RisC.

Anonymized individual record data from sources included in NCD-RisC were reanalysed by the Pooled Analysis and Writing Group or by data holders according to a common protocol. Within each survey, we included participants aged 18 years and older who were not pregnant. We dropped participants with implausible cholesterol levels (defined as TC <1.75 or >20 mmol/L; HDL cholesterol <0.4 or >5 mmol/L; TC values < HDL values) (<0.1% of all subjects). To ensure summaries were prepared according to the study protocol, the Pooled Analysis and Writing Group provided computer code to NCD-RisC members who requested assistance. All submitted data were checked by at least two independent members of the Pooled Analysis and Writing Group. Questions and clarifications were discussed with NCD-RisC members and resolved before data were incorporated into the database. Finally, we incorporated all nationally representative data from sources that were identified but not accessed via the above routes, by extracting summary statistics from published reports. Data were extracted from published reports only when reported by sex and in age groups no wider than 20 years. We also used data from a previous global data pooling study^5^ when such data had not been accessed through the routes described.

All NCD-RisC members are asked periodically to review the list of sources from their country, to suggest additional sources not in the database and to verify that the included data meet the inclusion criteria listed below and are not duplicates. The NCD-RisC database is continuously updated through this contact with NCD-RisC members and all the above routes. For this paper, we used data from the NCD-RisC database for the 23 countries included in the analysis, for years 1970–2018 and ages 40–79 years.

### Data inclusion and exclusion

Data sources were included in the NCD-RisC lipids database if:

- measured data on total, LDL, HDL cholesterol or triglycerides were available;
- study participants were 10 years of age or older;
- data were collected using a probabilistic sampling method with a defined sampling frame;
- data were from population samples at the national, subnational (i.e. covering one or more subnational regions, more than three urban communities or more than five rural communities) or community level.

We excluded all data sources that included only hypercholesterolemia or dyslipidaemia diagnosis history or medication status without measurement of at least one of the above biomarkers. We also excluded data sources on population subgroups whose lipid profile may differ systematically from the general population, including:

- studies that had included or excluded people based on their health status or cardiovascular risk;
- studies whose participants were only ethnic minorities;
- specific educational, occupational or socio-economic subgroups, with the exception noted below; and
- those recruited through health facilities, with the exception noted below.

We used data whose sampling frame was health insurance schemes in countries where at least 80% of the population were insured. Finally, we used data collected through general practice and primary care systems in high-income and central European countries with universal insurance because contact with the primary care systems tends to be as good as, or better than, response rates for population-based surveys.

We used data sources regardless of fasting status because the differences between fasting and non-fasting measurements are negligible for our primary outcomes.^49^ From the CDC-NHLBI Lipid Standardization Program in the 1950s, there has been an understanding of the need for, and systematic efforts to achieve, standardization of lipid measurements. The difference between any standardized method and the CDC Reference method should be less than 3% for TC and less than 5% for HDL cholesterol (less than 10% before the mid-1990s).^50^ More than three-quarters of the studies in our analysis participated in a lipid standardization programme (Supplementary Table 1 and Supplementary Figure 2, available as Supplementary data at *IJE* online). A summary of data available by country is shown in Supplementary Table 2, available as Supplementary data at *IJE* online, and characteristics of each study are shown in Supplementary Table 1, available as Supplementary data at *IJE* online.

We extracted data for ages 40–79 years because people aged below 40 years have a lower cardiovascular risk and because data in older ages were available in fewer surveys. CHD mortality increases with age whereas hazard ratios for the effects of cholesterol on CHD decrease with age.^1^^,^^2^ As a result, a larger share of CHD deaths are attributable to elevated cholesterol in middle-older ages, but the number of cholesterol-attributable deaths continues to increase with age.^4^ We present results for 40–59 years as the primary analysis because data on these age groups were available for all countries included in the analysis. To investigate the role of age in our findings, we compared results for ages 40–59 years to those of 60–79 years in countries with data for the entire age range of 40–79 years.

### Statistical methods

For each study, we calculated mean total, HDL and non-HDL cholesterol and mean total-to-HDL cholesterol ratio by sex and 10-year age groups. The total-to-HDL cholesterol ratio was calculated using individual records before averaging for each sex and age group. All analyses incorporated appropriate complex survey design and survey sample weights in calculating age- and sex-specific means.

For each primary outcome and for each country, sex and age group, we calculated average annual change over the entire period of data availability by fitting a linear regression with the study-specific means as the dependent variable and year as the independent variable. Each data point was weighted by the inverse of the square of its standard error, so that larger studies had more influence on the estimated change. We multiplied the slope of the fitted line by 10 to calculate average change per decade. We also used the fitted line to estimate total, HDL and non-HDL cholesterol and the total-to-HDL cholesterol ratio values for a consistent period of 1980–2015 for all countries. For countries with data starting before 1980 and/or ending after 2015, this is equivalent to using the fitted line to interpolate for 1980 and/or 2015; for those with data starting after 1980 and/or ending before 2015, values for 1980 and/or 2015 were extrapolated using the fitted line. In a sensitivity analysis, we fitted a non-linear (LOESS) regression to examine by how much our results are influenced by use of linear trend. For each primary outcome and for each country, results were calculated by 10-year age groups, separately for men and women, and then age-standardized into two age bands (40–59 and 60–79 years) by taking a weighted average of age-specific results using weights from the WHO standard population. Analyses were performed in R version 3.4.0 (The R Foundation for Statistical Computing).

## Results

### Data availability

We used 438 population-based studies, collected from 1970 to 2018 in 21 countries that met our inclusion criteria for TC as well as lipid fractions. An additional 20 studies were used for analysis of TC in two additional countries (Thailand and Sweden). Together, these studies included blood lipid measurements in 82.1 million participants, 79 million of whom were aged 40–79 years. The number of data sources ranged from 5 in Slovakia to 56 in Japan. The average time between the first and last studies in a country was around three decades. For the primary analysis, we used 425 studies with data for ages 40–59 years. All these 425 studies had data on TC. In the 21 countries included in the analysis of lipid fractions, 368 of 405 studies (90.9%) had data on HDL cholesterol and 367 (90.6%) on the total-to-HDL cholesterol ratio. Details of data availability by country and characteristics of each study are shown in Supplementary Tables 1 and 2, available as Supplementary data at *IJE* online.

### Total cholesterol

**Figure 1.**
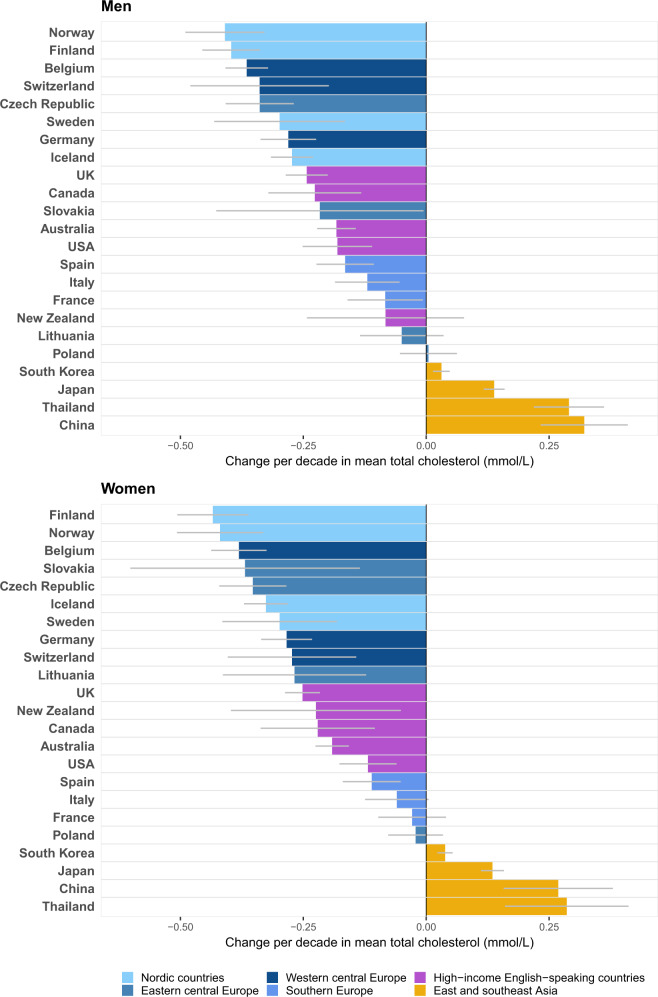
Change per decade in mean total cholesterol by sex in people aged 40–59 years. Results for each country apply to its period of total cholesterol data availability (Supplementary Table 2, available as Supplementary data at *IJE* online). See Supplementary Table 3, available as Supplementary data at *IJE* online, for numerical results and 95% confidence intervals.

Mean TC declined in men and women aged 40–59 years in most Western countries, except in Polish men, whose TC was about the same at the beginning and end of the analysis period (Figure 1). The absence of long-term change in Poland was a result of a rise in mean TC until the late 1990s, followed by a decline (Supplementary Figure 3, available as Supplementary data at *IJE* online). In both sexes, the decline was larger in Nordic countries and central Europe than in English-speaking countries and southern Europe. The TC decline in men ranged from <0.1 mmol/L per decade in Lithuania, New Zealand and France to ∼0.4 mmol/L per decade in Norway, Finland and Belgium. In women, the range was from <0.1 mmol/L per decade in Poland, France and Italy to ∼0.4 mmol/L per decade in Finland, Norway and Belgium. TC increased in all four Asian countries, with the largest increase in China and Thailand, by ∼0.3 mmol/L per decade. Despite this rise, Chinese women (but not men) still had the lowest estimated mean TC of all 23 countries in 2015 (5.0 mmol/L) (Supplementary Figure 4, available as Supplementary data at *IJE* online). The highest mean TCs in 2015 were those in Lithuanian and French men and Thai women, all above 5.7 mmol/L.

### HDL and non-HDL cholesterol

**Figure 2.**
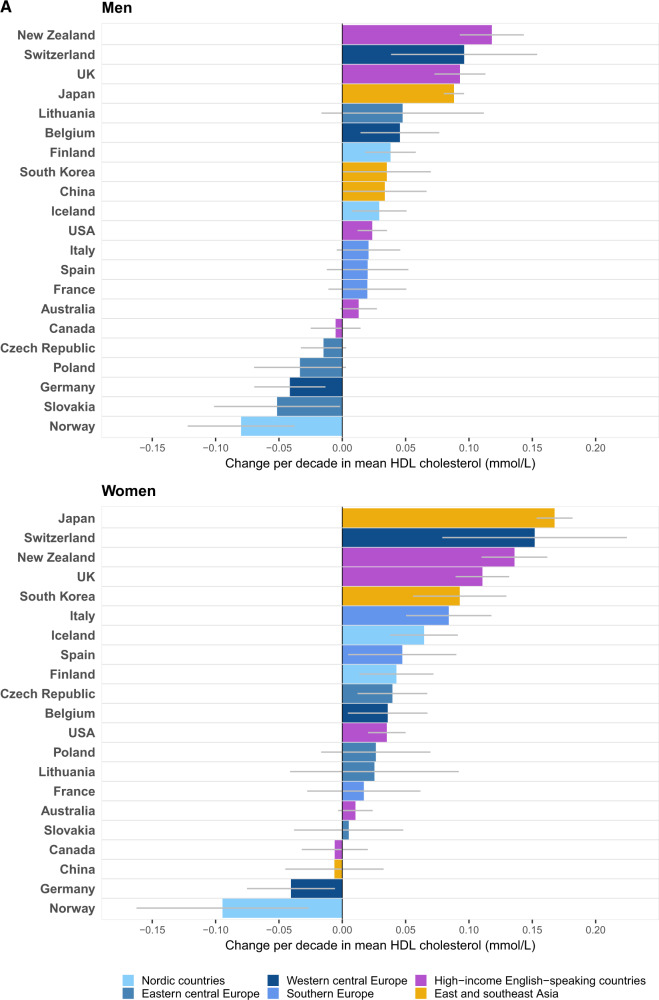

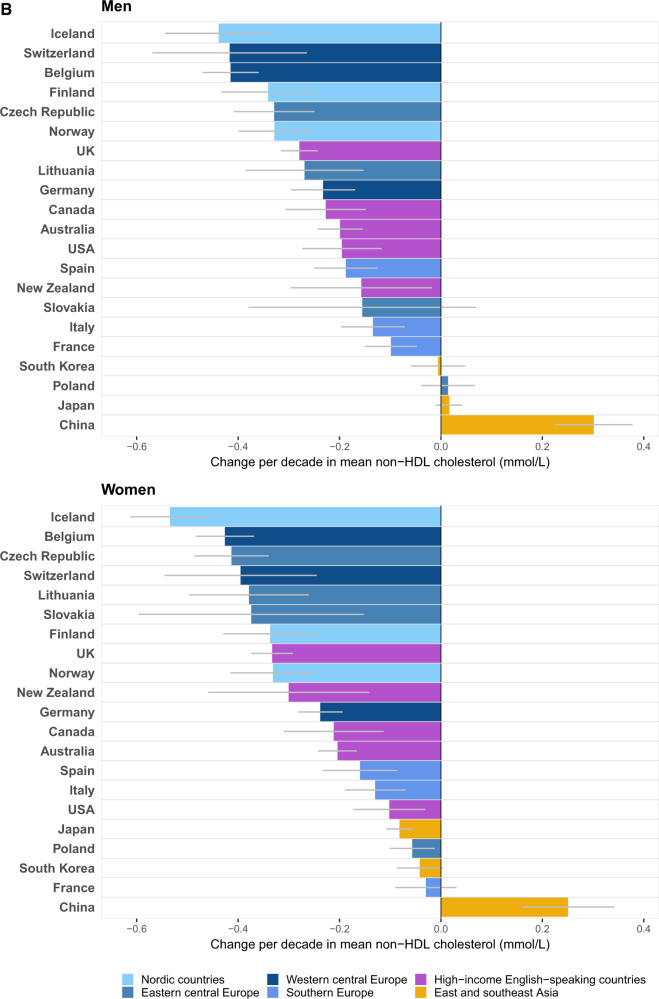
Change per decade in mean (**A**) HDL and (**B**) non-HDL cholesterol by sex in people aged 40–59 years. Results for each country apply to its period of HDL and non-HDL cholesterol data availability (Supplementary Table 2, available as Supplementary data at *IJE* online). See Supplementary Table 4, available as Supplementary data at *IJE* online, for numerical results and 95% confidence intervals.

Among the three Asian countries with data on lipid fractions, the rise in mean TC in Japan and South Korea was largely due to an increase in mean HDL cholesterol, which, among Japanese and South Korean women, was offset partly by a decline in non-HDL cholesterol (Figure 2). The rise in HDL cholesterol ranged from 0.04 mmol/L per decade in South Korean men to 0.17 mmol/L per decade in Japanese women. In contrast, the TC rise in China was due to an increase in non-HDL cholesterol whereas HDL cholesterol remained unchanged in women and increased slightly in men.

The decline in mean TC in many Western countries was the net effect of a decline in non-HDL cholesterol and an increase in HDL cholesterol (Figure 2). The key exceptions were men and women in Germany and Norway, and men in the Czech Republic and Slovakia, where both HDL and non-HDL cholesterol declined. Similar to TC, mean non-HDL cholesterol generally declined more in Nordic countries and central Europe than in English-speaking and southern European countries. The largest rise in mean HDL cholesterol occurred in New Zealand and Switzerland, by 0.10–0.15 mmol/L per decade in the two sexes.

**Figure 3.**
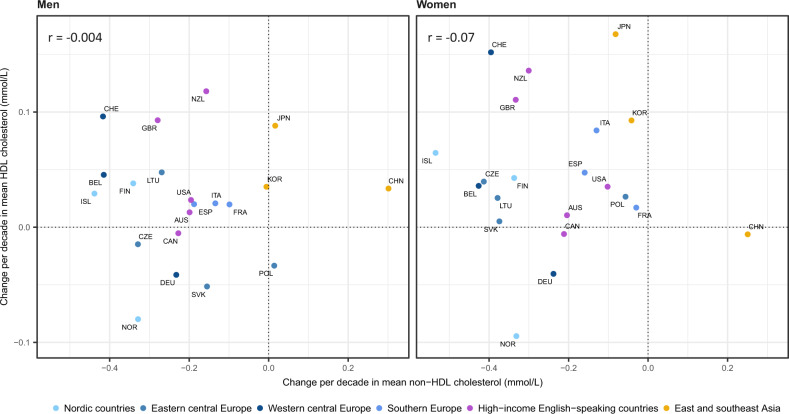
Change per decade in mean non-HDL vs HDL cholesterol in people aged 40–59 years. Results for each country apply to its period of HDL and non-HDL cholesterol data availability (Supplementary Table 2, available as Supplementary data at *IJE* online). See Supplementary Table 4, available as Supplementary data at *IJE* online, for numerical results and 95% confidence intervals. AUS, Australia; BEL, Belgium; CAN, Canada; CHE, Switzerland; CHN, China; CZE, Czech Republic; DEU, Germany; ESP, Spain; FIN, Finland; FRA, France; GBR, United Kingdom; ISL, Iceland; ITA, Italy; JPN, Japan; KOR, South Korea; LTU, Lithuania; NOR, Norway; NZL, New Zealand; POL, Poland; SVK, Slovakia; USA, United States of America.

The change in mean HDL cholesterol and change in mean non-HDL cholesterol were not correlated (*r* = –0.004 for men and –0.07 for women) (Figure 3). In 2015, the lowest levels of mean non-HDL cholesterol were those in China and Belgium for men (3.7 mmol/L) and in Iceland for women (3.3 mmol/L) (Supplementary Figure 5, available as Supplementary data at *IJE* online). The highest were in France: 4.4 mmol/L for men and 4.0 mmol/L for women.

### Total-to-HDL cholesterol ratio

**Figure 4.**
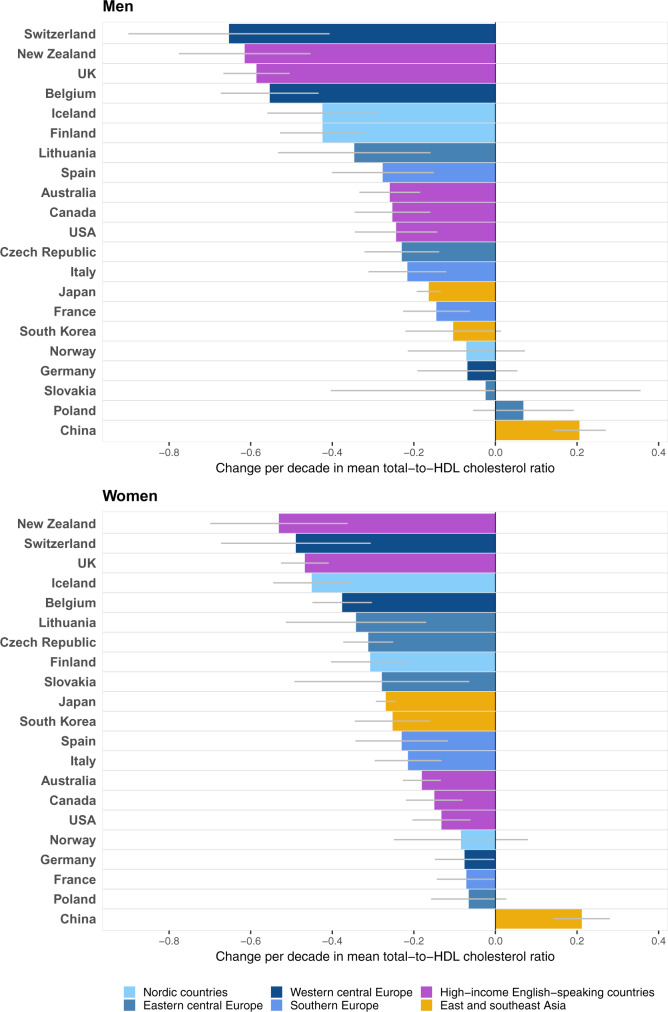
Change per decade in mean total-to-HDL cholesterol ratio by sex in people aged 40–59 years. Results for each country apply to its period of HDL and non-HDL cholesterol data availability (Supplementary Table 2, available as Supplementary data at *IJE* online). See Supplementary Table 4, available as Supplementary data at *IJE* online, for numerical results and 95% confidence intervals.

Mean total-to-HDL cholesterol ratio declined in most Western countries, by as much as ∼0.7 per decade in Swiss men and ∼0.5 per decade in New Zealand and Swiss women (Figure 4). The ratio changed little in Slovakian men. In Asia, China experienced a rise in mean total-to-HDL cholesterol ratio because of the above-mentioned non-favourable changes in lipid fractions. In contrast, despite the rise in mean TC, the total-to-HDL cholesterol ratio declined in Japan and South Korea because HDL cholesterol increased by a larger proportion than did TC.

**Figure 5.**
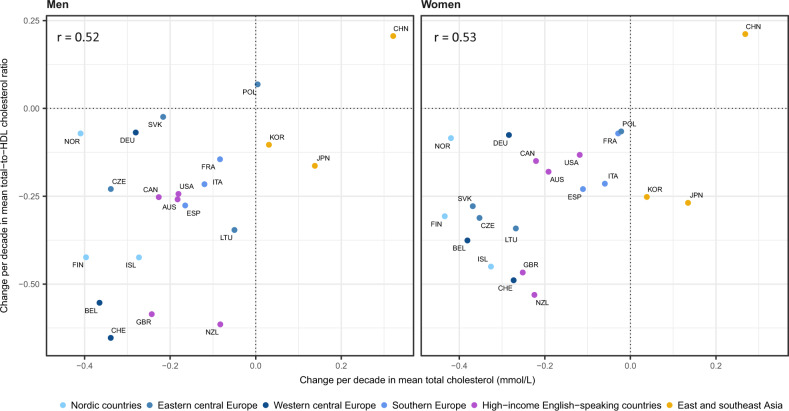
Change per decade in mean total cholesterol vs total-to-HDL cholesterol ratio, in people aged 40–59 years. AUS, Australia; BEL, Belgium; CAN, Canada; CHE, Switzerland; CHN, China; CZE, Czech Republic; DEU, Germany; ESP, Spain; FIN, Finland; FRA, France; GBR, United Kingdom; ISL, Iceland; ITA, Italy; JPN, Japan; KOR, South Korea; LTU, Lithuania; NOR, Norway; NZL, New Zealand; POL, Poland; SVK, Slovakia; USA, United States of America.

The change in mean total-to-HDL cholesterol ratio was only moderately correlated with the change in mean TC (correlation coefficient = 0.52 for men and 0.53 for women) (Figure 5). Japan and South Korea were particularly notable in having had a rise in TC but a decline in the total-to-HDL cholesterol ratio, while Norway, Germany and men in Slovakia had declining TC with little change in the total-to-HDL cholesterol ratio. In 2015, the lowest ratio was that of Japanese women (2.9) and Japanese men (3.7) (Supplementary Figure 6, available as Supplementary data at *IJE* online).

### Results for people aged 60–79 years

**Figure 6.**
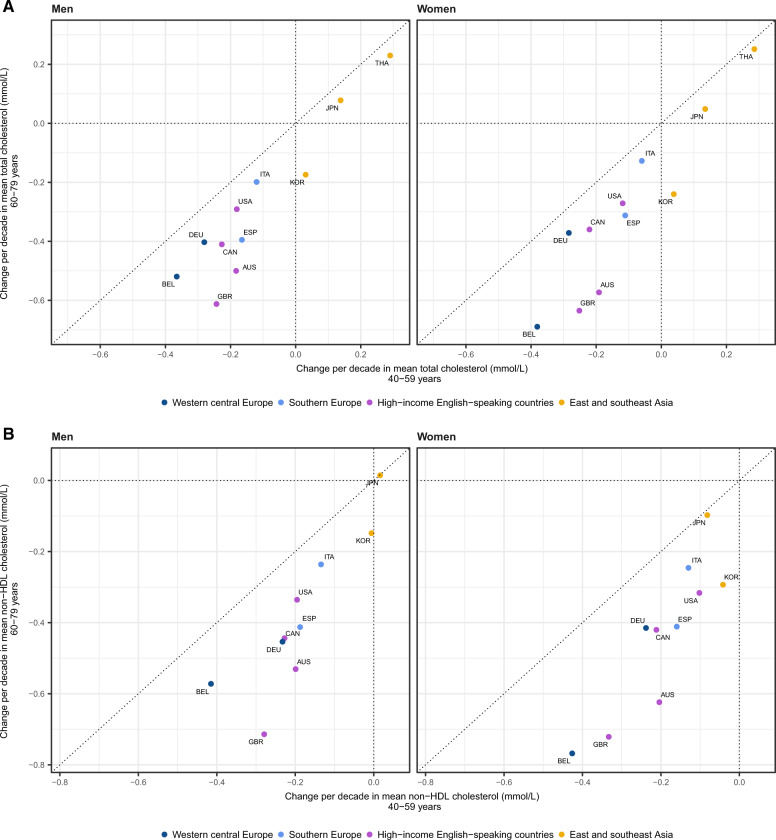

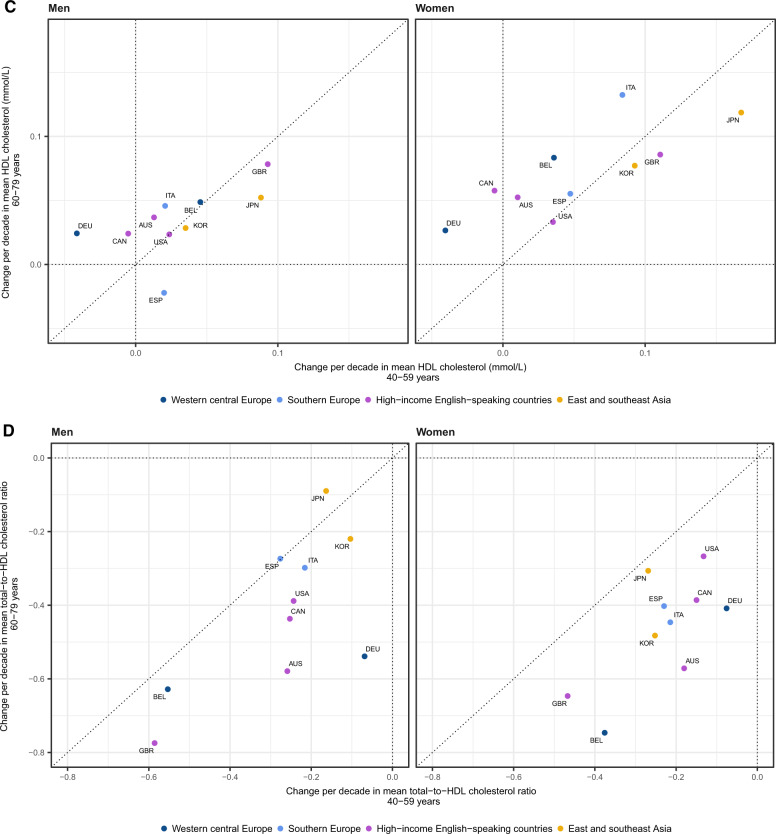
Change per decade in mean (**A**) total cholesterol, (**B**) non-HDL cholesterol, (**C**) HDL cholesterol and (**D**) total-to-HDL cholesterol ratio in people aged 40–59 vs 60–79 years. AUS, Australia; BEL, Belgium; CAN, Canada; DEU, Germany; ESP, Spain; GBR, United Kingdom; ITA, Italy; JPN, Japan; KOR, South Korea; THA, Thailand; USA, United States of America.

Results in people aged 60–79 years were moderately to strongly correlated with those aged 40–59 years (Figure 6 and Supplementary Figure 3, available as Supplementary data at *IJE* online). In virtually all countries, mean TC, non-HDL cholesterol and total-to-HDL cholesterol ratio declined more in these older age groups than in people aged 40–59 years. The decline advantage in older ages was particularly evident for Australia and the UK, where women and men aged 60–79 years experienced a decline in non-HDL cholesterol twice as large as those aged 40–59 years. The change in mean HDL cholesterol was larger in older ages in some countries and smaller in others, indicating that its change may be due to factors that are at least partly different from those affecting non-HDL cholesterol.

## Discussion

By conducting a comparative analysis of changes in TC and lipid fractions and ratios, we found varying rates of decline in TC in Western countries and a rise in Asian countries, leading to an overall convergence in TC among these countries. Underlying this convergence were more heterogeneous trends in HDL and non-HDL cholesterol, with HDL cholesterol rising in more than half the countries included in the analysis. The diverse trends in HDL and non-HDL cholesterol resulted in substantial cross-country variation in trends for mean total-to-HDL cholesterol ratio, with the ratio declining in most countries, but increasing in China.

Our findings on TC trends are largely consistent with prior multi- and single-country reports. Differences from previous studies—e.g. in some countries that participated in the MONICA Project,^6^ Poland^21^ and Switzerland^24^—mostly arise because our study covered a longer period and used a larger number of data sources. Fewer studies have reported trends in lipid fractions and, to our knowledge, none has done so consistently across countries. Studies that have reported trends in lipid fractions for a period longer than 15 years^8^^,^^11^^,^^16^^,^^18–20^^,^^25–27^ have found changes in non-HDL cholesterol (or in LDL cholesterol for some studies) that were consistent with our results.

The observed decline in non-HDL cholesterol in Western countries is likely to have been mostly due to changes in diet—especially the replacement of saturated with unsaturated fats and reduction in trans-fats.^8^^,^^20^^,^^51^ Statins have also been widely used in high-risk patients since the 1990s^26^^,^^52^ and may have helped lower the population mean, especially in older ages. In the majority of countries in our analysis, the decline in non-HDL cholesterol started in the 1980s, before statins were widely used. Further, we observed a decline in non-HDL cholesterol in men and women aged 40–49 years, among whom statin use is relatively low. Nonetheless, the higher use of statins in older ages may at least partly explain the larger decline in non-HDL cholesterol observed in those aged 60–79 years.^26^^,^^53^

Dietary changes in Western countries contrast with the substantial rise in consumption of animal fats in China,^54^ where statin use remains low.^55^ Focusing on non-HDL cholesterol alone, however, conceals important changes in HDL cholesterol and the total-to-HDL cholesterol ratio. Although HDL cholesterol does not have a dominant non-genetic determinant, it is affected adversely (i.e. is lower) by adiposity, type 2 diabetes, intake of trans-fats and carbohydrates, especially those with a high glycaemic index, smoking and the use of some drugs (e.g. β-blockers, anabolic steroids).^56–63^ Conversely, increases in physical activity, alcohol consumption, total fat intake and oestrogens increase HDL cholesterol.^56^^,^^57^^,^^59–63^ A decrease in carbohydrate intake and an increase in fat intake may have contributed to the increase in HDL cholesterol in Japan,^64^^,^^65^ South Korea^62^^,^^66^ and Switzerland,^67^ whereas declines in carbohydrate intake and smoking may have contributed to the rise in the USA^26^ and some other countries. In contrast, an increase in carbohydrate intake^67^ and a decline in alcohol consumption^68^ have been observed in Germany, where we observed a slight decline in HDL cholesterol. The decline in smoking in most Western countries may have also contributed to the observed increase in HDL cholesterol.

The strengths of our study include its novel scope of comparing lipid fractions across countries and using a large number of high-quality population-based studies over more than three decades. Such comprehensive data allowed us to document a significant rise in HDL cholesterol, which is considered difficult to change, in a number of Western and Asian countries as a contributor to the decline in the total-to-HDL cholesterol ratio. A multi-country study, such as ours, is also affected by some limitations. Clinical trials of drugs that raise HDL cholesterol and genetic and epidemiologic studies have shown the complexity of the relationship between HDL cholesterol, HDL particles and cardiovascular and other diseases.^35^^,^^37^^,^^38^^,^^69^ We used HDL and non-HDL cholesterol because there were significantly more data available than on LDL cholesterol and because the total-to-HDL cholesterol ratio is commonly used in clinical practice. We did not analyse trends in different HDL particles because this information is not available in most population-based health surveys and because it is not commonly used to make clinical decisions. For the same reason, we also did not analyse emerging lipid markers such as apolipoprotein B and apolipoprotein A-I.^56^^,^^70^ We used the average change per decade, estimated in a linear model, which has the advantage of being parsimonious, but trends in some countries may be non-linear. When we fitted a non-linear LOESS regression (Supplementary Figure 3, available as Supplementary data at *IJE* online), the estimated average decadal change was similar to the estimates from the linear model in most countries. Almost 80% of the studies in our analysis had used enzymatic methods for measuring TC, which have been well standardized since at least the 1980s. Although methods to measure HDL cholesterol have evolved over time—chemical precipitation methods to separate HDL and, more recently, homogeneous assays^71^—more than three-quarters of the studies in our analysis participated in a lipid standardization programme (Supplementary Figure 2, available as Supplementary data at *IJE* online). A rise in HDL cholesterol was also seen in countries and over periods where measurement methods did not change. Nonetheless, the observed changes in HDL cholesterol in some countries were in the same order of magnitude to which laboratories’ accuracies can be standardized. Although most studies had measured cholesterol in serum, ∼11% had used plasma. Adjusting for plasma-serum differences had little impact on our results and did not change our conclusions (Supplementary Figure 7, available as Supplementary data at *IJE* online) because cholesterol measured in plasma and serum differ by only about 3%.^50^ Finally, although all our data were from samples of the general population, 40% came from community-based studies. In some countries, community-based studies came from the same community in different years; in others, studies were from different parts of the same country, which led to additional variability in data and uncertainty in the estimated change. Our key findings on lipid fractions were also seen where the data sources covered the entire country or large parts of it. In 11 countries, our analysis was limited to ages 40–59 years because fewer studies had data in people older than 60 years of age, for whom non-HDL cholesterol may have declined more due to the use of statins, as indicated by the results in the 10 countries with data covering ages 40–79 years.

Whereas early epidemiological studies used TC as a marker of cardiovascular risk in individuals and populations,^72^ our study shows that the populations of Asian and Western countries have experienced large and heterogeneous changes in lipid fractions, including substantial increases in HDL cholesterol and substantial falls in non-HDL cholesterol. In the best-performing countries, those in Europe and New Zealand, the total-to-HDL cholesterol ratio has declined by 1.5–2.3 since the 1980s, which is equivalent to a 48–63% reduction in the risk of CHD.^1^ In Japan and South Korea, the total-to-HDL cholesterol ratio has declined, which provides a simple explanation for the apparent paradox of declining CHD while TC increased.^73^ A key implication of our findings is the need for national surveillance systems that, consistently with modern clinical practice, measure relevant lipid fractions and their determinants, including diet, health behaviours such as smoking and alcohol use, and use of statins to support the design and evaluation of public-health programmes.

Despite the improvements that we have documented, the populations of all countries analysed here would benefit from lower non-HDL cholesterol and total-to-HDL cholesterol ratios. In China, which had some of the lowest recorded non-HDL cholesterol and TC levels a few decades ago, changes in diet and relatively low treatment coverage have led to unfavourable trends in lipid profiles. Therefore, population-based policies and targeted interventions to improve nutrition and enhance treatment are still needed in all these countries and should be designed and evaluated based on their impacts on all health-relevant lipid fractions and on the corresponding health outcomes.

## Funding

This work was supported by the Wellcome Trust (grant numbers 101506/Z/13/Z and Research Training Fellowship 203616/Z/16/Z). R.C. acknowledges funding from the Ministry of Health of the Czech Republic (grant number 15-27109A).

## Supplementary Material

dyz099_Supplementary_Material

## NCD Risk Factor Collaboration (NCD-RisC)

### Pooled Analysis and Writing

Cristina Taddei (Imperial College London, UK); Rod Jackson (University of Auckland, New Zealand); Bin Zhou (Imperial College London, UK); Honor Bixby (Imperial College London, UK); Goodarz Danaei (Harvard T.H. Chan School of Public Health, USA); Mariachiara Di Cesare (Middlesex University, UK); Kari Kuulasmaa (National Institute for Health and Welfare, Finland); Kaveh Hajifathalian (New York-Presbyterian/Weill Cornell Medicine, USA); James Bentham (University of Kent, UK); James E Bennett (Imperial College London, UK); Wichai Aekplakorn (Mahidol University, Thailand); Renata Cifkova (Charles University in Prague, Czech Republic; Thomayer Hospital, Czech Republic); Jean Dallongeville (Institut Pasteur de Lille, France); Dirk De Bacquer (Ghent University, Belgium); Simona Giampaoli (Istituto Superiore di Sanità, Italy); Vilmundur Gudnason (Icelandic Heart Association, Iceland); Young-Ho Khang (Seoul National University, South Korea); Tiina Laatikainen (National Institute for Health and Welfare, Finland); Jim I Mann (University of Otago, New Zealand); Pedro Marques-Vidal (Lausanne University Hospital, Switzerland); George A Mensah (National Institutes of Health, USA); Martina Müller-Nurasyid (Helmholtz Zentrum München, Germany); Toshiharu Ninomiya (Kyushu University, Japan); Janina Petkeviciene (Lithuanian University of Health Sciences, Lithuania); Fernando Rodríguez-Artalejo (Universidad Autónoma de Madrid/CIBERESP, Spain); Jennifer Servais (Statistics Canada, Canada); Stefan Söderberg (Umeå University, Sweden); Bill Stavreski (Heart Foundation, Australia); Tom Wilsgaard (UiT The Arctic University of Norway, Norway); Tomasz Zdrojewski (Medical University of Gdansk, Poland); Dong Zhao (Capital Medical University Beijing An Zhen Hospital, China); Gretchen A Stevens (World Health Organization, Switzerland); Stefan Savin (World Health Organization, Switzerland); Melanie J Cowan (World Health Organization, Switzerland); Leanne M Riley (World Health Organization, Switzerland); Majid Ezzati (Imperial College London, UK)

### Country and Regional Data (* equal contribution; listed alphabetically)

Robert J Adams (The University of Adelaide, Australia)*; Wichai Aekplakorn (Mahidol University, Thailand)*; Wolfgang Ahrens (Leibniz Institute for Prevention Research and Epidemiology - BIPS, Germany)*; Philippe Amouyel (Lille University and Hospital, France)*; Antoinette Amuzu (London School of Hygiene & Tropical Medicine, UK)*; Sigmund A Anderssen (Norwegian School of Sport Sciences, Norway)*; Inger Ariansen (Norwegian Institute of Public Health, Norway)*; Dominique Arveiler (Strasbourg University and Hospital, France)*; Thor Aspelund (University of Iceland, Iceland)*; Juha Auvinen (University of Oulu, Finland)*; Mária Avdicová (Regional Authority of Public Health, Banska Bystrica, Slovakia)*; Maciej Banach (Medical University of Lodz, Poland)*; Piotr Bandosz (Medical University of Gdansk, Poland)*; José R Banegas (Universidad Autónoma de Madrid, Spain)*; Carlo M Barbagallo (University of Palermo, Italy)*; Iqbal Bata (Dalhousie University, Canada)*; Louise A Baur (University of Sydney, Australia)*; Robert Beaglehole (University of Auckland, New Zealand)*; James E Bennett (Imperial College London, UK)*; Gailute Bernotiene (Lithuanian University of Health Sciences, Lithuania)*; Yufang Bi (Shanghai Jiao-Tong University School of Medicine, China)*; Asako Bienek (Public Health Agency of Canada, Canada)*; Cecilia Björkelund (University of Gothenburg, Sweden)*; Simona Bo (University of Turin, Italy)*; Bernhard O Boehm (Nanyang Technological University, Singapore)*; Marialaura Bonaccio (IRCCS Neuromed, Italy)*; Vanina Bongard (Toulouse University School of Medicine, France)*; Rossana Borchini (University Hospital of Varese, Italy)*; Herman Borghs (University Hospital KU Leuven, Belgium)*; Juergen Breckenkamp (Bielefeld University, Germany)*; Hermann Brenner (German Cancer Research Center, Germany)*; Graziella Bruno (University of Turin, Italy)*; Markus A Busch (Robert Koch Institute, Germany)*; Antonio Cabrera de León (Universidad de La Laguna, Spain)*; Vincenzo Capuano (Cardiologia di Mercato S. Severino, Italy)*; Felipe F Casanueva (Santiago de Compostela University, Spain)*; Juan-Pablo Casas (University College London, UK)*; Carmelo A Caserta (Associazione Calabrese di Epatologia, Italy)*; Laura Censi (Council for Agricultural Research and Economics, Italy)*; Fangfang Chen (Capital Institute of Pediatrics, China)*; Shuohua Chen (Kailuan General Hospital, China)*; María-Dolores Chirlaque (Murcia Health Council, CIBERESP, IMIB-Arrixaca, Spain)*; Belong Cho (Seoul National University College of Medicine, South Korea)*; Yumi Cho (Korea Centers for Disease Control and Prevention, South Korea)*; Jerzy Chudek (Medical University of Silesia, Poland)*; Renata Cifkova (Charles University in Prague, Czech Republic; Thomayer Hospital, Czech Republic)*; Frank Claessens (Katholieke Universiteit Leuven, Belgium)*; Janine Clarke (Statistics Canada, Canada)*; Els Clays (Ghent University, Belgium)*; Cyrus Cooper (University of Southampton, UK)*; Simona Costanzo (IRCCS Neuromed, Italy)*; Dominique Cottel (Institut Pasteur de Lille, France)*; Chris Cowell (University of Sydney, Australia)*; Ana B Crujeiras (CIBEROBN, Spain)*; Liufu Cui (Kailuan General Hospital, China)*; Graziella D'Arrigo (National Council of Research, Italy)*; Jean Dallongeville (Institut Pasteur de Lille, France)*; Luc Dauchet (Lille University Hospital, France)*; Guy De Backer (Ghent University, Belgium)*; Dirk De Bacquer (Ghent University, Belgium)*; Giovanni de Gaetano (IRCCS Neuromed, Italy)*; Stefaan De Henauw (Ghent University, Belgium)*; Delphine De Smedt (Ghent University, Belgium)*; Elaine Dennison (University of Southampton, UK)*; Valérie Deschamps (French Public Health Agency, France)*; Augusto Di Castelnuovo (Mediterranea Cardiocentro, Italy)*; Annette J Dobson (University of Queensland, Australia)*; Chiara Donfrancesco (Istituto Superiore di Sanità, Italy)*; Angela Döring (Helmholtz Zentrum München, Germany)*; Wojciech Drygas (The Cardinal Wyszynski Institute of Cardiology, Poland)*; Yong Du (Robert Koch Institute, Germany)*; Elzbieta Dziankowska-Zaborszczyk (Medical University of Lodz, Poland)*; Robert Eggertsen (University of Gothenburg, Sweden)*; Ulf Ekelund (Norwegian School of Sport Sciences, Norway)*; Roberto Elosua (Institut Hospital del Mar d'Investigacions Mèdiques, Spain)*; Johan G Eriksson (National Institute for Health and Welfare, Finland)*; Alun Evans (Queen's University of Belfast, UK)*; David Faeh (University of Zurich, Switzerland)*; Francisco J Felix-Redondo (Centro de Salud Villanueva Norte, Spain)*; Daniel Fernández-Bergés (Servicio Extremeño de Salud, Spain)*; Marika Ferrari (Council for Agricultural Research and Economics, Italy)*; Jean Ferrieres (Toulouse University School of Medicine, France)*; Joseph D Finn (University of Manchester, UK)*; Ann-Sofie Forslund (Umeå University, Sweden)*; Maria Forsner (Umeå University, Sweden)*; Guillermo Frontera (Hospital Universitario Son Espases, Spain)*; Yuki Fujita (Kindai University, Japan)*; Zbigniew Gaciong (Medical University of Warsaw, Poland)*; Fabio Galvano (University of Catania, Italy)*; Jingli Gao (Kailuan General Hospital, China)*; Manoli Garcia-de-la-Hera (CIBER en Epidemiología y Salud Pública, Spain)*; Sarah P Garnett (University of Sydney, Australia)*; Jean-Michel Gaspoz (Geneva University Hospitals, Switzerland)*; Magda Gasull (CIBER en Epidemiología y Salud Pública, Spain)*; Louise Gates (Australian Bureau of Statistics, Australia)*; Simona Giampaoli (Istituto Superiore di Sanità, Italy)*; Francesco Gianfagna (University of Insubria, Italy; Mediterranea Cardiocentro, Italy)*; Tiffany K Gill (The University of Adelaide, Australia)*; Jonathan Giovannelli (Lille University Hospital, France)*; Aleksander Giwercman (Lund University, Sweden)*; David Goltzman (McGill University, Canada)*; Marcela Gonzalez Gross (Universidad Politécnica de Madrid, Spain)*; Frederic Gottrand (Université de Lille 2, France)*; Sidsel Graff-Iversen (Norwegian Institute of Public Health, Norway)*; Dušan Grafnetter (Institute for clinical and experimental medicine, Czech Republic)*; Ronald D Gregor (Dalhousie University, Canada)*; Tomasz Grodzicki (Jagiellonian University Medical College, Poland)*; Giuseppe Grosso (University of Catania, Italy)*; Gabriella Gruden (University of Turin, Italy)*; Dongfeng Gu (National Center of Cardiovascular Diseases, China)*; Pilar Guallar-Castillón (Universidad Autónoma de Madrid, Spain)*; Elias F Gudmundsson (Icelandic Heart Association, Iceland)*; Vilmundur Gudnason (University of Iceland, Iceland)*; Idris Guessous (Geneva University Hospitals, Switzerland)*; Johanna Gunnlaugsdottir (Icelandic Heart Association, Iceland)*; Felix Gutzwiller (University of Zurich, Switzerland)*; Rebecca Hardy (University College London, UK)*; Jun Hata (Kyushu University, Japan)*; Teresa Haugsgjerd (University of Bergen, Norway)*; Alison J Hayes (University of Sydney, Australia)*; Jiang He (Tulane University, USA)*; Yuna He (Chinese Center  for Disease Control and Prevention, China)*; Sauli Herrala (Oulu University Hospital, Finland)*; Ilpo Tapani Hihtaniemi (Imperial College London, UK)*; Michael Hobbs (University of Western Australia, Australia)*; Wilma M Hopman (Kingston Health Sciences Centre, Canada)*; José María Huerta (CIBER en Epidemiología y Salud Pública, Spain)*; Inge Huybrechts (International Agency for Research on Cancer, France)*; Licia Iacoviello (University of Insubria, Italy; IRCCS Neuromed, Italy)*; Anna G Iannone (Cardiologia di Mercato S. Severino, Italy)*; Nayu Ikeda (National Institute of Health and Nutrition, Japan)*; Masanori Iwasaki (Niigata University, Japan)*; Rod Jackson (University of Auckland, New Zealand)*; Konrad Jamrozik (The University of Adelaide, Australia; deceased)*; Imre Janszky (Norwegian University of Science and Technology, Norway)*; Marjo-Riitta Jarvelin (Imperial College London, UK; University of Oulu, Finland)*; Grazyna Jasienska (Jagiellonian University Medical College, Poland)*; Garry Jennings (Heart Foundation, Australia)*; Seung-lyeal Jeong (National Health Insurance Service, South Korea)*; Chao Qiang Jiang (Guangzhou 12^th^ Hospital, China)*; Michel Joffres (Simon Fraser University, Canada)*; Jari J Jokelainen (Oulu University Hospital, Finland)*; Jost B Jonas (Ruprecht-Karls-University of Heidelberg, Germany)*; Jacek Jóźwiak (University of Opole, Poland)*; Eero O Kajantie (National Institute for Health and Welfare, Finland)*; Jussi Kauhanen (University of Eastern Finland, Finland)*; Ulrich Keil (University of Münster, Germany)*; Sirkka Keinänen-Kiukaanniemi (Oulu University Hospital, Finland)*; Mathilde Kersting (Research Institute of Child Nutrition, Germany)*; Young-Ho Khang (Seoul National University, South Korea)*; Ursula Kiechl-Kohlendorfer (Medical University of Innsbruck, Austria)*; Stefan Kiechl (Medical University of Innsbruck, Austria)*; Jeongseon Kim (National Cancer Center, South Korea)*; Yeon-Yong Kim (National Health Insurance Service, South Korea)*; Jurate Klumbiene (Lithuanian University of Health Sciences, Lithuania)*; Michael Knoflach (Medical University of Innsbruck, Austria)*; Stephanie Ko (Public Health Agency of Canada, Canada)*; Elin Kolle (Norwegian School of Sport Sciences, Norway)*; Raija Korpelainen (University of Oulu, Finland; Oulu Deaconess Institute Foundation, Finland)*; Seppo Koskinen (National Institute for Health and Welfare, Finland)*; Katsuyasu Kouda (Kindai University, Japan)*; Wolfgang Kratzer (University Hospital Ulm, Germany)*; Susi Kriemler (University of Zürich, Switzerland)*; Steinar Krokstad (Norwegian university of science and technology, Norway)*; Urho M Kujala (University of Jyväskylä, Finland)*; Pawel Kurjata (The Cardinal Wyszynski Institute of Cardiology, Poland)*; Kari Kuulasmaa (National Institute for Health and Welfare, Finland)*; Tiina Laatikainen (National Institute for Health and Welfare, Finland)*; Tai Hing Lam (University of Hong Kong, China)*; Vera Lanska (Institute for Clinical and Experimental Medicine, Czech Republic)*; Georg Lappas (Sahlgrenska Academy, Sweden)*; Lars E Laugsand (Norwegian University of Science and Technology, Norway)*; Jeonghee Lee (National Cancer Center, South Korea)*; Terho Lehtimäki (Tampere University Hospital, Finland; Tampere University, Finland)*; Yanping Li (Harvard TH Chan School of Public Health, USA)*; Christa L Lilly (West Virginia University, USA)*; Xu Lin (University of Chinese Academy of Sciences, China)*; Lars Lind (Uppsala University, Sweden)*; Lauren Lissner (Gothenburg University, Sweden)*; Jing Liu (Capital Medical University Beijing An Zhen Hospital, China)*; Esther Lopez-Garcia (Universidad Autónoma de Madrid, Spain)*; Roberto Lorbeer (University Medicine Greifswald, Germany)*; José Eugenio Lozano (Consejería de Sanidad Junta de Castilla y León, Spain)*; Dalia Luksiene (Lithuanian University of Health Sciences, Lithuania)*; Annamari Lundqvist (National Institute for Health and Welfare, Finland)*; Robert Lundqvist (Norrbotten County Council, Sweden)*; Per Lytsy (University of Uppsala, Sweden)*; Guansheng Ma (Peking University, China)*; Suka Machi (The Jikei University School of Medicine, Japan)*; Stefania Maggi (National Research Council, Italy)*; Dianna J Magliano (Baker Heart and Diabetes Institute, Australia)*; Jim I Mann (University of Otago, New Zealand)*; Enzo Manzato (University of Padova, Italy)*; Pedro Marques-Vidal (Lausanne University Hospital, Switzerland)*; Ellisiv B Mathiesen (UiT The Arctic University of Norway, Norway)*; Stela McLachlan (University of Edinburgh, UK)*; Rachael M McLean (University of Otago, New Zealand)*; Scott B McLean (Statistics Canada, Canada)*; Aline Meirhaeghe (Institut National de la Santé et de la Recherche Médicale, France)*; Christa Meisinger (Helmholtz Zentrum München, Germany)*; Patricia Metcalf (University of Auckland, New Zealand)*; Jie Mi (Capital Institute of Pediatrics, China)*; Jody C Miller (University of Otago, New Zealand)*; Luis A Moreno (Universidad de Zaragoza, Spain)*; Suzanne N Morin (McGill University, Canada)*; Malgorzata Mossakowska (International Institute of Molecular and Cell Biology, Poland)*; Maria L Muiesan (University of Brescia, Italy)*; Martina Müller-Nurasyid (Helmholtz Zentrum München, Germany)*; Jaakko Mursu (University of Eastern Finland, Finland)*; Harunobu Nakamura (Kobe University, Japan)*; Jana Námešná (Regional Authority of Public Health, Banska Bystrica, Slovakia)*; Matthias Nauck (University Medicine of Greifswald, Germany)*; Eva Maria Navarrete-Muñoz (CIBER en Epidemiología y Salud Pública, Spain)*; William A Neal (West Virginia University, USA)*; Ilona Nenko (Jagiellonian University Medical College, Poland)*; Teemu J Niiranen (National Institute for Health and Welfare, Finland; University of Turku, Finland)*; Guang Ning (Shanghai Jiao-Tong University School of Medicine, China)*; Toshiharu Ninomiya (Kyushu University, Japan)*; Marianna Noale (National Research Council, Italy)*; Davide Noto (University of Palermo, Italy)*; Terence O'Neill (University of Manchester, UK)*; Dermot O'Reilly (Queen's University of Belfast, UK)*; Kyungwon Oh (Korea Centers for Disease Control and Prevention, South Korea)*; Örn Olafsson (Icelandic Heart Association, Iceland)*; Fred Michel Paccaud (Institute for social and preventive medicine, Canada)*; Andrzej Pajak (Jagiellonian University Medical College, Poland)*; Luigi Palmieri (Istituto Superiore di Sanità, Italy)*; Francesco Panza (IRCCS Casa Sollievo della Sofferenza, Italy)*; Winsome R Parnell (University of Otago, New Zealand)*; Markku Peltonen (National Institute for Health and Welfare, Finland)*; Annette Peters (Helmholtz Zentrum München, Germany)*; Astrid Petersmann (University Medicine of Greifswald, Germany)*; Janina Petkeviciene (Lithuanian University of Health Sciences, Lithuania)*; Iris Pigeot (Leibniz Institute for Prevention Research and Epidemiology - BIPS, Germany)*; Lorenza Pilotto (Cardiovascular Prevention Centre Udine, Italy)*; Aleksandra Piwonska (The Cardinal Wyszynski Institute of Cardiology, Poland)*; Pedro Plans-Rubió (Public Health Agency of Catalonia, Spain)*; Miquel Porta (Institut Hospital del Mar d'Investigacions Mèdiques, Spain)*; Jacqueline F Price (University of Edinburgh, UK)*; Jardena J Puder (Lausanne University Hospital, Switzerland)*; Soile E Puhakka (University of Oulu; Oulu Deaconess Institute Foundation, Finland)*; Ricardas Radisauskas (Lithuanian University of Health Sciences, Lithuania)*; Olli Raitakari (University of Turku, Finland)*; Rafel Ramos (Institut Universitari d’Investigació en Atenció Primària Jordi Gol, Spain)*; Josep Redon (University of Valencia, Spain)*; Fernando Rigo (Health Center San Agustín, Spain)*; Fernando Rodríguez-Artalejo (Universidad Autónoma de Madrid/CIBERESP, Spain)*; María del Cristo Rodriguez-Perez (Canarian Health Service, Spain)*; Dora Romaguera (CIBEROBN, Spain)*; Kimmo Ronkainen (University of Eastern Finland, Finland)*; Annika Rosengren (University of Gothenburg, Sweden)*; Joel GR Roy (Statistics Canada, Canada)*; Jean-Bernard Ruidavets (Toulouse University Hospital, France)*; Marcin Rutkowski (Medical University of Gdansk, Poland)*; Benoit Salanave (French Public Health Agency, France)*; Diego Salmerón (CIBER de Epidemiología y Salud Pública, Spain)*; Veikko Salomaa (National Institute for Health and Welfare, Finland)*; Jukka T Salonen (University of Helsinki, Finland)*; Massimo Salvetti (University of Brescia, Italy)*; Susana Sans (Catalan Department of Health, Spain)*; Jouko L Saramies (South Karelia Social and Health Care District, Finland)*; Kai-Uwe Saum (German Cancer Research Center, Germany)*; Norie Sawada (National Cancer Center, Japan)*; Christa Scheidt-Nave (Robert Koch Institute, Germany)*; Anja Schienkiewitz (Robert Koch Institute, Germany)*; Sabine Schipf (University Medicine of Greifswald, Germany)*; Carsten O Schmidt (University Medicine of Greifswald, Germany)*; Ben Schöttker (German Cancer Research Center, Germany)*; Sylvain Sebert (University of Oulu, Finland)*; Abhijit Sen (Norwegian University of Science and Technology, Norway)*; Jennifer Servais (Statistics Canada, Canada)*; Jonathan E Shaw (Baker Heart and Diabetes Institute, Australia)*; Kenji Shibuya (King's College London, UK)*; Dong Wook Shin (Sungkyunkwan University, South Korea)*; Rahman Shiri (Finnish Institute of Occupational Health, Finland)*; Judith Simons (St Vincent's Hospital, Australia)*; Leon A Simons (University of New South Wales, Australia)*; Michael Sjöström (Karolinska Institutet, Sweden)*; Jolanta Slowikowska-Hilczer (Medical University of Lodz, Poland)*; Przemyslaw Slusarczyk (International Institute of Molecular and Cell Biology, Poland)*; Stefan Söderberg (Umeå University, Sweden)*; Vincenzo Solfrizzi (University of Bari, Italy)*; Emily Sonestedt (Lund University, Sweden)*; Aicha Soumare (University of Bordeaux, France)*; Jan A Staessen (University of Leuven, Belgium)*; Maria G Stathopoulou (INSERM, France)*; Bill Stavreski (Heart Foundation, Australia)*; Jostein Steene-Johannessen (Norwegian School of Sport Sciences, Norway)*; Peter Stehle (Bonn University, Germany)*; Jutta Stieber (Helmholtz Zentrum München, Germany; deceased)*; Doris Stöckl (Helmholtz Zentrum München, Germany)*; Jakub Stokwiszewski (National Institute of Public Health-National Institute of Hygiene, Poland)*; Johan Sundström (Uppsala University, Sweden)*; Paibul Suriyawongpaisal (Mahidol University, Thailand)*; Abdonas Tamosiunas (Lithuanian University of Health Sciences, Lithuania)*; Eng Joo Tan (University of Sydney, Australia)*; Anne Taylor (The University of Adelaide, Australia)*; Grethe Tell (University of Bergen, Norway)*; Lutgarde Thijs (University of Leuven, Belgium)*; Hanna K Tolonen (National Institute for Health and Welfare, Finland)*; Roman Topór-Madry (Jagiellonian University Medical College, Poland)*; María José Tormo (Health Service of Murcia, Spain)*; Maties Torrent (IB-SALUT Area de Salut de Menorca, Spain)*; Shoichiro Tsugane (National Cancer Center, Japan)*; Tomi-Pekka Tuomainen (University of Eastern Finland, Finland)*; Jaakko Tuomilehto (National Institute for Health and Welfare, Finland)*; Christophe Tzourio (University of Bordeaux, France)*; Hannu MT Uusitalo (University of Tampere Tays Eye Center, Finland)*; Koen Van Herck (Ghent University, Belgium)*; Dirk Vanderschueren (Katholieke Universiteit Leuven, Belgium)*; Diego Vanuzzo (Cardiovascular Prevention Centre Udine, Italy)*; Lars Vatten (Norwegian University of Science and Technology, Norway)*; Tomas Vega (Consejería de Sanidad Junta de Castilla y León, Spain)*; Giovanni Veronesi (University of Insubria, Italy)*; Jesus Vioque (Universidad Miguel Hernandez, Spain)*; Jyrki K Virtanen (University of Eastern Finland, Finland)*; Sophie Visvikis-Siest (INSERM, France)*; Peter Vollenweider (Lausanne University Hospital, Switzerland)*; Sari Voutilainen (University of Eastern Finland, Finland)*; Martine Vrijheid (ISGlobal Centre for Research in Environmental Epidemiology, Spain)*; Aline Wagner (University of Strasbourg, France)*; Anne Wagner (National Institute for Health and Welfare, Finland)*; Ming-Dong Wang (Health Canada, Canada)*; Qian Wang (Xinjiang Medical University, China)*; Ya Xing Wang (Capital Medical University, China)*; S Goya Wannamethee (University College London, UK)*; Wenbin Wei (Capital Medical University, China)*; Peter H Whincup (St George’s, University of London, UK)*; Andrzej Wiecek (Medical University of Silesia, Poland)*; Johann Willeit (Medical University of Innsbruck, Austria)*; Peter Willeit (Medical University of Innsbruck, Austria)*; Tom Wilsgaard (UiT The Arctic University of Norway, Norway)*; Bogdan Wojtyniak (National Institute of Public Health-National Institute of Hygiene, Poland)*; Andrew Wong (University College London, UK)*; Mark Woodward (University of New South Wales, Australia; University of Oxford, UK)*; Frederick C Wu (University of Manchester, UK)*; Shouling Wu (Kailuan General Hospital, China)*; Haiquan Xu (Institute of Food and Nutrition Development of Ministry of Agriculture, China)*; Liang Xu (Beijing Institute of Ophthalmology, China)*; Weili Yan (Children's Hospital of Fudan University, China)*; Xiaoguang Yang (Chinese Center for Disease Control and Prevention, China)*; Xingwang Ye (University of Chinese Academy of Sciences, China)*; Akihiro Yoshihara (Niigata University, Japan)*; Sabina Zambon (University of Padova, Italy)*; Tomasz Zdrojewski (Medical University of Gdansk, Poland)*; Dong Zhao (Capital Medical University Beijing An Zhen Hospital, China)*; Wenhua Zhao (Chinese Center for Disease Control and Prevention, China)*
