# Supplementary material for: National trends in total cholesterol obscure heterogeneous changes in HDL and non-HDL cholesterol and total-to-HDL cholesterol ratio: a pooled analysis of 458 population-based studies in Asian and Western countries
Source: Int J Epidemiol. 2019 Jul 18;49(1):173–92. doi: 10.1093/ije/dyz099 (PMC7245049; doi:10.1093/ije/dyz099)
Supplement: dyz099_Supplementary_Material [file dyz099_supplementary_material.pdf]

Supplementary Data

National trends in total cholesterol obscure heterogeneous changes in HDL and non-HDL cholesterol and total-to-HDL cholesterol ratio: a pooled analysis of 458 population-based studies in Asian and Western countries

NCD Risk Factor Collaboration (NCD-RisC)

Table of Contents

Supplementary Table 1: Data sources used in the analysis. 3

Supplementary Table 2: Data availability by country for ages 40-59 years. 29

Supplementary Table 3: Change per decade in mean total cholesterol by sex in people aged 40-59 years, shown graphically in Figure 1 in the main paper. 30

Supplementary Table 4: Change per decade in mean HDL and non-HDL cholesterol, and in mean total-to-HDL cholesterol ratio by sex in people aged 40-59 years, shown graphically in Figure 2 and Figure 4 in the main paper. 31

Supplementary Figure 1: The association between mean LDL and non-HDL cholesterol in studies with data on both variables. 33

Supplementary Figure 2: Measurement methods and participation in a lipid standardisation programme in studies with data on HDL cholesterol. 35

Supplementary Figure 3: Linear and nonlinear (LOESS) regression fits by country, sex and age group. 57

Supplementary Figure 4: Mean total cholesterol at the beginning and end of analysis period by country and sex in people aged 40 to 59 years. 81

Supplementary Figure 5: Mean (A) non-HDL and (B) HDL cholesterol at the beginning and end of analysis period by country and sex in people aged 40 to 59 years. 83

Supplementary Figure 6: Mean total-to-HDL cholesterol ratio at the beginning and end of analysis period by country and sex in people aged 40 to 59 years. 86

Supplementary Figure 7: Change per decade in mean (A) total cholesterol, (B) non-HDL cholesterol and (C) HDL cholesterol in people aged 40-59 years unadjusted versus adjusted for plasma-serum differences. 88

Supplementary Table 1: Data sources used in the analysis.

	Country	Data years	Survey/Study name/Citation	Level of representati-veness	Age range as used for the analysis		Sample size (Total cholesterol)		Sample size (HDL cholesterol)		Sample size (Non-HDL cholesterol)		Method used to measure		Whether lipids were measured in serum or plasma samples	Participating to a lipid standardisation programme or quality control schemes	Note
					Male	Female	Male	Female	Male	Female	Male	Female	Total cholesterol	HDL cholesterol (separation and quantification methods)			
1	Australia	1980	Risk Factor Prevalence Study	National	40-59	40-59	1,372	1,418	1,279	1,330	1,278	1,330	LRC method	Heparin-Manganese method; LRC method	Plasma	Yes (CDC/CRMLN)	
2	Australia	1983	MONICA, Newcastle	Subnational	40-59	40-59	775	788	736	772	735	765	Extraction/Enzymatic	Heparin-Manganese method; Extraction	Plasma	Yes (WHO-LRC)	
3	Australia	1983	Risk Factor Prevalence Study	National	40-59	40-59	1,754	1,796	1,739	1,807	1,722	1,785	LRC method	Heparin-Manganese method; LRC method	Plasma	Yes (CDC/CRMLN)	
4	Australia	1988-1989	Dubbo Study of Australian Elderly	Community	60-79	60-79	807	1,084	805	1,084	805	1,084	Enzymatic	Phosphotungstate-Magnesium (PTA) method; Enzymatic	Serum	Yes (CDC/CRMLN)	
5	Australia	1988-1989	MONICA, Newcastle	Subnational	40-59	40-59	416	411	415	411	415	411	Enzymatic	PEG 6000 method; Enzymatic	Serum	Yes (WHO-LRC)	
6	Australia	1989	Risk Factor Prevalence Study	National	40-69	40-69	2,555	2,577	2,555	2,577	2,555	2,577	Enzymatic	PEG 6000 method; Enzymatic	Plasma	Yes (WHO-LRC)	
7	Australia	1992-1993	Australia Longitudinal Study of Ageing	Community	70-79	70-79	341	316	340	316	340	316	Unknown	Unknown	Serum	Unknown	
8	Australia	1994	MONICA, Newcastle	Subnational	40-59	40-59	383	422	381	421	381	421	Enzymatic	PEG 6000 method; Enzymatic	Serum	Yes (WHO-LRC)	
9	Australia	1994	MONICA, Perth inner	Community	40-59	40-59	192	185	192	185	192	185	Enzymatic	Heparin-Manganese method; Enzymatic	Plasma	Yes (WHO-LRC)	
10	Australia	1994	MONICA, Perth outer	Community	40-59	40-59	206	212	206	212	206	212	Enzymatic	Heparin-Manganese method; Enzymatic	Plasma	Yes (WHO-LRC)	
11	Australia	1999-2000	The Australian Diabetes, Obesity and Lifestyle Study 1999-2000	National	40-79	40-79	3,822	4,559	3,820	4,558	3,820	4,558	Enzymatic	Unknown; Enzymatic	Serum	Yes (other)	
12	Australia	1999-2003	North West Adelaide Health Study	Community	40-79	40-79	1,312	1,462	1,312	1,462	1,311	1,462	Enzymatic	Homogeneous assay (Antibody, two reagents)	Serum	Yes (other)	
13	Australia	2004-2005	The Australian Diabetes, Obesity and Lifestyle Study 2004-2005	National	40-79	40-79	2,480	2,986	2,480	2,984	2,480	2,984	Enzymatic	Homogeneous assay (Dextran sulphate-Magnesium + PEG-coupled enzymes)	Serum	Yes (other)	
14	Australia	2004-2006	North West Adelaide Health Study	Community	40-79	40-79	1,141	1,304	1,141	1,304	1,141	1,304	Enzymatic	Homogeneous assay (Antibody, two reagents)	Serum	Yes (other)	
15	Australia	2008-2010	North West Adelaide Health Study	Community	40-79	40-79	935	1,070	935	1,070	935	1,070	Enzymatic	Homogeneous assay (Antibody, two reagents)	Serum	Yes (other)	
16	Australia	2011-2012	Australian Health Survey	National	40-79	40-79	3,023	3,590	3,023	3,590	3,023	3,590	Enzymatic	Homogeneous assay (Accelerator Selective Detergent; Enzymatic)	Serum	Unknown	
17	Australia	2012	The Australian Diabetes, Obesity and Lifestyle Study 2012	National	40-79	40-79	1,863	2,342	1,863	2,342	1,863	2,342	Enzymatic	Unknown; Enzymatic	Serum	Yes (other)	
18	Belgium	1984-1985	Belgian Interuniversity Research on Nutrition and Health	National	40-69	40-69	3,584	3,135	3,542	3,121	3,541	3,120	Extraction	Heparin-Manganese method; Extraction	Serum	Yes (CDC/CRMLN)	
19	Belgium	1985-1987	MONICA, Charleroi	Community	40-59	40-59	173	144	172	144	172	144	Enzymatic	Phosphotungstate-Magnesium (PTA) method; Enzymatic	Serum	Yes (WHO-LRC)	
20	Belgium	1985-1987	MONICA, Ghent	Community	40-59	40-59	266	221	262	220	262	220	Enzymatic	Phosphotungstate-Magnesium (PTA) method; Enzymatic	Serum	Yes (WHO-LRC)	
21	Belgium	1985-1990	Flemish Study on Environment, Genes and Health Outcomes	Community	40-79	40-79	408	403	292	292	292	292	Enzymatic	PEG 6000 method; Enzymatic	Serum	Yes (other)	

	Country	Data years	Survey/Study name/Citation	Level of representati-veness	Age range as used for the analysis		Sample size (Total cholesterol)		Sample size (HDL cholesterol)		Sample size (Non-HDL cholesterol)		Method used to measure		Whether lipids were measured in serum or plasma samples	Participating to a lipid standardisation programme or quality control schemes	Note
					Male	Female	Male	Female	Male	Female	Male	Female	Total cholesterol	HDL cholesterol (separation and quantification methods)			
22	Belgium	1987-1990	MONICA, Charleroi	Community	40-59	40-59	161	140	160	140	160	140	Enzymatic	Phosphotungstate-Magnesium (PTA) method; Enzymatic	Serum	Yes (WHO-LRC)	
23	Belgium	1988-1990	MONICA, Ghent	Community	40-59	40-59	207	227	207	227	207	227	Enzymatic	Phosphotungstate-Magnesium (PTA) method; Enzymatic	Serum	Yes (WHO-LRC)	
24	Belgium	1990-1993	MONICA, Charleroi	Community	40-59	40-59	151	148	151	144	151	144	Enzymatic	Phosphotungstate-Magnesium (PTA) method; Enzymatic	Serum	Yes (WHO-LRC)	
25	Belgium	1990-1992	MONICA, Ghent	Community	40-59	40-59	236	237	236	237	236	237	Enzymatic	Phosphotungstate-Magnesium (PTA) method; Enzymatic	Serum	Yes (WHO-LRC)	
26	Belgium	1991-1994	Flemish Study on Environment, Genes and Health Outcomes	Community	40-79	40-79	281	288	280	288	280	288	Enzymatic	PEG 6000 method; Enzymatic	Serum	Yes (other)	
27	Belgium	1996-1998	Flemish Study on Environment, Genes and Health Outcomes	Community	40-79	40-79	188	183	186	183	186	183	Enzymatic	Homogeneous assay (Antibody, two reagents)	Serum	Yes (other)	
28	Belgium	1998	Flemish Study on Environment, Genes and Health Outcomes	Community	40-79	40-79	276	306	276	306	276	306	Enzymatic	Homogeneous assay (Antibody, two reagents)	Serum	Yes (other)	
29	Belgium	1999-2001	Flemish Study on Environment, Genes and Health Outcomes	Community	40-79	40-79	122	130	122	130	122	130	Enzymatic	Homogeneous assay (Antibody, two reagents)	Serum	Yes (other)	
30	Belgium	2001	Flemish Study on Environment, Genes and Health Outcomes	Community	40-69	40-69	121	109	121	109	121	109	Enzymatic	Homogeneous assay (Antibody, two reagents)	Serum	Yes (other)	
31	Belgium	2002-2003	Flemish Study on Environment, Genes and Health Outcomes	Community	40-79	40-79	92	102	92	102	92	102	Enzymatic	Homogeneous assay (Antibody, two reagents)	Serum	Yes (other)	
32	Belgium	2003	The European Male Ageing Study	Community	40-79		444		443		443		Unknown	Unknown	Unknown	Unknown	
33	Belgium	2002-2005	Flemish Study on Environment, Genes and Health Outcomes	Community	40-79	40-79	261	264	260	264	260	264	Enzymatic	Homogeneous assay (Antibody, two reagents)	Serum	Yes (other)	
34	Belgium	2005-2008	Flemish Study on Environment, Genes and Health Outcomes	Community	40-79	40-79	323	347	323	347	323	347	Enzymatic	Homogeneous assay (Antibody, two reagents)	Serum	Yes (other)	
35	Belgium	2008	The European Male Ageing Study	Community	40-79		359		360		358		Unknown	Unknown	Unknown	Unknown	
36	Belgium	2009-2013	Flemish Study on Environment, Genes and Health Outcomes	Community	40-79	40-79	256	272	255	271	255	271	Enzymatic	Homogeneous assay (2 assays used: Antibody, two reagents and Dextran sulphate-Magnesium + PEG-coupled enzymes)	Serum	Yes (other)	
37	Belgium	2010-2015	Flemish Study on Environment, Genes and Health Outcomes	Community	40-79	40-79	287	279	287	279	287	279	Enzymatic	Homogeneous assay (2 assays used: Antibody, two reagents and Dextran sulphate-Magnesium + PEG-coupled enzymes)	Serum	Yes (other)	
38	Canada	1985-1988	MONICA, Halifax	Community	40-59	40-59	240	235	238	235	238	235	Enzymatic	Heparin-Manganese method; Enzymatic	Serum	Yes (WHO-LRC)	
39	Canada	1986-1992	Canada Heart Health Survey	National	40-69	40-69	3,452	3,394	3,428	3,370	3,427	3,370	LRC method/Enzymatic	Heparin-Manganese method; Enzymatic	Plasma	Yes (CDC/CRMLN)	
40	Canada	1995	MONICA, Halifax	Community	40-59	40-59	143	155	143	155	143	155	Enzymatic	Dextran sulphate-Magnesium method; Enzymatic	Serum	Yes (WHO-LRC)	

	Country	Data years	Survey/Study name/Citation	Level of representativeness	Age range as used for the analysis		Sample size (Total cholesterol)		Sample size (HDL cholesterol)		Sample size (Non-HDL cholesterol)		Method used to measure		Whether lipids were measured in serum or plasma samples	Participating to a lipid standardisation programme or quality control schemes	Note
					Male	Female	Male	Female	Male	Female	Male	Female	Total cholesterol	HDL cholesterol (separation and quantification methods)			
41	Canada	1995-1997	Canadian Multicentre Osteoporosis Study (CaMos)	Community	40-79	40-79	133	344	133	344	133	344	Enzymatic	Homogeneous assay (Accelerator Selective Detergent; Enzymatic)	Serum	No	
42	Canada	2005-2008	Canadian Multicentre Osteoporosis Study (CaMos)	Subnational	40-79	40-79	528	1,288	528	1,288	528	1,288	Enzymatic	Homogeneous assay (Accelerator Selective Detergent; Enzymatic)	Serum	No	
43	Canada	2007-2009	Canadian Health Measures Survey, Cycle 1	National	40-79	40-79	1,104	1,183	1,104	1,183	1,104	1,183	Enzymatic	Phosphotungstate-Magnesium (PTA) method; Enzymatic	Serum	Yes (other)	
44	Canada	2009-2011	Canadian Health Measures Survey, Cycle 2	National	40-79	40-79	1,120	1,180	1,122	1,181	1,120	1,180	Enzymatic	Phosphotungstate-Magnesium (PTA) method; Enzymatic	Serum	Yes (other)	
45	Canada	2012-2013	Canadian Health Measures Survey, Cycle 3	National	40-79	40-79	1,050	1,062	1,050	1,062	1,050	1,062	Enzymatic	Phosphotungstate-Magnesium (PTA) method; Enzymatic	Serum	Yes (CDC/CRMLN)	
46	Canada	2014-2015	Canadian Health Measures Survey, Cycle 4	National	40-79	40-79	1,020	1,027	1,019	1,027	1,019	1,027	Enzymatic	Phosphotungstate-Magnesium (PTA) method; Enzymatic	Serum	Yes (CDC/CRMLN)	
47	China	1983	Sino-MONICA Shanghai	Community	40-59	40-59	406	398	404	397	404	397	Enzymatic	Phosphotungstate-Magnesium (PTA) method; Enzymatic	Serum	Yes (WHO-LRC)	
48	China	1984-1985	Sino-MONICA Beijing	Community	40-59	40-59	441	445	440	443	440	443	Enzymatic	Phosphotungstate-Magnesium (PTA) method; Enzymatic	Serum	Yes (WHO-LRC)	
49	China	1988	Sino-MONICA Hebei	Community	40-59		356		345		345		Enzymatic	Phosphotungstate-Magnesium (PTA) method; Enzymatic	Serum	Yes (WHO-LRC)	
50	China	1988	Sino-MONICA Heilongjiang	Community	40-59	40-59	432	426	432	426	432	426	Enzymatic	Phosphotungstate-Magnesium (PTA) method; Enzymatic	Serum	Yes (WHO-LRC)	
51	China	1988	Sino-MONICA Henan	Community	40-59	40-59	186	240	186	240	186	240	Enzymatic	Phosphotungstate-Magnesium (PTA) method; Enzymatic	Serum	Yes (WHO-LRC)	
52	China	1988	Sino-MONICA Neimenggu	Community	40-59	40-59	215	207	208	207	208	207	Enzymatic	Phosphotungstate-Magnesium (PTA) method; Enzymatic	Serum	Yes (WHO-LRC)	
53	China	1988	Sino-MONICA Sichuan	Community	40-59	40-59	169	186	169	186	169	186	Enzymatic	Phosphotungstate-Magnesium (PTA) method; Enzymatic	Serum	Yes (WHO-LRC)	
54	China	1988	Sino-MONICA Shandong	Community	40-59	40-59	87	94	87	94	87	94	Enzymatic	Phosphotungstate-Magnesium (PTA) method; Enzymatic	Serum	Yes (WHO-LRC)	
55	China	1986-1989	Sino-MONICA Shanghai	Community	40-59	40-59	409	441	409	441	409	441	Enzymatic	Phosphotungstate-Magnesium (PTA) method; Enzymatic	Serum	Yes (WHO-LRC)	
56	China	1988-1989	Sino-MONICA Beijing	Community	40-59	40-59	358	466	355	466	355	465	Enzymatic	Phosphotungstate-Magnesium (PTA) method; Enzymatic	Serum	Yes (WHO-LRC)	

	Country	Data years	Survey/Study name/Citation	Level of representati-veness	Age range as used for the analysis		Sample size (Total cholesterol)		Sample size (HDL cholesterol)		Sample size (Non-HDL cholesterol)		Method used to measure		Whether lipids were measured in serum or plasma samples	Participating to a lipid standardisation programme or quality control schemes	Note
					Male	Female	Male	Female	Male	Female	Male	Female	Total cholesterol	HDL cholesterol (separation and quantification methods)			
57	China	1989	Sino-MONICA Fujian	Community	40-59	40-59	92	88	92	88	92	88	Enzymatic	Phosphotungstate-Magnesium (PTA) method; Enzymatic	Serum	Yes (WHO-LRC)	
58	China	1988-1989	Sino-MONICA Jilin	Community	40-59	40-59	211	234	201	220	201	220	Enzymatic	Phosphotungstate-Magnesium (PTA) method; Enzymatic	Serum	Yes (WHO-LRC)	
59	China	1989	Sino-MONICA Jiangsu	Community	40-59	40-59	189	184	189	184	189	184	Enzymatic	Phosphotungstate-Magnesium (PTA) method; Enzymatic	Serum	Yes (WHO-LRC)	
60	China	1988-1989	Sino-MONICA Jiangxi	Community	40-59	40-59	187	205	187	205	187	205	Enzymatic	Phosphotungstate-Magnesium (PTA) method; Enzymatic	Serum	Yes (WHO-LRC)	
61	China	1988-1989	Sino-MONICA Liaoning	Community	40-59	40-59	360	381	360	381	360	380	Enzymatic	Phosphotungstate-Magnesium (PTA) method; Enzymatic	Serum	Yes (WHO-LRC)	
62	China	1991	Sino-MONICA Shanghai	Community	40-59	40-59	384	441	384	441	384	441	Enzymatic	Phosphotungstate-Magnesium (PTA) method; Enzymatic	Serum	Yes (WHO-LRC)	
63	China	1992-1993	Anzhen 02 Cohort Study	Community	40-59	40-59	1,435	1,271	1,430	1,270	1,430	1,268	Unknown	Unknown	Serum	Unknown	
64	China	1991-1992	Fangshan Cohort Study	Community	40-79	40-79	163	366	153	351	151	347	Unknown	Unknown	Serum	Unknown	
65	China	1992	Huashan Study	Community	40-69	40-69	398	436	395	434	395	434	Unknown	Unknown	Serum	Unknown	
66	China	1992	Sino-MONICA Sichuan	Community	40-59	40-59	424	311	424	311	424	311	Enzymatic	Phosphotungstate-Magnesium (PTA) method; Enzymatic	Serum	Yes (WHO-LRC)	
67	China	1993	Sino-MONICA Anhui	Community	40-59	40-59	85	84	85	84	85	84	Enzymatic	Phosphotungstate-Magnesium (PTA) method; Enzymatic	Serum	Yes (WHO-LRC)	
68	China	1993	Sino-MONICA Beijing	Community	40-59	40-59	306	435	306	435	306	435	Enzymatic	Phosphotungstate-Magnesium (PTA) method; Enzymatic	Serum	Yes (WHO-LRC)	
69	China	1993	Sino-MONICA Jiangsu	Community	40-59	40-59	261	171	261	171	261	171	Enzymatic	Phosphotungstate-Magnesium (PTA) method; Enzymatic	Serum	Yes (WHO-LRC)	
70	China	1993	Sino-MONICA Liaoning	Community	40-59	40-59	265	237	265	237	265	237	Enzymatic	Phosphotungstate-Magnesium (PTA) method; Enzymatic	Serum	Yes (WHO-LRC)	
71	China	2000-2001	The International Collaborative Study of Cardiovascular Disease in ASIA	National	40-69	40-69	4,220	4,616	4,218	4,615	4,215	4,612	Enzymatic	Dextran sulphate-Magnesium method; Enzymatic	Serum	Yes (CDC/CRMLN)	
72	China	2002	China National Nutrition and Health Survey	National	40-79	40-79	9,719	11,194	9,741	11,223	9,715	11,190	Enzymatic	Homogeneous assay	Plasma	Yes (CDC/CRMLN)	
73	China	2006	Beijing Eye Study	Community	50-79	50-79	354	559	361	567	354	558	Enzymatic	Homogeneous assay (PEG-coupled enzymes)	Serum	Unknown	
74	China	2008	China Health and Retirement Longitudinal Study (CHARLS), pilot survey	Subnational	50-79	50-79	347	358	336	357	336	355	Cardiocheck	Cardiocheck	Capillary	Unknown	
75	China	2007-2008	China National Diabetes and Metabolic Disorders Study; Yang et al., Circulation 2012; 125: 2212-21	National	40-79	40-79	6,970	10,538	6,970	10,538	6,970	10,538	Enzymatic	Unknown	Serum	Yes (other)	1

	Country	Data years	Survey/Study name/Citation	Level of representati-veness	Age range as used for the analysis		Sample size (Total cholesterol)		Sample size (HDL cholesterol)		Sample size (Non-HDL cholesterol)		Method used to measure		Whether lipids were measured in serum or plasma samples	Participating to a lipid standardisation programme or quality control schemes	Note
					Male	Female	Male	Female	Male	Female	Male	Female	Total cholesterol	HDL cholesterol (separation and quantification methods)			
76	China	2009	China Health and Nutrition Study	National	40-79	40-79	1,885	2,174	1,880	2,167	1,879	2,167	Enzymatic	Homogeneous assay (alpha-Cyclodextrin + PEG-coupled enzymes)	Serum	Yes (other)	4
77	China	2010	China Noncommunicable Disease Surveillance	National	40-79	40-79	20,047	25,259	20,062	25,329	19,998	25,232	Enzymatic	Unknown; Enzymatic	Serum	Unknown	
78	China	2011	Beijing Eye Study	Community	50-79	50-79	406	606	406	606	406	606	Enzymatic	Homogeneous assay (PEG-coupled enzymes)	Serum	Unknown	
79	China	2011-2012	China Health and Retirement Longitudinal Study (CHARLS), baseline survey	National	50-79	50-79	1,584	1,858	1,583	1,859	1,580	1,856	Enzymatic	Homogeneous assay (alpha-Cyclodextrin + PEG-coupled enzymes)	Plasma	Yes (other)	
80	China	2012-2013	The Kailuan Study	Community	40-79	40-79	40,331	12,057	40,192	12,037	40,177	12,035	Enzymatic	Homogeneous assay	Serum	Unknown	
81	China	2014-2015	The Kailuan Study	Community	40-79	40-79	31,110	9,335	31,060	9,332	31,034	9,328	Enzymatic	Homogeneous assay (Catalase)	Plasma	Yes (CDC/CRMLN)	
82	Czech Republic	1985	MONICA, Czech Republic	National	40-59	40-59	634	651	634	651	634	651	Enzymatic	Updated phosphotungstate-Magnesium method after the Boehringer-Ms (PTA 543004); Enzymatic	Serum	Yes (WHO-LRC)	
83	Czech Republic	1988	MONICA, Czech Republic	National	40-59	40-59	702	705	702	705	701	705	Enzymatic	Updated phosphotungstate-Magnesium method after the Boehringer-Ms (PTA 543004); Enzymatic	Serum	Yes (WHO-LRC)	
84	Czech Republic	1992	MONICA, Czech Republic	National	40-59	40-59	602	615	597	613	597	613	Enzymatic	Updated phosphotungstate-Magnesium method after the Boehringer-Ms (PTA 543004); Enzymatic	Serum	Yes (WHO-LRC)	
85	Czech Republic	1997-1998	Czech post-MONICA	National	40-59	40-59	899	987	894	987	894	987	Enzymatic	Phosphotungstate-Magnesium (PTA) method; Enzymatic	Serum	Yes (CDC/CRMLN)	
86	Czech Republic	2000-2001	Czech post-MONICA	National	40-59	40-59	944	946	940	941	940	941	Enzymatic	Phosphotungstate-Magnesium (PTA) method; Enzymatic	Serum	Yes (CDC/CRMLN)	
87	Czech Republic	2006-2009	Czech post-MONICA	National	40-59	40-59	934	1,022	922	1,003	922	1,003	Enzymatic	Phosphotungstate-Magnesium (PTA) method; Enzymatic	Serum	Yes (CDC/CRMLN)	
88	Czech Republic	2015-2018	MONICA	National	40-59	40-59	673	761	672	760	672	760	Enzymatic	Homogeneous assay (Dextran sulphate-Magnesium + PEG-coupled enzymes)	Serum	Yes (CDC/CRMLN)	
89	Finland	1972	North Karelia project	Subnational	40-59	40-59	3,033	3,328					Direct		Serum	No	
90	Finland	1977	North Karelia project	Subnational	40-59	40-59	3,015	3,315					Direct		Serum	Yes (other)	
91	Finland	1982	MONICA, North Karelia/Kuopio/Turku/Loimaa	Subnational	40-59	40-59	2,346	2,349	2,342	2,348	2,342	2,348	Enzymatic	Dextran sulphate-Magnesium method; Enzymatic	Serum	Yes (WHO-LRC)	
92	Finland	1987	MONICA, North Karelia/Kuopio/Turku/Loimaa	Subnational	40-59	40-59	1,553	1,663	1,552	1,663	1,552	1,663	Enzymatic	Dextran sulphate-Magnesium method; Enzymatic	Serum	Yes (WHO-LRC)	
93	Finland	1992	The National FINRISK Study	Subnational	40-59	40-59	1,529	1,589	1,529	1,589	1,529	1,589	Enzymatic	Dextran sulphate-Magnesium method; Enzymatic	Serum	Yes (WHO-LRC)	
94	Finland	1997	The National FINRISK Study	National	40-69	40-69	1,835	1,949	1,833	1,949	1,833	1,949	Enzymatic	Dextran sulphate-Magnesium method; Enzymatic	Serum	Yes (WHO-LRC)	

	Country	Data years	Survey/Study name/Citation	Level of representati-veness	Age range as used for the analysis		Sample size (Total cholesterol)		Sample size (HDL cholesterol)		Sample size (Non-HDL cholesterol)		Method used to measure		Whether lipids were measured in serum or plasma samples	Participating to a lipid standardisation programme or quality control schemes	Note
					Male	Female	Male	Female	Male	Female	Male	Female	Total cholesterol	HDL cholesterol (separation and quantification methods)			
95	Finland	1996-1998	Savitaipale Study, Baseline	Community	40-59	40-59	443	458	442	457	442	457	Enzymatic	Homogeneous assay (Dextran sulphate-Magnesium + PEG-coupled enzymes)	Serum	Yes (other)	
96	Finland	2000	Viiri et al., Atherosclerosis 2005; 179: 161-7	Community	50-59		74						Enzymatic		Serum	Unknown	
97	Finland	2000	Viiri et al., Atherosclerosis 2005; 179: 161-7	Community	50-59		101						Enzymatic		Serum	Unknown	
98	Finland	2000	Viiri et al., Atherosclerosis 2005; 179: 161-7	Community	50-59		42						Enzymatic		Serum	Unknown	
99	Finland	2000-2001	Health 2000 Survey	National	40-79	40-79	1,439	1,551	1,439	1,551	1,439	1,551	Enzymatic	Homogeneous assay (Dextran sulphate-Magnesium + PEG-coupled enzymes)	Serum	Yes (other)	
100	Finland	2002	The National FINRISK Study	National	40-69	40-69	1,597	1,831	1,596	1,831	1,596	1,831	Enzymatic	Homogeneous assay (PEG-coupled enzymes)	Serum	Yes (CDC/CRMLN)	
101	Finland	2007	The National FINRISK Study	National	40-69	40-69	1,248	1,397	1,248	1,397	1,248	1,397	Enzymatic	Homogeneous assay (PEG-coupled enzymes)	Serum	Yes (CDC/CRMLN)	
102	Finland	2012	The National FINRISK Study	National	40-69	40-69	1,086	1,231	1,086	1,230	1,086	1,230	Enzymatic	Homogeneous assay (Accelerator Selective Detergent; Enzymatic)	Serum	Yes (CDC/CRMLN)	
103	Finland	2011-2012	Health 2011 Survey	National	40-79	40-79	884	1,099	883	1,099	883	1,099	Enzymatic	Homogeneous assay (Accelerator Selective Detergent; Enzymatic)	Serum	Yes (CDC/CRMLN)	
104	France	1985-1987	MONICA, Strasbourg	Subnational	40-59	40-59	446	431	444	430	444	430	Enzymatic	Phosphotungstate-Magnesium (PTA) method/Updated phosphotungstate-Magnesium method after the Boehringer-Ms (PTA 543004); Enzymatic	Plasma	Yes (WHO-LRC)	
105	France	1985-1987	MONICA, Toulouse	Subnational	40-59	40-59	422	394	417	391	417	391	Enzymatic	Phosphotungstate-Magnesium (PTA) method; Enzymatic	Plasma	Yes (WHO-LRC)	
106	France	1986-1989	MONICA, Lille	Community	40-59	40-59	353	252	350	250	350	250	Enzymatic	Updated phosphotungstate-Magnesium method after the Boehringer-Ms (PTA 543004); Enzymatic	Plasma	Yes (WHO-LRC)	
107	France	1988-1991	MONICA, Toulouse	Subnational	40-59	40-59	394		394		394		Enzymatic	Updated phosphotungstate-Magnesium method after the Boehringer-Ms (PTA 543004); Enzymatic	Plasma	Yes (WHO-LRC)	
108	France	1994-1996	MONICA, Toulouse	Subnational	40-59	40-59	412	391	412	391	412	391	Enzymatic	Updated phosphotungstate-Magnesium method after the Boehringer-Ms (PTA 543004); Enzymatic	Serum	Yes (WHO-LRC)	

	Country	Data years	Survey/Study name/Citation	Level of representati-veness	Age range as used for the analysis		Sample size (Total cholesterol)		Sample size (HDL cholesterol)		Sample size (Non-HDL cholesterol)		Method used to measure		Whether lipids were measured in serum or plasma samples	Participating to a lipid standardisation programme or quality control schemes	Note
					Male	Female	Male	Female	Male	Female	Male	Female	Total cholesterol	HDL cholesterol (separation and quantification methods)			
109	France	1995-1996	Multinational mONItoring of trends and determinants of CArdiovascular disease in Lille (MONICA Lille)	Community	40-59	40-59	388	412	385	411	385	411	Enzymatic	Updated phosphotungstate-Magnesium method after the Boehringer-Ms (PTA 543004); Enzymatic	Serum	Yes (WHO-LRC)	
110	France	1995-1997	MONICA, Strasbourg	Subnational	40-59	40-59	382	356	374	344	374	344	Enzymatic	Updated phosphotungstate-Magnesium method after the Boehringer-Ms (PTA 543004); Enzymatic	Serum	Yes (WHO-LRC)	
111	France	2004-2006	National Monitoring of Arterial Risk in Lille (MONA LISA Lille)	Community	40-69	40-69	413	416	413	416	413	416	Enzymatic	Phosphotungstate-Magnesium (PTA) method; Enzymatic	Serum	Yes (other)	
112	France	2005-2007	National Monitoring of Arterial Risk in Bas-Rhin (MONA LISA Bas-Rhin)	Subnational	40-69	40-69	407	396	407	396	407	396	Enzymatic	Phosphotungstate-Magnesium (PTA) method; Enzymatic	Serum	Yes (other)	
113	France	2005-2007	Monitoring National du Risque Artériel (MONA LISA study Haute-Garonne)	Subnational	40-69	40-69	465	426	465	426	465	426	Enzymatic	Phosphotungstate-Magnesium (PTA) method; Enzymatic	Serum	Yes (other)	
114	France	2006-2007	Etude Nationale Nutrition Santé	National	40-69	40-69	363	658	359	649	359	649	Enzymatic	Homogeneous assay (Antibody, two reagents)	Serum	No	
115	France	2011-2013	Enquête LIttorale Souffle Air Biologie EnvironnemenT (ELISABET) Dunkerque	Community	40-59	40-59	573	612	573	612	573	612	Enzymatic	Homogeneous assay (Accelerator Selective Detergent; Enzymatic)	Serum	Yes (other)	
116	France	2011-2013	Enquête LIttorale Souffle Air Biologie EnvironnemenT (ELISABET) Lille	Community	40-59	40-59	581	681	581	682	581	681	Enzymatic	Homogeneous assay (Accelerator Selective Detergent; Enzymatic)	Serum	Yes (other)	
117	Germany	1982	MONICA, Erfurt	Community	40-59	40-59	48	47					Direct		Serum	Yes (WHO-LRC)	
118	Germany	1982-1984	MONICA, Chemnitz	Community	40-59	40-59	136	168	132	162	132	161	Direct	PEG 6000 method; Direct	Serum	Yes (WHO-LRC)	
119	Germany	1982-1984	MONICA, Zwickau	Community	40-59	40-59	131	128	128	133	126	127	Direct	PEG 6000 method; Direct	Serum	Yes (WHO-LRC)	
120	Germany	1984	German Cardiovascular Prevention Study (GCP) - National Health Survey 1984	Subnational	40-69	40-69	1,568	1,484	1,445	1,359	1,443	1,357	Enzymatic	Phosphotungstate-Magnesium (PTA) method; Enzymatic	Serum	Yes (WHO-LRC)	
121	Germany	1984-1985	MONICA, Berlin-Lichtenberg	Community	40-59	40-59	405	379	393	372	393	370	Direct	Phosphotungstate-Magnesium (PTA) method; Enzymatic	Serum	Yes (WHO-LRC)	
122	Germany	1984	MONICA, Bremen North/West	Community	40-59	40-59	453	454	412	405	405	400	Enzymatic	Updated phosphotungstate-Magnesium method after the Boehringer-Ms (PTA 543004); Enzymatic	Serum	Yes (WHO-LRC)	
123	Germany	1983-1984	MONICA, Halle County	Subnational	40-59	40-59	584	576	535	528	528	520	Direct	Phosphotungstate-Magnesium (PTA) method; Direct	Serum	Yes (WHO-LRC)	
124	Germany	1982-1985	MONICA, Rest of Karl-Marx-Stadt County	Subnational	40-59	40-59	292	334					Direct		Serum	Yes (WHO-LRC)	
125	Germany	1982-1985	MONICA, Rest of DDR-MONICA	Subnational	40-59	40-59	125	143	59	67	59	67	Direct	Phosphotungstate-Magnesium (PTA) method; Direct	Serum	Yes (WHO-LRC)	

	Country	Data years	Survey/Study name/Citation	Level of representati-veness	Age range as used for the analysis		Sample size (Total cholesterol)		Sample size (HDL cholesterol)		Sample size (Non-HDL cholesterol)		Method used to measure		Whether lipids were measured in serum or plasma samples	Participating to a lipid standardisation programme or quality control schemes	Note
					Male	Female	Male	Female	Male	Female	Male	Female	Total cholesterol	HDL cholesterol (separation and quantification methods)			
126	Germany	1984-1985	MONICA, Augsburg	Community	40-59	40-59	1,035	977	1,034	1,015	985	965	Enzymatic	Phosphotungstate-Magnesium (PTA) method; Enzymatic	Serum	Yes (WHO-LRC)	
127	Germany	1984-1986	MONICA, Cottbus County	Community	40-59	40-59	331	374	326	371	326	371	Direct	Phosphotungstate-Magnesium (PTA) method; Direct	Serum	Yes (WHO-LRC)	
128	Germany	1987-1988	MONICA, Erfurt	Community	40-59	40-59	445	434	444	433	444	433	Direct	Phosphotungstate-Magnesium (PTA) method; Direct	Serum	Yes (WHO-LRC)	
129	Germany	1988	German Cardiovascular Prevention Study (GCP) - National Health Survey 1988	Subnational	40-69	40-69	1,613	1,643	1,590	1,633	1,590	1,631	Enzymatic	Phosphotungstate-Magnesium (PTA) method; Enzymatic	Serum	Yes (WHO-LRC)	
130	Germany	1988	MONICA, Berlin-Lichtenberg	Community	40-59	40-59	393	389	393	389	393	389	Direct	Phosphotungstate-Magnesium (PTA) method; Enzymatic	Serum	Yes (WHO-LRC)	
131	Germany	1988	MONICA, Bremen North/West	Community	40-69	40-69	388	416	364	381	364	381	Direct/Enzymatic	Updated phosphotungstate-Magnesium method after the Boehringer-Ms (PTA 543004); Enzymatic	Serum	Yes (WHO-LRC)	
132	Germany	1988	MONICA, Bremen Center/South/East	Community	40-69	40-69	317	366	309	356	309	356	Direct/Enzymatic	Updated phosphotungstate-Magnesium method after the Boehringer-Ms (PTA 543004); Enzymatic	Serum	Yes (WHO-LRC)	
133	Germany	1988	MONICA, Chemnitz	Community	40-59	40-59	141	202	142	202	141	201	Direct	PEG 6000 method; Direct	Serum	Yes (WHO-LRC)	
134	Germany	1988	MONICA, Zwickau	Community	40-59	40-59	97	120	96	117	96	116	Direct	PEG 6000 method; Direct	Serum	Yes (WHO-LRC)	
135	Germany	1989-1990	MONICA, Cottbus County	Community	40-59	40-59	258	241	256	236	256	236	Direct	Phosphotungstate-Magnesium (PTA) method; Direct	Serum	Yes (WHO-LRC)	
136	Germany	1988-1989	MONICA, Halle County	Subnational	40-59	40-59	488	576	481	573	481	571	Direct	PEG 6000 method; Direct	Serum	Yes (WHO-LRC)	
137	Germany	1988-1989	MONICA, Rest of Karl-Marx-Stadt County	Subnational	40-59	40-59	258	282	257	283	257	282	Direct	PEG 6000 method; Direct	Serum	Yes (WHO-LRC)	
138	Germany	1989-1990	MONICA, Augsburg	Community	40-59	40-59	1,014	996	1,003	991	1,003	991	Enzymatic	Phosphotungstate-Magnesium (PTA) method; Enzymatic	Serum	Yes (WHO-LRC)	
139	Germany	1991-1992	MONICA, Bremen North/West	Community	40-69	40-69	352	390	325	360	325	359	Enzymatic	Updated phosphotungstate-Magnesium method after the Boehringer-Ms (PTA 543004); Enzymatic	Serum	Yes (WHO-LRC)	
140	Germany	1991-1992	MONICA, Bremen Center/South/East	Community	40-69	40-69	330	324	321	314	321	314	Enzymatic	Updated phosphotungstate-Magnesium method after the Boehringer-Ms (PTA 543004); Enzymatic	Serum	Yes (WHO-LRC)	
141	Germany	1991-1992	German Cardiovascular Prevention Study (GCP) - National Health Survey 1991	Subnational	40-69	40-69	1,590	1,628	1,516	1,559	1,515	1,558	Enzymatic	Phosphotungstate-Magnesium (PTA) method; Enzymatic	Serum	Yes (WHO-LRC)	
142	Germany	1991-1992	First National Examination of life conditions, Environment and Health in East Germany 1991/92	Subnational	40-69	40-69	651	684	591	626	591	625	Enzymatic	Phosphotungstate-Magnesium (PTA) method; Enzymatic	Serum	Yes (WHO-LRC)	

	Country	Data years	Survey/Study name/Citation	Level of representati-veness	Age range as used for the analysis		Sample size (Total cholesterol)		Sample size (HDL cholesterol)		Sample size (Non-HDL cholesterol)		Method used to measure		Whether lipids were measured in serum or plasma samples	Participating to a lipid standardisation programme or quality control schemes	Note
					Male	Female	Male	Female	Male	Female	Male	Female	Total cholesterol	HDL cholesterol (separation and quantification methods)			
143	Germany	1991-1992	MONICA, Erfurt	Community	40-59	40-59	284	285	284	284	284	284	Unknown	Phosphotungstate-Magnesium (PTA) method; Enzymatic	Serum	Yes (WHO-LRC)	
144	Germany	1993-1994	MONICA, Chemnitz	Community	40-59	40-59	223	256	223	256	223	256	Enzymatic	Updated phosphotungstate-Magnesium method after the Boehringer-Ms (PTA 543004); Enzymatic	Serum	Yes (WHO-LRC)	
145	Germany	1993-1994	MONICA, Zwickau	Community	40-59	40-59	60	64	60	64	60	64	Enzymatic	Updated phosphotungstate-Magnesium method after the Boehringer-Ms (PTA 543004); Enzymatic	Serum	Yes (WHO-LRC)	
146	Germany	1994-1995	MONICA, Augsburg	Community	40-59	40-59	955	1,036	951	1,036	951	1,036	Enzymatic	Updated phosphotungstate-Magnesium method after the Boehringer-Ms (PTA 543004); Enzymatic	Serum	Yes (WHO-LRC)	
147	Germany	1997-1999	German National Health Interview and Examination Survey (GNHIES98)	National	40-79	40-79	1,940	2,122	1,937	2,122	1,937	2,122	Enzymatic	Homogeneous assay (Antibody, two reagents)	Serum	Yes (other)	
148	Germany	1997-2001	Study of Health in Pomerania (SHIP-0) baseline study	Subnational	40-79	40-79	1,461	1,446	1,456	1,441	1,455	1,441	Enzymatic	Phosphotungstate-Magnesium (PTA) method; Enzymatic	Serum	Yes (other)	
149	Germany	1999-2001	KORA S4 Study: Kooperative Research in the Region of Augsburg Survey 4	Community	40-69	40-69	1,260	1,316	1,257	1,314	1,257	1,314	Enzymatic	Phosphotungstate-Magnesium (PTA) method; Enzymatic	Serum	Unknown	
150	Germany	2000-2002	Epidemiological study of the chances of prevention, early recognition and optimal treatment of chronic diseases in an elderly population (ESTHER)	Subnational	50-69	50-69	3,661	4,442	2,351	2,730	2,334	2,708	Enzymatic	Unknown; Enzymatic	Serum	Unknown	
151	Germany	2002	Echinoccoccus Multilocularis and Internal Diseases in Leutkirch	Community	40-59	40-59	378	439	378	439	378	439	Enzymatic	Heparin-Manganese method; Enzymatic	Serum	Yes (CDC/CRMLN)	
152	Germany	2002-2006	Study of Health in Pomerania (SHIP-1) 5 year follow-up	Subnational	40-79	40-79	1,183	1,261					Enzymatic	Electrophoresis	Serum	Yes (other)	2
153	Germany	2006-2008	KORA F4 Study: Kooperative Research in the Region of Augsburg Follow-up of Survey 4	Community	40-79	40-79	1,273	1,346	1,273	1,346	1,273	1,346	Enzymatic	Homogeneous assay (Catalase)	Serum	Unknown	
154	Germany	2008-2011	Epidemiological study of the chances of prevention, early recognition and optimal treatment of chronic diseases in an elderly population (ESTHER)	Subnational	60-79	60-79	1,870	2,198	1,870	2,197	1,870	2,197	Enzymatic	Unknown	Serum	Unknown	
155	Germany	2008-2011	German Health Interview and Examination Survey for adults 2008-11 (DEGS1)	National	40-79	40-79	2,453	2,699	2,456	2,699	2,453	2,698	Enzymatic	Homogeneous assay (Accelerator Selective Detergent; Enzymatic)	Serum	Yes (other)	
156	Germany	2008-2012	Study of Health in Pomerania, second cohort (SHIP-TREND)	Subnational	40-79	40-79	1,601	1,657	1,600	1,656	1,600	1,656	Enzymatic	Homogeneous assay	Serum	Yes (other)	
157	Iceland	1970-1971	The Reykjavik Study (Men)	Subnational	40-59		3,295						Enzymatic		Serum	Yes (other)	
158	Iceland	1971-1972	The Reykjavik Study (Women)	Subnational		40-59		3,421					Enzymatic		Serum	Yes (other)	
159	Iceland	1974-1976	The Reykjavik Study (Men)	Subnational	40-69		4,525						Enzymatic		Serum	Yes (other)	
160	Iceland	1977-1979	The Reykjavik Study (Women)	Subnational		50-69		1,983					Enzymatic		Serum	Yes (other)	

	Country	Data years	Survey/Study name/Citation	Level of representati-veness	Age range as used for the analysis		Sample size (Total cholesterol)		Sample size (HDL cholesterol)		Sample size (Non-HDL cholesterol)		Method used to measure		Whether lipids were measured in serum or plasma samples	Participating to a lipid standardisation programme or quality control schemes	Note
					Male	Female	Male	Female	Male	Female	Male	Female	Total cholesterol	HDL cholesterol (separation and quantification methods)			
161	Iceland	1979-1981	The Reykjavik Study (Men)	Subnational	50-69		1,595						Enzymatic		Serum	Yes (other)	
162	Iceland	1983	MONICA, Arnes County	Community	40-59	40-59	196	231	194	231	194	231	Extraction	Heparin-Manganese method; Extraction	Serum	Yes (WHO-LRC)	
163	Iceland	1983	MONICA, Reykjavik	Subnational	40-59	40-59	226	241	224	241	224	241	Extraction	Heparin-Manganese method; Extraction	Serum	Yes (WHO-LRC)	
164	Iceland	1981-1984	The Reykjavik Study (Women)	Subnational		50-69		1,685					Enzymatic		Serum	Yes (other)	
165	Iceland	1988-1989	MONICA, Arnes County	Community	40-59	40-59	210	221	209	220	209	220	Enzymatic	Phosphotungstate-Magnesium (PTA) method; Enzymatic	Serum	Yes (WHO-LRC)	
166	Iceland	1988-1989	MONICA, Reykjavik	Subnational	40-59	40-59	222	232	222	232	222	232	Enzymatic	Phosphotungstate-Magnesium (PTA) method; Enzymatic	Serum	Yes (WHO-LRC)	
167	Iceland	1993-1994	MONICA, Arnes County	Community	40-59	40-59	229	259	228	259	228	259	Enzymatic	Phosphotungstate-Magnesium (PTA) method; Enzymatic	Serum	Yes (WHO-LRC)	
168	Iceland	1993-1994	MONICA, Reykjavik	Subnational	40-59	40-59	235	223	235	223	235	223	Enzymatic	Phosphotungstate-Magnesium (PTA) method; Enzymatic	Serum	Yes (WHO-LRC)	
169	Iceland	2001-2003	The Reykjavik Study for the young	Subnational	50-59	50-59	469	495	469	495	469	495	Enzymatic	Homogeneous assay (Dextran sulphate-Magnesium + PEG-coupled enzymes)	Serum	Yes (other)	
170	Iceland	2005-2011	Risk Evaluation For INfarct Estimates (REFINE)	Subnational	40-69	40-69	1,831	1,928	1,830	1,927	1,830	1,927	Enzymatic	Homogeneous assay (Dextran sulphate-Magnesium + PEG-coupled enzymes)	Serum	Yes (other)	
171	Iceland	2010-2012	Risk Evaluation For INfarct Estimates (REFINE) follow-up visit (REFINELO)	Subnational	40-69	40-69	318	328	318	328	318	328	Enzymatic	Homogeneous assay (Dextran sulphate-Magnesium + PEG-coupled enzymes)	Serum	Yes (other)	
172	Italy	1982-1987	MONICA, Latina	Community	40-59	40-59	445	455	445	453	445	453	Enzymatic	Phosphotungstate-Magnesium (PTA) method; Enzymatic	Serum	Yes (WHO-LRC)	
173	Italy	1985	Finland, Italy, Netherlands, Elderly (Fine Italy)	Community	70-79		421		420		420		Enzymatic	Phosphotungstate-Magnesium (PTA) method; Enzymatic	Serum	Yes (WHO-LRC)	
174	Italy	1986	MONICA, Friuli	Subnational	40-59	40-59	488	471	487	464	487	463	Enzymatic	PEG 6000 method; Enzymatic	Serum	Yes (WHO-LRC)	
175	Italy	1986-1987	MONICA-Brianza survey	Subnational	40-59	40-59	430	444	430	445	429	444	Enzymatic	Updated phosphotungstate-Magnesium method after the Boehringer-Ms (PTA 543004); Enzymatic	Serum	Yes (WHO-LRC)	
176	Italy	1989	MONICA, Friuli	Subnational	40-59	40-59	491	481	491	480	489	479	Enzymatic	PEG 6000 method; Enzymatic	Serum	Yes (WHO-LRC)	
177	Italy	1989	Ventimiglia Heart Study	Community	40-79	40-79	308	378	308	378	308	378	Enzymatic	Phosphotungstate-Magnesium (PTA) method; Enzymatic	Plasma	Yes (WHO-LRC)	

	Country	Data years	Survey/Study name/Citation	Level of representati-veness	Age range as used for the analysis		Sample size (Total cholesterol)		Sample size (HDL cholesterol)		Sample size (Non-HDL cholesterol)		Method used to measure		Whether lipids were measured in serum or plasma samples	Participating to a lipid standardisation programme or quality control schemes	Note
					Male	Female	Male	Female	Male	Female	Male	Female	Total cholesterol	HDL cholesterol (separation and quantification methods)			
178	Italy	1990	Bruneck Study	Community	40-79	40-79	469	450	469	450	469	450	Enzymatic	Phosphotungstate-Magnesium (PTA) method; Enzymatic	Serum	No	
179	Italy	1983-1996	Malattie cardiovascolari ATerosclerotiche Istituto Superiore di Sanità	Community	40-69	40-69	2,479	2,828	2,476	2,826	2,475	2,826	Enzymatic	Phosphotungstate-Magnesium (PTA) method; Enzymatic	Serum	Yes (WHO-LRC)	
180	Italy	1989-1990	MONICA-Brianza survey	Subnational	40-59	40-59	421	429	423	429	421	429	Enzymatic	Updated phosphotungstate-Magnesium method after the Boehringer-Ms (PTA 543004); Enzymatic	Serum	Yes (WHO-LRC)	
181	Italy	1992-1993	Italian Longitudinal Study on Aging	National	70-79	70-79	861	771	863	762	852	758	Enzymatic	Phosphotungstate-Magnesium (PTA) method; Enzymatic	Serum	No	
182	Italy	1994	MONICA, Friuli	Subnational	40-59	40-59	486	509	485	508	485	507	Enzymatic	PEG 6000 method; Enzymatic	Serum	Yes (WHO-LRC)	
183	Italy	1993-1994	MONICA-Brianza survey	Subnational	40-59	40-59	423	459	423	459	422	459	Enzymatic	Updated phosphotungstate-Magnesium method after the Boehringer-Ms (PTA 543004); Enzymatic	Serum	Yes (WHO-LRC)	
184	Italy	1995	Bruneck Study	Community	50-79	50-79	313	322	313	322	313	322	Enzymatic	Phosphotungstate-Magnesium (PTA) method; Enzymatic	Serum	No	
185	Italy	1995-1996	Italian Longitudinal Study on Aging	National	70-79	70-79	591	506	582	496	580	496	Enzymatic	Phosphotungstate-Magnesium (PTA) method; Enzymatic	Serum	No	
186	Italy	1995-1999	PROgetto Veneto Anziani (PROVA)	Subnational	70-79	70-79	485	738	481	735	481	735	Enzymatic	Dextran sulphate-Magnesium method; Enzymatic	Serum	Yes (other)	
187	Italy	1998-1999	progetto VIP	Community	40-69	40-69	358	353	340	335	340	334	Enzymatic	Homogeneous assay (Antibody, two reagents)	Serum	Yes (other)	
188	Italy	2000	Bruneck Study	Community	50-79	50-79	295	309	295	309	295	309	Enzymatic	Homogeneous assay (Accelerator Selective Detergent; Enzymatic)	Serum	No	
189	Italy	1998-2002	Osservatorio Epidemiologico Cardiovascolare	National	40-69	40-69	3,738	3,624	3,743	3,630	3,737	3,624	Enzymatic	Updated phosphotungstate-Magnesium method after the Boehringer-Ms (PTA 543004); Enzymatic	Serum	Yes (CDC/CRMLN)	
190	Italy	2001-2003	The Study of Asti	Community	50-59	50-59	411	453	411	453	411	453	Enzymatic	Heparin-Manganese method; Enzymatic	Plasma	No	
191	Italy	2000-2003	PROgetto Veneto Anziani (PROVA)	Subnational	70-79	70-79	440	749	361	621	361	620	Enzymatic	Dextran sulphate-Magnesium method; Enzymatic	Serum	Yes (other)	
192	Italy	2003	The European Male Ageing Study	Community	40-79		431		431		431		Unknown	Unknown	Unknown	Unknown	
193	Italy	2002-2005	PROgetto Veneto Anziani (PROVA)	Subnational	70-79	70-79	307	542	308	540	307	540	Enzymatic	Dextran sulphate-Magnesium method; Enzymatic	Serum	Yes (other)	
194	Italy	2005	Bruneck Study	Community	60-79	60-79	181	187	181	187	181	187	Enzymatic	Homogeneous assay (Dextran sulphate-Magnesium + PEG-coupled enzymes)	Serum	No	

	Country	Data years	Survey/Study name/Citation	Level of representativeness	Age range as used for the analysis		Sample size (Total cholesterol)		Sample size (HDL cholesterol)		Sample size (Non-HDL cholesterol)		Method used to measure		Whether lipids were measured in serum or plasma samples	Participating to a lipid standardisation programme or quality control schemes	Note
					Male	Female	Male	Female	Male	Female	Male	Female	Total cholesterol	HDL cholesterol (separation and quantification methods)			
195	Italy	2004-2005	Italian Project on the Epidemiology of Alzheimer's Disease	National	70-79	70-79	709	623					Unknown		Unknown	Unknown	
196	Italy	2004-2005	Vobarno study	Community	60-69	60-69	53	63	53	62	53	62	Enzymatic	Homogeneous assay (Accelerator Selective Detergent; Enzymatic)	Serum	Yes (other)	
197	Italy	2008	The European Male Ageing Study	Community	40-79		262		263		262		Unknown	Unknown	Unknown	Unknown	
198	Italy	2005-2010	Moli-sani Study	Subnational	40-79	40-79	10,459	11,199	10,458	11,200	10,456	11,198	Enzymatic	Homogeneous assay (Immuno-enzymatic)	Serum	Yes (CDC/CRMLN)	
199	Italy	2008-2009	progetto VIP	Community	40-69	40-69	356	372	356	370	354	370	Enzymatic	Homogeneous assay (Antibody, two reagents)	Serum	Yes (other)	
200	Italy	2010	Bruneck Study	Community	60-79	60-79	181	185	181	185	181	185	Enzymatic	Homogeneous assay (Dextran sulphate-Magnesium + PEG-coupled enzymes)	Serum	No	
201	Italy	2009-2010	Grosso et al., J Epidemiol. 2014; 24: 327-33	Community	40-79	40-79	498	822	498	822	498	822	Enzymatic	Heparin-Manganese method; Enzymatic	Plasma	No	
202	Italy	2008-2012	Osservatorio Epidemiologico Cardiovascolare/Health Examination Survey	National	40-79	40-79	3,927	3,912	3,928	3,911	3,926	3,911	Enzymatic	Homogeneous assay (Immuno-enzymatic)	Serum	Yes (CDC/CRMLN)	
203	Italy	2010-2012	CArdiovascular risk MEtabolic syndrome LIver and Autoimmunity diseases (CA.ME.LI.A)	Community	40-69	40-69	291	303	290	304	290	303	Enzymatic	Homogeneous assay (Dextran sulphate-Magnesium + PEG-coupled enzymes)	Serum	No	
204	Italy	2011-2012	Vobarno study	Community	50-59	50-59	87	111	86	111	86	111	Enzymatic	Homogeneous assay (Accelerator Selective Detergent; Enzymatic)	Serum	Yes (other)	
205	Italy	2015	Bruneck Study	Community	70-79	70-79	84	79	84	79	84	79	Enzymatic	Homogeneous assay (Dextran sulphate-Magnesium + PEG-coupled enzymes)	Serum	No	
206	Japan	1980	APCSC-Hisayama	Community	40-79	40-79	3,494	2,435					Unknown		Unknown	Unknown	
207	Japan	1980	National Cardiovascular Survey	National	40-79	40-79	3,376	4,233					Direct		Serum	Yes (CDC/CRMLN)	
208	Japan	1981	APCSC-Hisayama	Community	40-69	40-69	820	1,074					Unknown		Unknown	Unknown	
209	Japan	1980-1983	Aito Town Study	Community	40-69	40-69	672	848	442	449	442	449	Unknown	Unknown	Serum	Unknown	
210	Japan	1985-1986	Akabane Study	Community	40-69	40-69	812	1,022	812	1,022	812	1,022	Unknown	Unknown	Serum	Unknown	
211	Japan	1987	Konan Town Study	Community	40-79	40-79	46	62	46	62	46	62	Unknown	Unknown	Serum	Unknown	
212	Japan	1988	Konan Town Study	Community	40-79	40-79	54	62	54	62	54	62	Unknown	Unknown	Serum	Unknown	
213	Japan	1989	Konan Town Study	Community	40-79	40-79	42	43	42	43	42	43	Unknown	Unknown	Serum	Unknown	
214	Japan	1988-1990	Miyama Cohort Study	Community	40-79	40-79	155	256	151	255	151	255	Unknown	Unknown	Serum	Unknown	
215	Japan	1989	National Nutrition Survey	National	40-79	40-79	2,125	3,044	2,124	3,043	2,123	3,043	Enzymatic	Heparin-Calcium method; Unknown	Serum	Yes (CDC/CRMLN)	
216	Japan	1990	Serum Lipid Survey; Yamamoto et al., J Atheroscler Thromb 2003; 10: 176-85	National	40-79	40-79	14,291	9,971					Enzymatic		Serum	Yes (CDC/CRMLN)	
217	Japan	1990	Konan Town Study	Community	40-79	40-79	20	36	20	36	20	36	Unknown	Unknown	Serum	Unknown	
218	Japan	1990	National Nutrition Survey and National Cardiovascular Survey	National	40-79	40-79	2,594	3,477	2,592	3,477	2,592	3,477	Enzymatic	Heparin-Calcium method; Unknown	Serum	Yes (CDC/CRMLN)	
219	Japan	1991	Konan Town Study	Community	40-79	40-79	77	97	77	97	77	97	Unknown	Unknown	Serum	Unknown	

	Country	Data years	Survey/Study name/Citation	Level of representati-veness	Age range as used for the analysis		Sample size (Total cholesterol)		Sample size (HDL cholesterol)		Sample size (Non-HDL cholesterol)		Method used to measure		Whether lipids were measured in serum or plasma samples	Participating to a lipid standardisation programme or quality control schemes	Note
					Male	Female	Male	Female	Male	Female	Male	Female	Total cholesterol	HDL cholesterol (separation and quantification methods)			
220	Japan	1991	Shigaraki Town Study	Community	40-79	40-79	203	277	203	277	203	277	Unknown	Unknown	Serum	Unknown	
221	Japan	1991	National Nutrition Survey	National	40-79	40-79	2,349	3,250	2,348	3,249	2,348	3,249	Enzymatic	Heparin-Calcium method; Unknown	Serum	Yes (CDC/CRMLN)	
222	Japan	1992	Konan Town Study	Community	40-79	40-79	34	41	34	41	34	41	Unknown	Unknown	Serum	Unknown	
223	Japan	1992	Shigaraki Town Study	Community	40-79	40-79	233	324	233	324	233	324	Unknown	Unknown	Serum	Unknown	
224	Japan	1990-1994	Japan Public Health Center-based prospective Study (JPHC Study), Cohort I	Subnational	40-59	40-59	8,762	14,504	2,856	3,708	2,851	3,702	Enzymatic	Unknown	Serum	Yes (CDC/CRMLN)	
225	Japan	1992	National Nutrition Survey	National	40-79	40-79	2,257	3,126	2,257	3,126	2,257	3,126	Enzymatic	Heparin-Calcium method; Unknown	Serum	Yes (CDC/CRMLN)	
226	Japan	1993	Konan Town Study	Community	40-79	40-79	39	49	39	49	39	49	Unknown	Unknown	Serum	Unknown	
227	Japan	1993	Shigaraki Town Study	Community	40-79	40-79	260	373	260	373	260	373	Unknown	Unknown	Serum	Unknown	
228	Japan	1993	National Nutrition Survey	National	40-79	40-79	2,007	2,890	2,007	2,890	2,007	2,890	Enzymatic	Heparin-Calcium method; Unknown	Serum	Yes (CDC/CRMLN)	
229	Japan	1994	Konan Town Study	Community	40-79	40-79	28	43	28	43	28	43	Unknown	Unknown	Serum	Unknown	
230	Japan	1994	Shigaraki Town Study	Community	40-79	40-79	206	269	206	269	206	269	Unknown	Unknown	Serum	Unknown	
231	Japan	1993-1994	Japan Public Health Center-based prospective Study (JPHC Study), Cohort II	Subnational	40-69	40-69	8,557	16,214	8,549	16,207	8,549	16,206	Enzymatic	Unknown	Serum	Yes (CDC/CRMLN)	
232	Japan	1994	National Nutrition Survey	National	40-59	40-59	1,067	1,675	1,067	1,675	1,067	1,675	Enzymatic	Heparin-Calcium method; Unknown	Serum	Yes (CDC/CRMLN)	
233	Japan	1995	Konan Town Study	Community	40-79	40-79	37	51	37	51	37	51	Unknown	Unknown	Serum	Unknown	
234	Japan	1995	Shigaraki Town Study	Community	40-79	40-79	237	380	237	380	237	380	Unknown	Unknown	Serum	Unknown	
235	Japan	1995	National Nutrition Survey	National	40-59	40-59	985	1,618	985	1,618	985	1,618	Enzymatic	Heparin-Calcium method; Unknown	Serum	Yes (CDC/CRMLN)	
236	Japan	1996	Shigaraki Town Study	Community	40-79	40-79	70	115	70	115	70	115	Unknown	Unknown	Serum	Unknown	
237	Japan	1996	National Nutrition Survey	National	40-79	40-79	1,799	2,731	1,799	2,730	1,799	2,730	Enzymatic	Homogeneous assay (selective inhibition)	Serum	Yes (CDC/CRMLN)	
238	Japan	1997	Shigaraki Town Study	Community	40-79	40-79	45	73	45	73	45	73	Unknown	Unknown	Serum	Unknown	
239	Japan	1997	National Nutrition Survey	National	40-79	40-79	1,845	2,685	1,844	2,685	1,844	2,685	Enzymatic	Homogeneous assay (Accelerator Selective Detergent; Enzymatic)	Serum	Yes (CDC/CRMLN)	
240	Japan	1998	National Nutrition Survey	National	40-79	40-79	1,900	2,740	1,899	2,740	1,899	2,740	Enzymatic	Homogeneous assay (Accelerator Selective Detergent; Enzymatic)	Serum	Yes (CDC/CRMLN)	
241	Japan	1999	National Nutrition Survey	National	40-79	40-79	1,547	2,268	1,546	2,268	1,546	2,268	Enzymatic	Homogeneous assay (Accelerator Selective Detergent; Enzymatic)	Serum	Yes (CDC/CRMLN)	
242	Japan	2000	National Nutrition Survey and National Cardiovascular Survey	National	40-79	40-79	1,714	2,402	1,714	2,402	1,713	2,402	Enzymatic	Homogeneous assay (Accelerator Selective Detergent; Enzymatic)	Serum	Yes (CDC/CRMLN)	
243	Japan	2001	The Japan Association of Health Service Database	Subnational	40-79	40-79	804,504	764,081	804,504	764,081	804,504	764,081	Unknown	Unknown	Serum	Yes (other)	
244	Japan	2001	National Nutrition Survey	National	40-79	40-79	1,647	2,436	1,646	2,436	1,646	2,436	Enzymatic	Homogeneous assay (Accelerator Selective Detergent; Enzymatic)	Serum	Yes (CDC/CRMLN)	

	Country	Data years	Survey/Study name/Citation	Level of representati-veness	Age range as used for the analysis		Sample size (Total cholesterol)		Sample size (HDL cholesterol)		Sample size (Non-HDL cholesterol)		Method used to measure		Whether lipids were measured in serum or plasma samples	Participating to a lipid standardisation programme or quality control schemes	Note
					Male	Female	Male	Female	Male	Female	Male	Female	Total cholesterol	HDL cholesterol (separation and quantification methods)			
245	Japan	2002	National Nutrition Survey	National	40-79	40-79	1,635	2,320	1,634	2,320	1,634	2,320	Enzymatic	Homogeneous assay (Accelerator Selective Detergent; Enzymatic)	Serum	Yes (CDC/CRMLN)	
246	Japan	2002-2003	The Hisayama Study	Community	40-79	40-79	1,312	1,672	1,312	1,672	1,312	1,672	Enzymatic	Unknown	Serum	No	
247	Japan	2003	National Health and Nutrition Survey	National	40-79	40-79	1,567	2,307	1,566	2,306	1,566	2,306	Enzymatic	Homogeneous assay (Accelerator Selective Detergent; Enzymatic)	Serum	Yes (CDC/CRMLN)	
248	Japan	2004	National Health and Nutrition Survey	National	40-79	40-79	1,173	1,736	1,172	1,736	1,172	1,736	Enzymatic	Homogeneous assay (Accelerator Selective Detergent; Enzymatic)	Serum	Yes (CDC/CRMLN)	
249	Japan	2005	National Health and Nutrition Survey	National	40-79	40-79	1,195	1,719	1,195	1,719	1,195	1,719	Enzymatic	Homogeneous assay (Accelerator Selective Detergent; Enzymatic)	Serum	Yes (CDC/CRMLN)	
250	Japan	2006	National Health and Nutrition Survey	National	40-79	40-79	1,316	1,858	1,316	1,858	1,316	1,858	Enzymatic	Homogeneous assay (Accelerator Selective Detergent; Enzymatic)	Serum	Yes (CDC/CRMLN)	
251	Japan	2007	National Health and Nutrition Survey	National	40-79	40-79	1,228	1,730	1,228	1,730	1,228	1,730	Enzymatic	Homogeneous assay (Accelerator Selective Detergent; Enzymatic)	Serum	Yes (CDC/CRMLN)	
252	Japan	2008	National Health and Nutrition Survey	National	40-79	40-79	1,388	1,956	1,387	1,956	1,387	1,956	Enzymatic	Homogeneous assay (Accelerator Selective Detergent; Enzymatic)	Serum	Yes (CDC/CRMLN)	
253	Japan	2009	National Health and Nutrition Survey	National	40-79	40-79	1,328	1,881	1,327	1,881	1,327	1,881	Cholesterol dehydrogenase-ultraviolet method	Homogeneous assay (Accelerator Selective Detergent; Enzymatic)	Serum	Yes (CDC/CRMLN)	
254	Japan	2010	National Health and Nutrition Survey	National	40-79	40-79	1,229	1,681	1,229	1,681	1,229	1,681	Cholesterol dehydrogenase-ultraviolet method	Homogeneous assay (Accelerator Selective Detergent; Enzymatic)	Serum	Yes (CDC/CRMLN)	
255	Japan	2011	National Health and Nutrition Survey	National	40-79	40-79	1,070	1,515	1,070	1,515	1,070	1,515	Cholesterol dehydrogenase-ultraviolet method	Homogeneous assay (Accelerator Selective Detergent; Enzymatic)	Serum	Yes (CDC/CRMLN)	
256	Japan	2011	The Tokyo Health Service Association Database	Community	40-79	40-79	22,650	8,013	40,651	20,574	22,650	8,013	Enzymatic	Homogeneous assay (Accelerator Selective Detergent; Enzymatic)	Serum	Yes (other)	
257	Japan	2012	National Health and Nutrition Survey	National	40-79	40-79	4,373	6,262	4,373	6,262	4,373	6,262	Cholesterol dehydrogenase-ultraviolet method	Homogeneous assay (Accelerator Selective Detergent; Enzymatic)	Serum	Yes (CDC/CRMLN)	
258	Japan	2013	National Health and Nutrition Survey	National	40-79	40-79	1,065	1,455	1,065	1,455	1,065	1,455	Cholesterol dehydrogenase-ultraviolet method	Homogeneous assay (Accelerator Selective Detergent; Enzymatic)	Serum	Yes (CDC/CRMLN)	
259	Japan	2014	National Health and Nutrition Survey	National	40-79	40-79	1,169	1,553	1,169	1,553	1,169	1,553	Cholesterol dehydrogenase-ultraviolet method	Homogeneous assay (Accelerator Selective Detergent; Enzymatic)	Serum	Yes (CDC/CRMLN)	
260	Japan	2015	National Health and Nutrition Survey	National	40-79	40-79	1,030	1,523	1,030	1,523	1,030	1,523	Cholesterol dehydrogenase-ultraviolet method	Homogeneous assay (Accelerator Selective Detergent; Enzymatic)	Serum	Yes (CDC/CRMLN)	

	Country	Data years	Survey/Study name/Citation	Level of representati-veness	Age range as used for the analysis		Sample size (Total cholesterol)		Sample size (HDL cholesterol)		Sample size (Non-HDL cholesterol)		Method used to measure		Whether lipids were measured in serum or plasma samples	Participating to a lipid standardisation programme or quality control schemes	Note
					Male	Female	Male	Female	Male	Female	Male	Female	Total cholesterol	HDL cholesterol (separation and quantification methods)			
261	Japan	2016	National Health and Nutrition Survey	National	40-79	40-79	3,571	5,092	3,571	5,092	3,571	5,092	Cholesterol dehydrogenase-ultraviolet method	Homogeneous assay (Accelerator Selective Detergent; Enzymatic)	Serum	Yes (CDC/CRMLN)	
262	Lithuania	1972-1974	Kaunas Rotterdam Intervention Study (KRIS)	Community	50-59		1,382						Enzymatic		Serum	Yes (other)	
263	Lithuania	1977-1980	Multifactorial Prevention of Ischaemic Heart Disease, Kaunas	Community	40-59		5,633						Enzymatic		Serum	Yes (other)	
264	Lithuania	1983-1985	MONICA, Kaunas	Community	40-59	40-59	504	518	464	448	463	448	Enzymatic	Heparin-Manganese method; Enzymatic	Serum	Yes (WHO-LRC)	
265	Lithuania	1987	Countrywide Integrated Noncommunicable Diseases Intervention Programme survey	Subnational	40-59	40-59	563	587	522	557	521	557	Enzymatic	Phosphotungstate-Magnesium (PTA) method; Enzymatic	Serum	Yes (WHO-LRC)	
266	Lithuania	1986-1987	MONICA, Kaunas	Community	40-59	40-59	620	587	595	564	595	564	Enzymatic	Phosphotungstate-Magnesium (PTA) method; Enzymatic	Serum	Yes (WHO-LRC)	
267	Lithuania	1992-1993	MONICA, Kaunas	Community	40-59	40-59	398	427	383	406	382	405	Enzymatic	Phosphotungstate-Magnesium (PTA) method; Enzymatic	Serum	Yes (WHO-LRC)	
268	Lithuania	1992-1993	Countrywide Integrated Noncommunicable Diseases Intervention Programme survey	Subnational	40-59	40-59	348	487	298	422	297	422	Enzymatic	Phosphotungstate-Magnesium (PTA) method; Enzymatic	Serum	Yes (WHO-LRC)	
269	Lithuania	1998-1999	Countrywide Integrated Noncommunicable Diseases Intervention Programme survey	Subnational	40-59	40-59	446	560	439	543	439	543	Enzymatic	Phosphotungstate-Magnesium (PTA) method; Enzymatic	Serum	Yes (WHO-LRC)	
270	Lithuania	2001-2002	MONICA4	Community	40-59	40-59	436	572	420	530	420	530	Enzymatic	Phosphotungstate-Magnesium (PTA) method; Enzymatic	Serum	Yes (WHO-LRC)	
271	Lithuania	2006-2007	Countrywide Integrated Noncommunicable Diseases Intervention Programme survey	Subnational	40-59	40-59	438	555	438	556	438	555	Enzymatic	Phosphotungstate-Magnesium (PTA) method; Enzymatic	Serum	Yes (other)	
272	Lithuania	2006-2008	MONICA4 Follow-up	Community	50-69	50-69	118	172	118	169	118	169	Enzymatic	Phosphotungstate-Magnesium (PTA) method; Enzymatic	Serum	Yes (WHO-LRC)	
273	New Zealand	1982	MONICA, Auckland	Community	40-59	40-59	737	392	629	363	629	362	Extraction	Phosphotungstate-Magnesium (PTA) method; Extraction	Serum	Yes (WHO-LRC)	
274	New Zealand	1989	The Life in New Zealand Survey	National	40-79	40-79	527	570					Enzymatic		Serum	Yes (CDC/CRMLN)	
275	New Zealand	1993-1994	MONICA, Auckland	Community	40-59	40-59	512	472	509	468	509	468	Enzymatic	Phosphotungstate-Magnesium (PTA) method; Enzymatic	Serum	Yes (WHO-LRC)	
276	New Zealand	1996-1997	National Nutrition Survey	National	40-79	40-79	505	525	504	525	504	525	Enzymatic	Homogeneous assay (alpha-Cyclodextrin + PEG-coupled enzymes)	Serum	Yes (CDC/CRMLN)	
277	New Zealand	2002-2003	Diabetes, Heart and Health Survey	Subnational	40-79	40-79	977	1,112	974	1,112	974	1,112	Enzymatic	Homogeneous assay (alpha-Cyclodextrin + PEG-coupled enzymes)	Serum	Yes (other)	

	Country	Data years	Survey/Study name/Citation	Level of representati-veness	Age range as used for the analysis		Sample size (Total cholesterol)		Sample size (HDL cholesterol)		Sample size (Non-HDL cholesterol)		Method used to measure		Whether lipids were measured in serum or plasma samples	Participating to a lipid standardisation programme or quality control schemes	Note
					Male	Female	Male	Female	Male	Female	Male	Female	Total cholesterol	HDL cholesterol (separation and quantification methods)			
278	New Zealand	2008-2009	2008/09 New Zealand Adult Nutrition Survey	National	40-79	40-79	332	442	332	442	332	442	Enzymatic	Homogeneous assay (Accelerator Selective Detergent; Enzymatic)	Serum	Yes (CDC/CRMLN)	
279	Norway	1979-1980	The Tromsø Study: Tromsø 2	Community	40-49	40-49	1,908	1,854	1,900	1,852	1,900	1,852	Enzymatic	Heparin-Manganese method; Enzymatic	Serum	Unknown	
280	Norway	1986-1987	The Tromsø Study: Tromsø 3	Community	40-59	40-49	4,272	2,564	4,269	2,564	4,266	2,562	Enzymatic	Heparin-Manganese method; Enzymatic	Serum	Yes (WHO-LRC)	
281	Norway	1994-1995	The Tromsø Study: Tromsø 4	Community	40-79	40-79	5,481	5,550	5,467	5,544	5,464	5,542	Enzymatic	Heparin-Manganese method; Enzymatic	Serum	Unknown	
282	Norway	1995-1997	HUNT2 study	Subnational	40-79	40-79	11,821	12,744	11,810	12,742	11,810	12,742	Enzymatic	Phosphotungstate-Magnesium (PTA) method; Enzymatic	Serum	Yes (other)	
283	Norway	2001-2002	The Tromsø Study: Tromsø 5, Tromsø Study Panel	Community	40-79	40-79	441	871	441	870	441	870	Enzymatic	Homogeneous assay (Dextran sulphate-Magnesium + PEG-coupled enzymes)	Serum	Yes (CDC/CRMLN)	
284	Norway	2006-2008	HUNT3 Study	Subnational	40-79	40-79	9,726	11,146	9,726	11,146	9,726	11,146	Enzymatic	Homogeneous assay (Accelerator Selective Detergent; Enzymatic)	Serum	Yes (other)	
285	Norway	2007-2008	The Tromsø Study: Tromsø 6	Community	40-79	40-79	2,785	3,141	2,784	3,139	2,784	3,139	Enzymatic	Homogeneous assay (Dextran sulphate-Magnesium + PEG-coupled enzymes)	Serum	Yes (CDC/CRMLN)	
286	Poland	1983-1984	MONICA, Tarnobrzeg Voivodship	Community	40-59	40-59	835	975	835	975	835	975	Direct	Heparin-Manganese method; Direct	Plasma	Yes (WHO-LRC)	
287	Poland	1983-1985	MONICA, Warsaw	Community	40-59	40-59	891	901	887	897	886	897	Direct	Heparin-Manganese method; Direct	Plasma	Yes (WHO-LRC)	
288	Poland	1987-1988	MONICA, Tarnobrzeg Voivodship	Community	40-59	40-59	419	440	419	440	419	440	Direct	Heparin-Manganese method; Direct	Plasma	Yes (WHO-LRC)	
289	Poland	1988-1989	MONICA, Warsaw	Community	40-59	40-59	475	492	467	490	467	490	Direct	Heparin-Manganese method; Direct	Plasma	Yes (WHO-LRC)	
290	Poland	1989-1990	Polish Program CINDI (CINDI Lodz 1989-1990)	Community	40-59	40-59	393	468					Enzymatic		Serum	Yes (other)	
291	Poland	1992-1993	MONICA, Tarnobrzeg Voivodship	Community	40-59	40-59	414	469	414	469	414	469	Direct	Heparin-Manganese method; Enzymatic	Plasma	Yes (WHO-LRC)	
292	Poland	1993	MONICA, Warsaw	Community	40-59	40-59	522	548	518	547	518	547	Enzymatic	Heparin-Manganese method; Enzymatic	Plasma	Yes (WHO-LRC)	
293	Poland	1995-1996	Polish Program CINDI (CINDI Lodz 1995)	Community	40-59	40-59	365	541	365	540	365	540	Enzymatic	Homogeneous assay	Serum	Yes (other)	
294	Poland	1997	NATPOL	National	40-79	40-79	241	214					Accutrend		Capillary	Unknown	
295	Poland	2000	The health status, risk factors of chronic diseases and health behaviors of residents of Torun (CINDI Torun 2000)	Community	40-79	40-79	452	497	451	497	451	497	Enzymatic	Homogeneous assay	Serum	Yes (other)	
296	Poland	2001-2002	The health status, risk factors of chronic diseases and health behaviors of residents of Lodz (CINDI Lodz 2001)	Community	40-59	40-59	485	354	485	353	485	353	Enzymatic	Homogeneous assay	Serum	Yes (other)	

	Country	Data years	Survey/Study name/Citation	Level of representativeness	Age range as used for the analysis		Sample size (Total cholesterol)		Sample size (HDL cholesterol)		Sample size (Non-HDL cholesterol)		Method used to measure		Whether lipids were measured in serum or plasma samples	Participating to a lipid standardisation programme or quality control schemes	Note
					Male	Female	Male	Female	Male	Female	Male	Female	Total cholesterol	HDL cholesterol (separation and quantification methods)			
297	Poland	2002	NATPOL	National	40-79	40-79	390	490	390	490	390	490	Enzymatic	Homogeneous assay (Dextran sulphate-Magnesium + PEG-coupled enzymes)	Serum	Yes (other)	
298	Poland	2003	The European Male Ageing Study	Community	40-79		196		196		196		Unknown	Unknown	Unknown	Unknown	
299	Poland	2004	LIPIDOGRAM2004 Study - National epidemiological study of lipid disorders and selected risk factors of cardiovascular disease in primary health care in Poland	National	40-79	40-79	4,138	5,883	4,138	5,883	4,138	5,883	Enzymatic	Homogeneous assay (Accelerator Selective Detergent; Enzymatic)	Serum	Yes (CDC/CRMLN)	
300	Poland	2003-2005	National Multicenter Health Survey in Poland. Project WOBASZ	National	40-69	40-69	2,741	2,992	2,739	2,990	2,739	2,990	Enzymatic	Heparin-Manganese method; Enzymatic	Serum	Yes (WHO-LRC)	
301	Poland	2006	The health, risk factors for chronic diseases, attitudes and behaviors of health residents of Torun (CINDI Torun 2006)	Community	40-59	40-59	376	588	376	588	376	588	Enzymatic	Homogeneous assay	Serum	Yes (other)	
302	Poland	2006	LIPIDOGRAM2006 Study - National epidemiological study of lipid disorders and selected risk factors of cardiovascular disease in primary health care in Poland	National	40-79	40-79	3,688	5,881	3,688	5,880	3,688	5,880	Enzymatic	Homogeneous assay (Accelerator Selective Detergent; Enzymatic)	Serum	Yes (CDC/CRMLN)	
303	Poland	2008	The European Male Ageing Study	Community	40-79		112		112		112		Unknown	Unknown	Unknown	Unknown	
304	Poland	2003-2013	Mogielica Human Ecology Study	Community	50-79	50-79	11	65	11	65	11	65	Enzymatic	Homogeneous assay (Accelerator Selective Detergent; Enzymatic)	Serum	Yes (other)	
305	Poland	2011	NATPOL	National	40-79	40-79	440	395	439	395	439	395	Enzymatic	Homogeneous assay (Accelerator Selective Detergent; Enzymatic)	Serum	Yes (other)	
306	Poland	2013-2014	National Multicenter Health Survey in Poland. Project WOBASZ II	National	40-79	40-79	1,019	1,298	1,014	1,297	1,014	1,297	Enzymatic	Homogeneous assay (Dextran sulphate-Magnesium + PEG-coupled enzymes)	Serum	Yes (CDC/CRMLN)	
307	Poland	2015-2016	LIPIDOGRAM2015 & LIPIDOGEN2015 Study - National epidemiological study of lipid disorders and selected risk factors of cardiovascular disease in primary health care in Poland	National	40-79	40-79	2,099	3,734	2,100	3,733	2,098	3,733	Enzymatic	Homogeneous assay (Immuno-enzymatic)	Serum	Yes (CDC/CRMLN)	
308	Slovakia	1993	Countrywide Integrated Noncommunicable Diseases Intervention Programme	National	40-59	40-59	331	564	314	538	313	538	Enzymatic	Homogeneous assay (Accelerator Selective Detergent; Enzymatic)	Serum	Yes (WHO-LRC)	
309	Slovakia	1998	Countrywide Integrated Noncommunicable Diseases Intervention Programme	National	40-59	40-59	415	476	406	475	405	474	Enzymatic	Homogeneous assay (Accelerator Selective Detergent; Enzymatic)	Serum	Yes (WHO-LRC)	
310	Slovakia	2003	Countrywide Integrated Noncommunicable Diseases Intervention Programme	National	40-59	40-59	302	467	301	465	301	465	Enzymatic	Homogeneous assay (Accelerator Selective Detergent; Enzymatic)	Serum	Yes (WHO-LRC)	

	Country	Data years	Survey/Study name/Citation	Level of representati-veness	Age range as used for the analysis		Sample size (Total cholesterol)		Sample size (HDL cholesterol)		Sample size (Non-HDL cholesterol)		Method used to measure		Whether lipids were measured in serum or plasma samples	Participating to a lipid standardisation programme or quality control schemes	Note
					Male	Female	Male	Female	Male	Female	Male	Female	Total cholesterol	HDL cholesterol (separation and quantification methods)			
311	Slovakia	2008	Countrywide Integrated Noncommunicable Diseases Intervention Programme	National	40-59	40-59	195	286	195	286	195	286	Enzymatic	Homogeneous assay (Accelerator Selective Detergent; Enzymatic)	Serum	Yes (WHO-LRC)	
312	Slovakia	2011-2012	European Health Examination Survey	National	40-59	40-59	395	507	395	506	395	506	Enzymatic	Homogeneous assay (Accelerator Selective Detergent; Enzymatic)	Serum	Yes (CDC/CRMLN)	
313	South Korea	1998	Korea National Health and Nutrition Examination Survey	National	40-79	40-79	1,737	2,135	1,737	2,135	1,737	2,135	Enzymatic	Homogeneous assay (Accelerator Selective Detergent; Enzymatic)	Serum	No	
314	South Korea	2001	Korea National Health and Nutrition Examination Survey	National	40-79	40-79	1,267	1,619	1,271	1,625	1,263	1,608	Enzymatic	Homogeneous assay (Accelerator Selective Detergent; Enzymatic)	Serum	No	
315	South Korea	2002-2003	Korean National Health Insurance	National	40-79	40-79	2,970,160	2,469,861					Enzymatic		Serum	Yes (other)	
316	South Korea	2005	Korea National Health and Nutrition Examination Survey	National	40-79	40-79	1,472	1,896	1,470	1,895	1,470	1,895	Enzymatic	Homogeneous assay	Serum	No	
317	South Korea	2004-2005	Korean National Health Insurance	National	40-79	40-79	3,572,663	3,239,712					Enzymatic		Serum	Yes (other)	
318	South Korea	2007	Korea National Health and Nutrition Examination Survey	National	40-79	40-79	757	1,014	757	1,014	757	1,014	Enzymatic	Homogeneous assay	Serum	No	
319	South Korea	2006-2007	Korean National Health Insurance	National	40-79	40-79	4,294,406	4,318,878					Enzymatic		Serum	Yes (other)	
320	South Korea	2008	Korea National Health and Nutrition Examination Survey	National	40-79	40-79	1,716	2,336	1,715	2,336	1,715	2,336	Enzymatic	Homogeneous assay (Accelerator Selective Detergent; Enzymatic)	Serum	Yes (CDC/CRMLN)	
321	South Korea	2009	Korea National Health and Nutrition Examination Survey	National	40-79	40-79	1,959	2,557	1,958	2,557	1,958	2,557	Enzymatic	Homogeneous assay (Accelerator Selective Detergent; Enzymatic)	Serum	Yes (CDC/CRMLN)	
322	South Korea	2008-2009	Korean National Health Insurance	National	40-79	40-79	5,440,602	5,674,367					Enzymatic		Serum	Yes (other)	
323	South Korea	2010	Korea National Health and Nutrition Examination Survey	National	40-79	40-79	1,698	2,117	1,698	2,117	1,698	2,117	Enzymatic	Homogeneous assay (Accelerator Selective Detergent; Enzymatic)	Serum	Yes (CDC/CRMLN)	
324	South Korea	2011	Korea National Health and Nutrition Examination Survey	National	40-79	40-79	1,719	2,203	1,719	2,203	1,719	2,203	Enzymatic	Homogeneous assay (Accelerator Selective Detergent; Enzymatic)	Serum	Yes (CDC/CRMLN)	
325	South Korea	2010-2011	Korean National Health Insurance	National	40-79	40-79	6,582,856	6,990,887					Enzymatic	Unknown (multiple separation techniques used); Enzymatic	Serum	Yes (other)	3
326	South Korea	2012	Korea National Health and Nutrition Examination Survey	National	40-79	40-79	1,635	2,141	1,634	2,140	1,634	2,140	Enzymatic	Homogeneous assay (Accelerator Selective Detergent; Enzymatic)	Serum	Yes (CDC/CRMLN)	
327	South Korea	2013	Korea National Health and Nutrition Examination Survey	National	40-79	40-79	1,515	1,929	1,515	1,929	1,515	1,929	Enzymatic	Homogeneous assay (Accelerator Selective Detergent; Enzymatic)	Serum	Yes (CDC/CRMLN)	
328	South Korea	2012-2013	Korean National Health Insurance	National	40-79	40-79	7,138,521	7,601,045					Enzymatic	Unknown (multiple separation techniques used); Enzymatic	Serum	Yes (other)	3
329	South Korea	2014	Korea National Health and Nutrition Examination Survey	National	40-79	40-79	1,452	1,933	1,452	1,933	1,452	1,933	Enzymatic	Homogeneous assay (Accelerator Selective Detergent; Enzymatic)	Serum	Yes (CDC/CRMLN)	

	Country	Data years	Survey/Study name/Citation	Level of representativeness	Age range as used for the analysis		Sample size (Total cholesterol)		Sample size (HDL cholesterol)		Sample size (Non-HDL cholesterol)		Method used to measure		Whether lipids were measured in serum or plasma samples	Participating to a lipid standardisation programme or quality control schemes	Note
					Male	Female	Male	Female	Male	Female	Male	Female	Total cholesterol	HDL cholesterol (separation and quantification methods)			
330	South Korea	2015	Korea National Health and Nutrition Examination Survey	National	40-79	40-79	1,575	2,011	1,575	2,011	1,575	2,011	Enzymatic	Homogeneous assay (Accelerator Selective Detergent; Enzymatic)	Serum	Yes (CDC/CRMLN)	
331	South Korea	2014-2015	Korean National Health Insurance	National	40-79	40-79	7,707,179	8,108,953					Enzymatic	Unknown (multiple separation techniques used); Enzymatic	Serum	Yes (other)	3
332	South Korea	2016	Korea National Health and Nutrition Examination Survey	National	40-79	40-79	1,674	2,132	1,673	2,132	1,673	2,132	Enzymatic	Homogeneous assay (Accelerator Selective Detergent; Enzymatic)	Serum	Yes (CDC/CRMLN)	
333	Spain	1986-1988	MONICA, Catalonia	Community	40-59	40-59	645	662	645	663	645	662	Enzymatic	PEG 6000 method; Enzymatic	Serum	Yes (WHO-LRC)	
334	Spain	1989	Cardiovascular Risk Factors Study in Catalonia	Subnational	40-79	40-79	99	90	99	90	99	90	Enzymatic	Phosphotungstate-Magnesium (PTA) method; Enzymatic	Serum	Yes (CDC/CRMLN)	
335	Spain	1990	Hernandez Lanchas et al., An Med Interna 1992; 9: 64-71	Community	40-79	40-49	103	17					Unknown		Unknown	Unknown	
336	Spain	1990	Hernandez Lanchas et al., An Med Interna 1992; 9: 64-71	Community	40-79	40-69	95	159					Unknown		Unknown	Unknown	
337	Spain	1990-1992	MONICA, Catalonia	Community	40-59	40-59	876	491	876	491	876	491	Enzymatic	Phosphotungstate-Magnesium (PTA) method; Enzymatic	Serum	Yes (WHO-LRC)	
338	Spain	1991-1993	Encuesta de Factores de Riesgo Cardiovascular en la Región de Murcia (Cardiovascular Risk Factors Survey)	Subnational	40-69	40-69	568	639	540	609	540	609	Enzymatic	Phosphotungstate-Magnesium (PTA) method; Enzymatic	Serum	Unknown	
339	Spain	1994-1996	MONICA, Catalonia	Community	40-59	40-59	888	775	888	775	888	775	Enzymatic	Updated phosphotungstate-Magnesium method after the Boehringer-Ms (PTA 543004); Enzymatic	Serum	Yes (WHO-LRC)	
340	Spain	1999-2000	Factores de riesgo en las islas Baleares: Estudio CORSAIB	Subnational	40-69	40-69	616	693	615	690	615	690	Enzymatic	Homogeneous assay (Catalase)	Unknown	Unknown	
341	Spain	2000-2001	EUREYE Study	Subnational	70-79	70-79	141	149					Enzymatic		Plasma	Yes (other)	
342	Spain	2001-2002	Catalan Health Interview Survey	Subnational	40-69	40-69	339	379	344	380	339	379	Enzymatic	Unknown; Enzymatic	Serum	Unknown	
343	Spain	2001-2003	DIabetes, Nutrición y Obesidad en la población adulta de la Región de Murcia (DINO)	Subnational	40-79	40-79	403	454	403	454	403	454	Enzymatic	Homogeneous assay (Catalase)	Serum	Yes (other)	
344	Spain	2000-2005	CDC of the Canary Islands	Subnational	40-69	40-69	1,629	2,130	1,628	2,130	1,628	2,130	Enzymatic	Dextran sulphate-Magnesium method; Enzymatic	Serum	Yes (other)	
345	Spain	2003	The European Male Ageing Study	Community	40-79		406		402		402		Unknown	Unknown	Unknown	Unknown	
346	Spain	2004	Vioque et al., Obesity 2008; 16: 664-670	Community	40-79	40-79	45	58	45	58	45	58	Enzymatic	Phosphotungstate-Magnesium (PTA) method; Enzymatic	Serum	Unknown	
347	Spain	2004	Cardiovascular Risk Study in Castilla y León (RECCyL)	Subnational	40-79	40-79	1,175	1,215	1,165	1,194	1,165	1,194	Enzymatic	Homogeneous assay (alpha-Cyclodextrin + PEG-coupled enzymes)	Serum	Unknown	
348	Spain	2003-2005	Registre Gironi del Cor (REGICOR)	Subnational	40-79	40-79	2,686	2,952	2,687	2,952	2,686	2,952	Enzymatic	Phosphotungstate-Magnesium (PTA) method; Enzymatic	Serum	Yes (WHO-LRC)	

	Country	Data years	Survey/Study name/Citation	Level of representati-veness	Age range as used for the analysis		Sample size (Total cholesterol)		Sample size (HDL cholesterol)		Sample size (Non-HDL cholesterol)		Method used to measure		Whether lipids were measured in serum or plasma samples	Participating to a lipid standardisation programme or quality control schemes	Note
					Male	Female	Male	Female	Male	Female	Male	Female	Total cholesterol	HDL cholesterol (separation and quantification methods)			
349	Spain	2004-2006	PREVICTUS	National	60-79	60-79	2,936	3,256	2,648	2,933	2,645	2,931	Unknown (multiple lab)	Unknown	Unknown	Unknown	
350	Spain	2008	The European Male Ageing Study	Community	40-79		243		240		240		Unknown	Unknown	Unknown	Unknown	
351	Spain	2007-2009	Harmonizing Equation of Risk in Mediterraneon countries EXtremadura	Subnational	40-79	40-79	969	1,118	968	1,116	968	1,116	Enzymatic	Homogeneous assay (Dextran sulphate-Magnesium + PEG-coupled enzymes)	Serum	Yes (other)	
352	Spain	2008-2010	Study on Nutrition and Cardiovascular Risk in Spain	National	40-79	40-79	3,894	4,282	3,894	4,282	3,894	4,282	Enzymatic	Homogeneous assay (Catalase)	Serum	Yes (other)	
353	Spain	2009	Cardiovascular Risk Study in Castilla y León (RECCyL)	Subnational	40-79	40-79	877	1,026	862	1,014	862	1,014	Enzymatic	Homogeneous assay (alpha-Cyclodextrin + PEG-coupled enzymes)	Serum	Unknown	
354	Spain	2014	Cardiovascular Risk Study in Castilla y León (RECCyL)	Subnational	40-79	40-79	857	1,005	830	975	829	974	Enzymatic	Homogeneous assay (alpha-Cyclodextrin + PEG-coupled enzymes)	Serum	Unknown	
355	Spain	2015	Study on Nutrition and Cardiovascular Risk in Spain (ENRICA)	National	70-79	70-79	353	454	353	454	353	454	Enzymatic	Homogeneous assay (Catalase)	Serum	Yes (other)	
356	Sweden	1985	MONICA Gothenburg	Community	40-59	40-59	336	377					Enzymatic		Serum	Yes (WHO-LRC)	
357	Sweden	1986	MONICA Northern Sweden	Subnational	40-59	40-59	417	426					Enzymatic		Serum	Yes (WHO-LRC)	
358	Sweden	1990	MONICA Northern Sweden	Subnational	40-59	40-59	397	407					Enzymatic		Serum	Yes (WHO-LRC)	
359	Sweden	1990	MONICA Gothenburg	Community	40-59	40-59	391	429					Enzymatic		Serum	Yes (WHO-LRC)	
360	Sweden	1992-1994	Malmö Diet and Cancer	Community	50-59	50-59	1,052	1,483					Unknown		Unknown	Unknown	
361	Sweden	1994	MONICA Northern Sweden	Subnational	40-69	40-69	393	404					Enzymatic		Serum	Yes (WHO-LRC)	
362	Sweden	1995	MONICA Gothenburg	Community	40-59	40-59	390	469					Enzymatic		Serum	Yes (WHO-LRC)	
363	Sweden	1999	MONICA Northern Sweden	Subnational	40-69	40-69	341	406					Enzymatic		Serum	Yes (other)	
364	Sweden	2003	The European Male Ageing Study	Community	40-79		210						Unknown		Unknown	Unknown	
365	Sweden	2001-2004	Swedish INTERGENE Cohort Study	Subnational	40-69	40-69	716	748					Enzymatic		Serum	No	
366	Sweden	2004	MONICA Northern Sweden	Subnational	40-69	40-69	372	410					Enzymatic		Serum	Yes (other)	
367	Sweden	2008	The European Male Ageing Study	Community	40-79		135						Unknown		Unknown	Unknown	
368	Sweden	2009	MONICA Northern Sweden	Subnational	40-69	40-69	358	360					Enzymatic		Serum	Yes (other)	
369	Sweden	2014	MONICA Northern Sweden	Subnational	40-69	40-69	305	343					Enzymatic		Serum	Yes (other)	
370	Switzerland	1984-1986	The Swiss MONICA Study Wave I	Subnational	40-69	40-69	930	827	929	827	929	827	Enzymatic	Phosphotungstate-Magnesium (PTA) method; Enzymatic	Serum/ plasma	Yes (WHO-LRC)	
371	Switzerland	1988-1989	The Swiss MONICA Study Wave II	Subnational	40-69	40-69	953	874	953	874	953	874	Enzymatic	Phosphotungstate-Magnesium (PTA) method; Enzymatic	Serum	Yes (WHO-LRC)	
372	Switzerland	1992-1993	The Swiss MONICA Study Wave III	Subnational	40-69	40-69	871	915	871	915	871	915	Enzymatic	Phosphotungstate-Magnesium (PTA) method; Enzymatic	Serum	Yes (WHO-LRC)	
373	Switzerland	2003-2006	Cohorte Lausannoise (CoLaus)	Community	40-69	40-69	1,840	2,007	1,840	2,006	1,840	2,006	Enzymatic	Homogeneous assay (alpha-Cyclodextrin + PEG-coupled enzymes)	Plasma	No	
374	Switzerland	2007-2012	Bus Santé Study	Subnational	40-79	40-79	1,020	1,009	1,020	1,009	1,020	1,009	Enzymatic	Homogeneous assay (Dextran sulphate-Magnesium + PEG-coupled enzymes)	Plasma	No	

	Country	Data years	Survey/Study name/Citation	Level of representati-veness	Age range as used for the analysis		Sample size (Total cholesterol)		Sample size (HDL cholesterol)		Sample size (Non-HDL cholesterol)		Method used to measure		Whether lipids were measured in serum or plasma samples	Participating to a lipid standardisation programme or quality control schemes	Note
					Male	Female	Male	Female	Male	Female	Male	Female	Total cholesterol	HDL cholesterol (separation and quantification methods)			
375	Switzerland	2009-2012	Cohorte Lausannoise (CoLaus)	Community	40-79	40-79	1,444	1,517	1,444	1,517	1,444	1,517	Enzymatic	Homogeneous assay (alpha-Cyclodextrin + PEG-coupled enzymes)	Plasma	No	
376	Switzerland	2014-2017	Cohorte Lausannoise (CoLaus)	Community	50-79	50-79	722	762	722	761	722	761	Enzymatic	Homogeneous assay (alpha-Cyclodextrin + PEG-coupled enzymes)	Plasma	No	
377	Thailand	1991	Thailand National Health Examination Survey I	National	40-79	40-79	2,508	3,165					Unknown		Serum	Unknown	
378	Thailand	1997	Thailand National Health Examination Survey II	National	40-59	40-59	447	689					Unknown		Unknown	Unknown	
379	Thailand	2000	InterASIA	National	40-79	40-79	1,739	2,619					Enzymatic		Serum	Yes (CDC/CRMLN)	
380	Thailand	2004	Thailand National Health Examination Survey III	National	40-79	40-79	13,645	15,122					Enzymatic		Serum	Unknown	
381	Thailand	2003-2004	The Fifth National Nutrition Survey of Thailand	National	40-79	40-79	300	386					Enzymatic		Plasma	No	
382	Thailand	2009	Thailand National Health Examination Survey IV	National	40-79	40-79	6,723	7,424					Enzymatic		Serum	Unknown	
383	United Kingdom	1983-1984	MONICA, Belfast	Subnational	40-59	40-59	605	610	594	598	592	598	Enzymatic	Phosphotungstate-Magnesium (PTA) method; Enzymatic	Serum	Yes (WHO-LRC)	
384	United Kingdom	1984-1986	Scottish Heart Health Survey	Subnational	40-59	40-59	4,068	3,926	3,870	3,777	3,867	3,774	Enzymatic	Dextran sulphate-Magnesium method; Unknown	Serum	Yes (other)	
385	United Kingdom	1986-1987	Dietary and Nutritional Survey of British Adults 1986-1987	National	40-59	40-59	385	380	384	380	384	380	Unknown	Unknown	Serum	Unknown	
386	United Kingdom	1986-1987	MONICA, Belfast	Subnational	40-59	40-59	617	611	617	615	614	610	Enzymatic	Phosphotungstate-Magnesium (PTA) method; Enzymatic	Serum	Yes (WHO-LRC)	
387	United Kingdom	1987-1988	Edinburgh Artery Study	Community	60-69	60-69	429	396	427	396	427	396	Unknown	Precipitation; Unknown	Serum	Yes (WHO-LRC)	
388	United Kingdom	1991-1992	Health Survey for England	National	40-79	40-79	1,389	1,483					Unknown		Serum	Unknown	
389	United Kingdom	1991-1992	MONICA, Belfast	Subnational	40-59	40-59	545	508	542	508	542	508	Enzymatic	Phosphotungstate-Magnesium (PTA) method; Enzymatic	Serum	Yes (WHO-LRC)	
390	United Kingdom	1992	MONICA, Glasgow	Community	40-59	40-59	356	358	332	342	332	342	Enzymatic	Dextran sulphate-Magnesium method/Phosphotungstate-Magnesiume (PTA) method; Enzymatic	Serum	Yes (WHO-LRC)	
391	United Kingdom	1993	Health Survey for England	National	40-79	40-79	3,208	3,453					Unknown		Serum	Unknown	
392	United Kingdom	1994	Health Survey for England	National	40-79	40-79	2,872	3,269					Enzymatic		Serum	Yes (other)	
393	United Kingdom	1995	MONICA, Glasgow	Community	40-59	40-59	431	429	412	417	412	417	Enzymatic	Dextran sulphate-Magnesium method; Enzymatic	Serum	Yes (WHO-LRC)	
394	United Kingdom	1994-1995	National Diet and Nutrition Survey (NDNS)	National	70-79	70-79	280	195	280	196	280	195	Unknown	Unknown	Plasma	Unknown	
395	United Kingdom	1995	Scottish Health Survey (SHeS)	Subnational	40-59	40-59	1,174	1,352					Enzymatic		Serum	Yes (other)	

	Country	Data years	Survey/Study name/Citation	Level of representati-veness	Age range as used for the analysis		Sample size (Total cholesterol)		Sample size (HDL cholesterol)		Sample size (Non-HDL cholesterol)		Method used to measure		Whether lipids were measured in serum or plasma samples	Participating to a lipid standardisation programme or quality control schemes	Note
					Male	Female	Male	Female	Male	Female	Male	Female	Total cholesterol	HDL cholesterol (separation and quantification methods)			
396	United Kingdom	1998	Health Survey for England	National	40-79	40-79	2,969	3,326	2,962	3,317	2,959	3,315	Enzymatic	Phosphotungstate-Magnesium (PTA) method; Enzymatic	Serum	Yes (other)	
397	United Kingdom	1998	Scottish Health Survey (SHeS)	Subnational	40-69	40-69	1,523	1,811	1,511	1,806	1,511	1,805	Enzymatic	Phosphotungstate-Magnesium (PTA) method; Enzymatic	Serum	Yes (other)	
398	United Kingdom	1998-2000	The British Regional Heart Study	National	60-79		3,938		3,912		3,912		Enzymatic	Homogeneous assay (alpha-Cyclodextrin + PEG-coupled enzymes)	Serum	Unknown	
399	United Kingdom	1999-2001	British Women's Heart and Health Study	National		60-79		3,697		3,691		3,691	Unknown	Precipitation; Unknown	Serum	Unknown	5
400	United Kingdom	2000	Health Survey for England	National	70-79	70-79	73	78	68	72	68	72	Unknown	Unknown	Unknown	Unknown	
401	United Kingdom	1999-2004	Hertfordshire Cohort Study	Subnational	60-69	60-69	1,324	1,161	1,324	1,161	1,324	1,161	Enzymatic	Unknown	Serum	Unknown	
402	United Kingdom	2000-2001	National Diet and Nutrition Survey 2000-2001	National	40-59	40-59	273	306	272	306	272	306	Enzymatic	Phosphotungstate-Magnesium (PTA) method; Enzymatic	Plasma	Yes (other)	
403	United Kingdom	2003	The European Male Ageing Study	Community	40-79		396		396		396		Unknown	Unknown	Unknown	Unknown	
404	United Kingdom	2003	Health Survey for England	National	40-79	40-79	2,439	2,837	2,440	2,837	2,439	2,837	Enzymatic	Homogeneous assay (Antibody, two reagents)	Serum	Yes (other)	
405	United Kingdom	2003	Scottish Health Survey (SHeS)	Subnational	40-79	40-79	1,290	1,502	1,291	1,502	1,290	1,502	Enzymatic	Homogeneous assay	Serum	Yes (other)	
406	United Kingdom	2004-2005	English Longitudinal Study of Ageing Wave 2 2004-2005	National	60-79	60-79	1,594	1,819	1,592	1,819	1,592	1,819	Enzymatic	Homogeneous assay (Antibody, two reagents)	Serum	Yes (other)	
407	United Kingdom	2005	Health Survey for England	National	70-79	70-79	487	563	487	563	487	563	Enzymatic	Homogeneous assay (Antibody, two reagents)	Serum	Yes (other)	
408	United Kingdom	2006	Health Survey for England	National	40-79	40-79	2,274	2,666	2,275	2,666	2,274	2,666	Enzymatic	Homogeneous assay (Antibody, two reagents)	Serum	Yes (other)	
409	United Kingdom	2008	The European Male Ageing Study	Community	40-79		284		280		280		Unknown	Unknown	Unknown	Unknown	
410	United Kingdom	2008	Health Survey for England	National	40-79	40-79	2,241	2,621	2,241	2,620	2,241	2,620	Enzymatic	Homogeneous assay (Antibody, two reagents)	Serum	Yes (other)	
411	United Kingdom	2008	Scottish Health Survey (SHeS)	Subnational	40-79	40-79	289	321	289	321	289	321	Enzymatic	Homogeneous assay	Serum	Yes (other)	
412	United Kingdom	2008-2009	English Longitudinal Study of Ageing Wave 4 2008-2009	National	50-79	50-79	2,657	3,173	2,655	3,170	2,655	3,170	Enzymatic	Homogeneous assay (Antibody, two reagents)	Serum	Yes (other)	
413	United Kingdom	2009	Health Survey for England	National	40-79	40-79	736	813	736	813	736	813	Enzymatic	Homogeneous assay (Antibody, two reagents)	Serum	Yes (other)	
414	United Kingdom	2009	Scottish Health Survey (SHeS)	Subnational	40-79	40-79	255	317	255	317	255	317	Enzymatic	Homogeneous assay	Serum	Yes (other)	
415	United Kingdom	2010	Health Survey for England	National	40-79	40-79	1,135	1,447	1,135	1,447	1,135	1,447	Enzymatic	Homogeneous assay	Serum	Yes (other)	
416	United Kingdom	2008-2012	National Diet and Nutrition Survey (NDNS)	National	40-79	40-79	294	390	294	390	294	390	Enzymatic	Homogeneous assay (Accelerator Selective Detergent; Enzymatic)	Serum	Yes (other)	

	Country	Data years	Survey/Study name/Citation	Level of representati-veness	Age range as used for the analysis		Sample size (Total cholesterol)		Sample size (HDL cholesterol)		Sample size (Non-HDL cholesterol)		Method used to measure		Whether lipids were measured in serum or plasma samples	Participating to a lipid standardisation programme or quality control schemes	Note
					Male	Female	Male	Female	Male	Female	Male	Female	Total cholesterol	HDL cholesterol (separation and quantification methods)			
417	United Kingdom	2010	Scottish Health Survey (SHeS)	Subnational	40-79	40-79	239	304	239	304	239	304	Enzymatic	Homogeneous assay	Serum	Yes (other)	
418	United Kingdom	2011	Health Survey for England	National	40-79	40-79	1,178	1,458	1,177	1,458	1,176	1,458	Enzymatic	Homogeneous assay	Serum	Yes (other)	
419	United Kingdom	2011	Scottish Health Survey (SHeS)	Subnational	40-79	40-79	230	258	230	258	230	258	Enzymatic	Homogeneous assay	Serum	Yes (other)	
420	United Kingdom	2012	Health Survey for England	National	40-79	40-79	1,236	1,492	1,233	1,492	1,233	1,492	Enzymatic	Homogeneous assay	Serum	Yes (other)	
421	United Kingdom	2012-2013	English Longitudinal Study of Ageing Wave 6 2012-2013	National	50-79	50-79	2,496	3,030	2,494	3,029	2,494	3,029	Enzymatic	Homogeneous assay	Serum	Yes (other)	
422	United Kingdom	2013	Health Survey for England	National	40-79	40-79	1,426	1,627	1,424	1,626	1,424	1,626	Enzymatic	Homogeneous assay	Serum	Yes (other)	
423	United Kingdom	2014	Health Survey for England	National	40-79	40-79	1,228	1,423	1,229	1,423	1,228	1,423	Enzymatic	Homogeneous assay	Serum	Yes (other)	
424	United Kingdom	2013-2014	National Diet and Nutrition Survey (NDNS)	National	40-79	40-79	199	286	199	286	199	286	Enzymatic	Homogeneous assay (Accelerator Selective Detergent; Enzymatic)	Serum	Yes (other)	
425	United Kingdom	2015	Health Survey for England	National	40-79	40-79	1,217	1,477	1,217	1,478	1,217	1,477	Enzymatic	Homogeneous assay	Serum	Yes (other)	
426	United Kingdom	2016	Health Survey for England	National	40-79	40-79	1,183	1,441	1,183	1,441	1,183	1,441	Enzymatic	Homogeneous assay	Serum	Yes (other)	
427	United States of America	1971-1975	US NHANES I	National	40-69	40-69	3,435	4,303					Unknown		Serum	Yes (CDC/CRMLN)	
428	United States of America	1976-1980	US NHANES II	National	40-69	40-69	2,965	3,288	2,374	2,703	2,374	2,703	LRC method	Heparin-Manganese method; LRC method	Serum	Yes (CDC/CRMLN)	
429	United States of America	1979-1980	MONICA, Stanford	Subnational	40-59	40-59	277	345	276	344	276	344	Extraction	Heparin-Manganese method; Direct	Plasma	Yes (WHO-LRC)	6
430	United States of America	1985-1986	MONICA, Stanford	Subnational	40-59	40-59	267	314	267	314	267	314	Extraction	Heparin-Manganese method; Extraction	Plasma	Yes (WHO-LRC)	6
431	United States of America	1987-1989	Atherosclerosis Risk in Communities Study	Subnational	50-59	50-59	2,460	3,006	2,458	3,006	2,458	3,006	Enzymatic	Dextran sulphate-Magnesium method; Enzymatic	Plasma	Yes (CDC/CRMLN)	
432	United States of America	1989-1990	Cardiovascular Health Study	Subnational	70-79	70-79	1,195	1,511	1,193	1,510	1,192	1,510	Enzymatic	Dextran sulphate-Magnesium method; Enzymatic	Plasma	Yes (other)	
433	United States of America	1989-1990	MONICA, Stanford	Subnational	40-59	40-59	263	326	263	325	263	325	Enzymatic	Dextran sulphate-Magnesium method; Direct/Enzymatic	Plasma	Yes (WHO-LRC)	6
434	United States of America	1990-1992	Atherosclerosis Risk in Communities Study	Subnational	50-69	50-69	4,018	4,897	4,000	4,868	3,999	4,868	Enzymatic	Dextran sulphate-Magnesium method; Enzymatic	Plasma	Yes (CDC/CRMLN)	
435	United States of America	1988-1994	US NHANES III	National	40-79	40-79	3,946	4,301	3,905	4,261	3,903	4,260	Enzymatic	Heparin-Manganese method; Enzymatic	Serum	Yes (CDC/CRMLN)	
436	United States of America	1992-1993	Cardiovascular Health Study	Subnational	70-79	70-79	1,226	1,695	1,222	1,694	1,222	1,694	Enzymatic	Dextran sulphate-Magnesium method; Enzymatic	Plasma	Yes (other)	
437	United States of America	1993-1995	Atherosclerosis Risk in Communities Study	Subnational	50-69	50-69	3,804	4,824	3,800	4,821	3,800	4,821	Enzymatic	Dextran sulphate-Magnesium method; Enzymatic	Plasma	Yes (CDC/CRMLN)	
438	United States of America	1993-1994	Cardiovascular Health Study	Subnational	70-79	70-79	1,243	1,776					Enzymatic		Plasma	Yes (other)	
439	United States of America	1994-1995	Cardiovascular Health Study	Subnational	70-79	70-79	1,170	1,707					Enzymatic		Plasma	Yes (other)	

	Country	Data years	Survey/Study name/Citation	Level of representati-veness	Age range as used for the analysis		Sample size (Total cholesterol)		Sample size (HDL cholesterol)		Sample size (Non-HDL cholesterol)		Method used to measure		Whether lipids were measured in serum or plasma samples	Participating to a lipid standardisation programme or quality control schemes	Note
					Male	Female	Male	Female	Male	Female	Male	Female	Total cholesterol	HDL cholesterol (separation and quantification methods)			
440	United States of America	1996-1998	Atherosclerosis Risk in Communities Study	Subnational	50-69	50-69	2,896	3,789	2,895	3,788	2,895	3,788	Enzymatic	Dextran sulphate-Magnesium method; Enzymatic	Plasma	Yes (CDC/CRMLN)	
441	United States of America	1996-1997	Cardiovascular Health Study	Subnational	70-79	70-79	936	1,418					Enzymatic		Plasma	Yes (other)	
442	United States of America	1996-1997	Study of Women's Health Across the Nation	Subnational		40-49		2,862		2,862		2,862	Enzymatic	Heparin-Manganese method; Enzymatic	Plasma	Yes (CDC/CRMLN)	7
443	United States of America	1997-1998	Cardiovascular Health Study	Subnational	70-79	70-79	694	1,125					Enzymatic		Plasma	Yes (other)	
444	United States of America	1997-1999	Study of Women's Health Across the Nation	Subnational		40-49		2,202		2,199		2,199	Enzymatic	Heparin-Manganese method; Enzymatic	Plasma	Yes (CDC/CRMLN)	7
445	United States of America	1999-2000	US NHANES 1999-2000	National	40-79	40-79	1,165	1,200	1,163	1,199	1,163	1,198	Enzymatic	Heparin-Manganese method; Enzymatic and Homogeneous assay (alpha-Cyclodextrin + PEG-coupled enzymes)	Serum	Yes (CDC/CRMLN)	
446	United States of America	1999-2001	Study of Women's Health Across the Nation	Subnational		40-49		1,364		1,362		1,362	Enzymatic	Heparin-Manganese method; Enzymatic	Plasma	Yes (CDC/CRMLN)	7
447	United States of America	2000-2002	Study of Women's Health Across the Nation	Subnational		40-49		1,043		1,043		1,043	Enzymatic	Heparin-Manganese method; Enzymatic	Plasma	Yes (CDC/CRMLN)	7
448	United States of America	2001-2002	US NHANES 2001-2002	National	40-79	40-79	1,349	1,267	1,349	1,267	1,349	1,267	Enzymatic	Heparin-Manganese method; Enzymatic and Homogeneous assay (alpha-Cyclodextrin + PEG-coupled enzymes)	Serum	Yes (CDC/CRMLN)	
449	United States of America	2003-2004	US NHANES 2003-2004	National	40-79	40-79	1,257	1,252	1,257	1,252	1,257	1,252	Enzymatic	Homogeneous assay (alpha-Cyclodextrin + PEG-coupled enzymes)	Serum	Yes (CDC/CRMLN)	
450	United States of America	2005-2006	Coronary Artery Risk Development in Young Adults (CARDIA)	Subnational	40-49	40-49	1,237	1,549	1,236	1,549	1,235	1,549	Enzymatic	Dextran sulphate-Magnesium method; Enzymatic	Plasma	Unknown	
451	United States of America	2005-2006	Cardiovascular Health Study	Subnational	70-79	70-79	266	495	266	495	266	495	Enzymatic	Dextran sulphate-Magnesium method; Enzymatic	Plasma	Yes (other)	
452	United States of America	2005-2006	US NHANES 2005-2006	National	40-79	40-79	1,247	1,203	1,247	1,203	1,247	1,203	Enzymatic	Homogeneous assay (alpha-Cyclodextrin + PEG-coupled enzymes)	Serum	Yes (CDC/CRMLN)	
453	United States of America	2007-2008	US NHANES 2007-2008	National	40-79	40-79	1,617	1,659	1,511	1,536	1,511	1,536	Enzymatic	Homogeneous assay (Dextran sulphate-Magnesium + PEG-coupled enzymes)	Serum	Yes (CDC/CRMLN)	
454	United States of America	2009-2010	US NHANES 2009-2010	National	40-79	40-79	1,692	1,730	1,691	1,730	1,691	1,730	Enzymatic	Homogeneous assay (Dextran sulphate-Magnesium + PEG-coupled enzymes)	Serum	Yes (CDC/CRMLN)	
455	United States of America	2011-2013	Atherosclerosis Risk in Communities Study	Subnational	70-79	70-79	1,172	1,639	1,172	1,639	1,172	1,639	Enzymatic	Homogeneous assay	Plasma	Yes (CDC/CRMLN)	
456	United States of America	2011-2012	US NHANES 2011-2012	National	40-79	40-79	1,417	1,455	1,417	1,455	1,417	1,455	Enzymatic	Homogeneous assay (Dextran sulphate-Magnesium + PEG-coupled enzymes)	Serum	Yes (CDC/CRMLN)	

	Country	Data years	Survey/Study name/Citation	Level of representati-veness	Age range as used for the analysis		Sample size (Total cholesterol)		Sample size (HDL cholesterol)		Sample size (Non-HDL cholesterol)		Method used to measure		Whether lipids were measured in serum or plasma samples	Participating to a lipid standardisation programme or quality control schemes	Note
					Male	Female	Male	Female	Male	Female	Male	Female	Total cholesterol	HDL cholesterol (separation and quantification methods)			
457	United States of America	2013-2014	US NHANES 2013-2014	National	40-79	40-79	1,523	1,697	1,523	1,698	1,523	1,697	Enzymatic	Homogeneous assay (Dextran sulphate-Magnesium + PEG-coupled enzymes)	Serum	Yes (CDC/CRMLN)	
458	United States of America	2015-2016	US NHANES 2015-2016	National	40-79	40-79	1,498	1,599	1,497	1,598	1,497	1,598	Enzymatic	Homogeneous assay (Dextran sulphate-Magnesium + PEG-coupled enzymes)	Serum	Yes (CDC/CRMLN)	

1. Data were available only as summary statistics, which did not include mean total-to-HDL cholesterol ratio.
2. Electrophoresis was used for measuring lipid fractions. As this method may be inaccurate in quantifying lipid fractions, mean HDL and non-HDL cholesterol and mean total-to-HDL cholesterol ratio were not included in this analysis.
3. Lipid fractions were measured only since 2009. As nationally representative health examination surveys measured lipid fractions almost every year since 1998 we excluded this data from the analysis.
4. This research uses data from China Health and Nutrition Survey (CHNS). We thank the National Institute of Nutrition and Food Safety, China Center for Disease Control and Prevention, Carolina Population Center (5 R24 HD050924), the University of North Carolina at Chapel Hill, the NIH (R01-HD30880, DK056350, R24-HD050924, and R01-HD38700) and the Fogarty International Center, NIH for financial support for the CHNS data collection and analysis files from 1989 to 2011 and future surveys, and the China-Japan Friendship Hospital, Ministry of Health for support for CHNS 2009.
5. The British Women’s Heart and Health Study is supported by the British Heart Foundation (PG/13/66/30442). British Women’s Heart and Health Study data are available to bona fide researchers for research purposes. Please refer to the BWHHS data sharing policy at http://www.ucl.ac.uk/british-womens-heart-health-study.
6. We thank Prof Stephen Fortmann for data from the Stanford Five-City Project.
7. The bibliographic citation for this data source is: Sutton-Tyrrell, Kim, Faith Selzer, MaryFran Sowers, Robert Neer, Lynda Powell, Ellen Gold, Gail Greendale, Gerson Weiss, Karen Matthews, and Sonja McKinlay. Study of Women's Health Across the Nation (SWAN), 1996-1997: Baseline Dataset. ICPSR28762-v2. Ann Arbor, MI: Inter-university Consortium for Political and Social Research[distributor], 2014-02-04. http://doi.org/10.3886/ICPSR28762.v2

Participating to a lipid standardisation programme or quality control schemes abbreviations: CDC/CRMLN = CDC/Cholesterol Reference Method Laboratory Network Lipid Standardization Program; Other = Participation in internal and external quality control schemes; WHO-LRC = WHO Regional Lipid Reference Centre in Prague; Unknown = Information unavailable or lipids measured in multiple laboratories.

Supplementary Table 2: Data availability by country for ages 40-59 years.

Country (abbreviation)	Total cholesterol			Non-HDL and HDL cholesterol		
	Start year	End year	Number of data sources	Start year	End year	Number of data sources
Australia (AUS)	1980	2012	15	1980	2012	15
Belgium (BEL)	1984	2015	20 (men)<br>18 (women)	1984	2015	20 (men)<br>18 (women)
Canada (CAN)	1985	2015	9	1985	2015	9
China (CHN)	1983	2015	35 (men)<br>34 (women)	1983	2015	35 (men)<br>34 (women)
Czech Republic (CZE)	1985	2018	7	1985	2018	7
Finland (FIN)	1972	2012	15 (men)<br>12 (women)	1982	2012	10
France (FRA)	1985	2013	13 (men)<br>12 (women)	1985	2013	13 (men)<br>12 (women)
Germany (DEU)	1982	2012	39	1982	2012	36
Iceland (ISL)	1970/1971*	2012	12	1983	2012	9
Italy (ITA)	1982	2012	23 (men)<br>21 (women)	1982	2012	23 (men)<br>21 (women)
Japan (JPN)	1980	2016	56	1980	2016	52
Lithuania (LTU)	1972/1983*	2008	11 (men)<br>9 (women)	1983	2008	9
New Zealand (NZL)	1982	2009	6	1982	2009	5
Norway (NOR)	1979	2008	7	1979	2008	7
Poland (POL)	1983	2016	22 (men)<br>20 (women)	1983	2016	20 (men)<br>18 (women)
Slovakia (SVK)	1993	2012	5	1993	2012	5
South Korea (KOR)	1998	2016	20	1998	2016	13
Spain (ESP)	1986	2014	20 (men)<br>18 (women)	1986	2014	18 (men)<br>16 (women)
Sweden (SWE)	1985	2014	14 (men)<br>12 (women)			
Switzerland (CHE)	1984	2017	7	1984	2017	7
Thailand (THA)	1991	2009	6			
United Kingdom (GBR)	1983	2016	36 (men)<br>34 (women)	1983	2016	32 (men)<br>30 (women)
United States of America (USA)	1971	2016	20 (men)<br>24 (women)	1976	2016	19 (men)<br>23 (women)

* Data were available from 1970 for men and from 1971 for women (Iceland), and from 1972 for men and from 1983 for women (Lithuania).

Supplementary Table 3: Change per decade in mean total cholesterol by sex in people aged 40-59 years, shown graphically in Figure 1 in the main paper. Results for each country apply to its entire period of total cholesterol data availability (Supplementary Table 2). Numbers in brackets show 95% confidence intervals.

Country	Change per decade in mean total cholesterol (mmol/L)	
	Men	Women
Australia	-0.18 (-0.22 to -0.14)	-0.19 (-0.23 to -0.16)
Belgium	-0.37 (-0.41 to -0.32)	-0.38 (-0.44 to -0.32)
Canada	-0.23 (-0.32 to -0.13)	-0.22 (-0.34 to -0.10)
China	0.32 (0.23 to 0.41)	0.27 (0.16 to 0.38)
Czech Republic	-0.34 (-0.41 to -0.27)	-0.35 (-0.42 to -0.28)
Finland	-0.40 (-0.46 to -0.34)	-0.43 (-0.51 to -0.36)
France	-0.08 (-0.16 to -0.01)	-0.03 (-0.10 to 0.04)
Germany	-0.28 (-0.34 to -0.22)	-0.28 (-0.34 to -0.23)
Iceland	-0.27 (-0.32 to -0.23)	-0.33 (-0.37 to -0.28)
Italy	-0.12 (-0.19 to -0.05)	-0.06 (-0.12 to 0.00)
Japan	0.14 (0.12 to 0.16)	0.13 (0.11 to 0.16)
Lithuania	-0.05 (-0.13 to 0.04)	-0.27 (-0.41 to -0.12)
New Zealand	-0.08 (-0.24 to 0.08)	-0.22 (-0.40 to -0.05)
Norway	-0.41 (-0.49 to -0.33)	-0.42 (-0.51 to -0.33)
Poland	0.00 (-0.05 to 0.06)	-0.02 (-0.08 to 0.03)
Slovakia	-0.22 (-0.43 to -0.01)	-0.37 (-0.60 to -0.14)
South Korea	0.03 (0.01 to 0.05)	0.04 (0.02 to 0.05)
Spain	-0.16 (-0.22 to -0.11)	-0.11 (-0.17 to -0.05)
Sweden	-0.30 (-0.43 to -0.17)	-0.30 (-0.41 to -0.18)
Switzerland	-0.34 (-0.48 to -0.20)	-0.27 (-0.40 to -0.14)
Thailand	0.29 (0.22 to 0.36)	0.29 (0.16 to 0.41)
United Kingdom	-0.24 (-0.29 to -0.20)	-0.25 (-0.29 to -0.22)
United States of America	-0.18 (-0.25 to -0.11)	-0.12 (-0.18 to -0.06)

Supplementary Table 4: Change per decade in mean HDL and non-HDL cholesterol, and in mean total-to-HDL cholesterol ratio by sex in people aged 40-59 years, shown graphically in Figure 2 and Figure 4 in the main paper. Results for each country apply to its period of HDL and non-HDL cholesterol data availability (Supplementary Table 2). Numbers in brackets show 95% confidence intervals.

Country	Change per decade in mean HDL cholesterol (mmol/L)		Change per decade in mean non-HDL cholesterol (mmol/L)		Change per decade in mean total-to-HDL cholesterol ratio	
	Men	Women	Men	Women	Men	Women
Australia	0.013 (-0.001 to 0.027)	0.010 (-0.003 to 0.024)	-0.20 (-0.24 to -0.15)	-0.20 (-0.24 to -0.17)	-0.26 (-0.33 to -0.18)	-0.18 (-0.23 to -0.13)
Belgium	0.045 (0.014 to 0.077)	0.036 (0.004 to 0.067)	-0.41 (-0.47 to -0.36)	-0.43 (-0.48 to -0.37)	-0.55 (-0.67 to -0.43)	-0.38 (-0.45 to -0.30)
Canada	-0.005 (-0.025 to 0.014)	-0.006 (-0.032 to 0.020)	-0.23 (-0.31 to -0.15)	-0.21 (-0.31 to -0.11)	-0.25 (-0.35 to -0.16)	-0.15 (-0.22 to -0.08)
China	0.034 (0.001 to 0.067)	-0.006 (-0.045 to 0.033)	0.30 (0.23 to 0.38)	0.25 (0.16 to 0.34)	0.21 (0.14 to 0.27)	0.21 (0.14 to 0.28)
Czech Republic	-0.015 (-0.033 to 0.003)	0.040 (0.012 to 0.067)	-0.33 (-0.41 to -0.25)	-0.41 (-0.49 to -0.34)	-0.23 (-0.32 to -0.14)	-0.31 (-0.37 to -0.25)
Finland	0.038 (0.018 to 0.058)	0.043 (0.014 to 0.072)	-0.34 (-0.43 to -0.25)	-0.34 (-0.43 to -0.24)	-0.42 (-0.53 to -0.32)	-0.31 (-0.40 to -0.21)
France	0.020 (-0.011 to 0.050)	0.017 (-0.028 to 0.062)	-0.10 (-0.15 to -0.05)	-0.03 (-0.09 to 0.03)	-0.14 (-0.23 to -0.06)	-0.07 (-0.14 to 0.00)
Germany	-0.041 (-0.069 to -0.013)	-0.041 (-0.075 to -0.006)	-0.23 (-0.30 to -0.17)	-0.24 (-0.28 to -0.19)	-0.07 (-0.19 to 0.05)	-0.08 (-0.15 to 0.00)
Iceland	0.029 (0.008 to 0.051)	0.065 (0.038 to 0.091)	-0.44 (-0.54 to -0.33)	-0.53 (-0.61 to -0.46)	-0.42 (-0.56 to -0.29)	-0.45 (-0.54 to -0.36)
Italy	0.021 (-0.004 to 0.046)	0.084 (0.050 to 0.118)	-0.13 (-0.20 to -0.07)	-0.13 (-0.19 to -0.07)	-0.22 (-0.31 to -0.12)	-0.21 (-0.30 to -0.13)
Japan	0.088 (0.080 to 0.096)	0.168 (0.153 to 0.182)	0.02 (-0.01 to 0.04)	-0.08 (-0.11 to -0.06)	-0.16 (-0.19 to -0.13)	-0.27 (-0.29 to -0.24)
Lithuania	0.048 (-0.016 to 0.112)	0.025 (-0.041 to 0.092)	-0.27 (-0.39 to -0.15)	-0.38 (-0.50 to -0.26)	-0.35 (-0.53 to -0.16)	-0.34 (-0.51 to -0.17)
New Zealand	0.118 (0.093 to 0.143)	0.136 (0.110 to 0.162)	-0.16 (-0.30 to -0.02)	-0.30 (-0.46 to -0.14)	-0.61 (-0.78 to -0.45)	-0.53 (-0.70 to -0.36)
Norway	-0.080 (-0.122 to -0.038)	-0.095 (-0.162 to -0.027)	-0.33 (-0.40 to -0.26)	-0.33 (-0.42 to -0.25)	-0.07 (-0.21 to 0.07)	-0.08 (-0.25 to 0.08)
Poland	-0.033 (-0.070 to 0.003)	0.026 (-0.017 to 0.070)	0.01 (-0.04 to 0.07)	-0.06 (-0.10 to -0.01)	0.07 (-0.05 to 0.19)	-0.07 (-0.16 to 0.03)
Slovakia	-0.051 (-0.101 to -0.002)	0.005 (-0.038 to 0.048)	-0.16 (-0.38 to 0.07)	-0.37 (-0.60 to -0.15)	-0.02 (-0.40 to 0.36)	-0.28 (-0.49 to -0.06)
South Korea	0.035 (0.000 to 0.070)	0.093 (0.056 to 0.130)	-0.01 (-0.06 to 0.05)	-0.04 (-0.09 to 0.00)	-0.10 (-0.22 to 0.01)	-0.25 (-0.34 to -0.16)
Spain	0.020 (-0.012 to 0.052)	0.047 (0.005 to 0.090)	-0.19 (-0.25 to -0.12)	-0.16 (-0.23 to -0.09)	-0.28 (-0.40 to -0.15)	-0.23 (-0.34 to -0.12)
Switzerland	0.096 (0.038 to 0.154)	0.152 (0.079 to 0.225)	-0.42 (-0.57 to -0.26)	-0.40 (-0.55 to -0.25)	-0.65 (-0.90 to -0.41)	-0.49 (-0.67 to -0.31)
United Kingdom	0.093 (0.073 to 0.113)	0.111 (0.089 to 0.132)	-0.28 (-0.32 to -0.24)	-0.33 (-0.37 to -0.29)	-0.59 (-0.67 to -0.50)	-0.47 (-0.53 to -0.41)
United States of America	0.024 (0.012 to 0.035)	0.035 (0.020 to 0.050)	-0.20 (-0.27 to -0.12)	-0.10 (-0.17 to -0.03)	-0.24 (-0.34 to -0.14)	-0.13 (-0.20 to -0.06)

Supplementary Figure 1: The association between mean LDL and non-HDL cholesterol in studies with data on both variables. Each data point is one study-sex-age group.

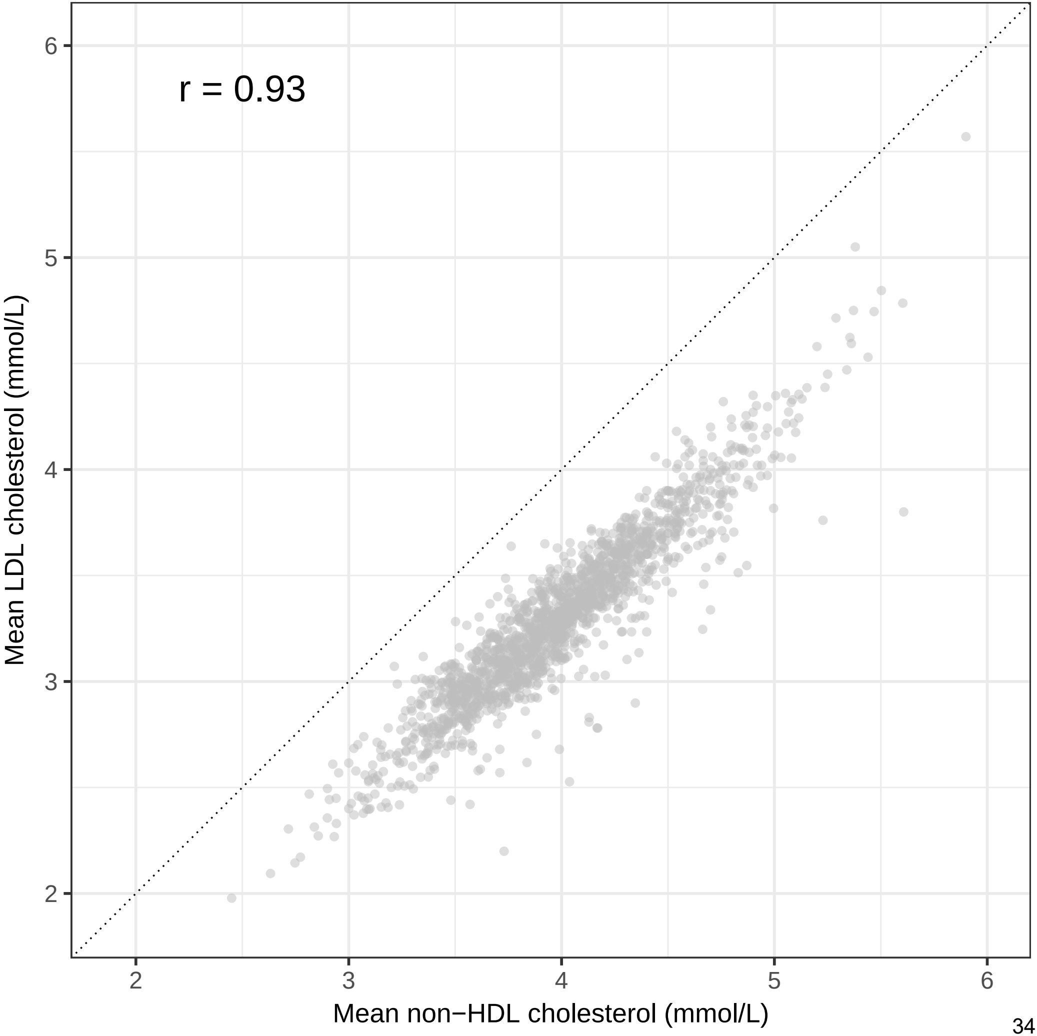
r = 0.93
Mean LDL cholesterol (mmol/L)
Mean non−HDL cholesterol (mmol/L)
2
3
4
5
6
2
3
4
5
6

Supplementary Figure 2: Measurement methods and participation in a lipid standardisation programme in studies with data on HDL cholesterol.

HDL cholesterol measurement method abbreviations: AB = Antibody, two reagents; ASD = Accelerator Selective Detergent; CAT = Catalase; DS = Dextran sulphate-Mg^{2+}; HC = Heparin-Ca^{2+}; HM = Heparin-Mn^{2+}; IE = Immuno-enzymatic; Other = Homogeneous assay: details unavailable or multiple homogeneous assays used, Precipitation: details unavailable or multiple methods used; PEG = Homogeneous assay: Polyethylene glycol-modified enzymes, Precipitation: Polyethylene glycol; PEG + Cyc = Polyethylene glycol-modified enzymes with cyclodextrin; PEG + DS = Polyethylene glycol-modified enzymes with dextran sulphate-Mg^{2+}; PTA = Phosphotungstate-Mg^{2+}; Unknown = Information unavailable or multiple methods used.

Lipid standardisation abbreviations: CDC/CRMLN = CDC/Cholesterol Reference Method Laboratory Network Lipid Standardization Program; Other = Participation in internal and external quality control schemes; WHO-LRC = WHO Regional Lipid Reference Centre in Prague; Unknown = Information unavailable or lipids measured in multiple laboratories.

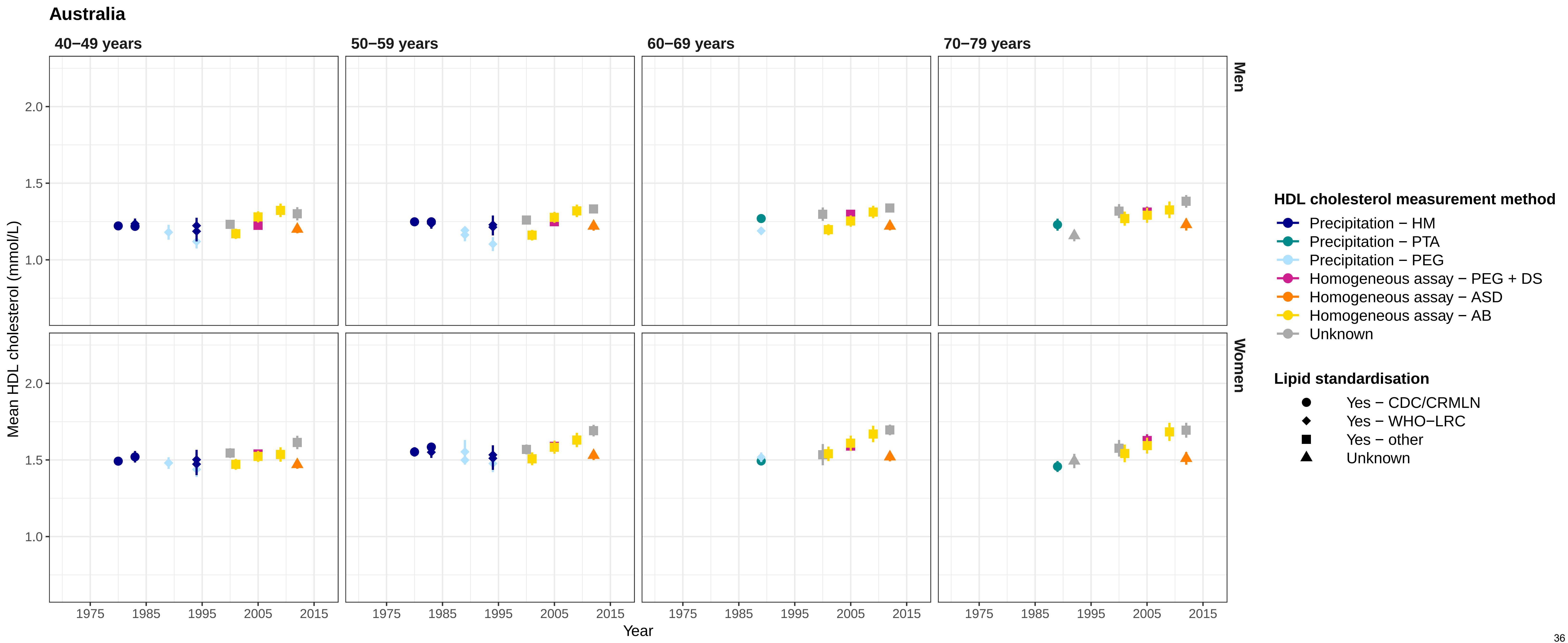

Australia
40−49 years
50−59 years
60−69 years
70−79 years
Men
Women
Mean HDL cholesterol (mmol/L)
Year
HDL cholesterol measurement method
Precipitation − HM
Precipitation − PTA
Precipitation − PEG
Homogeneous assay − PEG + DS
Homogeneous assay − ASD
Homogeneous assay − AB
Unknown
Lipid standardisation
Yes − CDC/CRMLN
Yes − WHO−LRC
Yes − other
Unknown

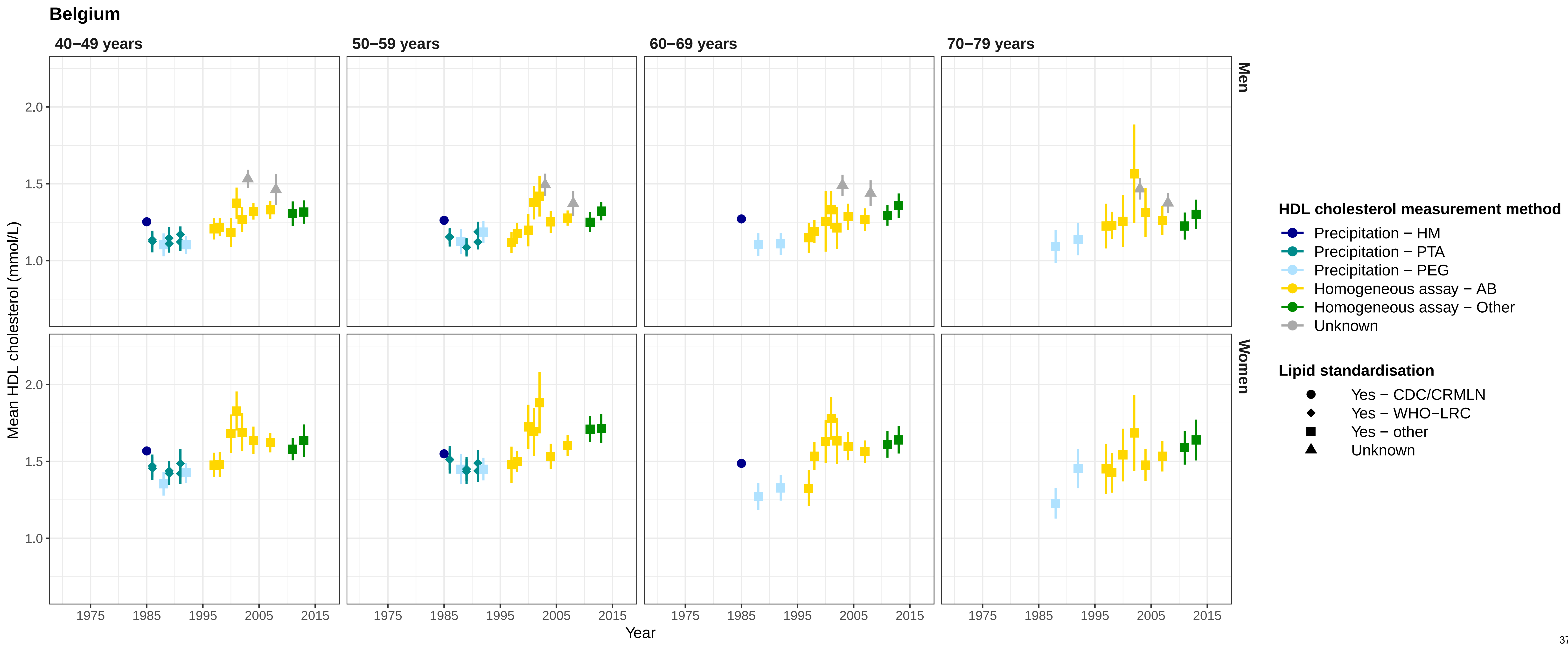

Belgium
40−49 years
50−59 years
60−69 years
70−79 years
Men
Women
Mean HDL cholesterol (mmol/L)
1.0
1.5
2.0
1975
1985
1995
2005
2015
Year
HDL cholesterol measurement method
Precipitation − HM
Precipitation − PTA
Precipitation − PEG
Homogeneous assay − AB
Homogeneous assay − Other
Unknown
Lipid standardisation
Yes − CDC/CRMLN
Yes − WHO−LRC
Yes − other
Unknown

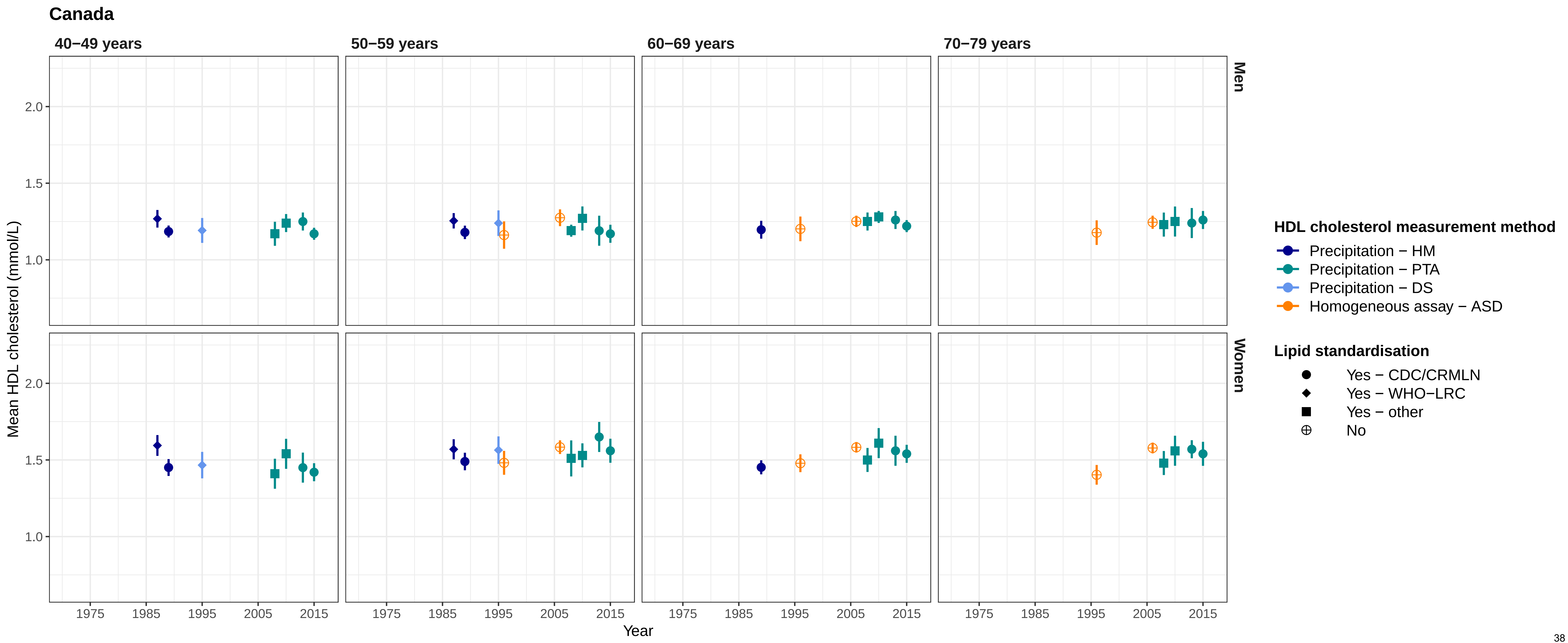

Canada
40−49 years
50−59 years
60−69 years
70−79 years
Men
Women
Mean HDL cholesterol (mmol/L)
2.0
1.5
1.0
1975
1985
1995
2005
2015
Year
HDL cholesterol measurement method
Precipitation − HM
Precipitation − PTA
Precipitation − DS
Homogeneous assay − ASD
Lipid standardisation
Yes − CDC/CRMLN
Yes − WHO−LRC
Yes − other
No

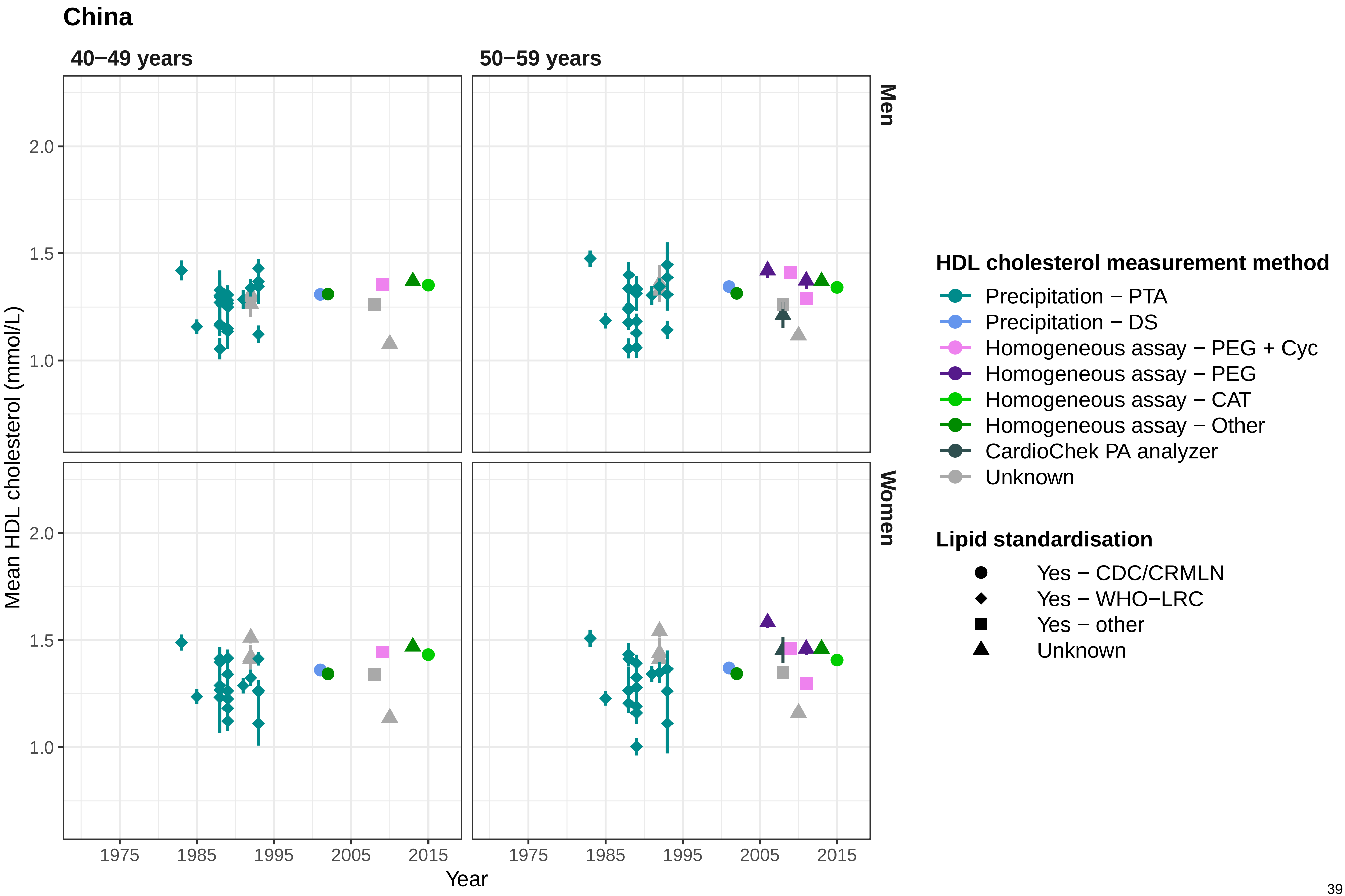
China
40−49 years
50−59 years
Men
Women
Mean HDL cholesterol (mmol/L)
2.0
1.5
1.0
1975
1985
1995
2005
2015
Year
HDL cholesterol measurement method
Precipitation − PTA
Precipitation − DS
Homogeneous assay − PEG + Cyc
Homogeneous assay − PEG
Homogeneous assay − CAT
Homogeneous assay − Other
CardioChek PA analyzer
Unknown
Lipid standardisation
Yes − CDC/CRMLN
Yes − WHO−LRC
Yes − other
Unknown

Czech Republic

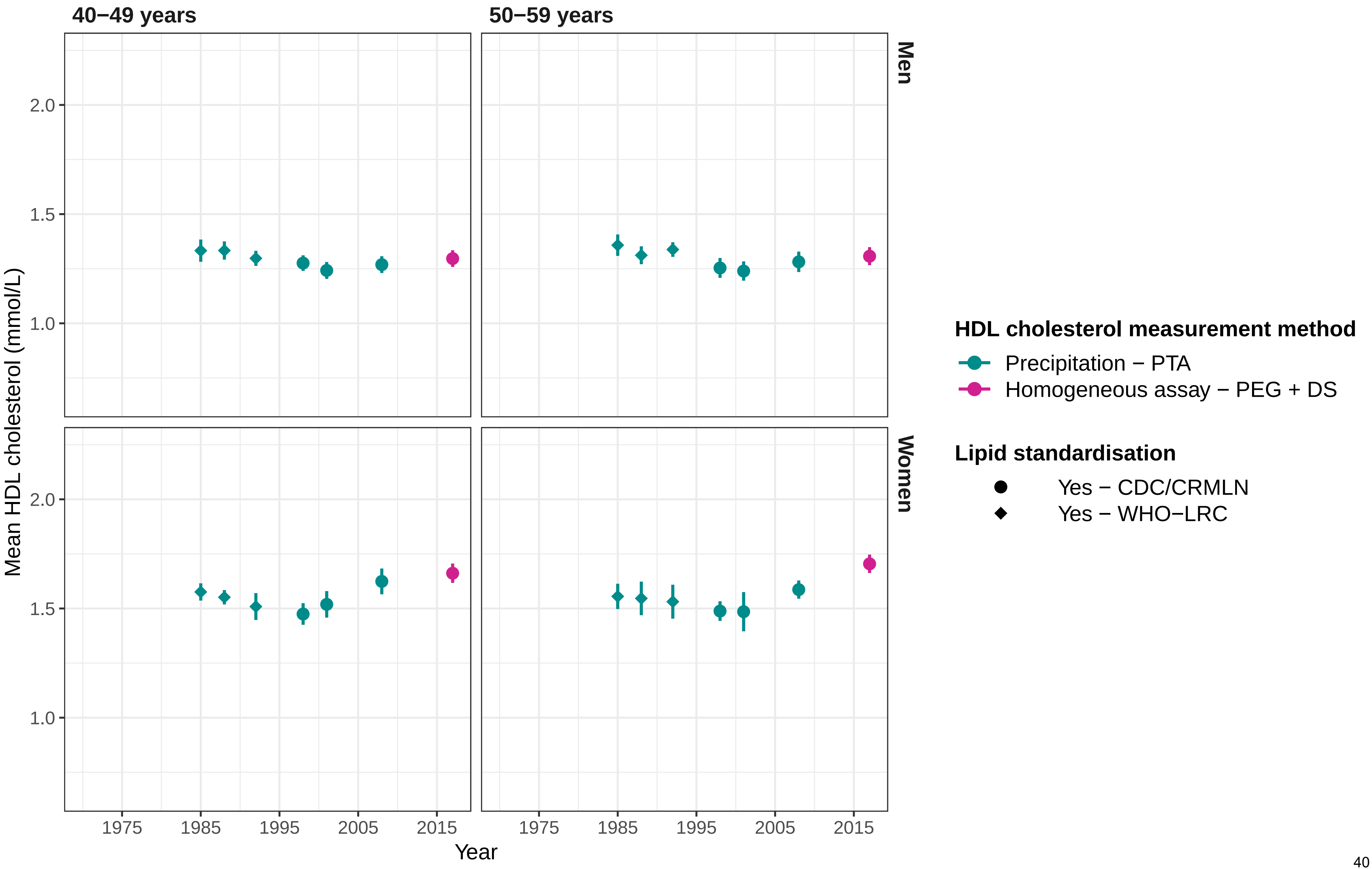

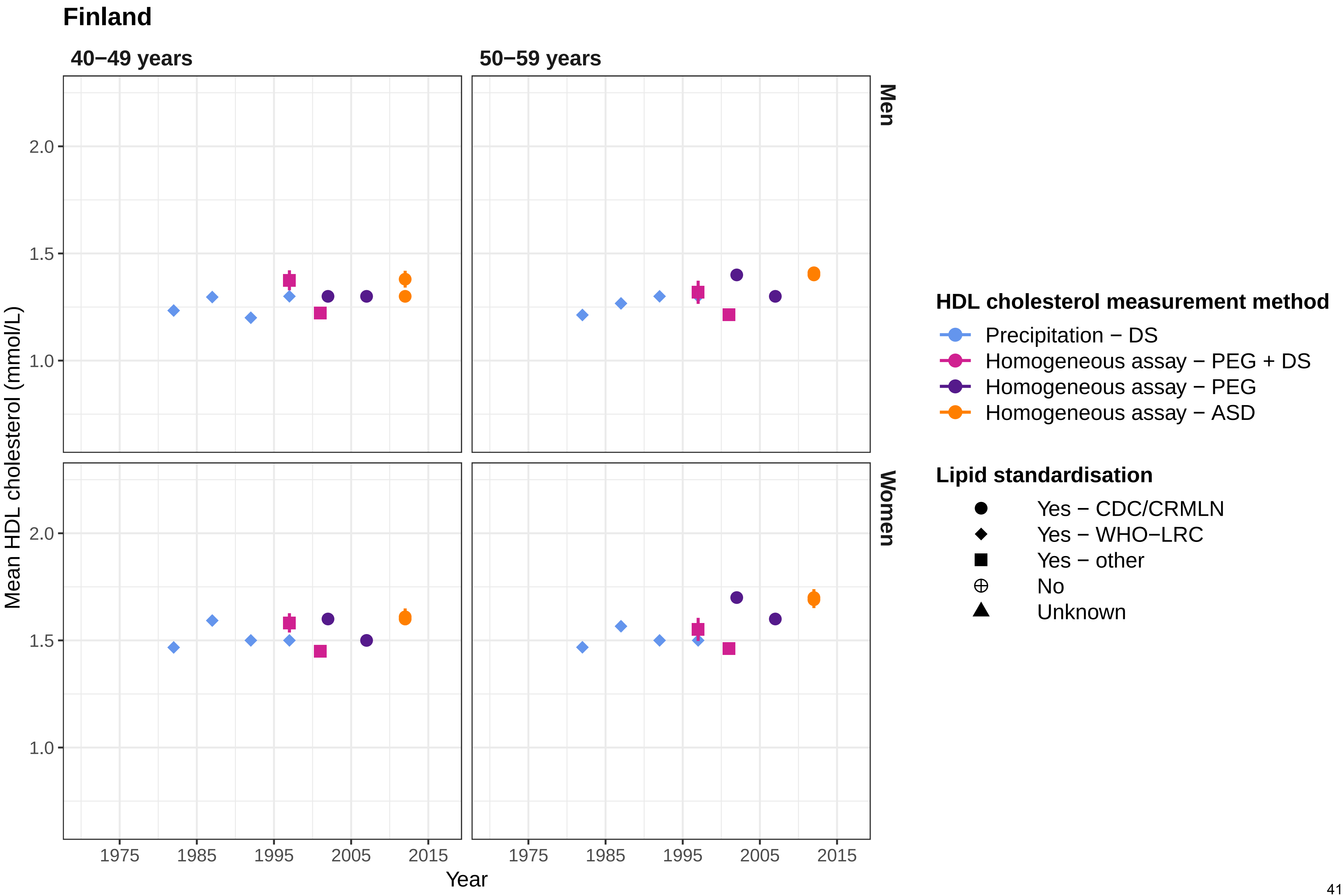
Finland
40−49 years
50−59 years
Men
Women
Mean HDL cholesterol (mmol/L)
2.0
1.5
1.0
1975
1985
1995
2005
2015
Year
HDL cholesterol measurement method
Precipitation − DS
Homogeneous assay − PEG + DS
Homogeneous assay − PEG
Homogeneous assay − ASD
Lipid standardisation
Yes − CDC/CRMLN
Yes − WHO−LRC
Yes − other
No
Unknown

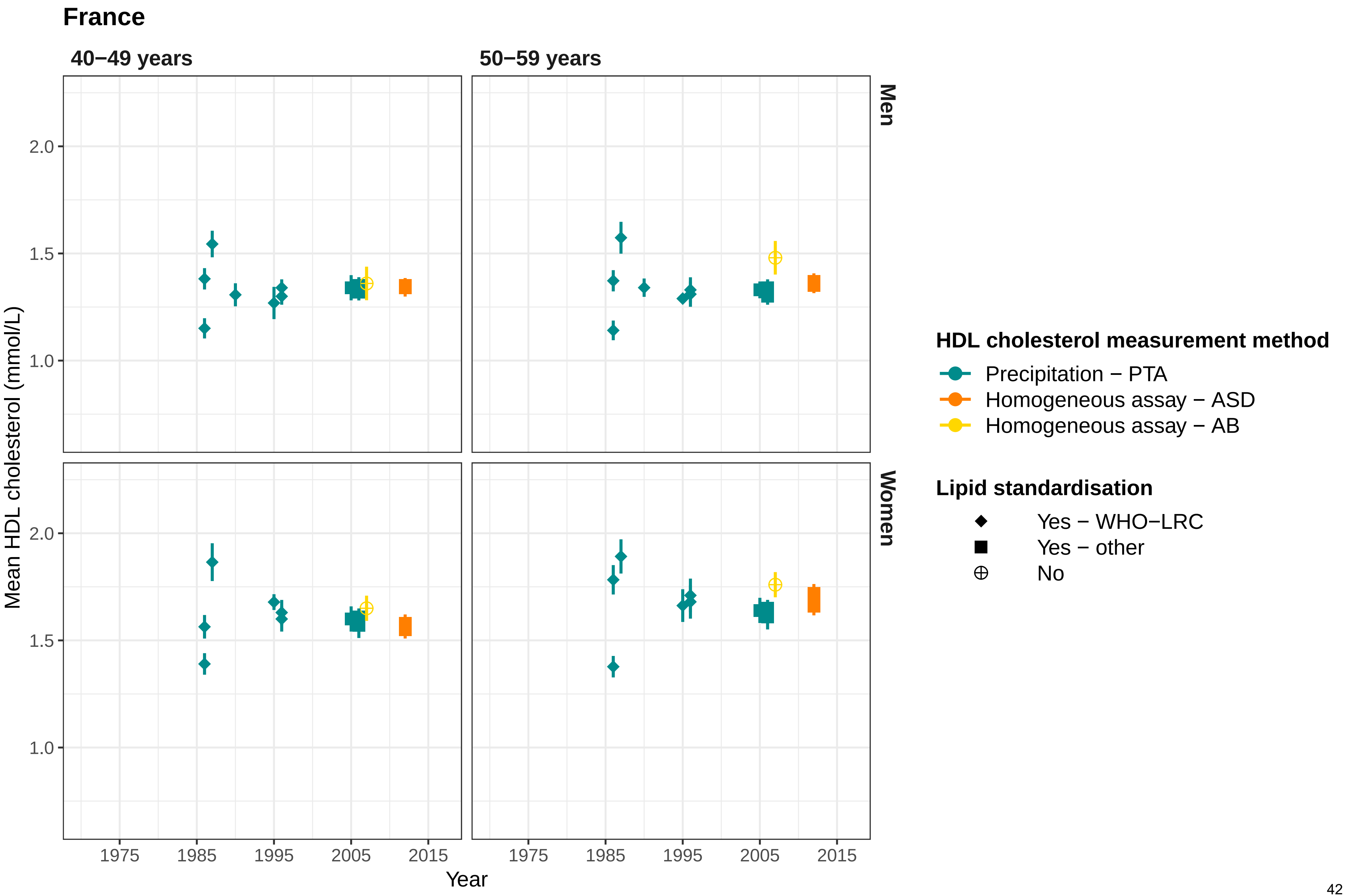

France
40−49 years
50−59 years
Men
Women
Mean HDL cholesterol (mmol/L)
2.0
1.5
1.0
1975
1985
1995
2005
2015
Year
HDL cholesterol measurement method
Precipitation − PTA
Homogeneous assay − ASD
Homogeneous assay − AB
Lipid standardisation
Yes − WHO−LRC
Yes − other
No

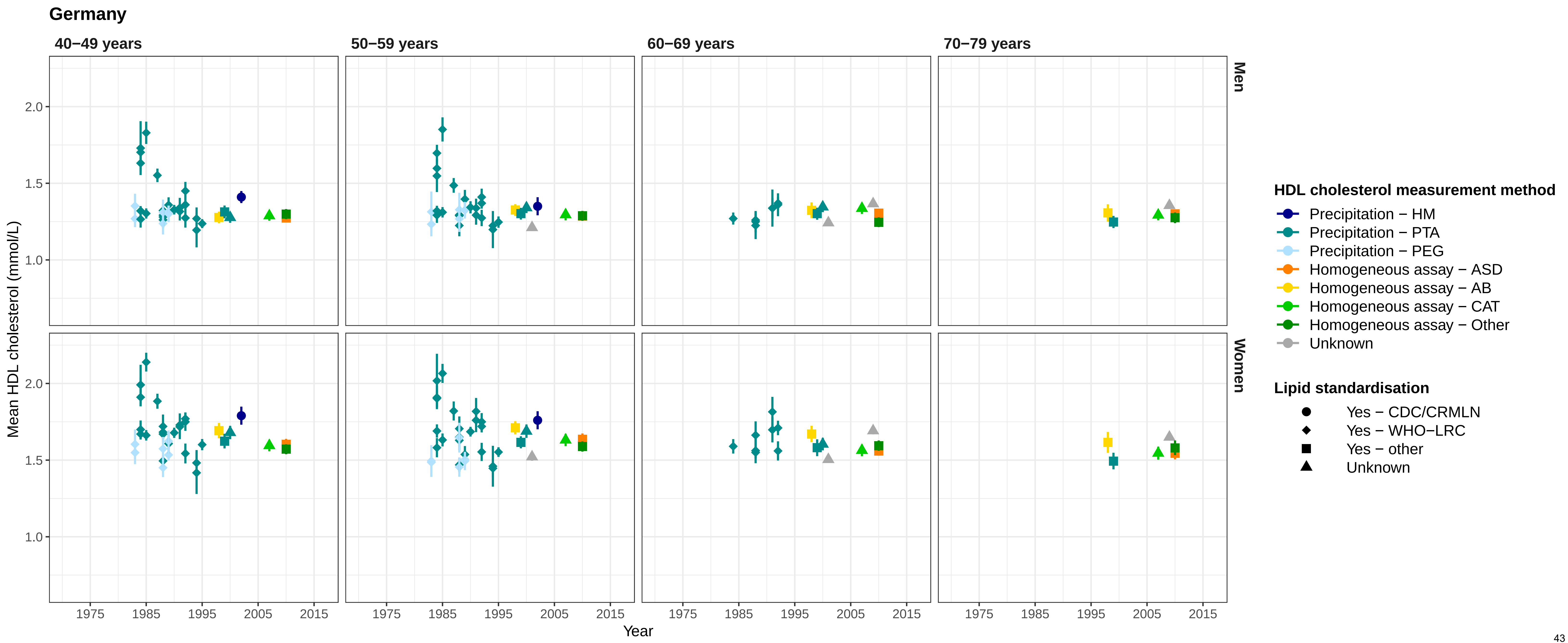

Germany
40−49 years
50−59 years
60−69 years
70−79 years
Men
Women
Mean HDL cholesterol (mmol/L)
2.0
1.5
1.0
1975
1985
1995
2005
2015
Year
HDL cholesterol measurement method
Precipitation − HM
Precipitation − PTA
Precipitation − PEG
Homogeneous assay − ASD
Homogeneous assay − AB
Homogeneous assay − CAT
Homogeneous assay − Other
Unknown
Lipid standardisation
Yes − CDC/CRMLN
Yes − WHO−LRC
Yes − other
Unknown

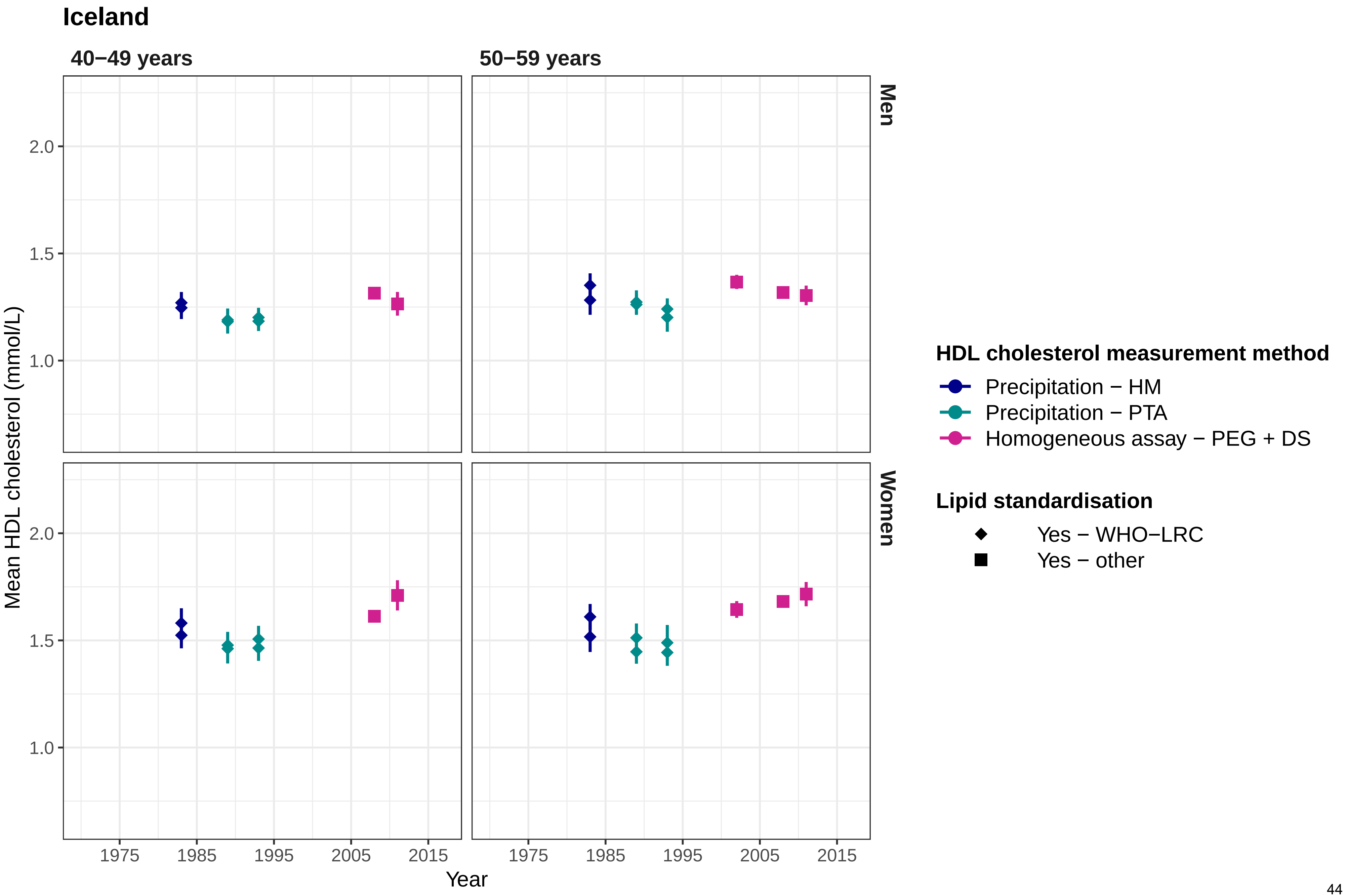
Iceland
40−49 years
50−59 years
Men
Women
Mean HDL cholesterol (mmol/L)
2.0
1.5
1.0
1975
1985
1995
2005
2015
Year
HDL cholesterol measurement method
Precipitation − HM
Precipitation − PTA
Homogeneous assay − PEG + DS
Lipid standardisation
Yes − WHO−LRC
Yes − other

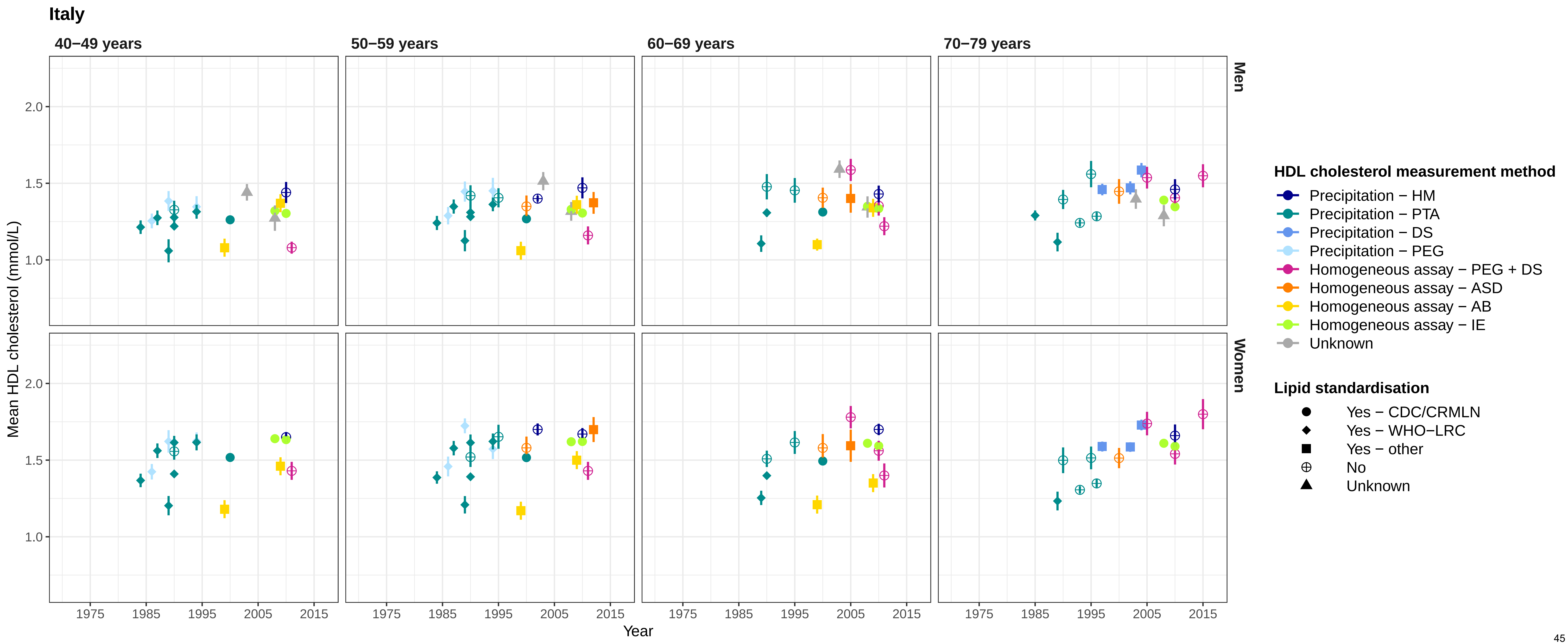
Italy
40−49 years
50−59 years
60−69 years
70−79 years
Men
Women
Mean HDL cholesterol (mmol/L)
1.0
1.5
2.0
1975
1985
1995
2005
2015
Year
HDL cholesterol measurement method
Precipitation − HM
Precipitation − PTA
Precipitation − DS
Precipitation − PEG
Homogeneous assay − PEG + DS
Homogeneous assay − ASD
Homogeneous assay − AB
Homogeneous assay − IE
Unknown
Lipid standardisation
Yes − CDC/CRMLN
Yes − WHO−LRC
Yes − other
No
Unknown

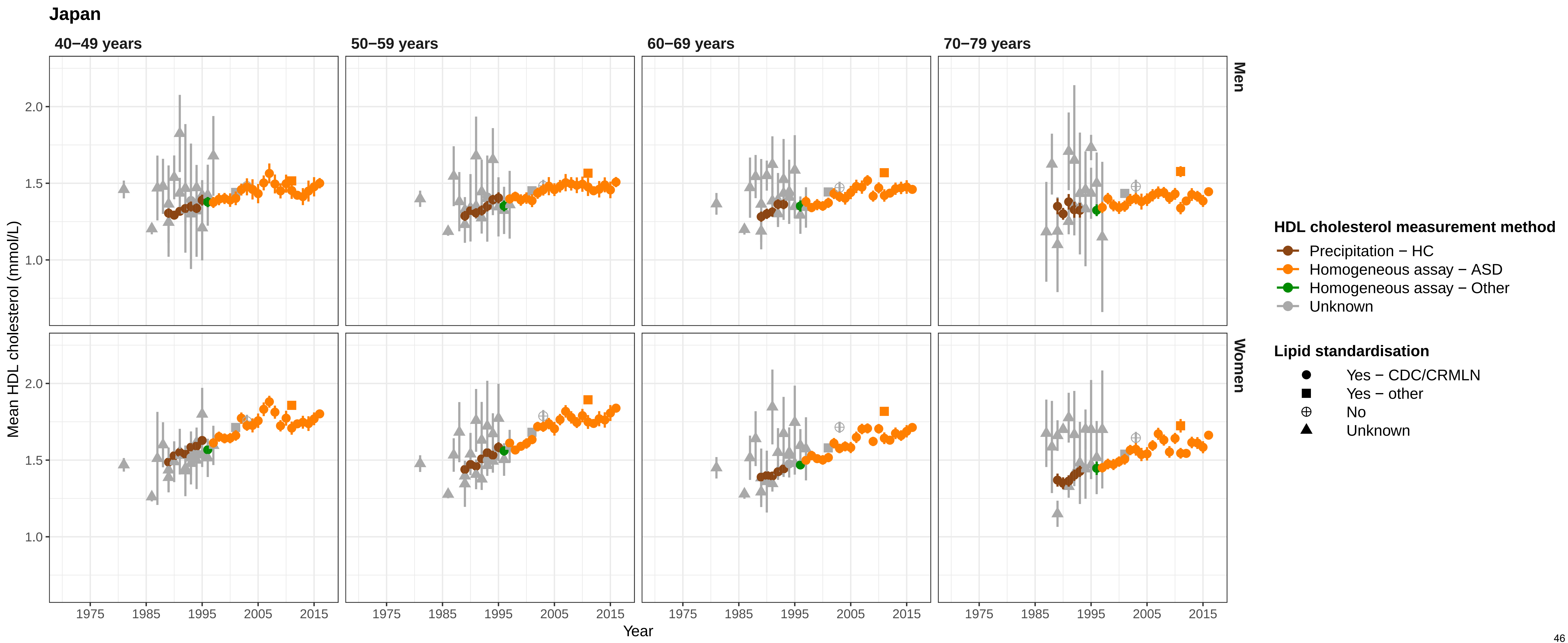

Japan
40−49 years
50−59 years
60−69 years
70−79 years
Men
Women
Mean HDL cholesterol (mmol/L)
1.0
1.5
2.0
1975
1985
1995
2005
2015
Year
HDL cholesterol measurement method
Precipitation − HC
Homogeneous assay − ASD
Homogeneous assay − Other
Unknown
Lipid standardisation
Yes − CDC/CRMLN
Yes − other
No
Unknown

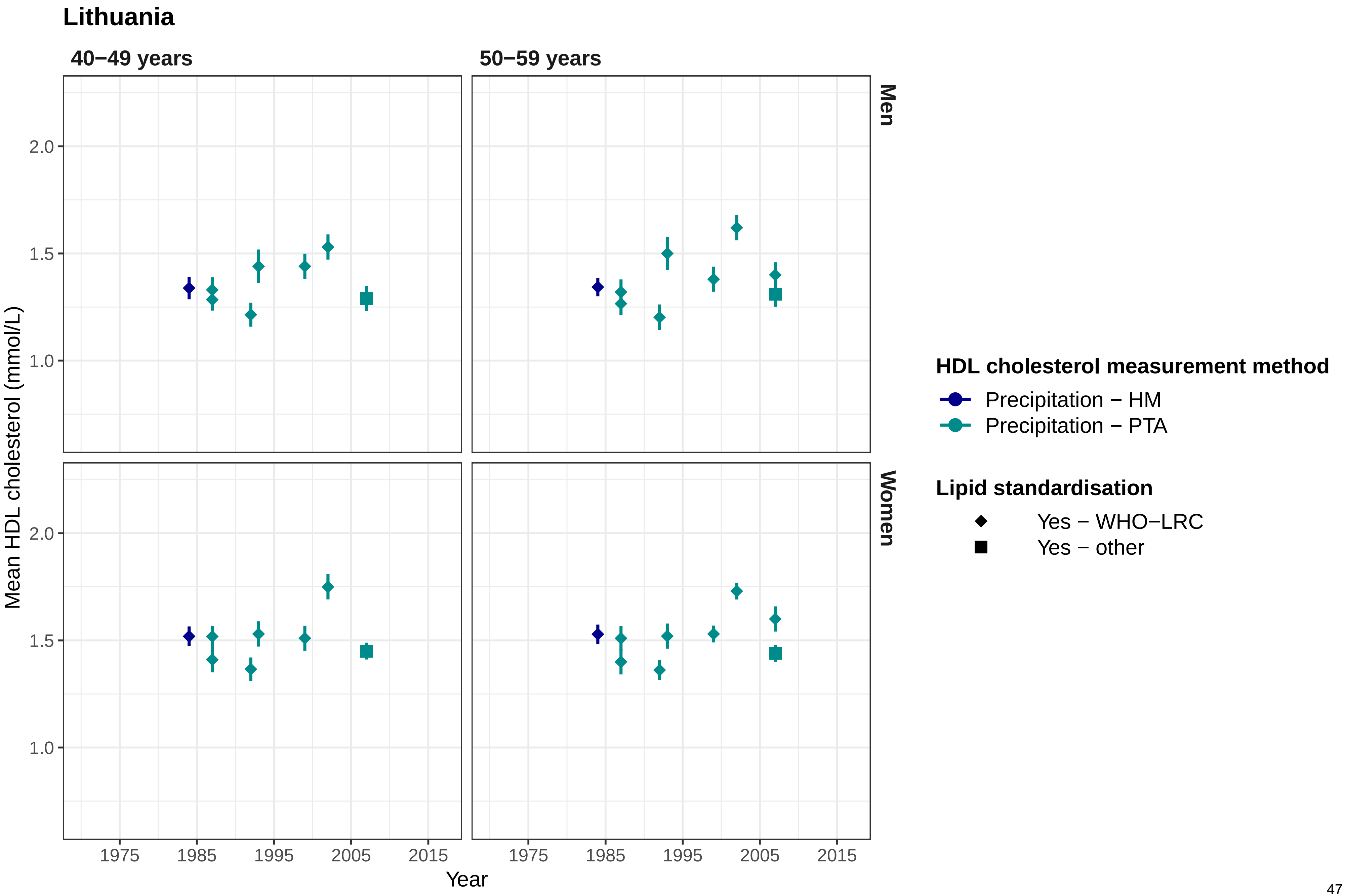

Lithuania
40−49 years
50−59 years
Men
Women
Mean HDL cholesterol (mmol/L)
2.0
1.5
1.0
1975
1985
1995
2005
2015
Year
HDL cholesterol measurement method
Precipitation − HM
Precipitation − PTA
Lipid standardisation
Yes − WHO−LRC
Yes − other

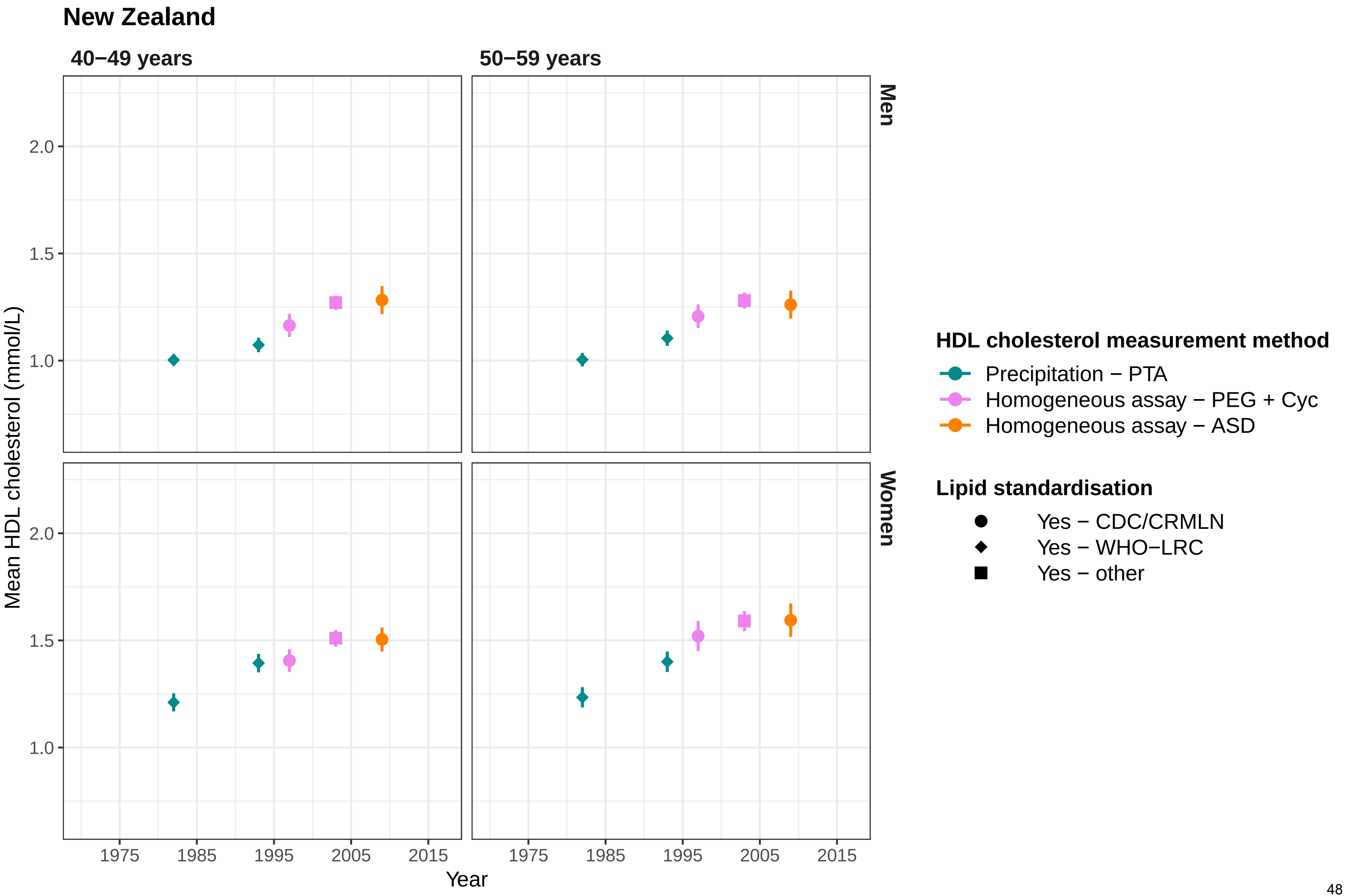
New Zealand
40−49 years
50−59 years
Men
Women
Mean HDL cholesterol (mmol/L)
2.0
1.5
1.0
1975
1985
1995
2005
2015
Year
HDL cholesterol measurement method
Precipitation − PTA
Homogeneous assay − PEG + Cyc
Homogeneous assay − ASD
Lipid standardisation
Yes − CDC/CRMLN
Yes − WHO−LRC
Yes − other

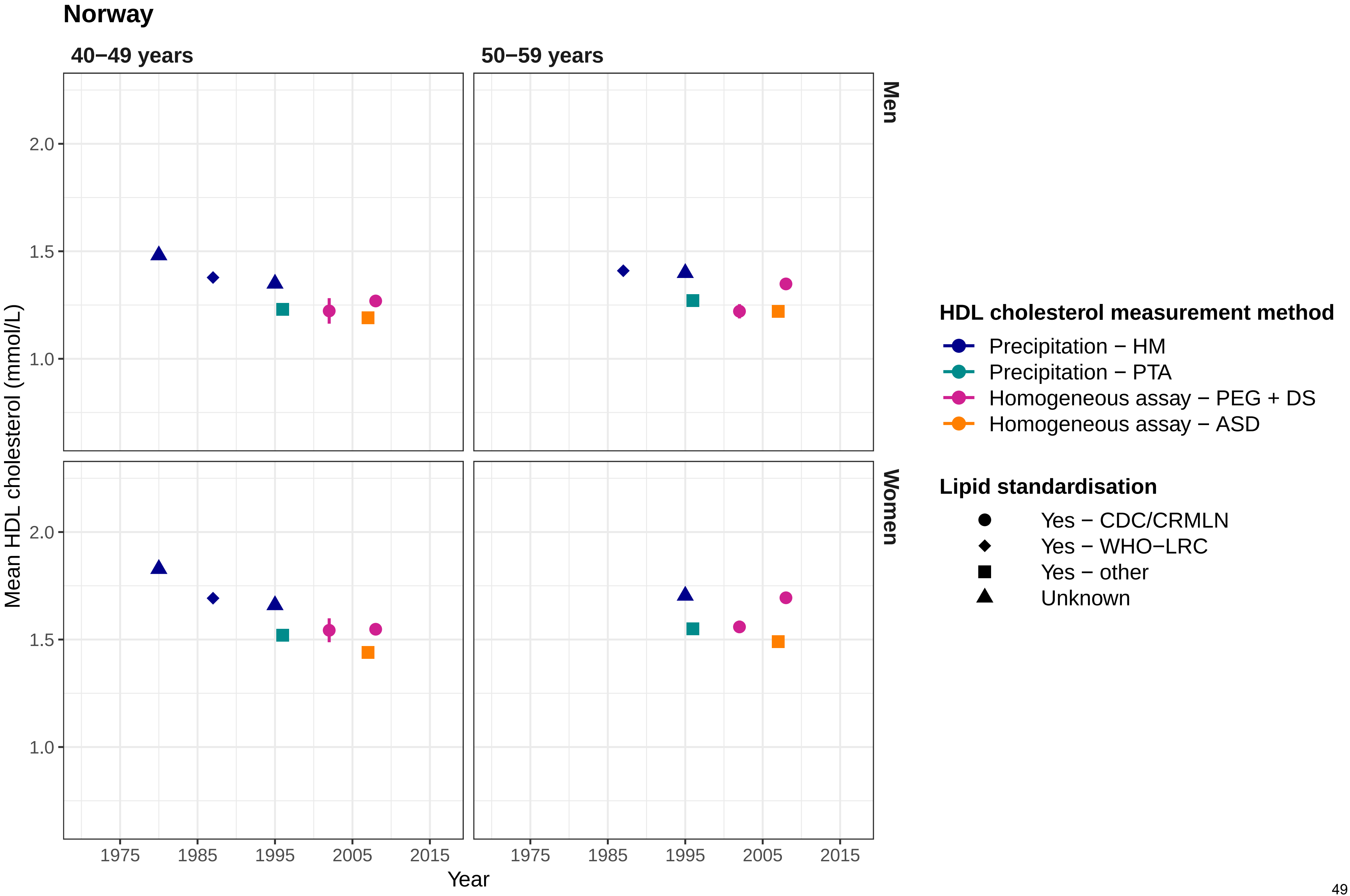

Norway
40−49 years
50−59 years
Men
Women
Mean HDL cholesterol (mmol/L)
2.0
1.5
1.0
1975
1985
1995
2005
2015
Year
HDL cholesterol measurement method
Precipitation − HM
Precipitation − PTA
Homogeneous assay − PEG + DS
Homogeneous assay − ASD
Lipid standardisation
Yes − CDC/CRMLN
Yes − WHO−LRC
Yes − other
Unknown

Poland

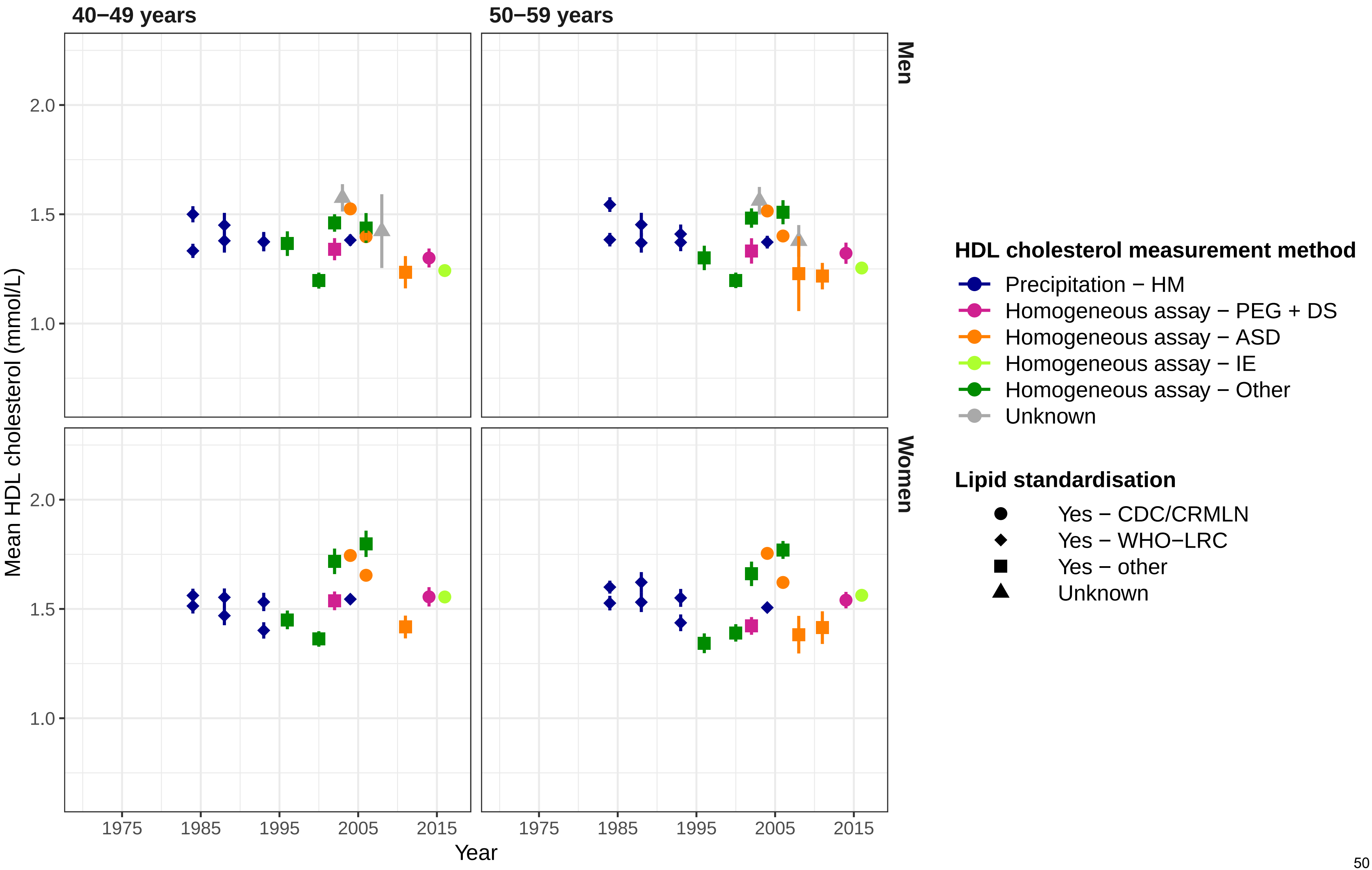
40−49 years
50−59 years
Men
Women
Mean HDL cholesterol (mmol/L)
2.0
1.5
1.0
Year
1975
1985
1995
2005
2015
HDL cholesterol measurement method
Precipitation − HM
Homogeneous assay − PEG + DS
Homogeneous assay − ASD
Homogeneous assay − IE
Homogeneous assay − Other
Unknown
Lipid standardisation
Yes − CDC/CRMLN
Yes − WHO−LRC
Yes − other
Unknown

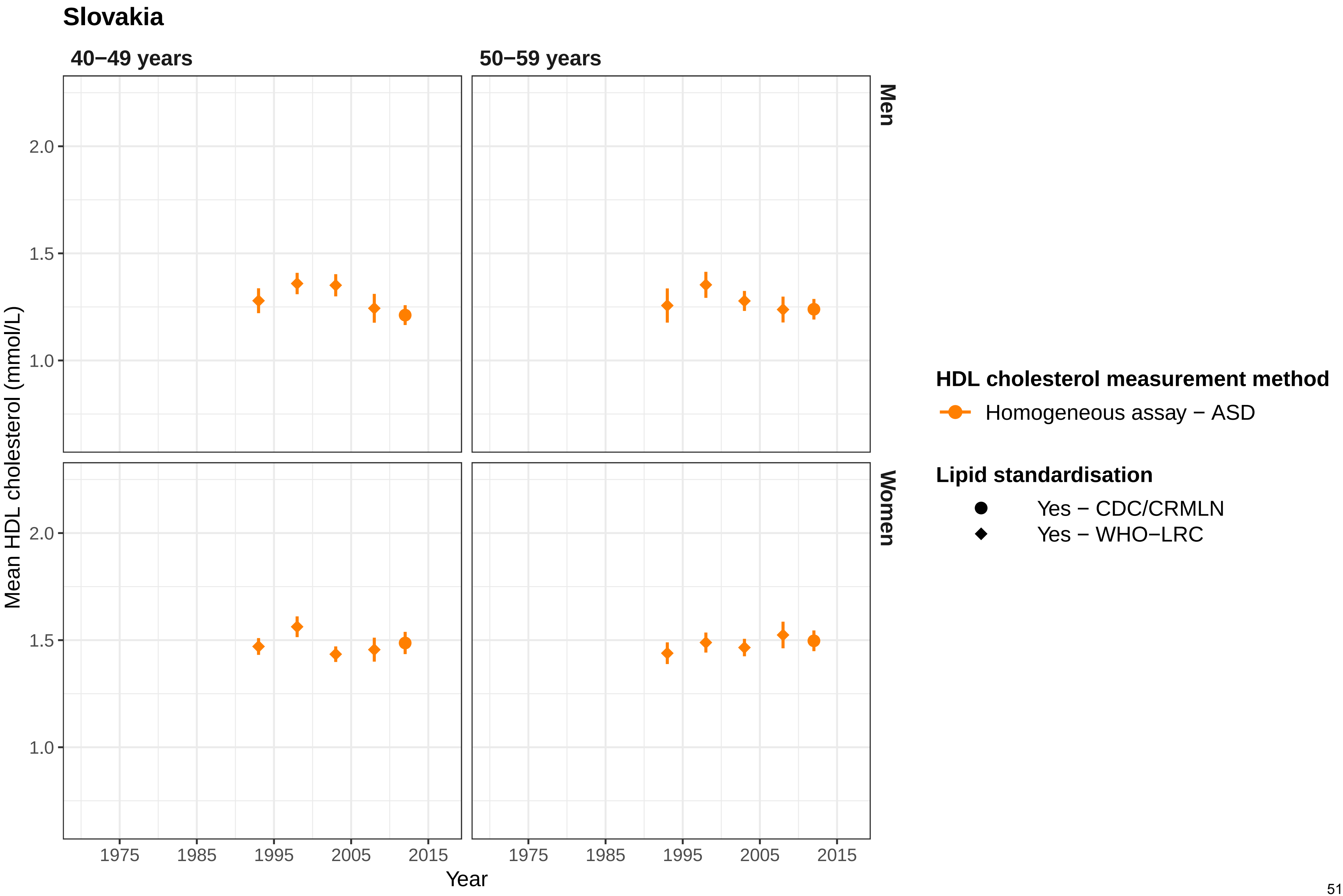
Slovakia
40−49 years
50−59 years
Men
Women
Mean HDL cholesterol (mmol/L)
2.0
1.5
1.0
1975
1985
1995
2005
2015
Year
HDL cholesterol measurement method
Homogeneous assay − ASD
Lipid standardisation
Yes − CDC/CRMLN
Yes − WHO−LRC

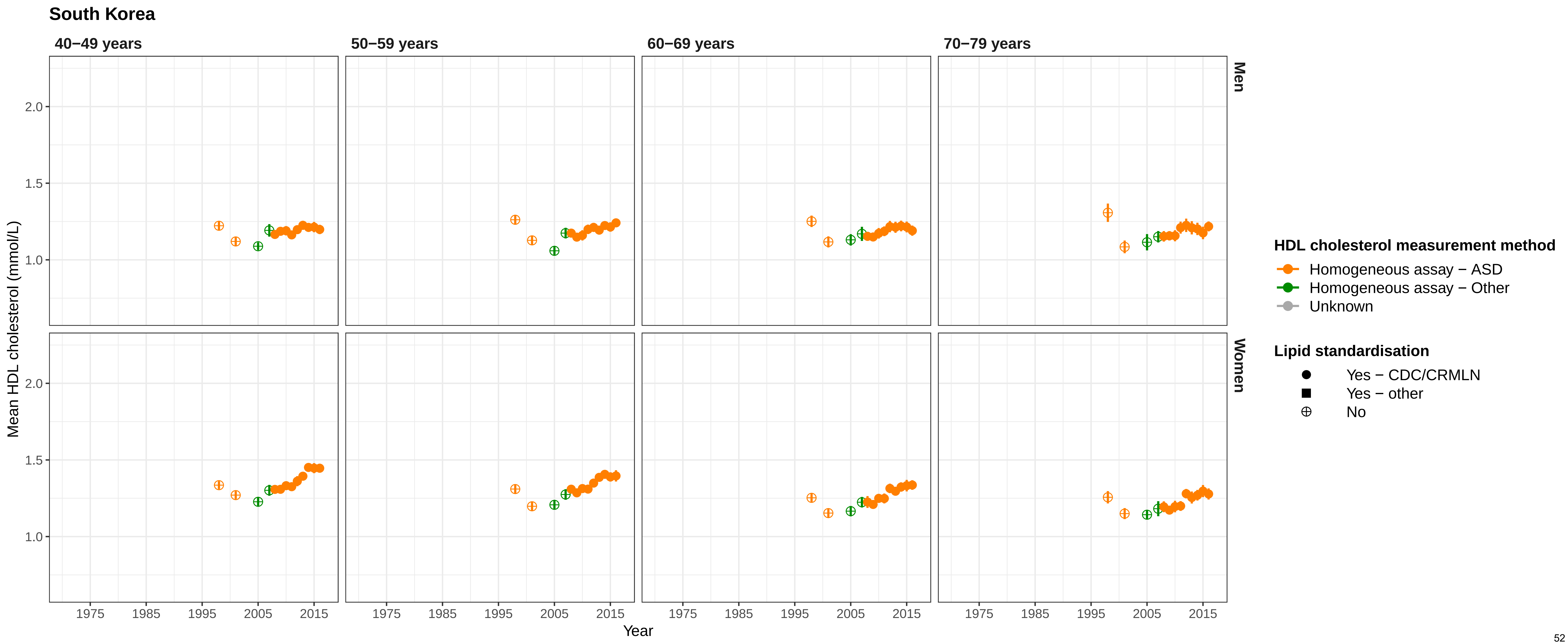
South Korea
40−49 years
50−59 years
60−69 years
70−79 years
Men
Women
Mean HDL cholesterol (mmol/L)
2.0
1.5
1.0
1975
1985
1995
2005
2015
Year
HDL cholesterol measurement method
Homogeneous assay − ASD
Homogeneous assay − Other
Unknown
Lipid standardisation
Yes − CDC/CRMLN
Yes − other
No

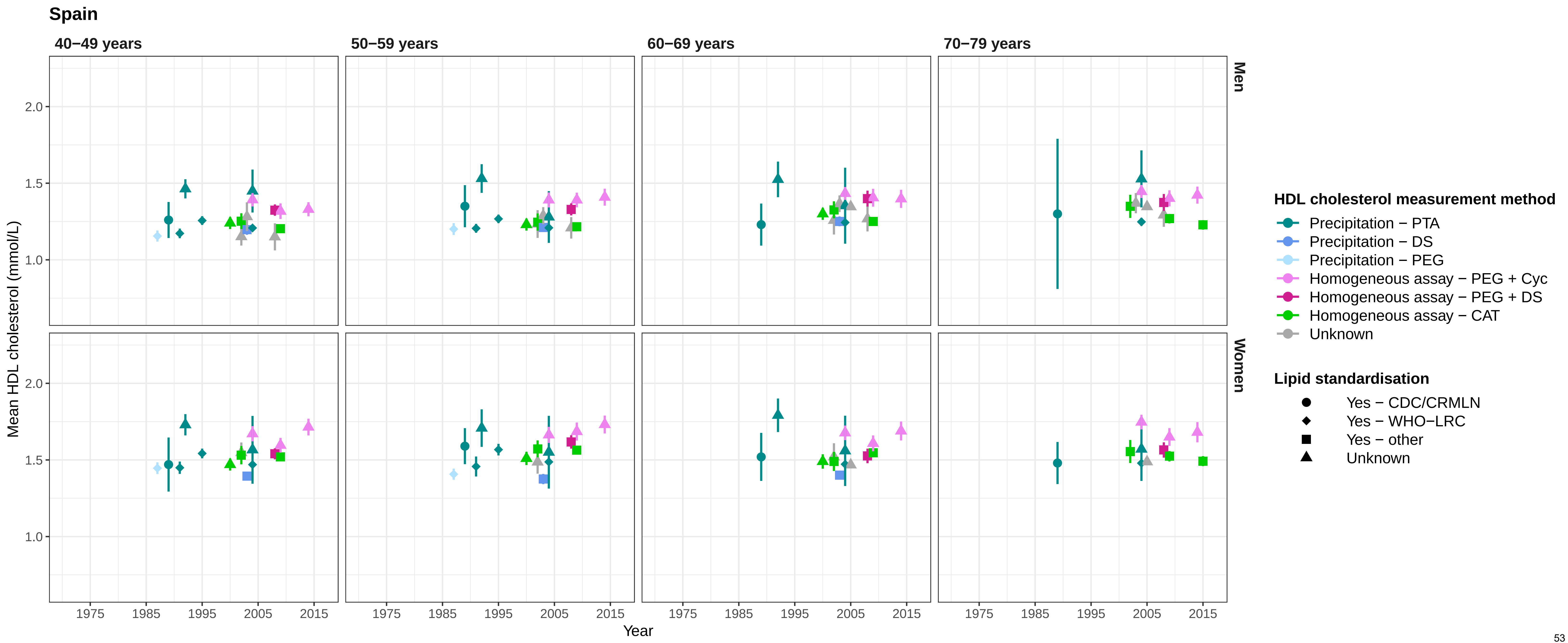

Spain
40−49 years
50−59 years
60−69 years
70−79 years
Men
Women
Mean HDL cholesterol (mmol/L)
1.0
1.5
2.0
1975
1985
1995
2005
2015
Year
HDL cholesterol measurement method
Precipitation − PTA
Precipitation − DS
Precipitation − PEG
Homogeneous assay − PEG + Cyc
Homogeneous assay − PEG + DS
Homogeneous assay − CAT
Unknown
Lipid standardisation
Yes − CDC/CRMLN
Yes − WHO−LRC
Yes − other
Unknown

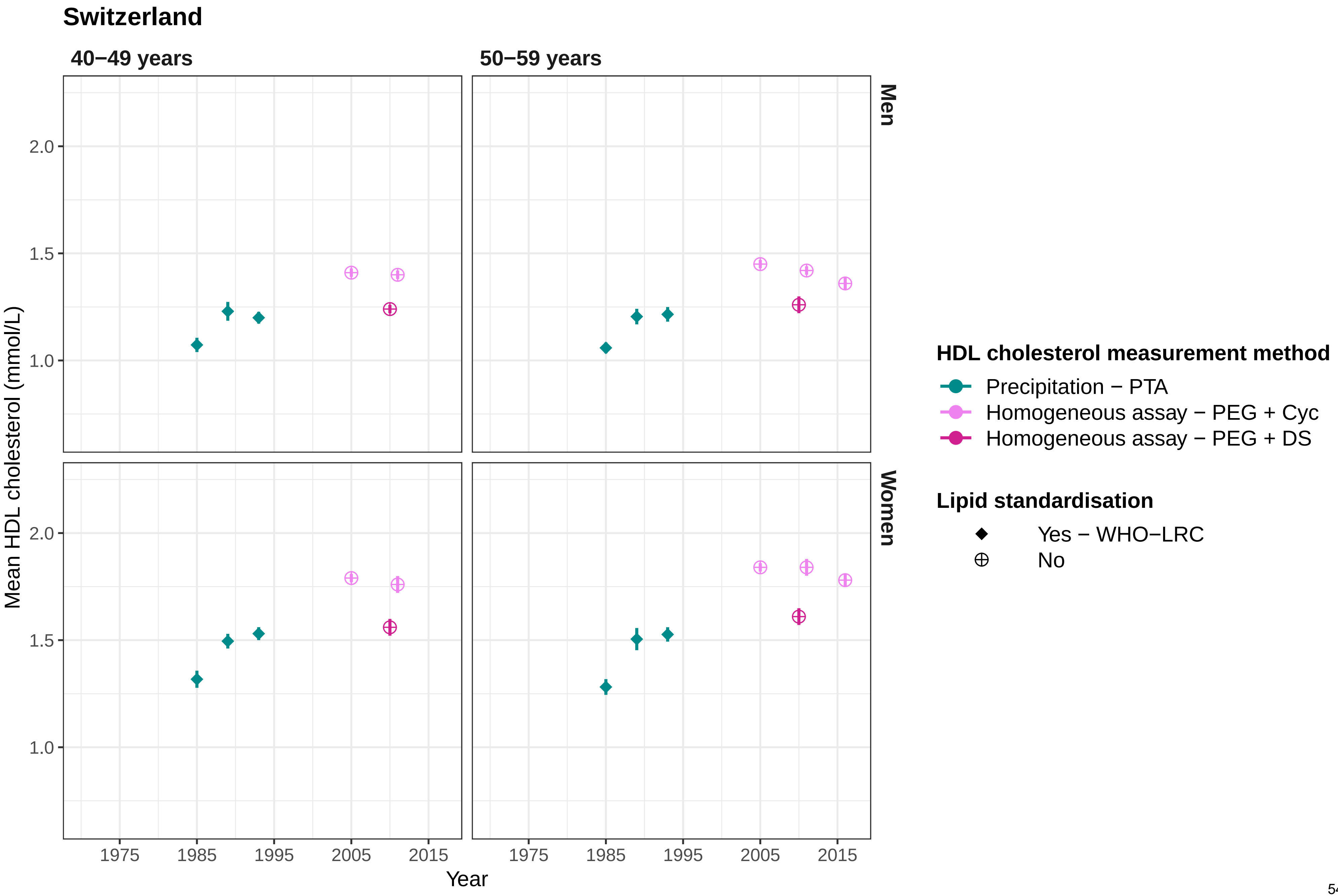
Switzerland
40−49 years
50−59 years
Men
Women
2.0
1.5
1.0
Mean HDL cholesterol (mmol/L)
1975
1985
1995
2005
2015
Year
HDL cholesterol measurement method
Precipitation − PTA
Homogeneous assay − PEG + Cyc
Homogeneous assay − PEG + DS
Lipid standardisation
Yes − WHO−LRC
No

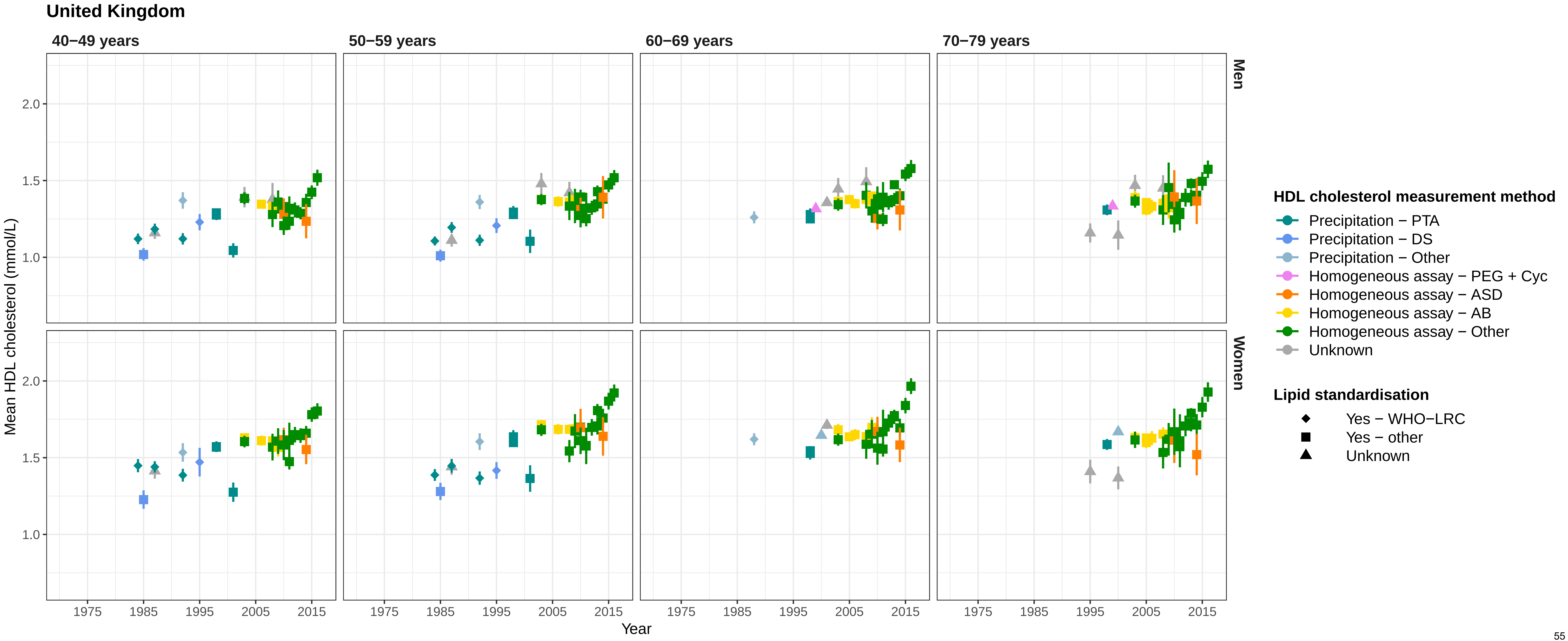

United Kingdom
40−49 years
50−59 years
60−69 years
70−79 years
Men
Women
Mean HDL cholesterol (mmol/L)
1.0
1.5
2.0
1975
1985
1995
2005
2015
Year
HDL cholesterol measurement method
Precipitation − PTA
Precipitation − DS
Precipitation − Other
Homogeneous assay − PEG + Cyc
Homogeneous assay − ASD
Homogeneous assay − AB
Homogeneous assay − Other
Unknown
Lipid standardisation
Yes − WHO−LRC
Yes − other
Unknown

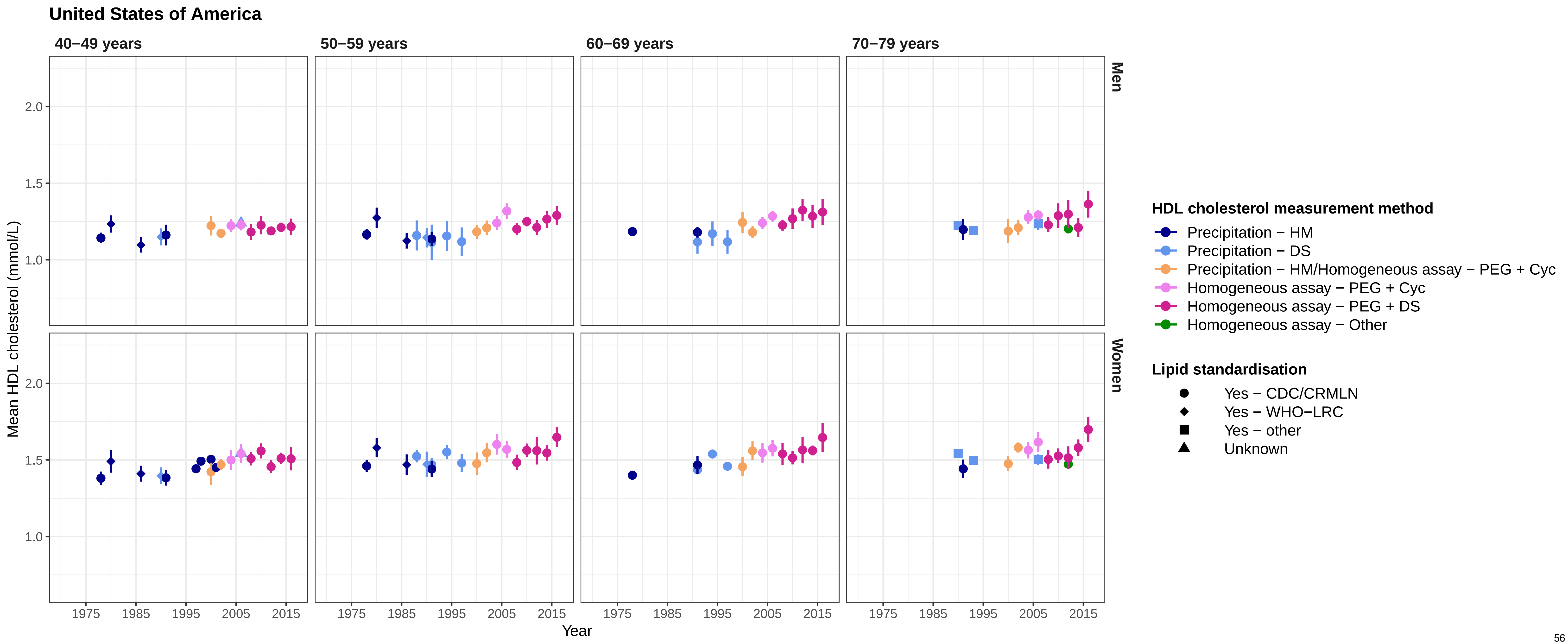

United States of America
40−49 years
50−59 years
60−69 years
70−79 years
Men
Women
Mean HDL cholesterol (mmol/L)
2.0
1.5
1.0
1975
1985
1995
2005
2015
Year
HDL cholesterol measurement method
Precipitation − HM
Precipitation − DS
Precipitation − HM/Homogeneous assay − PEG + Cyc
Homogeneous assay − PEG + Cyc
Homogeneous assay − PEG + DS
Homogeneous assay − Other
Lipid standardisation
Yes − CDC/CRMLN
Yes − WHO−LRC
Yes − other
Unknown

Supplementary Figure 3: Linear and nonlinear (LOESS) regression fits by country, sex and age group.

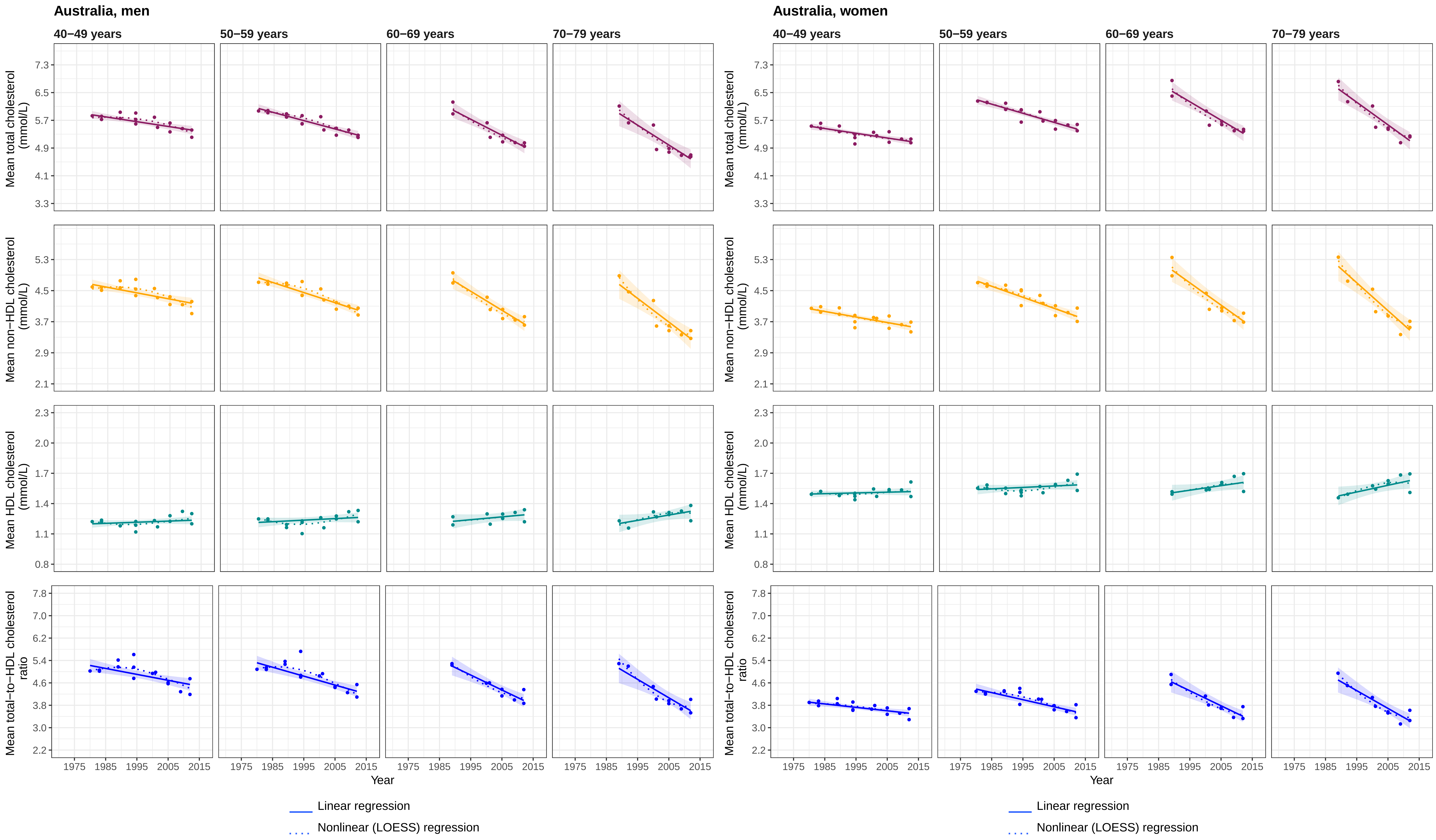
Australia, men
Australia, women
40–49 years
50–59 years
60–69 years
70–79 years
Mean total cholesterol (mmol/L)
Mean non–HDL cholesterol (mmol/L)
Mean HDL cholesterol (mmol/L)
Mean total–to–HDL cholesterol ratio
Year
Linear regression
Nonlinear (LOESS) regression

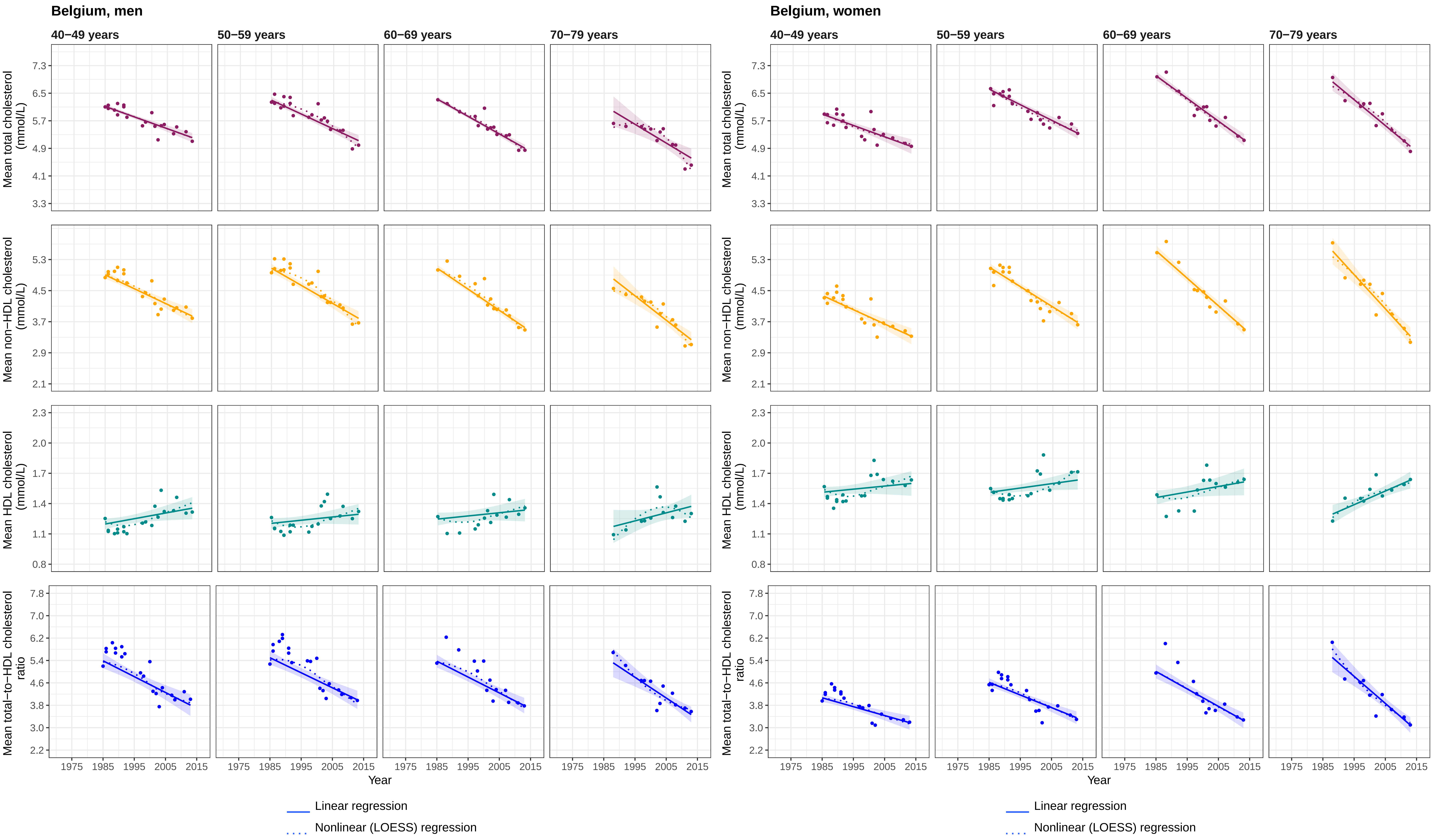
Belgium, men
Belgium, women
40−49 years
50−59 years
60−69 years
70−79 years
Mean total cholesterol (mmol/L)
Mean non−HDL cholesterol (mmol/L)
Mean HDL cholesterol (mmol/L)
Mean total-to-HDL cholesterol ratio
Year
Linear regression
Nonlinear (LOESS) regression

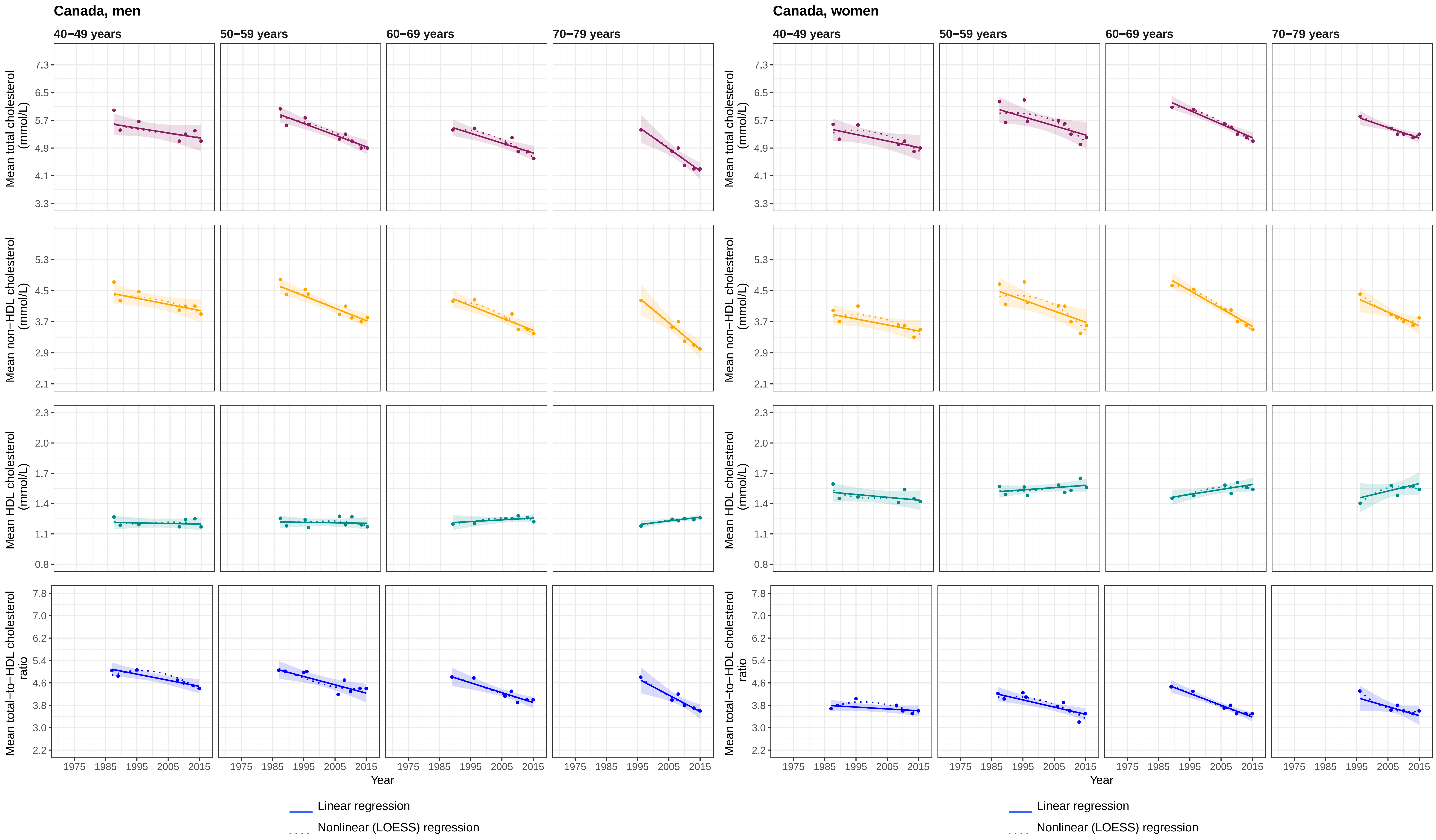

Canada, men
Canada, women
40−49 years
50−59 years
60−69 years
70−79 years
Mean total cholesterol (mmol/L)
Mean non−HDL cholesterol (mmol/L)
Mean HDL cholesterol (mmol/L)
Mean total−to−HDL cholesterol ratio
Year
Linear regression
Nonlinear (LOESS) regression

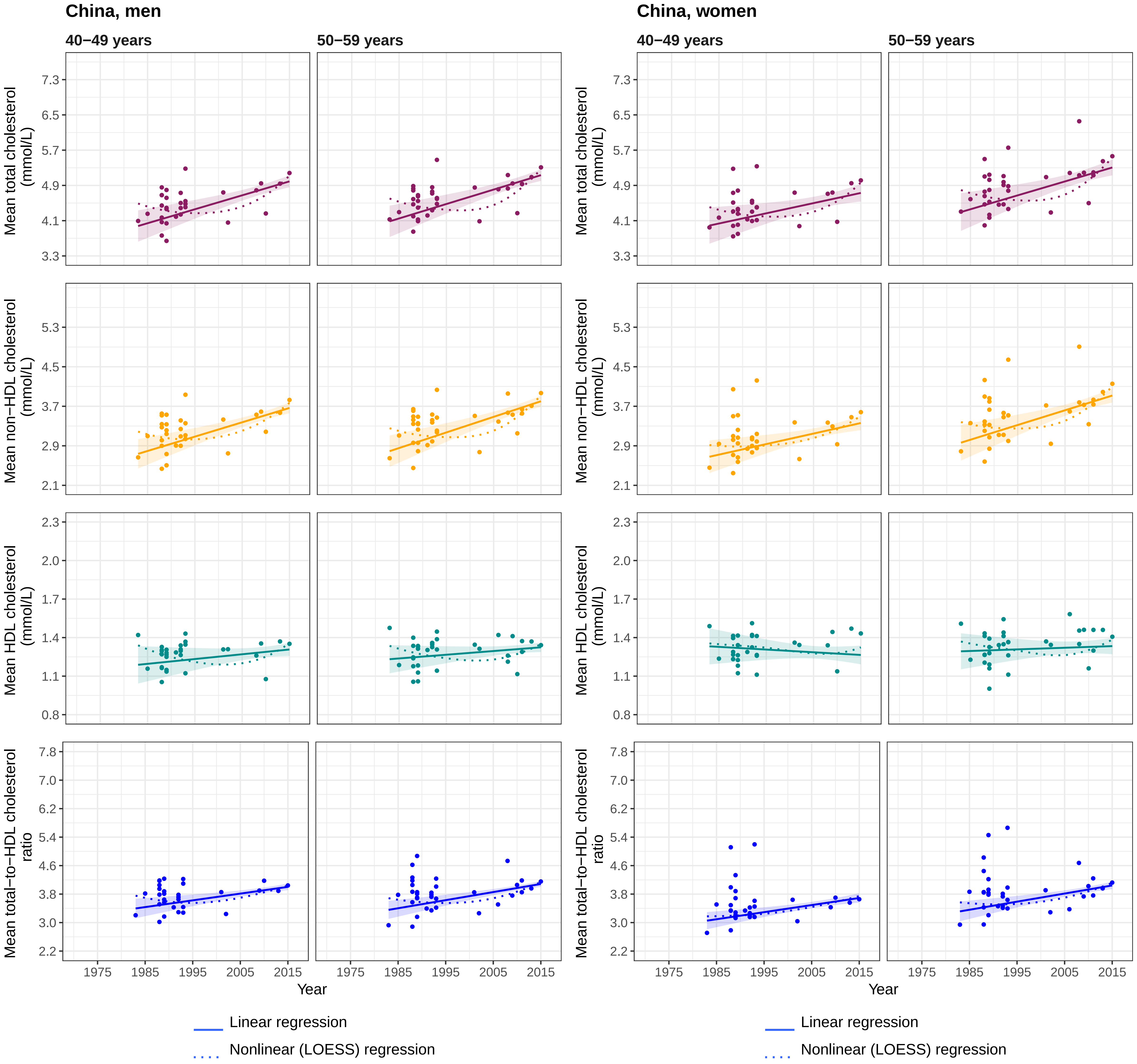

China, men
China, women
40−49 years
50−59 years
Mean total cholesterol (mmol/L)
Mean non−HDL cholesterol (mmol/L)
Mean HDL cholesterol (mmol/L)
Mean total−to−HDL cholesterol ratio
Year
Linear regression
Nonlinear (LOESS) regression

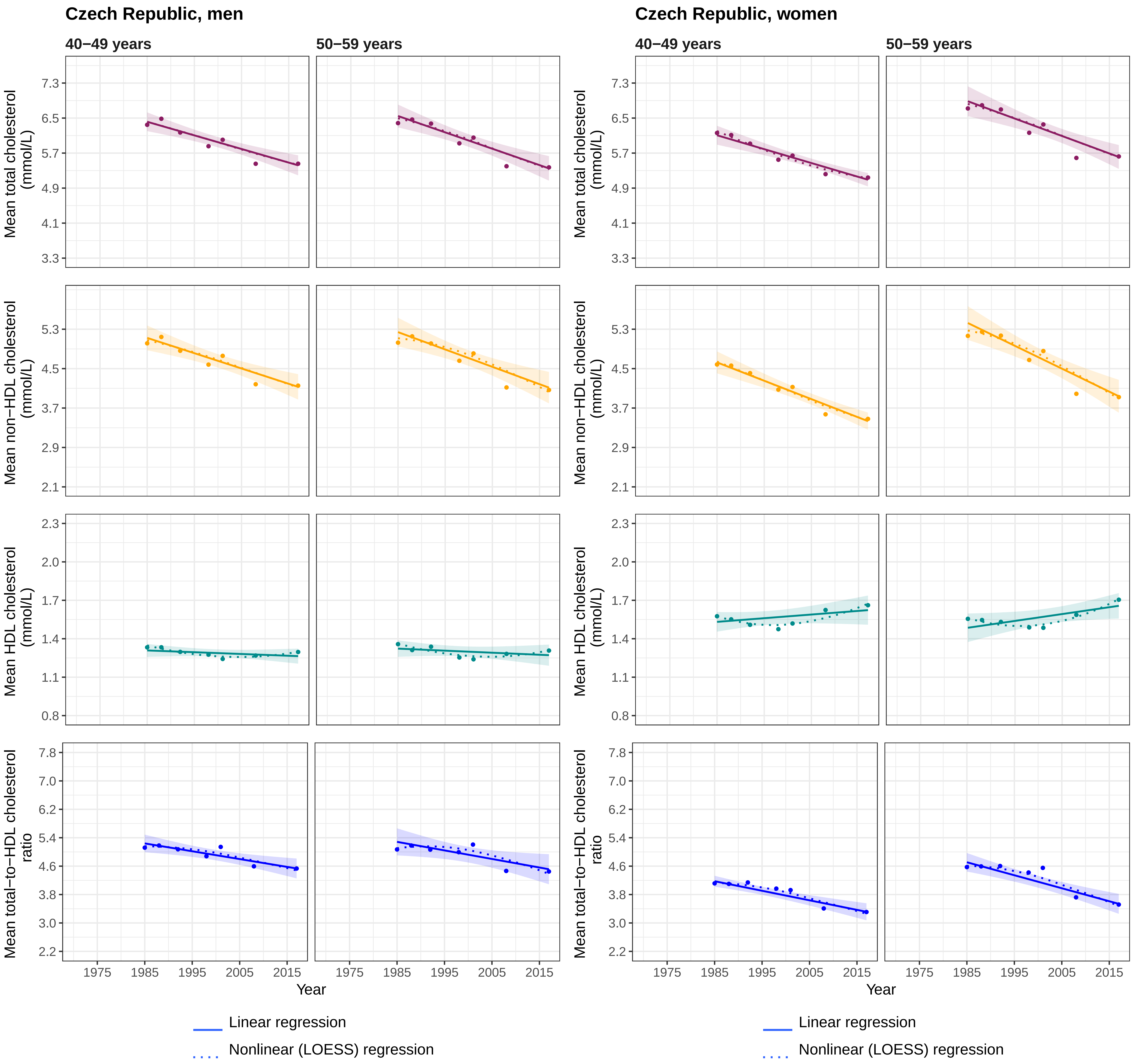

Czech Republic, men
Czech Republic, women
40−49 years
50−59 years
Mean total cholesterol (mmol/L)
Mean non−HDL cholesterol (mmol/L)
Mean HDL cholesterol (mmol/L)
Mean total−to−HDL cholesterol ratio
Year
Linear regression
Nonlinear (LOESS) regression

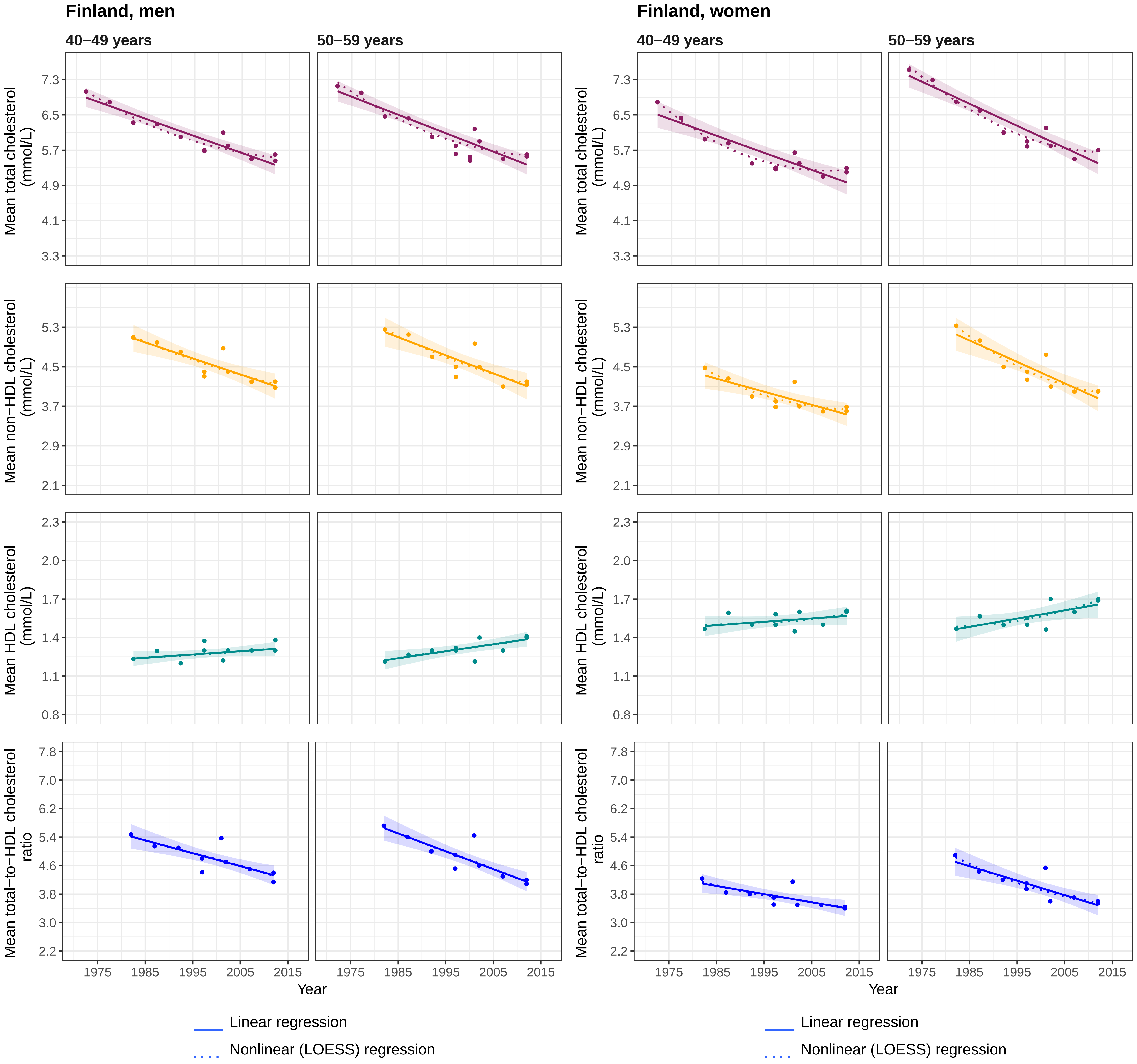

Finland, men
Finland, women
40−49 years
50−59 years
Mean total cholesterol (mmol/L)
Mean non−HDL cholesterol (mmol/L)
Mean HDL cholesterol (mmol/L)
Mean total−to−HDL cholesterol ratio
Year
Linear regression
Nonlinear (LOESS) regression

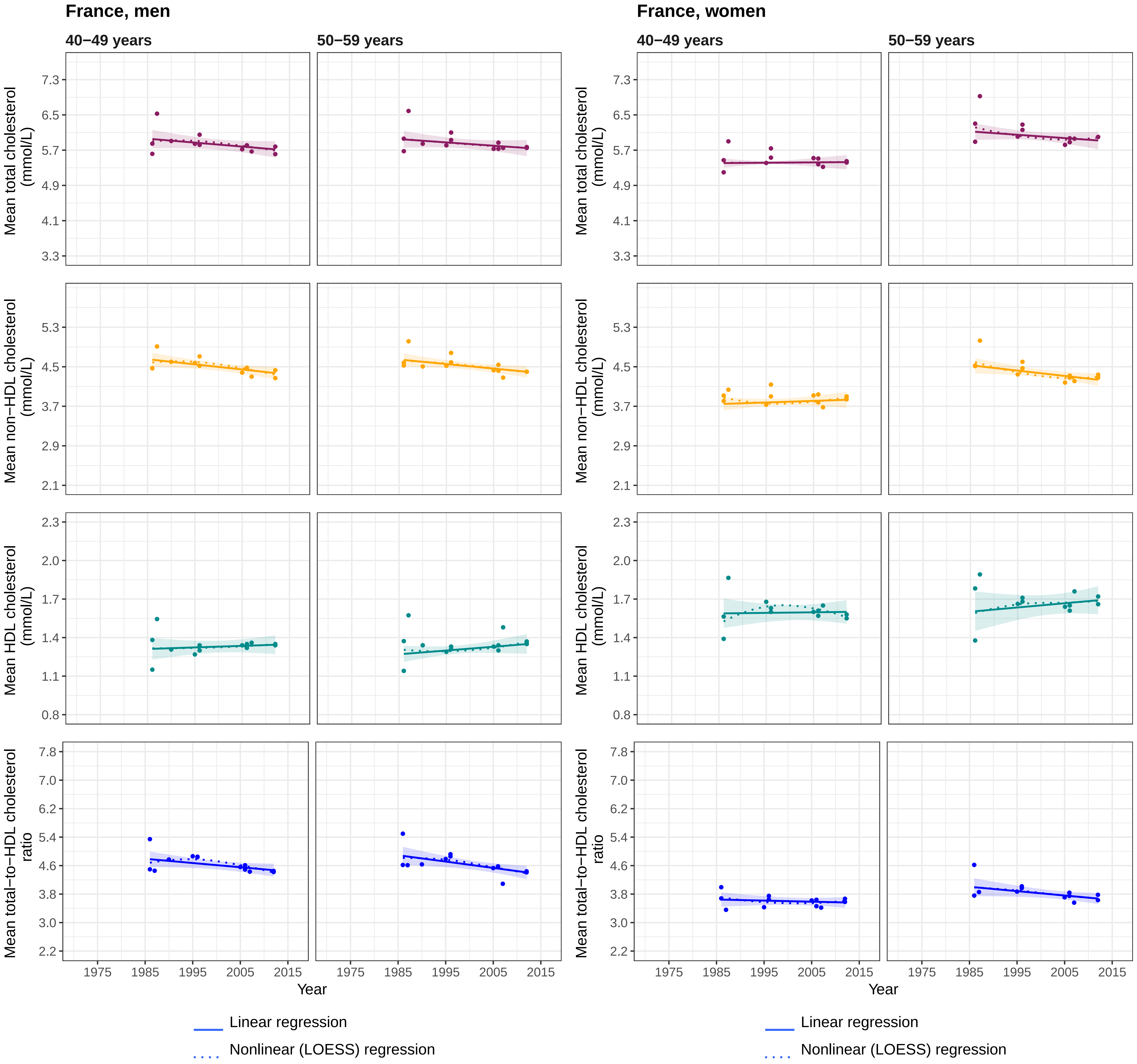
France, men
France, women
40−49 years
50−59 years
Mean total cholesterol (mmol/L)
Mean non−HDL cholesterol (mmol/L)
Mean HDL cholesterol (mmol/L)
Mean total−to−HDL cholesterol ratio
Year
Linear regression
Nonlinear (LOESS) regression

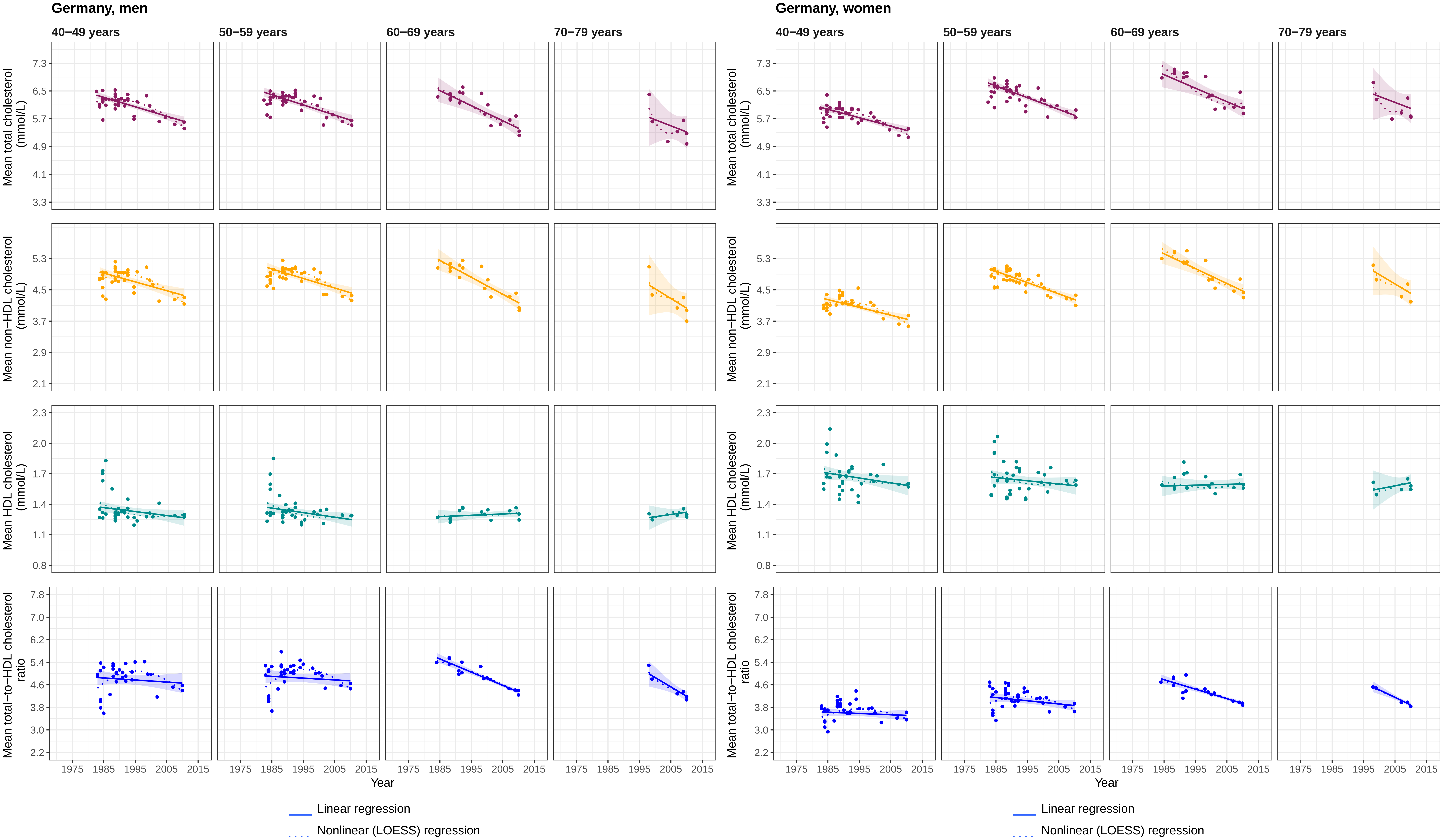

Germany, men
Germany, women
40−49 years
50−59 years
60−69 years
70−79 years
Mean total cholesterol (mmol/L)
Mean non−HDL cholesterol (mmol/L)
Mean HDL cholesterol (mmol/L)
Mean total−to−HDL cholesterol ratio
Year
Linear regression
Nonlinear (LOESS) regression

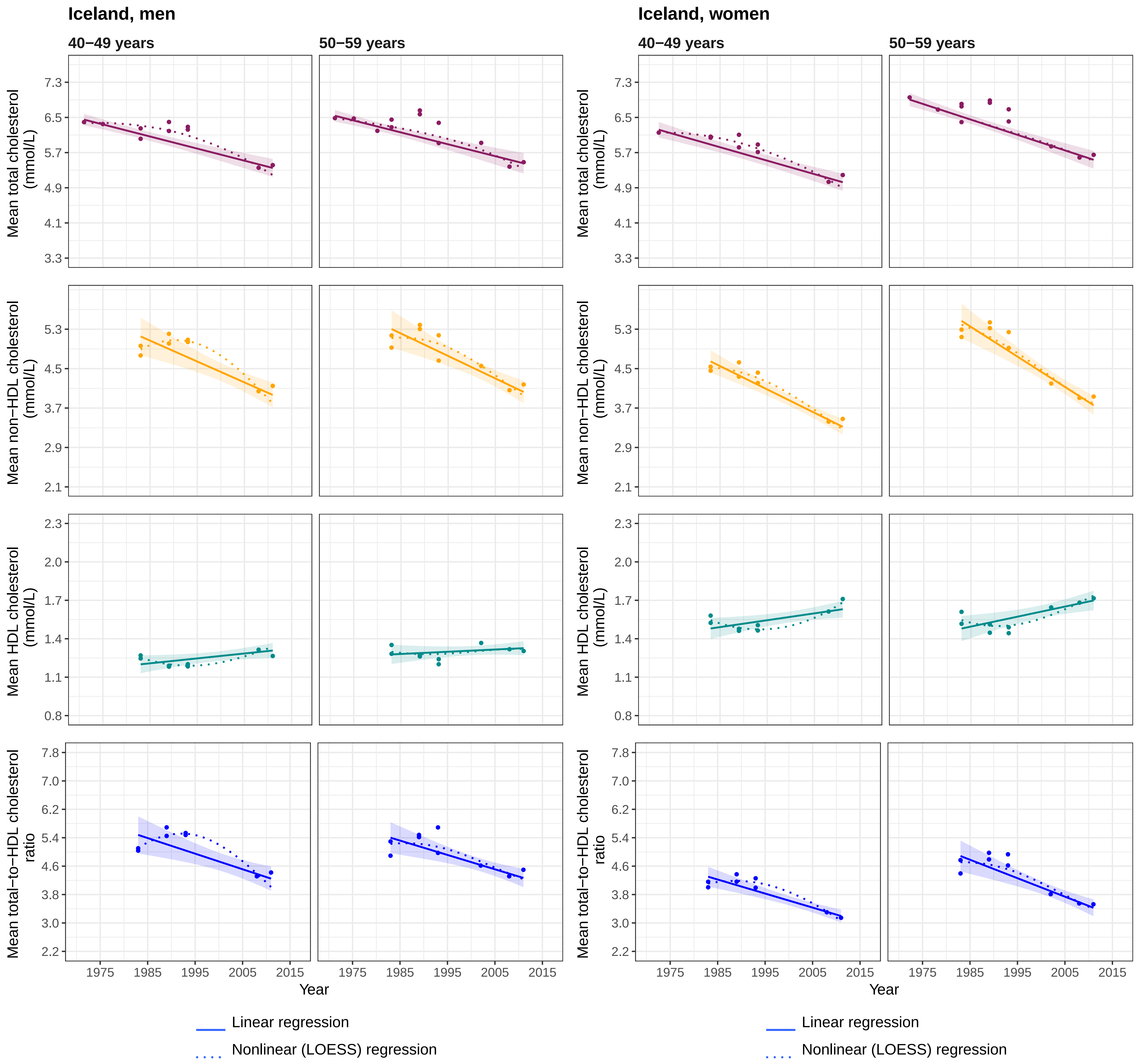

Iceland, men
Iceland, women
40−49 years
50−59 years
Mean total cholesterol (mmol/L)
Mean non−HDL cholesterol (mmol/L)
Mean HDL cholesterol (mmol/L)
Mean total−to−HDL cholesterol ratio
Year
Linear regression
Nonlinear (LOESS) regression

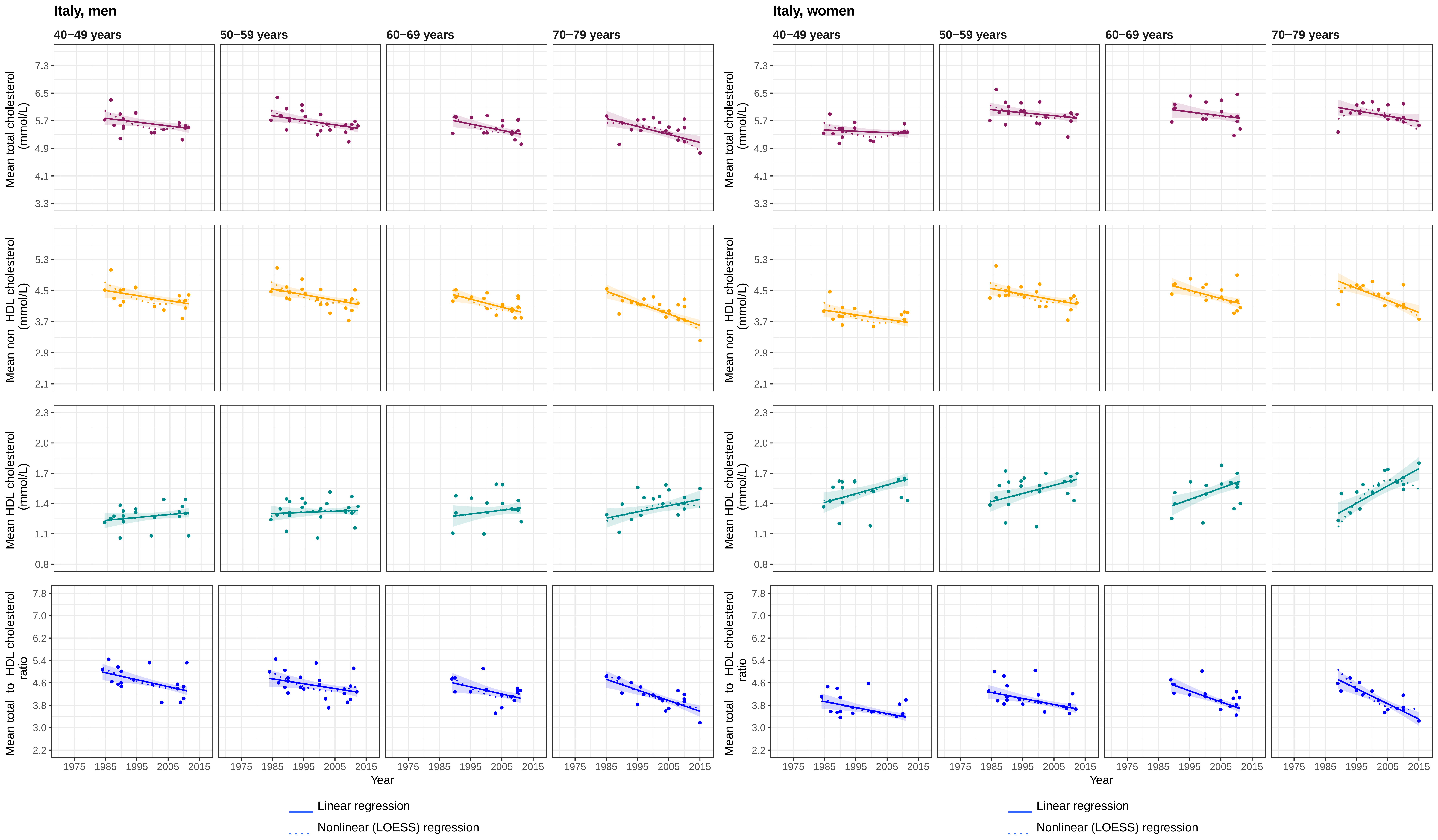
Italy, men
Italy, women
40−49 years
50−59 years
60−69 years
70−79 years
Mean total cholesterol (mmol/L)
Mean non−HDL cholesterol (mmol/L)
Mean HDL cholesterol (mmol/L)
Mean total−to−HDL cholesterol ratio
Year
Linear regression
Nonlinear (LOESS) regression

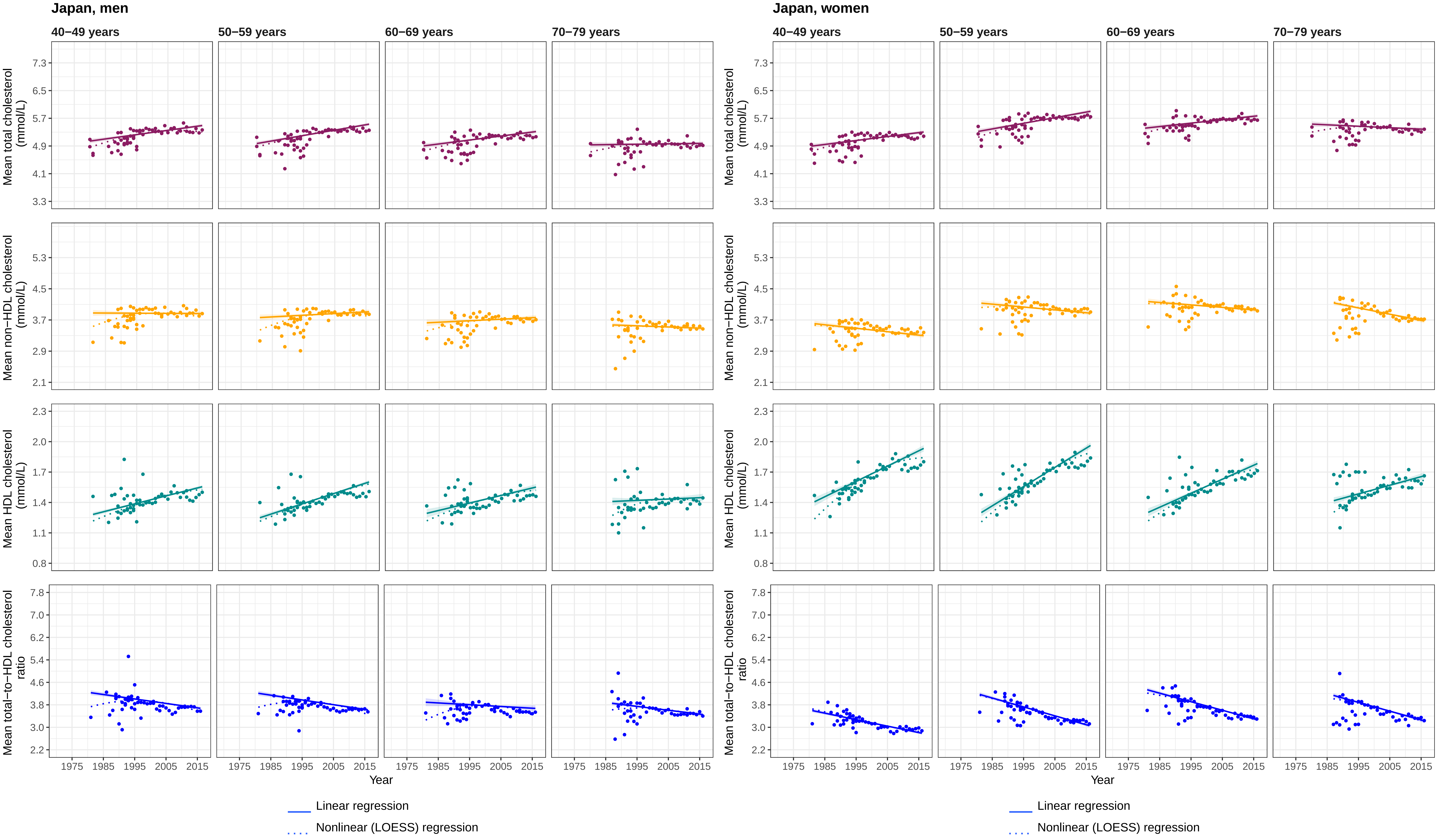

Japan, men
Japan, women
40−49 years
50−59 years
60−69 years
70−79 years
Mean total cholesterol (mmol/L)
Mean non−HDL cholesterol (mmol/L)
Mean HDL cholesterol (mmol/L)
Mean total−to−HDL cholesterol ratio
Year
Linear regression
Nonlinear (LOESS) regression

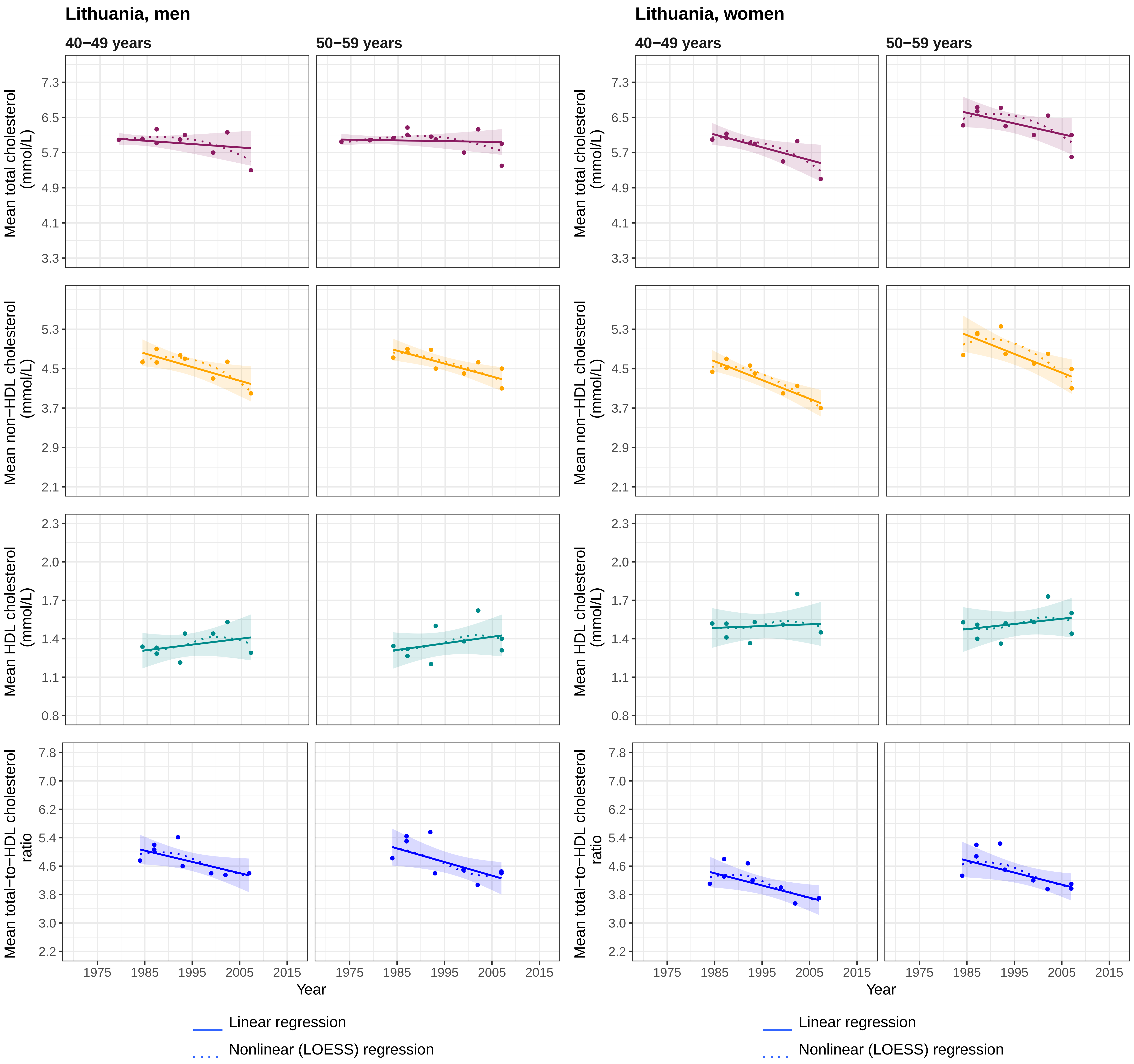

Lithuania, men
Lithuania, women
40−49 years
50−59 years
Mean total cholesterol (mmol/L)
7.3
6.5
5.7
4.9
4.1
3.3
Mean non−HDL cholesterol (mmol/L)
5.3
4.5
3.7
2.9
2.1
Mean HDL cholesterol (mmol/L)
2.3
2.0
1.7
1.4
1.1
0.8
Mean total−to−HDL cholesterol ratio
7.8
7.0
6.2
5.4
4.6
3.8
3.0
2.2
1975
1985
1995
2005
2015
Year
Linear regression
Nonlinear (LOESS) regression

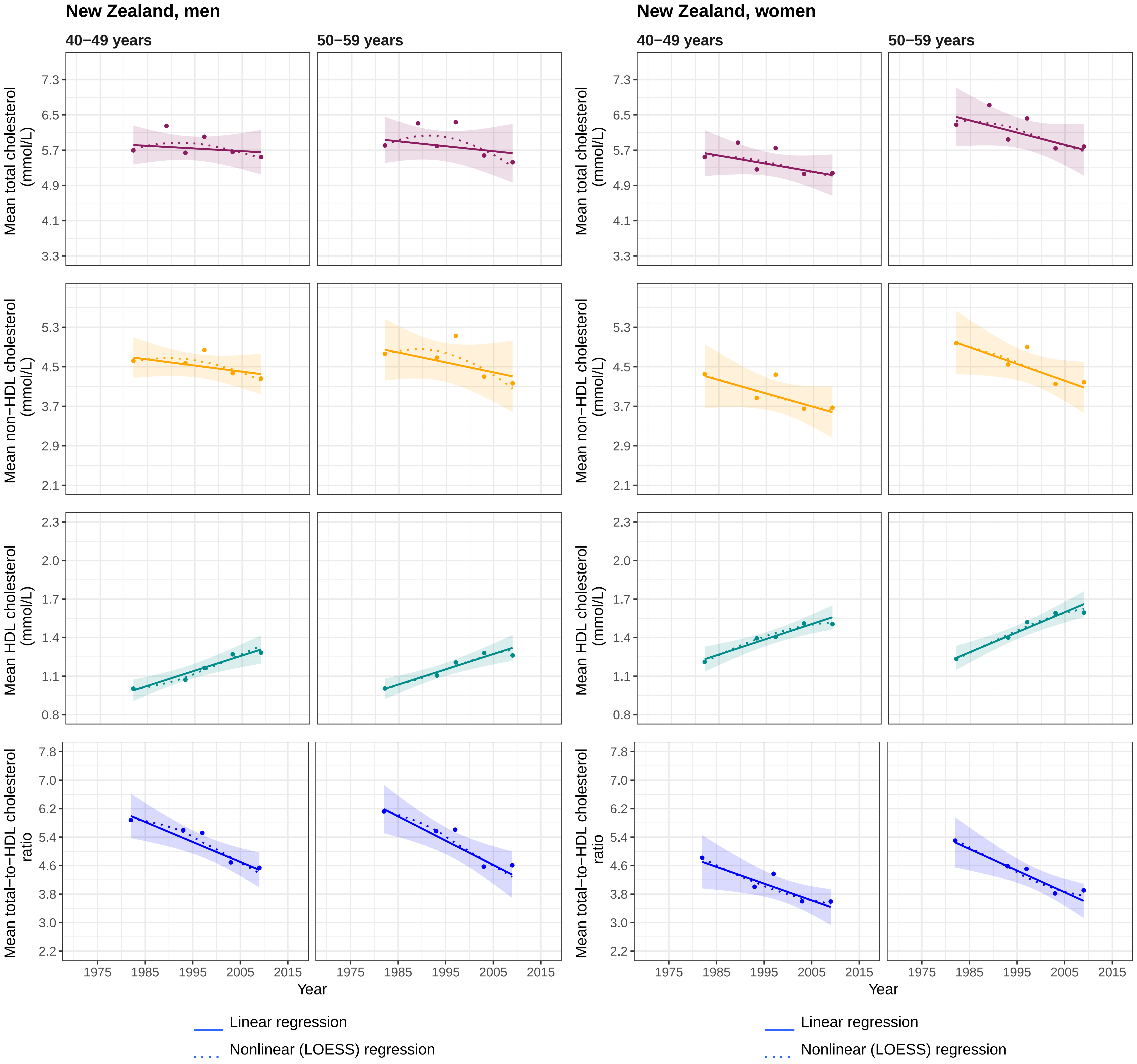

New Zealand, men
New Zealand, women
40−49 years
50−59 years
40−49 years
50−59 years
Mean total cholesterol (mmol/L)
7.3
6.5
5.7
4.9
4.1
3.3
Mean non−HDL cholesterol (mmol/L)
5.3
4.5
3.7
2.9
2.1
Mean HDL cholesterol (mmol/L)
2.3
2.0
1.7
1.4
1.1
0.8
Mean total−to−HDL cholesterol ratio
7.8
7.0
6.2
5.4
4.6
3.8
3.0
2.2
1975
1985
1995
2005
2015
Year
Linear regression
Nonlinear (LOESS) regression

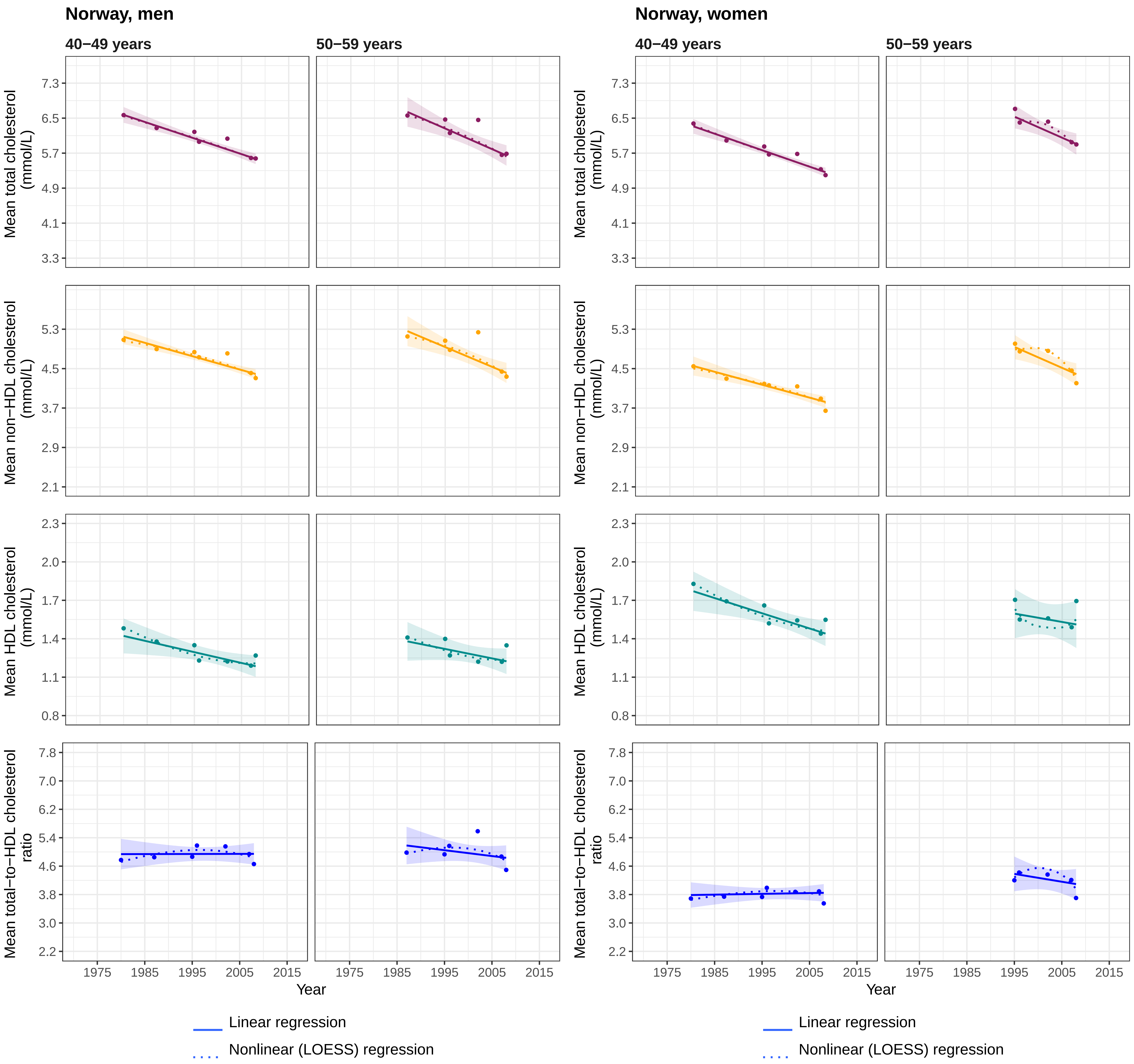

Norway, men
Norway, women
40−49 years
50−59 years
40−49 years
50−59 years
Mean total cholesterol (mmol/L)
Mean non−HDL cholesterol (mmol/L)
Mean HDL cholesterol (mmol/L)
Mean total−to−HDL cholesterol ratio
Year
Linear regression
Nonlinear (LOESS) regression

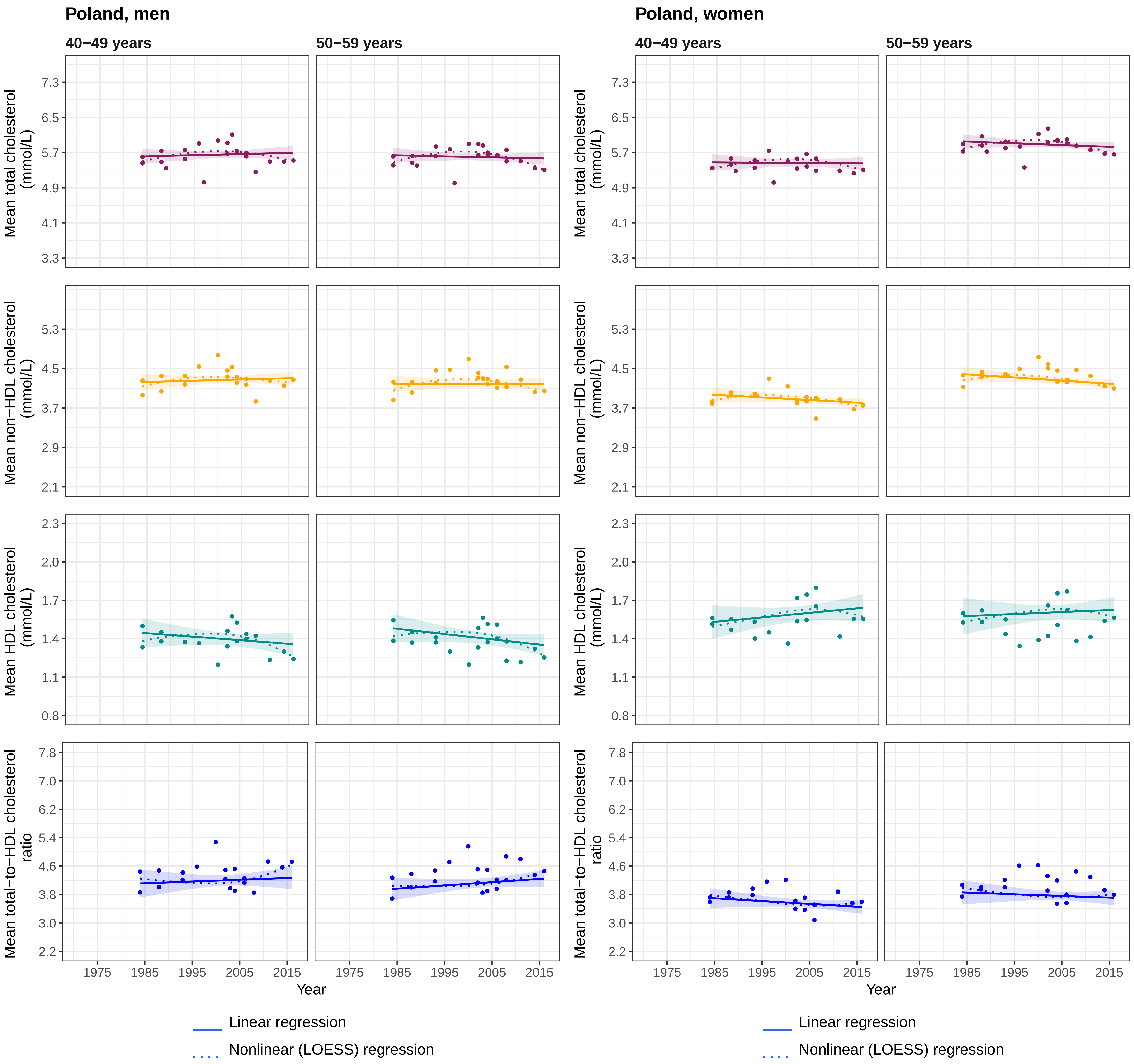
Poland, men
Poland, women
40−49 years
50−59 years
40−49 years
50−59 years
Mean total cholesterol (mmol/L)
Mean non−HDL cholesterol (mmol/L)
Mean HDL cholesterol (mmol/L)
Mean total−to−HDL cholesterol ratio
Year
Linear regression
Nonlinear (LOESS) regression
Linear regression
Nonlinear (LOESS) regression

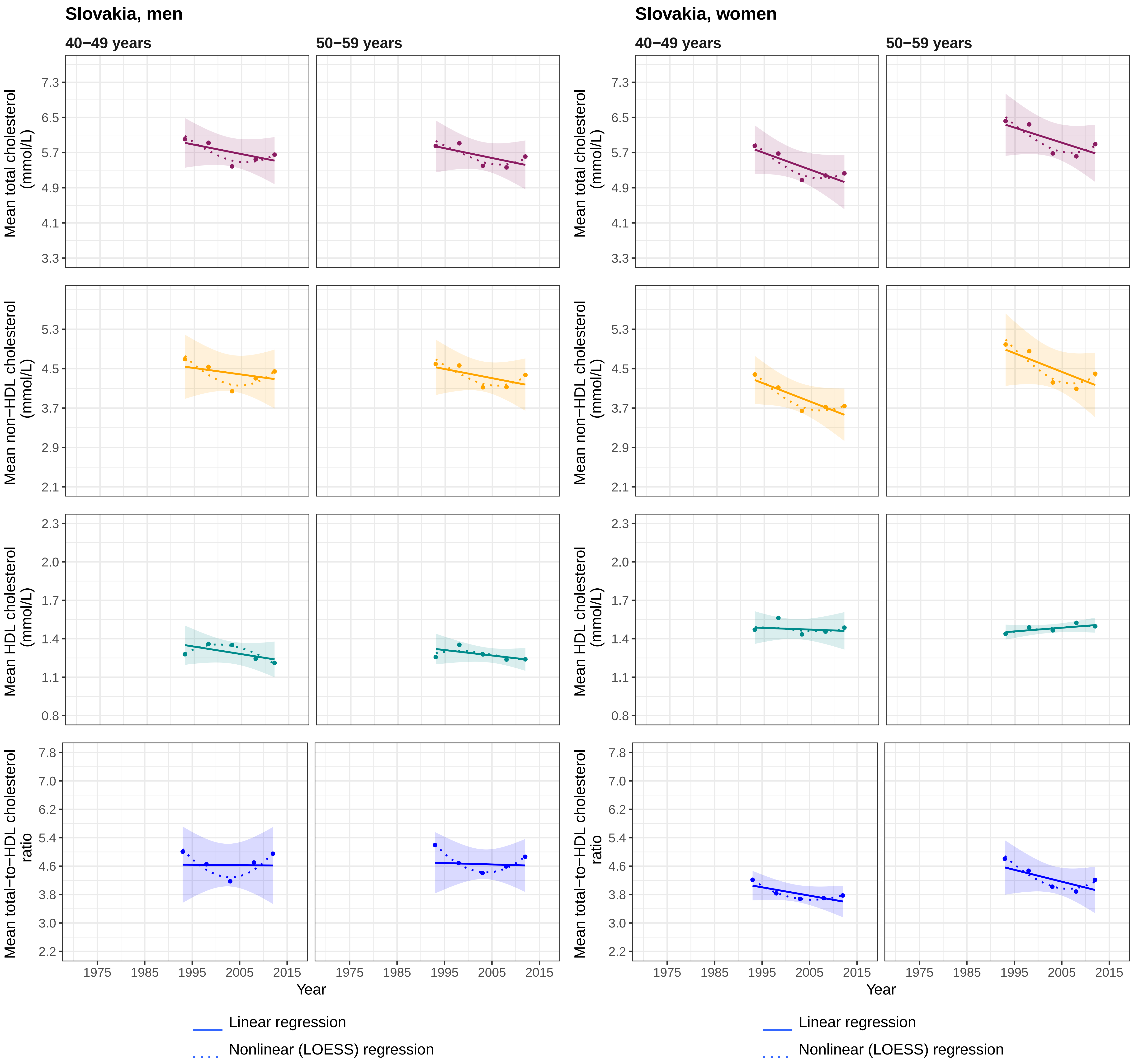

Slovakia, men
Slovakia, women
40−49 years
50−59 years
Mean total cholesterol (mmol/L)
Mean non−HDL cholesterol (mmol/L)
Mean HDL cholesterol (mmol/L)
Mean total−to−HDL cholesterol ratio
Year
Linear regression
Nonlinear (LOESS) regression

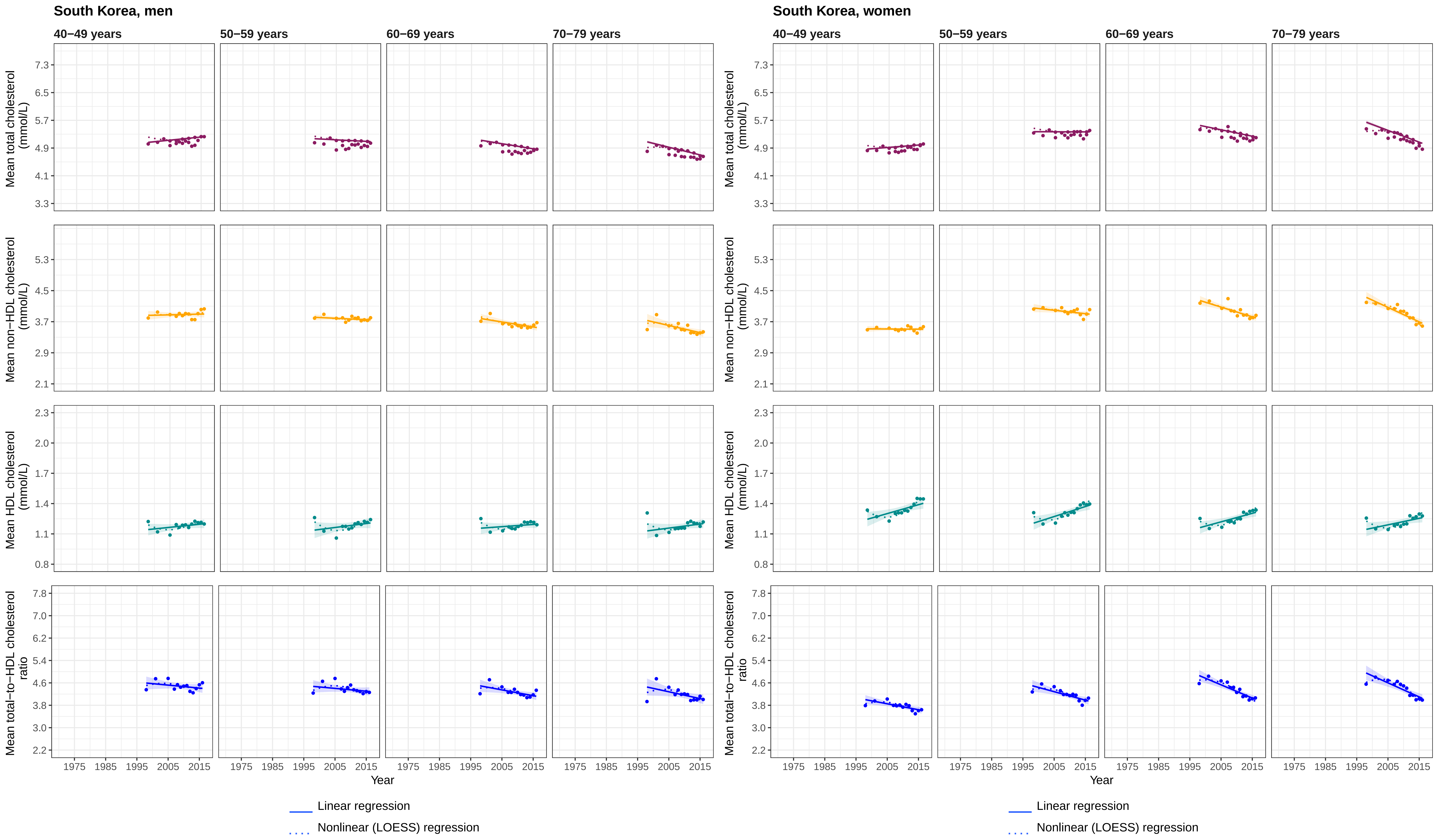

South Korea, men
South Korea, women
40−49 years
50−59 years
60−69 years
70−79 years
Mean total cholesterol (mmol/L)
Mean non−HDL cholesterol (mmol/L)
Mean HDL cholesterol (mmol/L)
Mean total-to-HDL cholesterol ratio
Year
Linear regression
Nonlinear (LOESS) regression

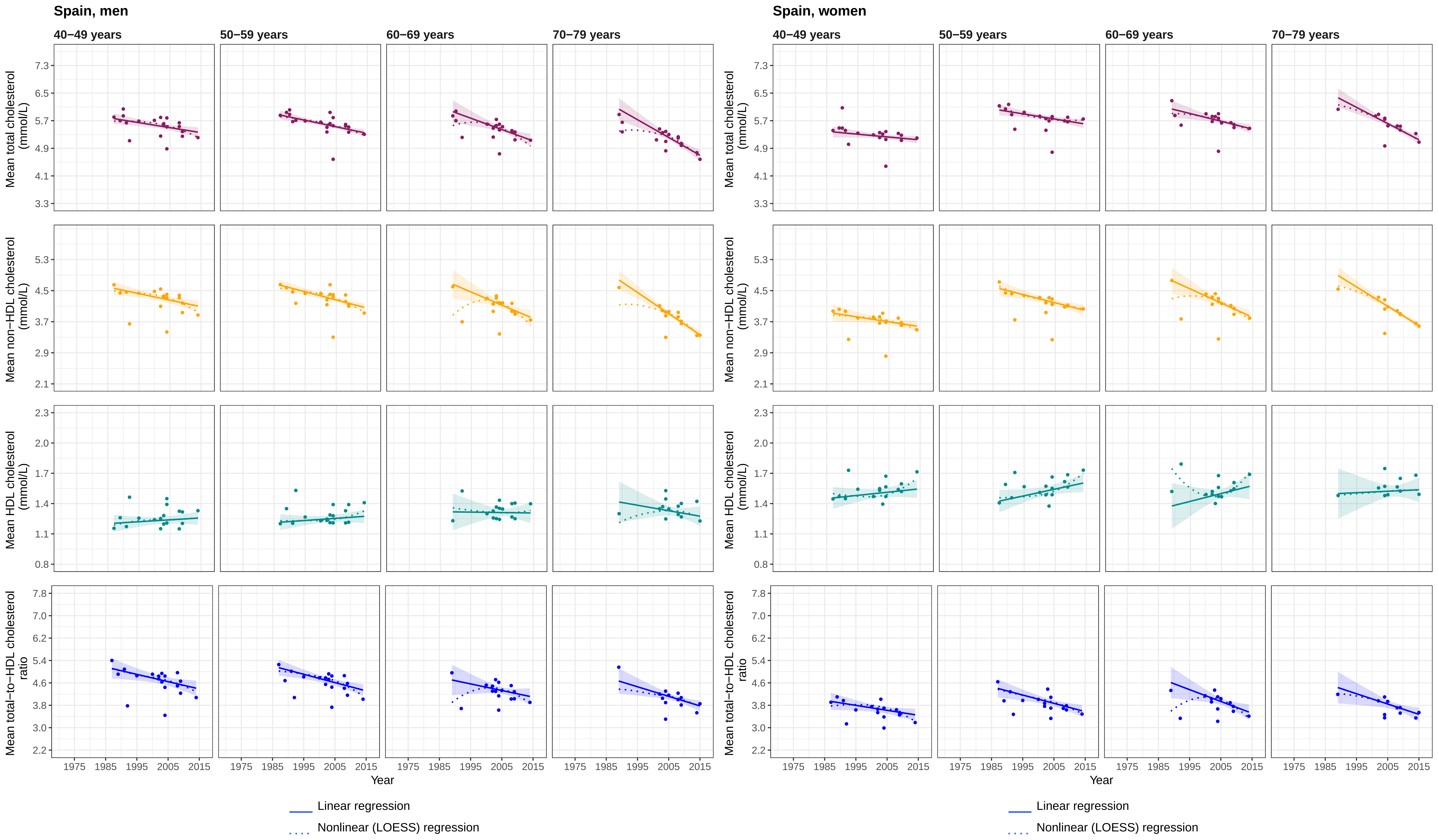

Spain, men
Spain, women
40–49 years
50–59 years
60–69 years
70–79 years
Mean total cholesterol (mmol/L)
Mean non−HDL cholesterol (mmol/L)
Mean HDL cholesterol (mmol/L)
Mean total−to−HDL cholesterol ratio
Year
Linear regression
Nonlinear (LOESS) regression

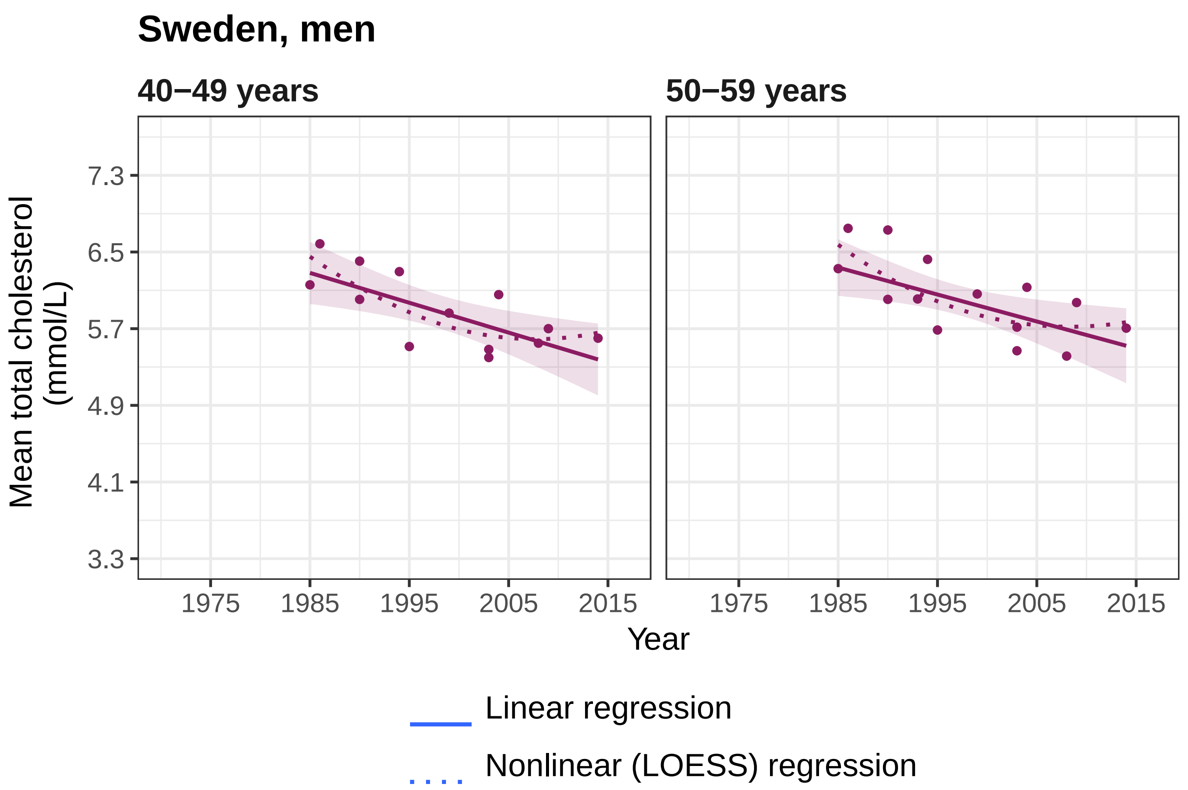
Sweden, men
40−49 years
50−59 years
Mean total cholesterol (mmol/L)
7.3
6.5
5.7
4.9
4.1
3.3
1975
1985
1995
2005
2015
Year
Linear regression
Nonlinear (LOESS) regression

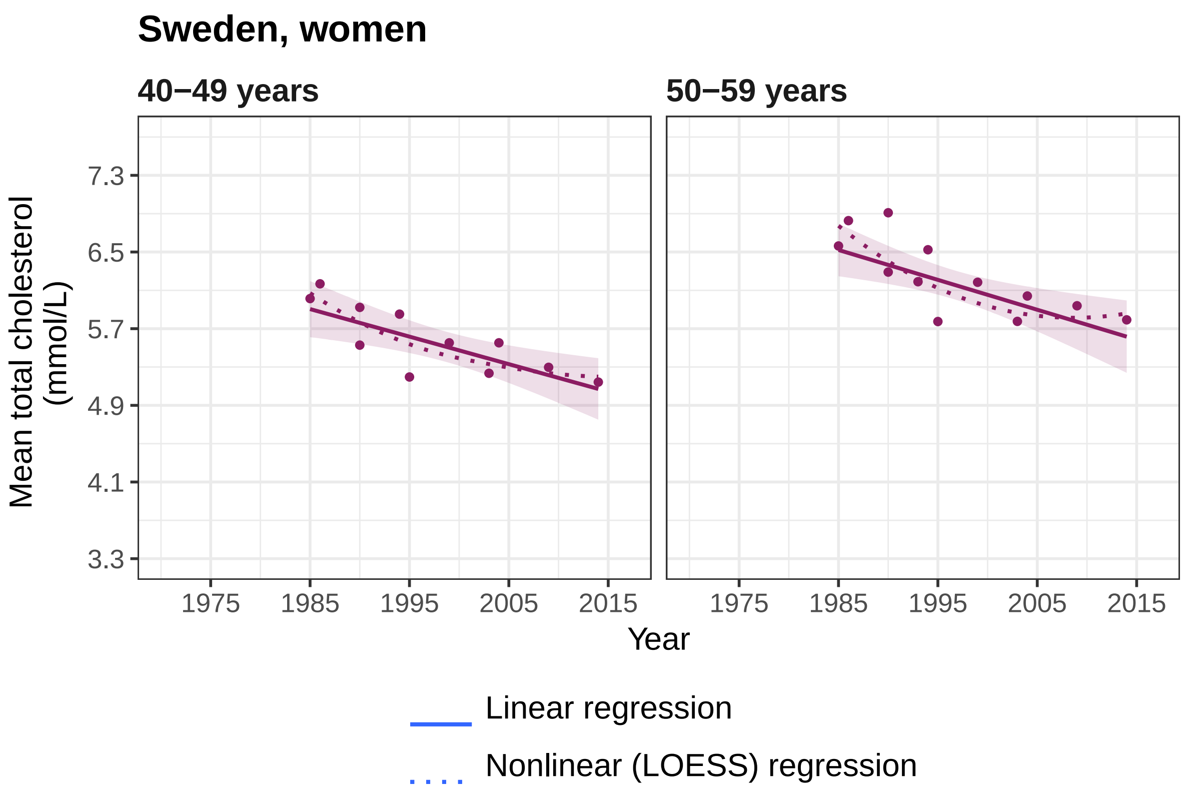
Sweden, women
40−49 years
50−59 years
Mean total cholesterol (mmol/L)
7.3
6.5
5.7
4.9
4.1
3.3
1975
1985
1995
2005
2015
Year
Linear regression
Nonlinear (LOESS) regression

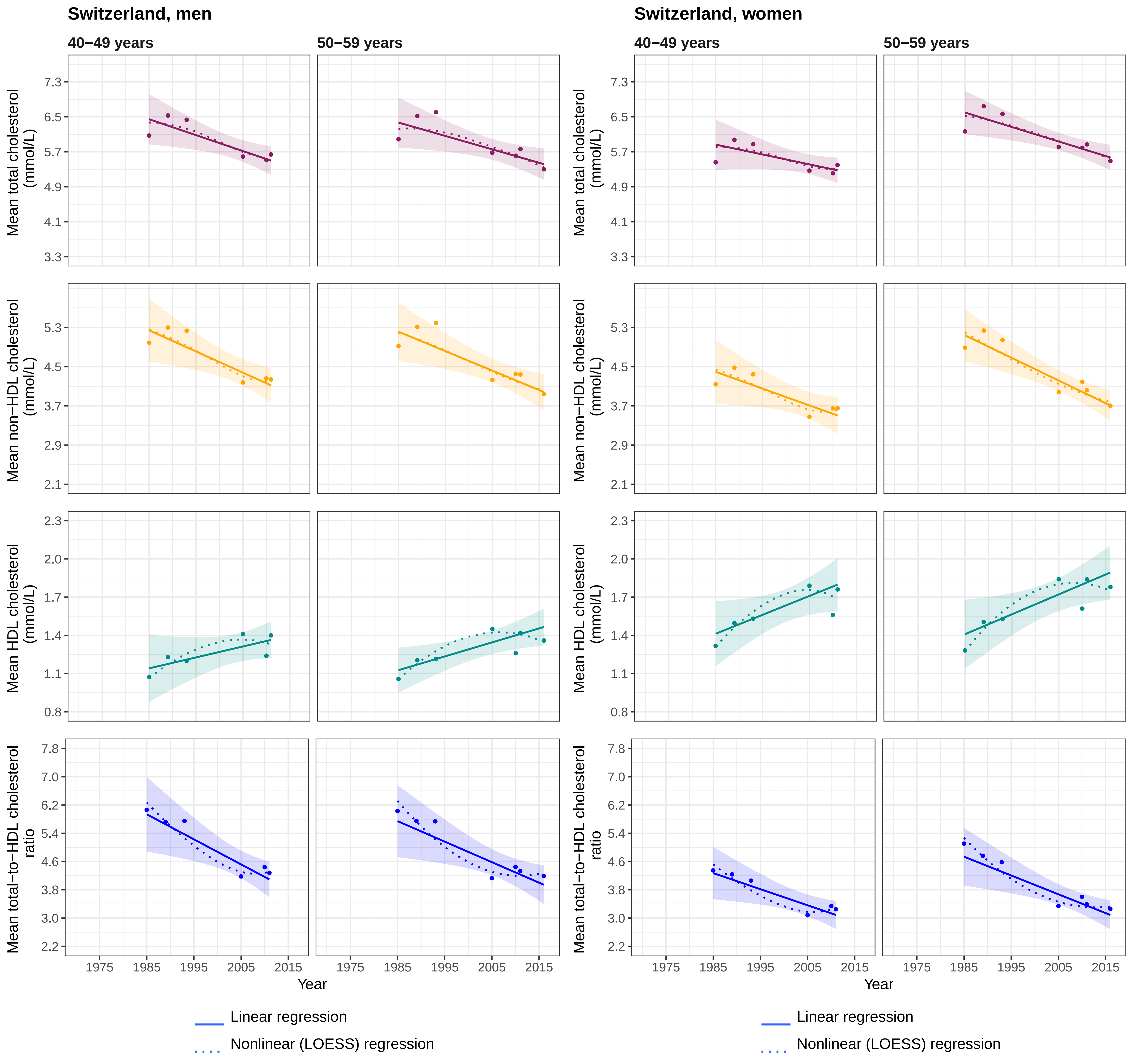

Switzerland, men
Switzerland, women
40−49 years
50−59 years
Mean total cholesterol (mmol/L)
Mean non−HDL cholesterol (mmol/L)
Mean HDL cholesterol (mmol/L)
Mean total−to−HDL cholesterol ratio
Year
Linear regression
Nonlinear (LOESS) regression

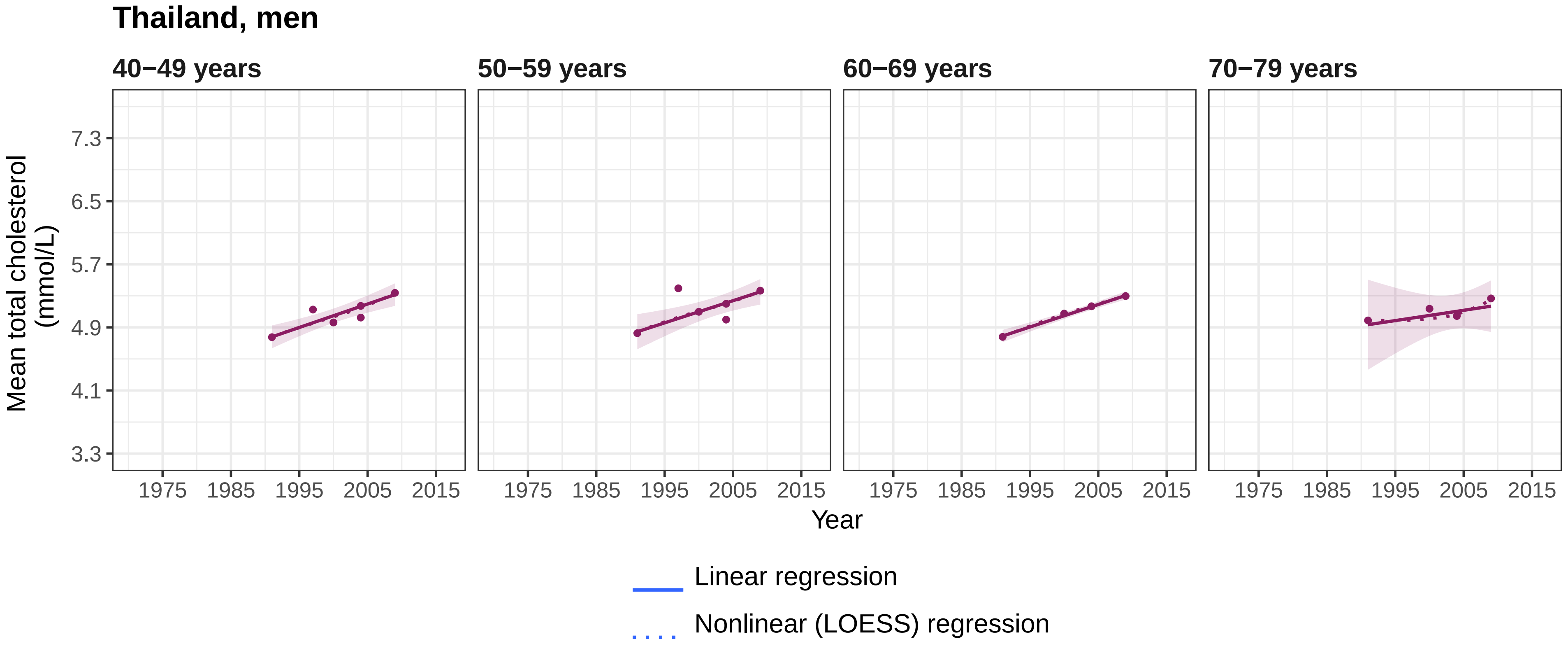

Thailand, men
40–49 years
50–59 years
60–69 years
70–79 years
Mean total cholesterol (mmol/L)
7.3
6.5
5.7
4.9
4.1
3.3
1975
1985
1995
2005
2015
Year
Linear regression
Nonlinear (LOESS) regression

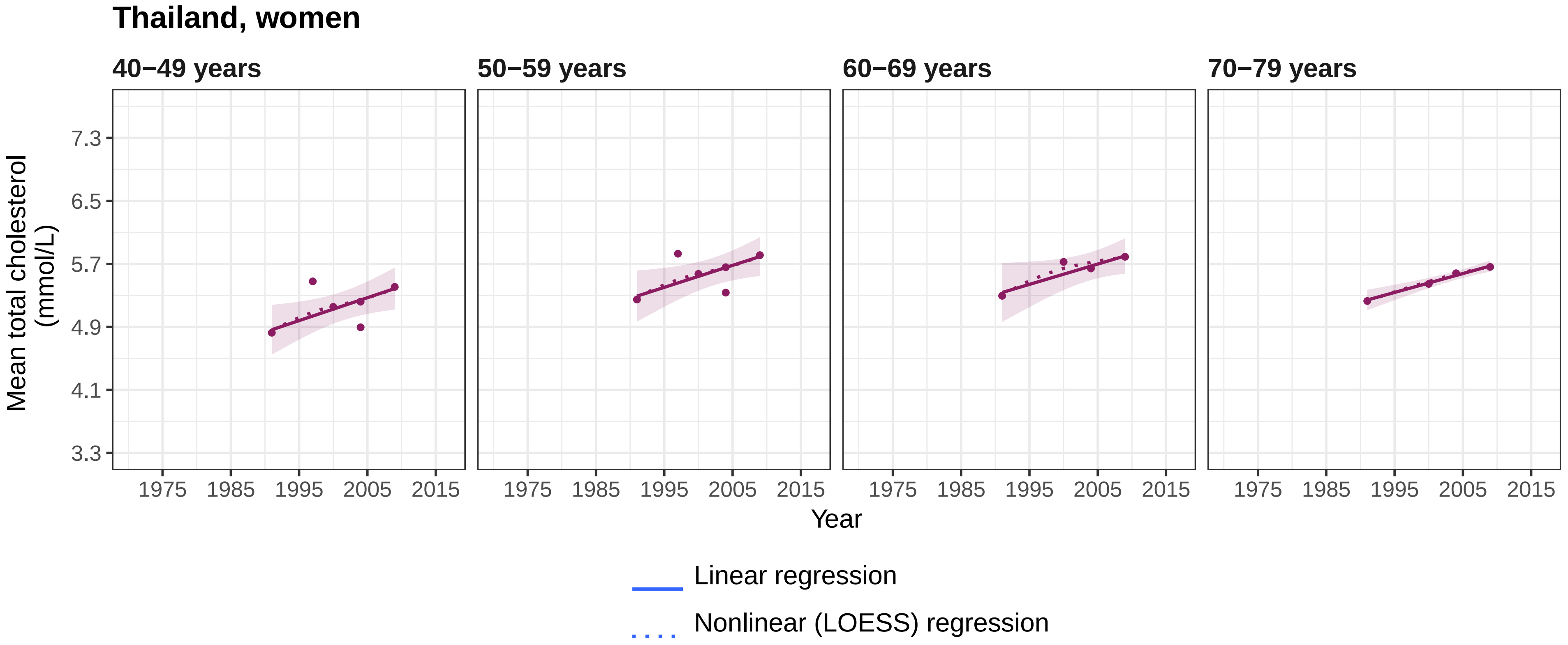

Thailand, women
40–49 years
50–59 years
60–69 years
70–79 years
Mean total cholesterol (mmol/L)
7.3
6.5
5.7
4.9
4.1
3.3
1975
1985
1995
2005
2015
Year
Linear regression
Nonlinear (LOESS) regression

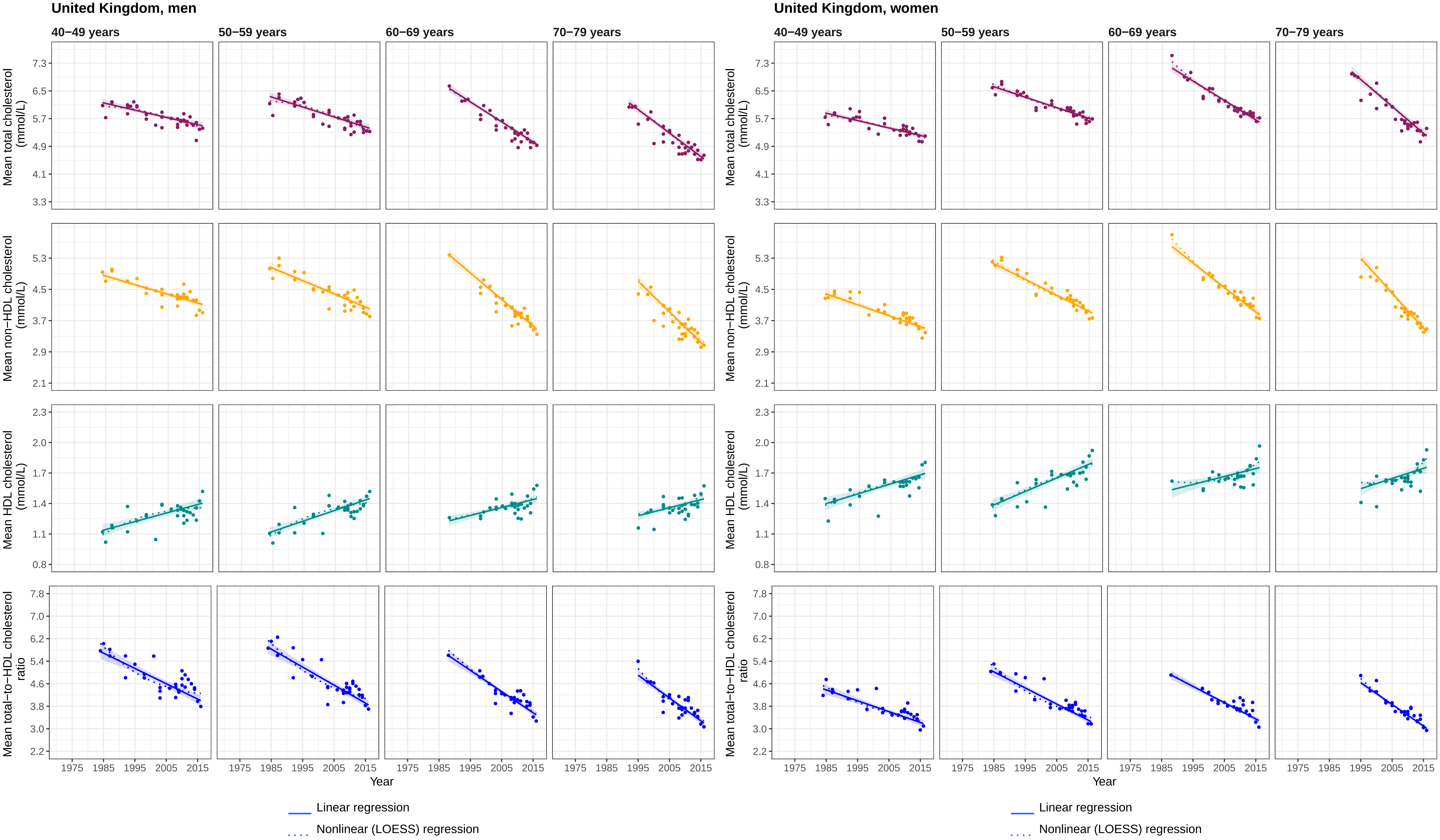
United Kingdom, men
United Kingdom, women
40−49 years
50−59 years
60−69 years
70−79 years
Mean total cholesterol (mmol/L)
Mean non−HDL cholesterol (mmol/L)
Mean HDL cholesterol (mmol/L)
Mean total−to−HDL cholesterol ratio
Year
Linear regression
Nonlinear (LOESS) regression

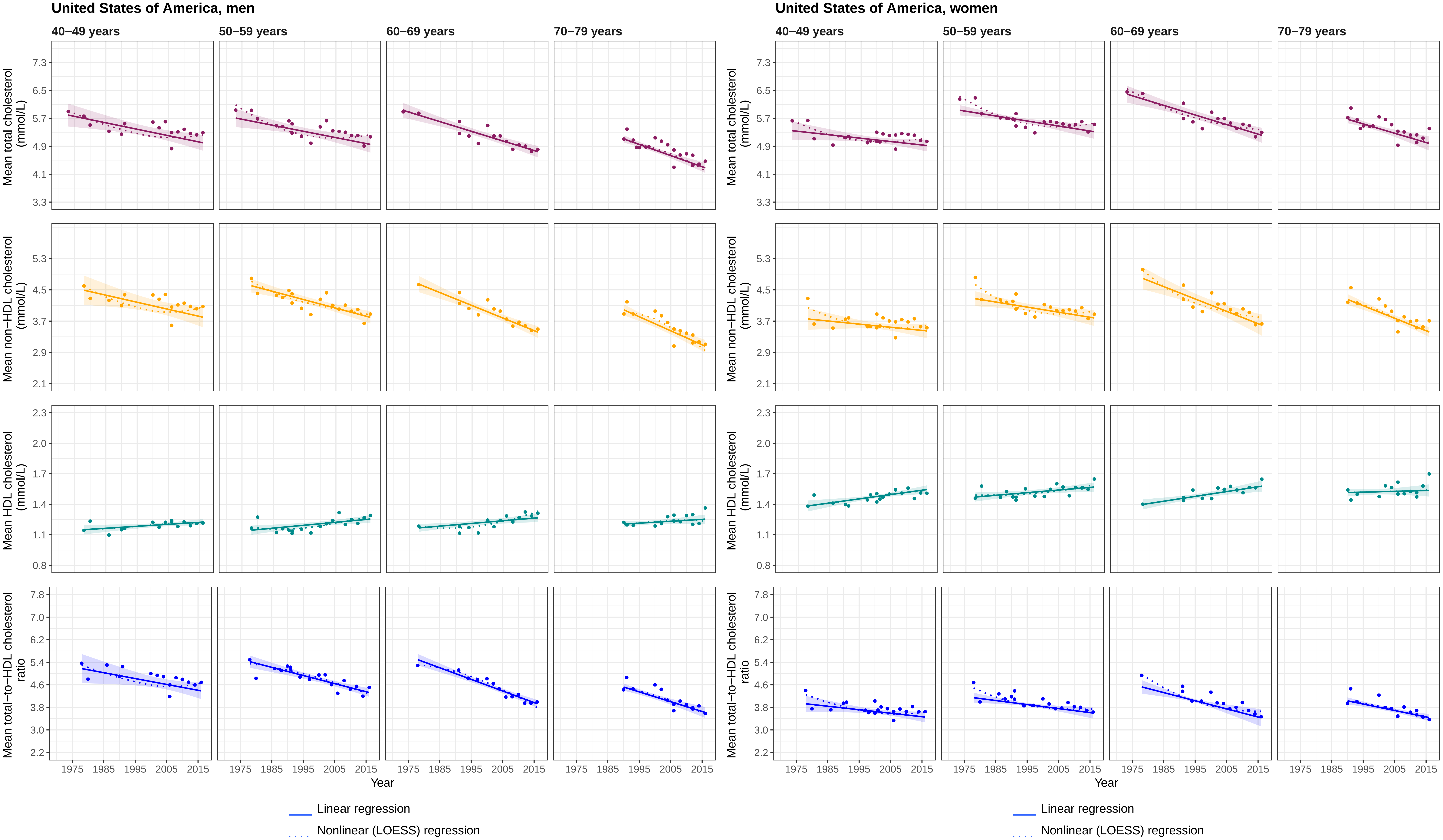

United States of America, men
United States of America, women
40−49 years
50−59 years
60−69 years
70−79 years
Mean total cholesterol (mmol/L)
Mean non−HDL cholesterol (mmol/L)
Mean HDL cholesterol (mmol/L)
Mean total−to−HDL cholesterol ratio
Year
Linear regression
Nonlinear (LOESS) regression

Supplementary Figure 4: Mean total cholesterol at the beginning and end of analysis period by country and sex in people aged 40 to 59 years. The dark lines show the results for the period of data availability for each country (Supplementary Table 2); the lighter segments extend the trends to the period from 1980 to 2015 so that the start and end years are comparable across countries.

[*] Finland, Iceland, Lithuania, Norway and USA had data prior to 1980 and Czech Republic, Japan, Poland, South Korea, Switzerland, UK and USA after 2015. This figure shows results from 1980 to 2015 so that the start and end years are comparable across countries.

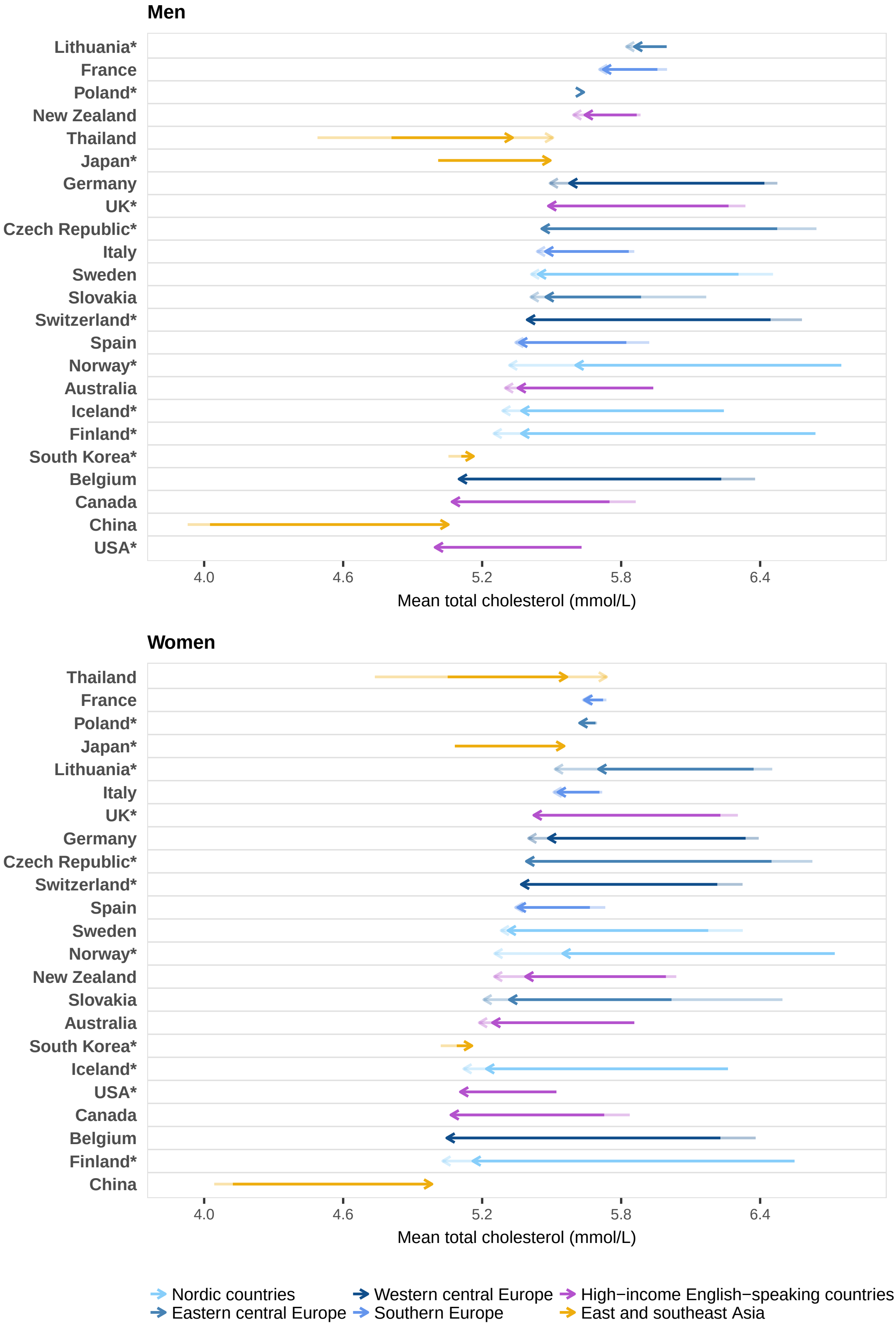

Men
Lithuania*
France
Poland*
New Zealand
Thailand
Japan*
Germany
UK*
Czech Republic*
Italy
Sweden
Slovakia
Switzerland*
Spain
Norway*
Australia
Iceland*
Finland*
South Korea*
Belgium
Canada
China
USA*
4.0
4.6
5.2
5.8
6.4
Mean total cholesterol (mmol/L)
Women
Thailand
France
Poland*
Japan*
Lithuania*
Italy
UK*
Germany
Czech Republic*
Switzerland*
Spain
Sweden
Norway*
New Zealand
Slovakia
Australia
South Korea*
Iceland*
USA*
Canada
Belgium
Finland*
China
4.0
4.6
5.2
5.8
6.4
Mean total cholesterol (mmol/L)
Nordic countries
Eastern central Europe
Western central Europe
Southern Europe
High−income English−speaking countries
East and southeast Asia

Supplementary Figure 5: Mean (A) non-HDL and (B) HDL cholesterol at the beginning and end of analysis period by country and sex in people aged 40 to 59 years. The dark lines show the results for the period of data availability for each country (Supplementary Table 2); the lighter segments extend the trends to the period from 1980 to 2015 so that the start and end years are comparable across countries.

* Norway and USA had data prior to 1980 and Czech Republic, Japan, Poland, South Korea, Switzerland, UK and USA after 2015. This figure shows results from 1980 to 2015 so that the start and end years are comparable across countries.

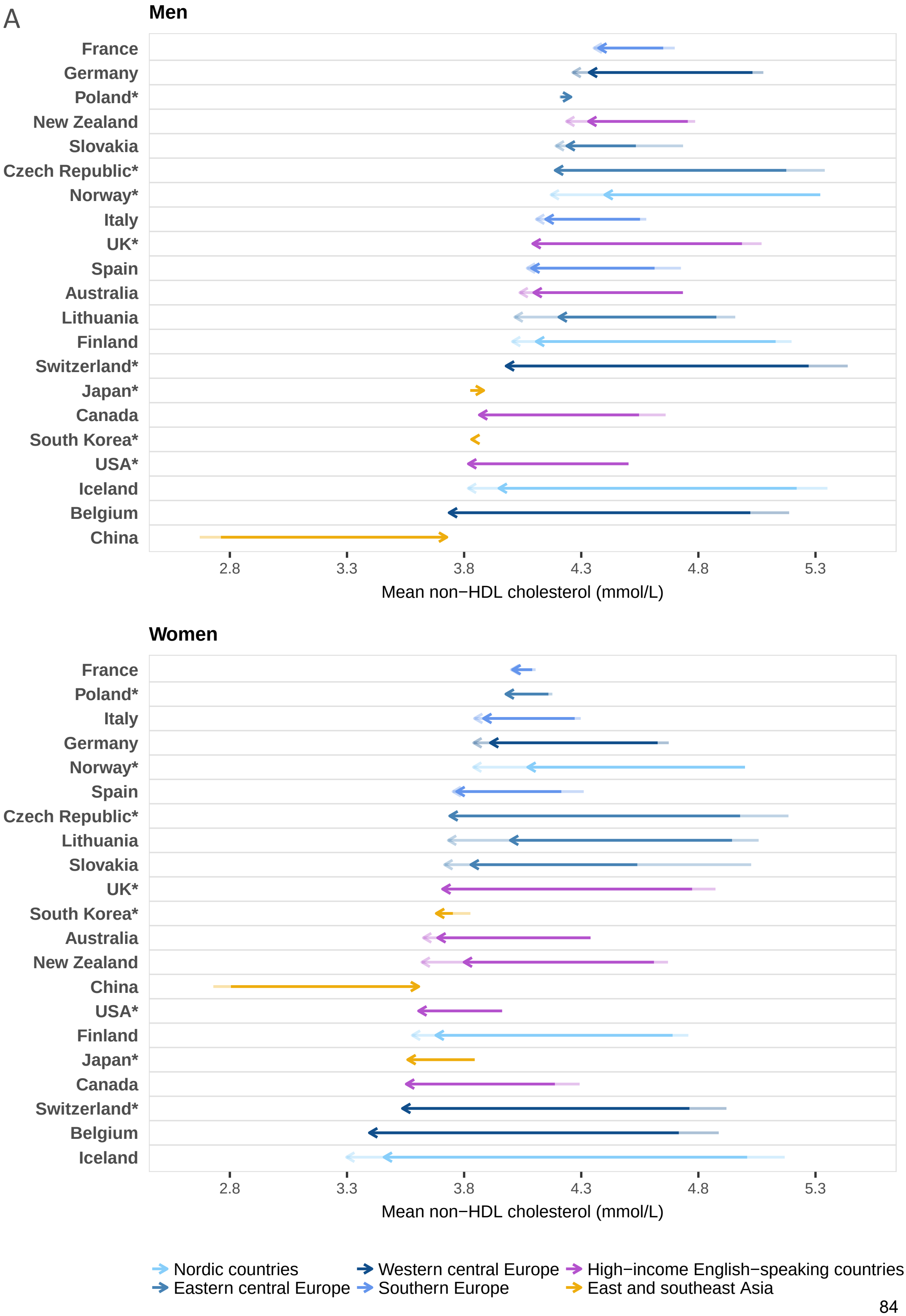
A
Men
France
Germany
Poland*
New Zealand
Slovakia
Czech Republic*
Norway*
Italy
UK*
Spain
Australia
Lithuania
Finland
Switzerland*
Japan*
Canada
South Korea*
USA*
Iceland
Belgium
China
2.8
3.3
3.8
4.3
4.8
5.3
Mean non−HDL cholesterol (mmol/L)
Women
France
Poland*
Italy
Germany
Norway*
Spain
Czech Republic*
Lithuania
Slovakia
UK*
South Korea*
Australia
New Zealand
China
USA*
Finland
Japan*
Canada
Switzerland*
Belgium
Iceland
2.8
3.3
3.8
4.3
4.8
5.3
Mean non−HDL cholesterol (mmol/L)
Nordic countries
Eastern central Europe
Western central Europe
Southern Europe
High−income English−speaking countries
East and southeast Asia

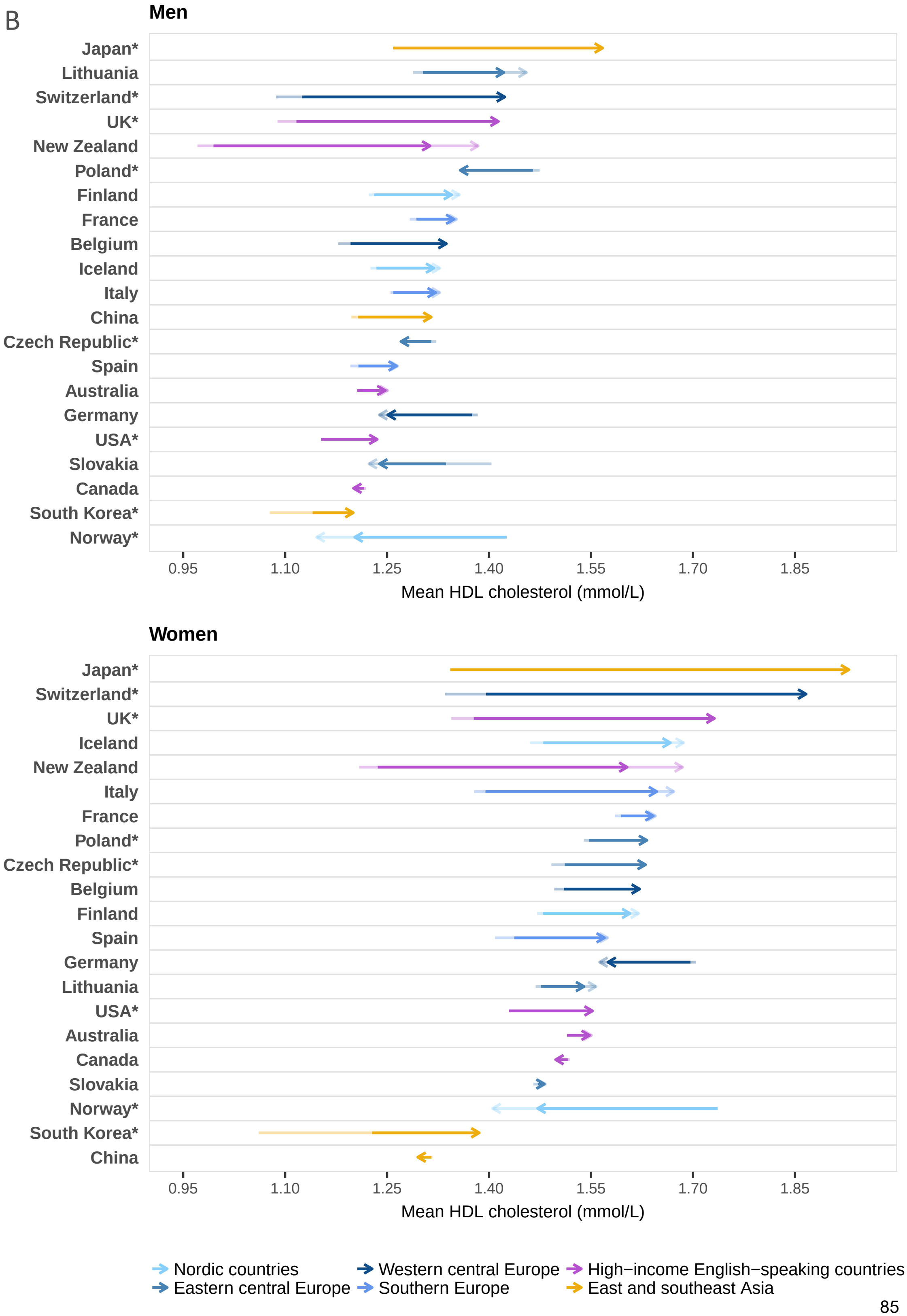
B
Men
Japan*
Lithuania
Switzerland*
UK*
New Zealand
Poland*
Finland
France
Belgium
Iceland
Italy
China
Czech Republic*
Spain
Australia
Germany
USA*
Slovakia
Canada
South Korea*
Norway*
0.95
1.10
1.25
1.40
1.55
1.70
1.85
Mean HDL cholesterol (mmol/L)
Women
Japan*
Switzerland*
UK*
Iceland
New Zealand
Italy
France
Poland*
Czech Republic*
Belgium
Finland
Spain
Germany
Lithuania
USA*
Australia
Canada
Slovakia
Norway*
South Korea*
China
0.95
1.10
1.25
1.40
1.55
1.70
1.85
Mean HDL cholesterol (mmol/L)
Nordic countries
Eastern central Europe
Western central Europe
Southern Europe
High−income English−speaking countries
East and southeast Asia

Supplementary Figure 6: Mean total-to-HDL cholesterol ratio at the beginning and end of analysis period by country and sex in people aged 40 to 59 years. The dark lines show the results for the period of data availability for each country (Supplementary Table 2); the lighter segments extend the trends to the period from 1980 to 2015 so that the start and end years are comparable across countries.

* Norway and USA had data prior to 1980 and Czech Republic, Japan, Poland, South Korea, Switzerland, UK and USA after 2015. This figure shows results from 1980 to 2015 so that the start and end years are comparable across countries.

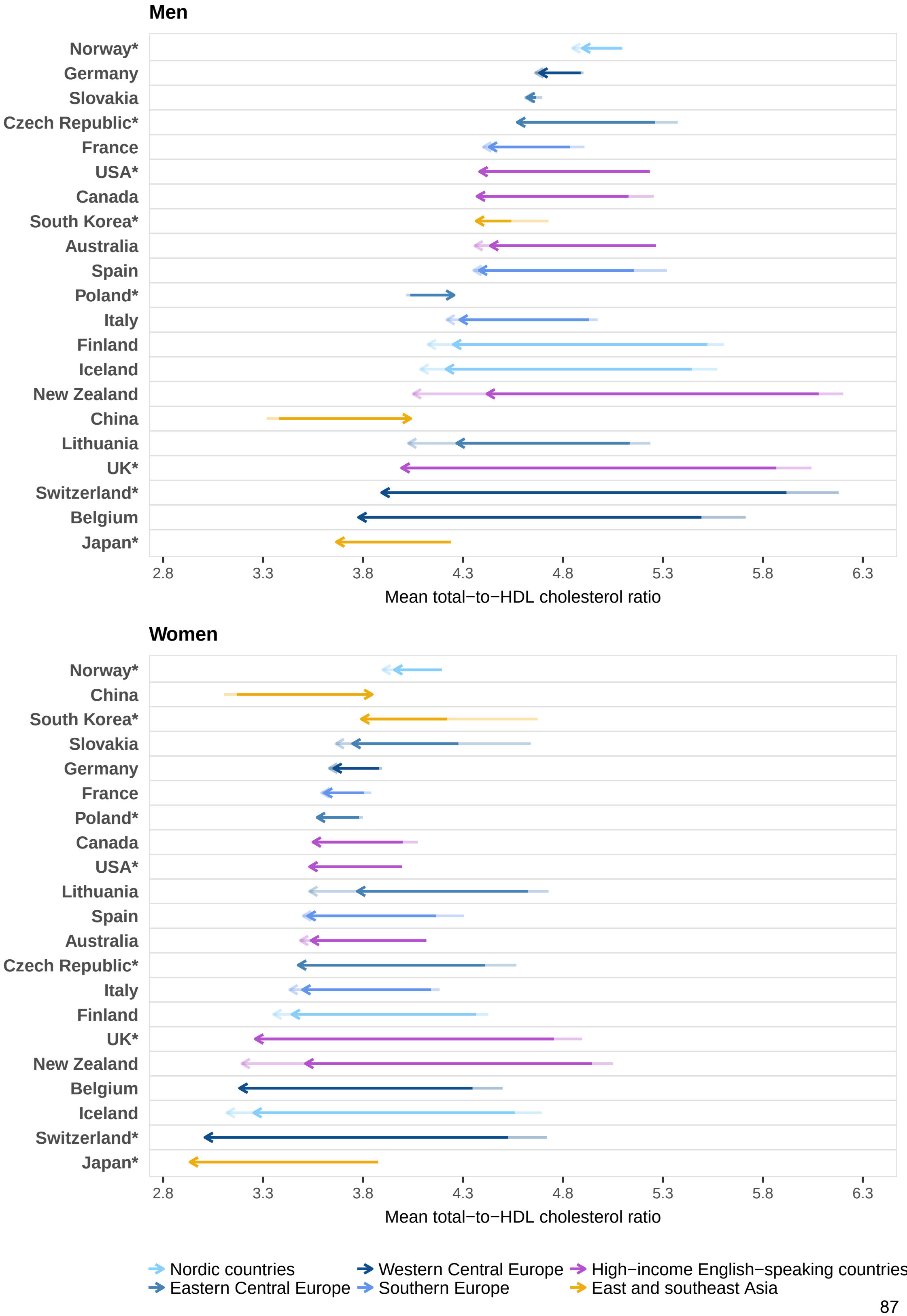
Men
Norway*
Germany
Slovakia
Czech Republic*
France
USA*
Canada
South Korea*
Australia
Spain
Poland*
Italy
Finland
Iceland
New Zealand
China
Lithuania
UK*
Switzerland*
Belgium
Japan*
2.8
3.3
3.8
4.3
4.8
5.3
5.8
6.3
Mean total−to−HDL cholesterol ratio
Women
Norway*
China
South Korea*
Slovakia
Germany
France
Poland*
Canada
USA*
Lithuania
Spain
Australia
Czech Republic*
Italy
Finland
UK*
New Zealand
Belgium
Iceland
Switzerland*
Japan*
2.8
3.3
3.8
4.3
4.8
5.3
5.8
6.3
Mean total−to−HDL cholesterol ratio
Nordic countries
Eastern Central Europe
Western Central Europe
Southern Europe
High−income English−speaking countries
East and southeast Asia

Supplementary Figure 7: Change per decade in mean (A) total cholesterol, (B) non-HDL cholesterol and (C) HDL cholesterol in people aged 40-59 years unadjusted versus adjusted for plasma-serum differences.

AUS = Australia; BEL = Belgium; CAN = Canada; CHE = Switzerland; CHN = China; CZE = Czech Republic; DEU = Germany; ESP = Spain; FIN = Finland; FRA = France; GBR = United Kingdom; ISL = Iceland; ITA = Italy; JPN = Japan; KOR = South Korea; LTU = Lithuania; NOR = Norway; NZL = New Zealand; POL = Poland; SVK = Slovakia; SWE = Sweden; THA = Thailand; USA = United States of America.

A

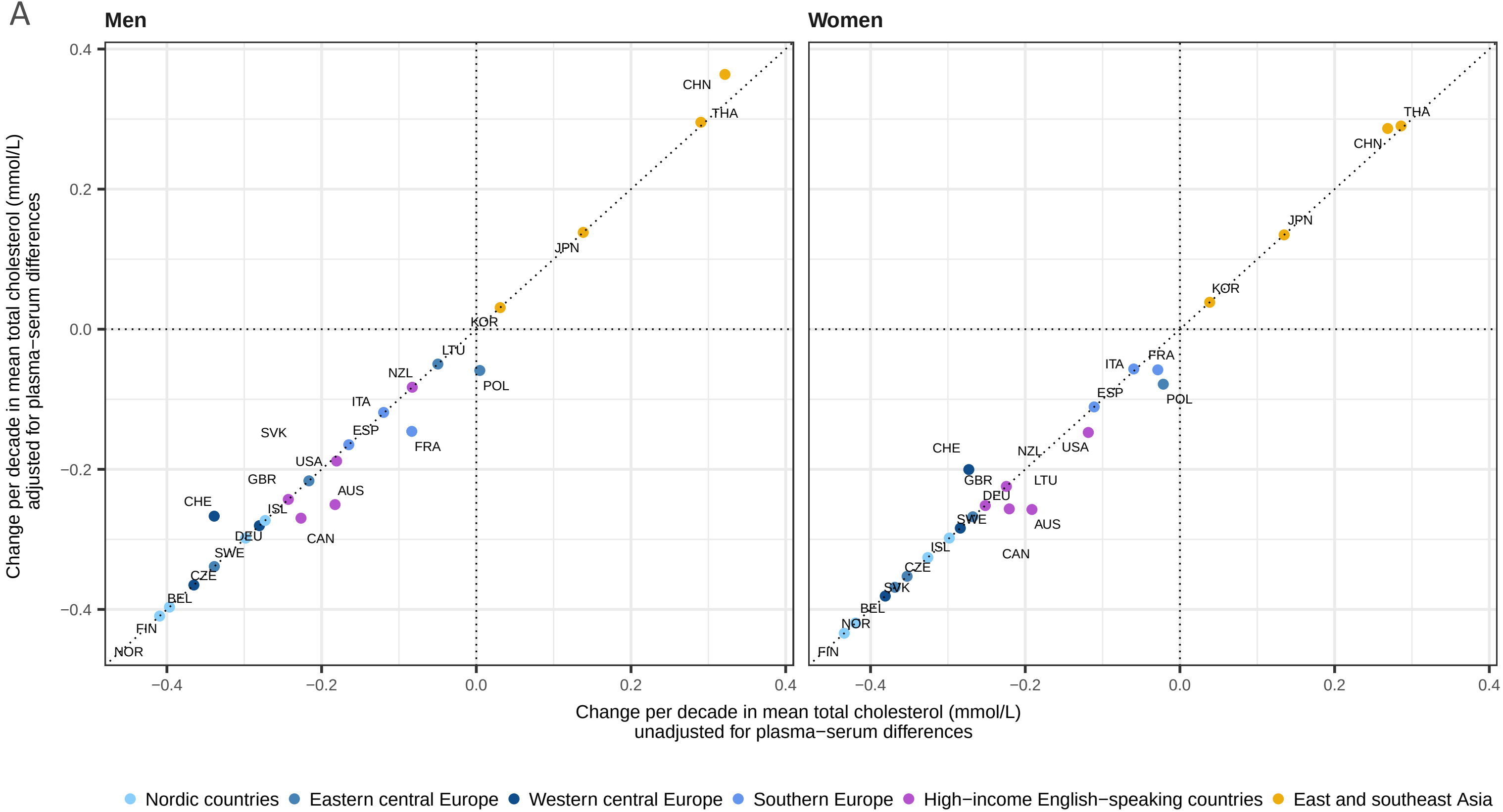

Men
Women
Change per decade in mean total cholesterol (mmol/L) adjusted for plasma−serum differences
Change per decade in mean total cholesterol (mmol/L) unadjusted for plasma−serum differences
0.4
0.2
0.0
−0.2
−0.4
CHN
THA
JPN
KOR
LTU
POL
NZL
ITA
SVK
ESP
FRA
USA
GBR
AUS
CHE
ISL
CAN
DEU
SWE
CZE
BEL
FIN
NOR
Nordic countries
Eastern central Europe
Western central Europe
Southern Europe
High−income English−speaking countries
East and southeast Asia

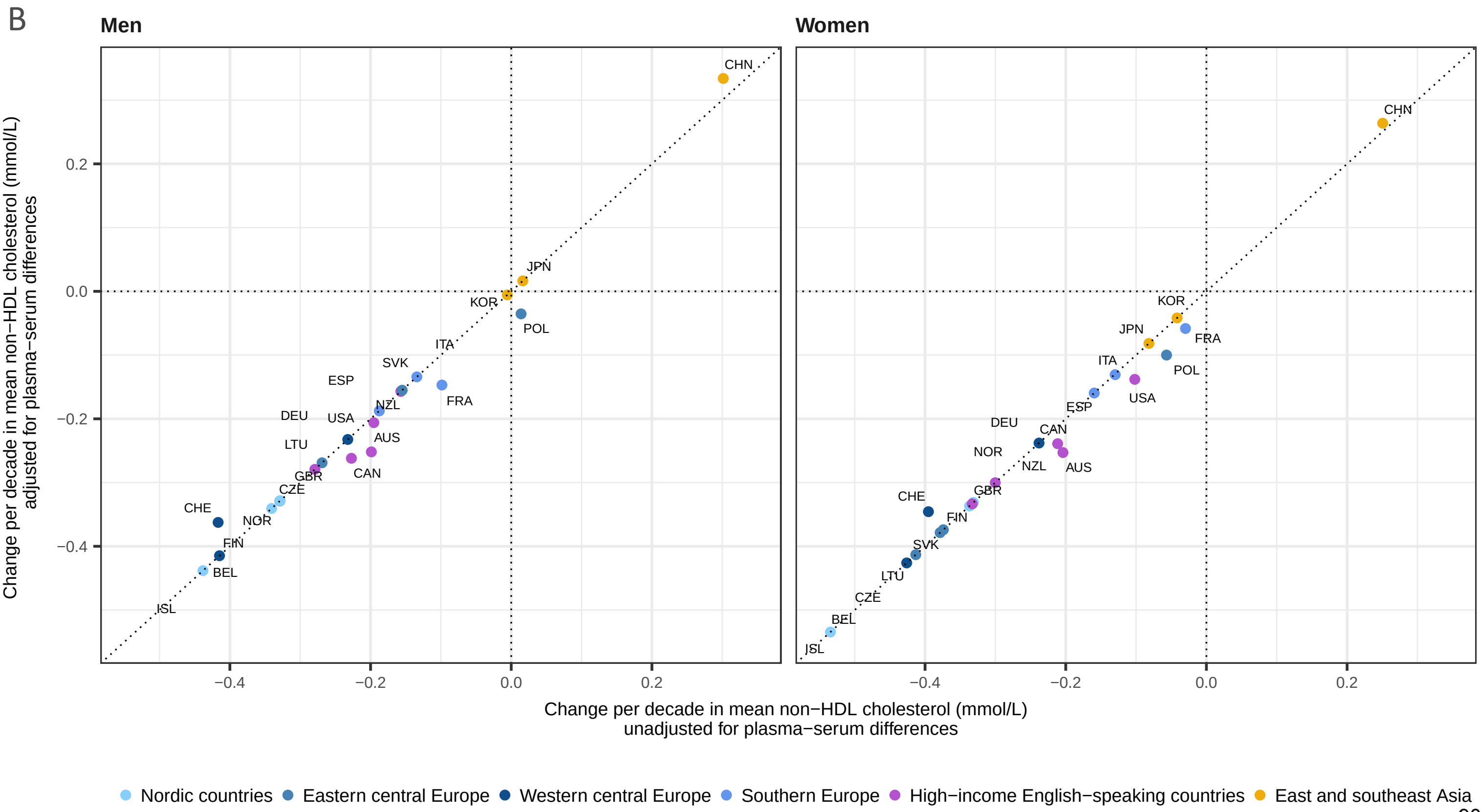

B
Men
Women
Change per decade in mean non−HDL cholesterol (mmol/L) adjusted for plasma−serum differences
Change per decade in mean non−HDL cholesterol (mmol/L) unadjusted for plasma−serum differences
0.2
0.0
−0.2
−0.4
−0.4
−0.2
0.0
0.2
CHN
JPN
KOR
POL
ITA
SVK
ESP
FRA
NZL
DEU
USA
AUS
LTU
CAN
GBR
CZE
CHE
NOR
FIN
BEL
ISL
Nordic countries
Eastern central Europe
Western central Europe
Southern Europe
High−income English−speaking countries
East and southeast Asia

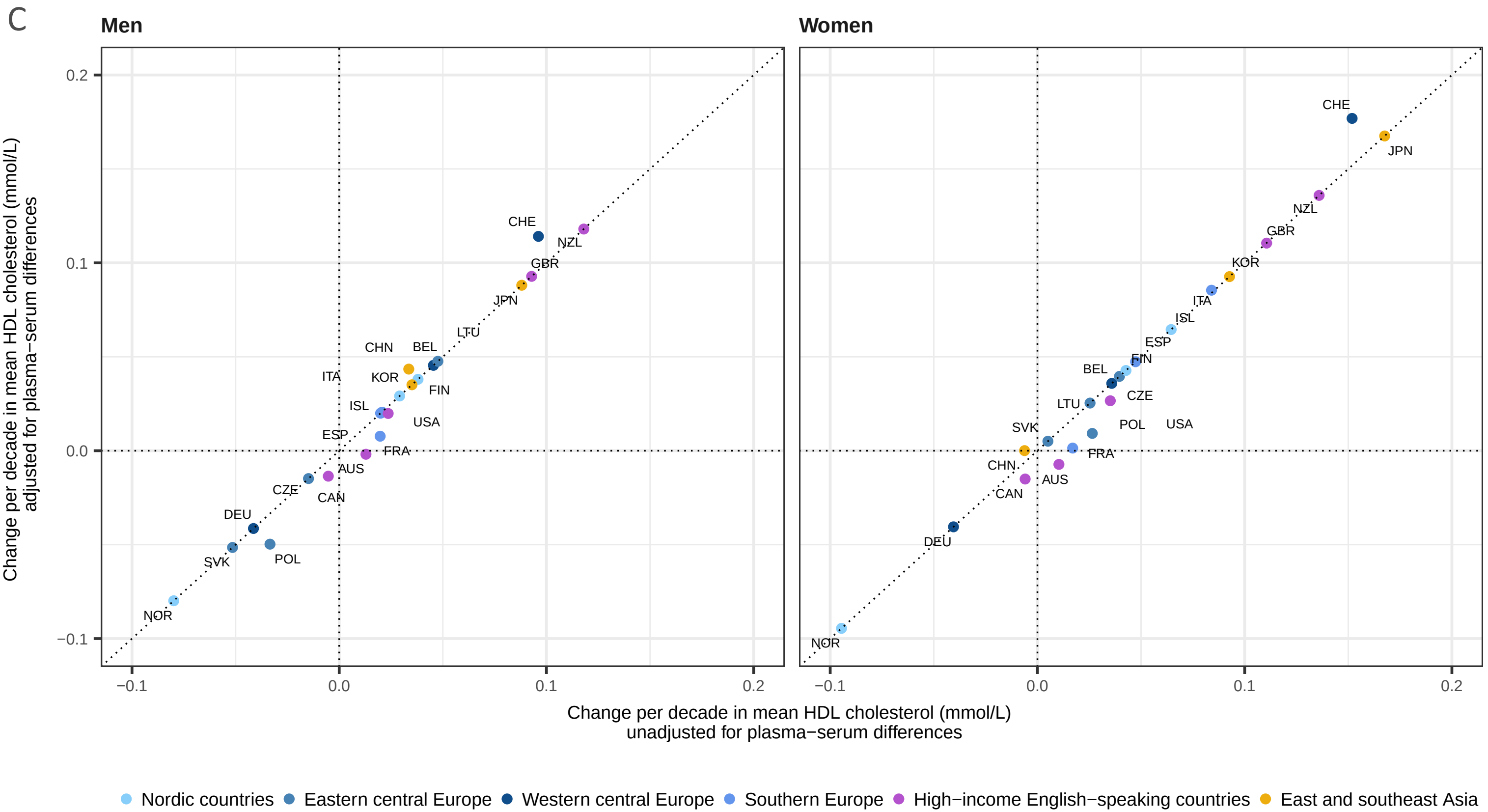
C
Men
Women
Change per decade in mean HDL cholesterol (mmol/L) adjusted for plasma−serum differences
Change per decade in mean HDL cholesterol (mmol/L) unadjusted for plasma−serum differences
0.2
0.1
0.0
−0.1
0.2
0.1
0.0
−0.1
CHE
NZL
GBR
JPN
LTU
CHN
BEL
ITA
KOR
FIN
ISL
USA
ESP
FRA
AUS
CZE
CAN
DEU
SVK
POL
NOR
CHE
JPN
NZL
GBR
KOR
ITA
ISL
ESP
FIN
BEL
CZE
LTU
POL
USA
SVK
FRA
CHN
AUS
CAN
DEU
NOR
Nordic countries
Eastern central Europe
Western central Europe
Southern Europe
High−income English−speaking countries
East and southeast Asia
